# Stereocontrolled Synthesis of Polysubstituted Housanes via *gem*-Bismetalated Cyclopropanes

**DOI:** 10.1021/jacs.6c02374

**Published:** 2026-03-17

**Authors:** Noam Orbach, Rahul Suresh, Ilan Marek

**Affiliations:** Schulich Faculty of Chemistry and the Resnick Sustainability Center for Catalysis, Technion−Israel Institute of Technology, Haifa 3200009, Israel

## Abstract

We report a stereoselective synthesis of polysubstituted housanes via bismetalated cyclopropane intermediates. The strategy relies on diastereoselective allylzincation of lithiated cyclopropenes followed by intramolecular cyclization, generating metalated housanes as key intermediates. Subsequent electrophile trapping enables efficient diversification of the housanes. Notably, this approach provides access to 2,3-dialkyl-substituted housanes, allowing the selective synthesis of both the diastereomers.

## Graphical abstract

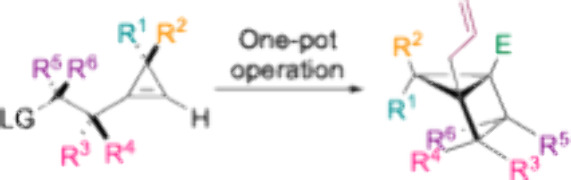

gem-Bismetalated species constitute a unique class of bifunctional organometallic intermediates with distinctive synthetic potential.^1,2^ Among the various methods available to access bismetalated species, a particularly powerful and well-established strategy is the allylzincation reaction of unsaturated metal species.^3,4^ This transformation is proposed to proceed via a zinca-ene-type allylation of an alkenyl metal species, to generate the corresponding *gem*-bismetalated species (Scheme 1a).^5,6^ When substituted allyl and alkenyl fragments are used, several new stereocenters can be formed with high levels of diastereoselectivity.^7,8^ It has also been shown that bismetalated species located in a 1,3-relationship to a methoxymethyl ether (R^1^ = OMOM) group are thermally labile and, upon heating, undergo intramolecular cyclization to afford metalated cyclopropanes.^9^ Trapping these intermediates with electrophiles provides trisubstituted cyclopropanes as single diastereomers (Scheme 1a).^10^ We hypothesized that this approach could be extended to more complex fused bicyclic systems, such as polysubstituted bicyclo[2.1.0]­pentanes (Scheme 1b),^11–13^ providing that both the allylmetalation step and the subsequent cyclization proceed with diastereocontrol.

**Scheme 1:**
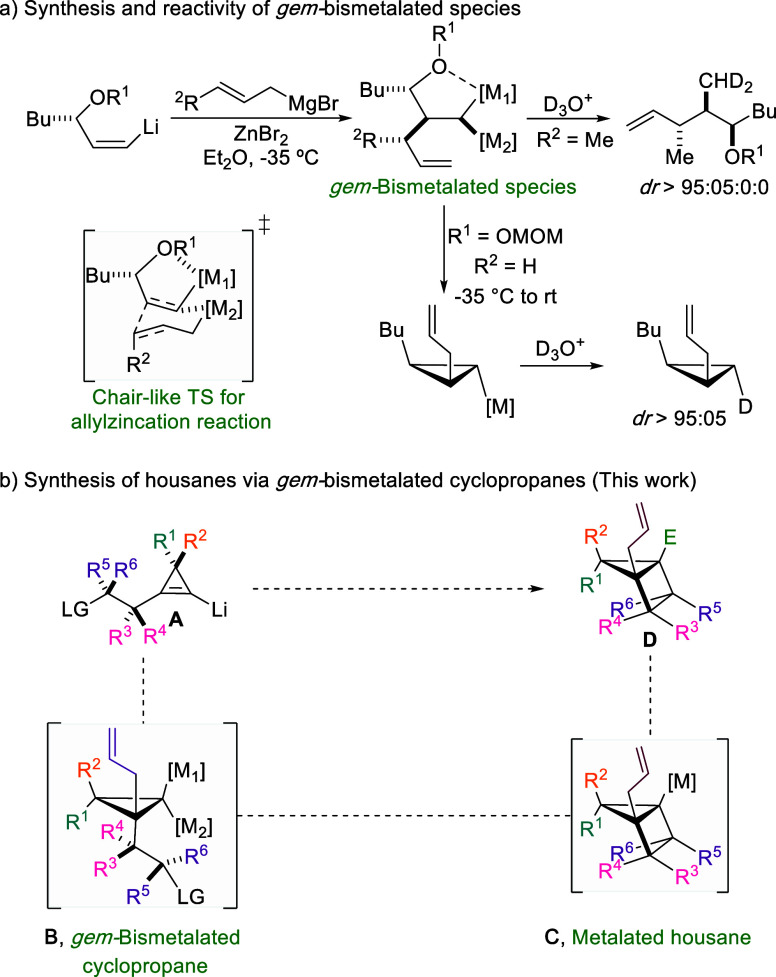
*gem*-Bismetalated Species and Their Application for the Synthesis of Strained Rings

Bicyclo­[2.1.0]­pentanes, commonly referred to as housanes, are highly strained fused bicyclic frameworks. These molecular scaffolds have attracted increasing attention in recent years, both as synthetic building blocks for the preparation of cyclopentanes and bicyclo[2.2.1]­heptanes^14–17^ and as bioisosteres of cyclopentane-containing biomolecules.^18,32^ The growing interest in housanes has created a need for the development of new synthetic methodologies. Several elegant strategies for their synthesis have been reported, including approaches based on [2 + 2] cycloaddition,^19–23^ inter- and intramolecular carbene insertion,^24–28^ and a variety of cyclization reactions.^14,29–32^ Despite these advances, stereoselective synthesis of polysubstituted bicyclo[2.1.0]­pentanes remains limited.

In particular, reliable access to both exo and endo substitution patterns on the four-membered rings, especially in densely substituted systems, continues to pose a major synthetic challenge.

Relying on the established methodology for the diastereoselective allylzincation of vinyl metals (Scheme 1a),^2–10^ including cyclopropenyl lithium derivatives,^33^ we sought to exploit such intermediates for the preparation of housanes (Scheme 1b). Specifically, we envisioned that lithiated cyclopropenes **A** bearing a suitable leaving group would undergo allylzincation to afford bismetalated cyclopropanes **B**, which could subsequently undergo a 4-exo-trig cyclization to generate metalated housanes **C** (Scheme 1b). These intermediates would provide a versatile platform for diversification through electrophile trapping or Pd-catalyzed cross-coupling reactions into functionalized housanes **D**, in a single-pot operation from readily accessible cyclopropene derivatives. Successful application of this strategy, however, requires overcoming several key challenges: (a) compatibility of the metalation and allylzincation of the cyclopropenes bearing leaving groups; (b) avoiding competing elimination or fragmentation pathways of the bismetalated species; (c) preservation of diastereocontrol as the degree of substitution increases; (d) reactivity of the sterically congested metalated housane toward electrophiles; and (e) stability of the highly strained housanes under Lewis acidic conditions. Addressing these challenges would provide a stereoselective, one-pot route to polysubstituted housanes from easily accessible cyclopropenes.

Our initial efforts focused on preparing cyclopropene derivatives via a Rh-catalyzed addition of diazo ester to alkynol derivatives.

These substrates can ultimately bear multiple stereocenters and were isolated as single diastereomers, as previously reported by our group^34^ (see Supporting Information for more details). The cyclopropenes were then converted to the corresponding iodides **1a**–**h** (Scheme 2) using either the classical Finkelstein exchange reaction (**1a**–**d**), modified Finkelstein conditions for **1e**,**f**,^35^ or Appel conditions for **1g**,**h** (see experimental procedure for all details).^36^ With convenient access to the starting materials, we examined the one-pot allylzincation–cyclization sequence using our model substrate **1a**. Iodide **1a** was first deprotonated using lithium diisopropylamide (LDA) at −78 °C, cleanly providing the cyclopropenyl lithium intermediate **2a**, with no detectable amount of elimination of the iodide leaving group. Subsequent addition of allylmagnesium bromide and zinc bromide led, after 5 hours at −40 °C, to an efficient and highly diastereoselective allylzincation providing the bismetalated species **3a**, which underwent cyclization to furnish metalated housane **5a**. When a less labile phosphate leaving group was employed, the intermediacy of a bis-metalated cyclopropane was confirmed by isolation of the hydrolyzed product **4** as a single diastereomer in good yield (Scheme 3a). To promote complete cyclization of **3a**, the reaction mixture was warmed to 30 °C, after which **6a** was obtained upon hydrolysis in a good yield with excellent diastereoselectivity. The relative configuration of **6a** was assigned by NMR analysis (see Supporting Information),^37^ indicating that the allylzincation reaction occurred in an antifashion relative to the ether substituent. This stereochemical outcome suggests a chelation model, in which the ether group shields one face of the cyclopropene, thereby directing allylzincation from the opposite face (Scheme 3a).^33^ The transformation proceeded with the same efficiency and selectivity for ethyl ether **1b** providing **6b**, supporting the chelation effect of the ether group (Scheme 3b).

**Scheme 2:**
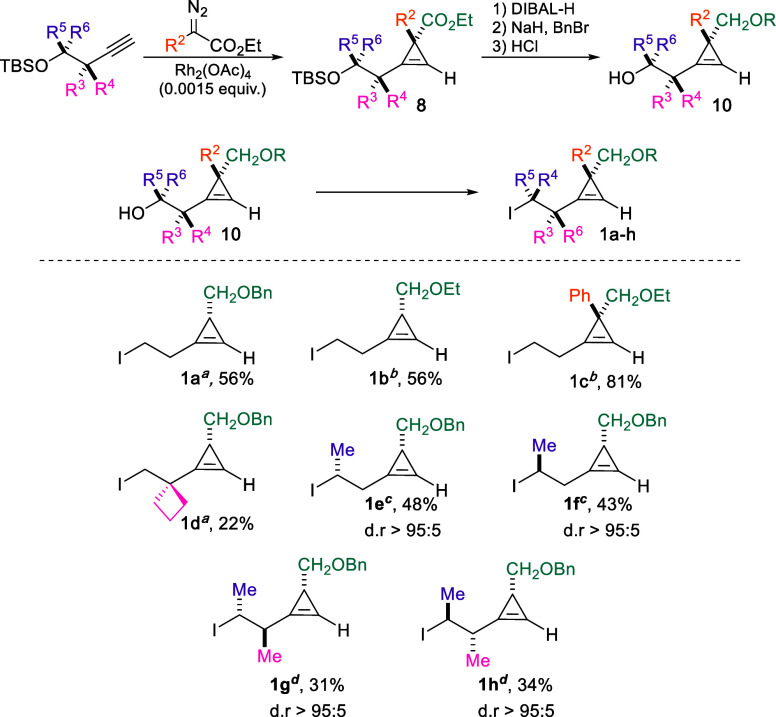
Synthesis of Starting Materials

a Et_3_N (1.5 equiv), MsCl (1.5 equiv), DCM (0.2 M), 1 h; NaI (5 equiv), acetone (0.2 M), 65 °C, 5 h.
b Starting with tosylates **11a**, **11b** - NaI (5 equiv), acetone (0.2 M), 65 °C, 5 h.
c Et_3_N (1.5 equiv), MsCl (1.5 equiv), DCM (0.2 M), 25 °C, 1 h; MeMgI (2 equiv), PhMe (0.2 M), 0 °C, 10 min.
d I_2_ (1.5 equiv), PPh_3_ (1.5 equiv), DCM (0.2 M), 0 °C, 1 h; *N*-methylimidazole (1.5 equiv), 0 °C, 1 h.

**Scheme 3:**
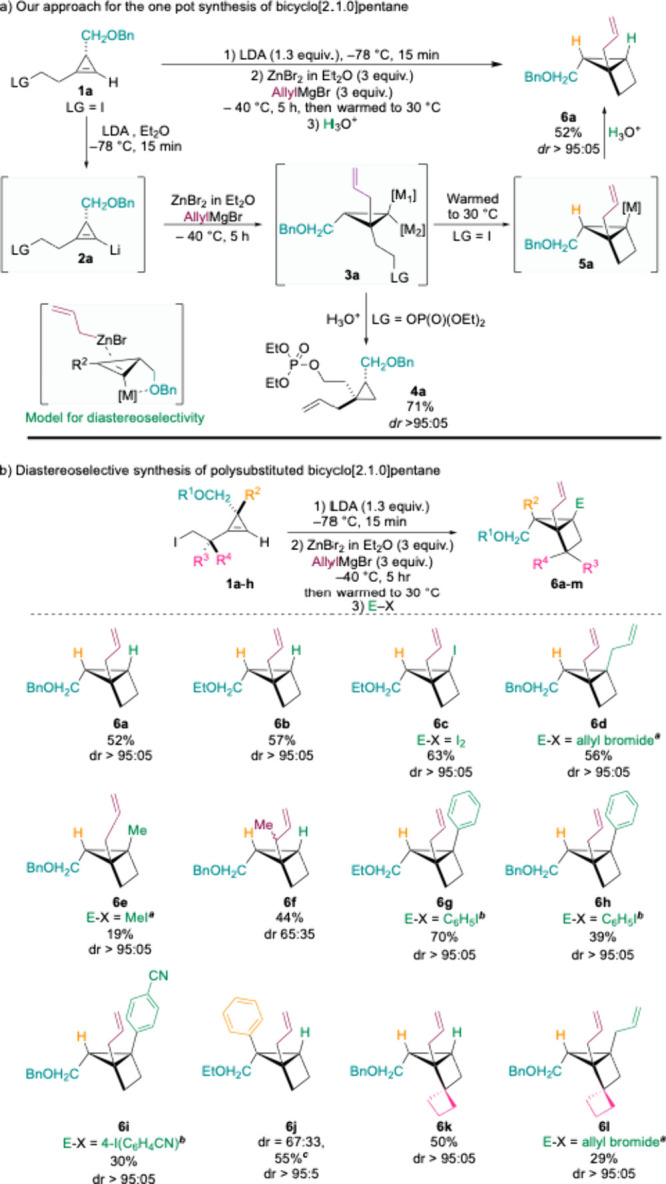
Synthesis and Functionalization of Metalated Housanes

a Transmetalation to CuCN·2LiCl (3 equiv) prior to electrophile addition.
b Pd­(PPh_3_)_4_ (0.15 equiv) was added followed by aryl iodide.
c Yield of the isolated major diastereomer after purification.

To probe the existence of a metalated housane, iodine was used as an electrophile, furnishing the corresponding iodo housane **6c** in good yield and with excellent diastereoselectivity. It should be noted that the reported chemical yields correspond to the overall one-pot sequence—metalation, allylzincation, cyclization, and electrophiles trapping—carried out consecutively. We next turned our attention to the more challenging carbon-based electrophiles. Achieving this required first enhancing the nucleophilicity of the metalated housane (**5a**, Scheme 3a) via transmetalation to copper by addition of CuCN·2LiCl. Subsequent addition of allyl bromide provided the disubstituted bridgehead housane **6d** in good yield with complete diastereoselectivity.^38^ Trapping with methyl iodide was also possible, giving housane **6e**, albeit in a low yield. We further explored the use of crotylzinc bromide, which introduces a new stereocenter in the product. In this case, housane **6f** was obtained with a disappointing diastereomeric ratio of 65:35 at the newly generated allylic center.

Next, we prepared several arylated housanes **6g**–**6i** with excellent diastereoselectivity and good to moderate yields, via one-pot metalation, allylzincation, cyclization, and Negishi Pd-catalyzed cross-coupling reactions.^39^ Notably, these cross couplings proceed with tertiary organometallic species adjacent to sterically demanding centers.^40,41^

To further increase the substitution pattern of the housane scaffold and generate two adjacent quaternary stereocenters, we performed the same reaction as that for cyclopropene **1c**, bearing an existing quaternary stereocenter on the cyclopropyl ring. Despite the high steric demand of the allylzincation step, the reaction proceeded smoothly, providing housane **6j**. However, the diastereoselectivity was only modest (67:33), which is likely attributed to steric interactions between the phenyl substituent and the incoming allylzinc species. Nonetheless, the diastereomers were easily separated, and the major diastereomer was cleanly isolated in 55% yield. This reaction was readily scaled to 200 mg of starting material with no detectable loss of either yield or selectivity. Housanes **6k** and **6l**, featuring a spirocyclobutyl group, were synthesized efficiently without erosion of diastereoselectivity or yield, even though allylzincation occurred adjacent to quaternary and tertiary centers. Moreover, the resulting metalated housane was trapped with allyl bromide to give the pentasubstituted housane **6l** containing three highly congested quaternary centers.

We next focused on the synthesis of housanes from cyclopropenes **1e**–**1h**, possessing secondary alkyl iodides. Under the standard reaction conditions, iodide **1e** furnished housane **6m** bearing a exo substituted methyl on the four-membered ring, with high yield and selectivity (Scheme 4). Remarkably, the reaction was successful for the complementary diastereomer **1f**, affording housane **6n** with a endo substituted methyl group. For this substrate, transmetalation to copper (CuCN·2LiCl) was necessary to facilitate the cyclization step. Access to both diastereomers offers a broadly applicable route to scaffolds featuring distinct three-dimensional architectures and exit vectors. This stereochemical versatility is particularly valuable when these frameworks serve as synthetic intermediates or bioisosteric replacements. Moreover, trapping with tosyl cyanide afforded tetrasubstituted housane **6o**, containing four distinct carbon substituents, albeit in low yield, due to the low reactivity of the electrophilic partner.

**Scheme 4:**
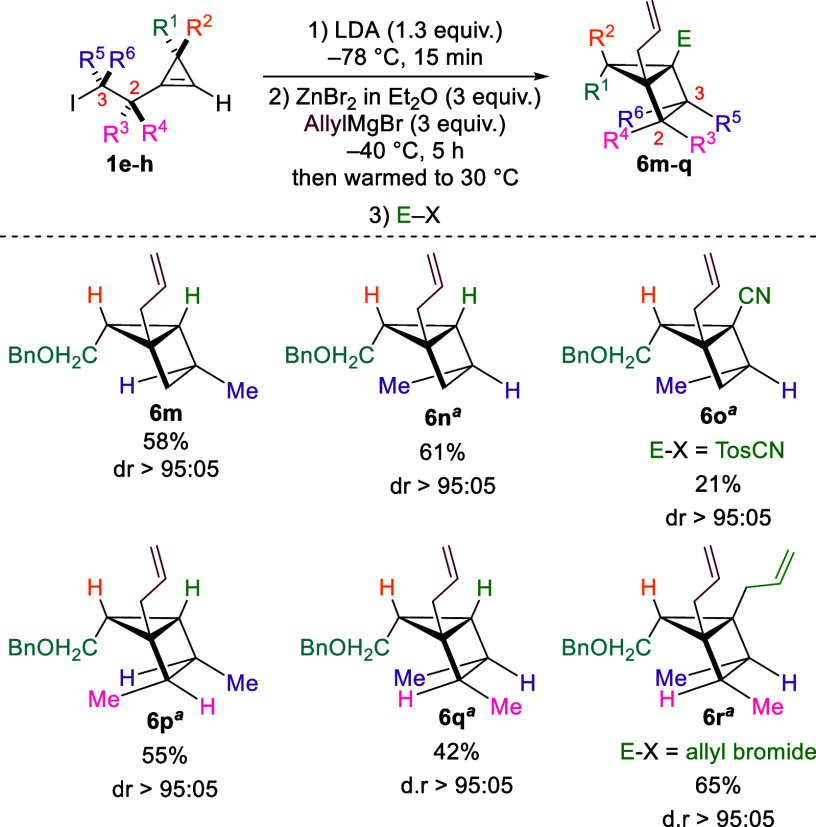
Diastereoselective Synthesis of Housanes Bearing Exo and Endo Substitution Patterns on the Four-Membered Ring

a Transmetalation to CuCN·2LiCl (3 equiv) was necessary for cyclization.

Finally, we applied the reaction conditions (including transmetalation to CuCN·2LiCl) to cyclopropenes **1g** and **1h**, bearing a 2,3-dimethyl substitution on the aliphatic chain (Scheme 4). Gratifyingly, housanes **6p** and **6q** were obtained as single diastereomers in decent overall yield, despite the high steric demand for both the allylzincation and the cyclization steps.

The relative configuration of the above housanes was determined from NMR analysis (see Supporting Information for more details).^37^ To our delight, the highly substituted metalated housane could be trapped with an allyl bromide, furnishing pentasubstituted housane **6r** with excellent yield and diastereoselectivity. To the best of our knowledge, this method provides unprecedented stereoselective access to both diastereomers of 2,3-disubstituted housanes on the four membered-ring.

To further highlight the synthetic utility of the allyl moiety resulting from allylmetalation of cyclopropenyl lithium species, we carried out postfunctionalization reactions. First, the iridium-catalyzed hydroboration oxidation of housane **6n** furnished the corresponding alcohol **7a** (Scheme 5). Alternatively, diallylated housane **6l** underwent ring-closing metathesis, providing tricyclo[4.2.1]­nonane **7b** featuring a spiro-cyclobutyl substituent. These transformations demonstrate that housanes are robust to late-transition metal-catalyzed transformations, facilitating diverse functionalization strategies.

**Scheme 5:**
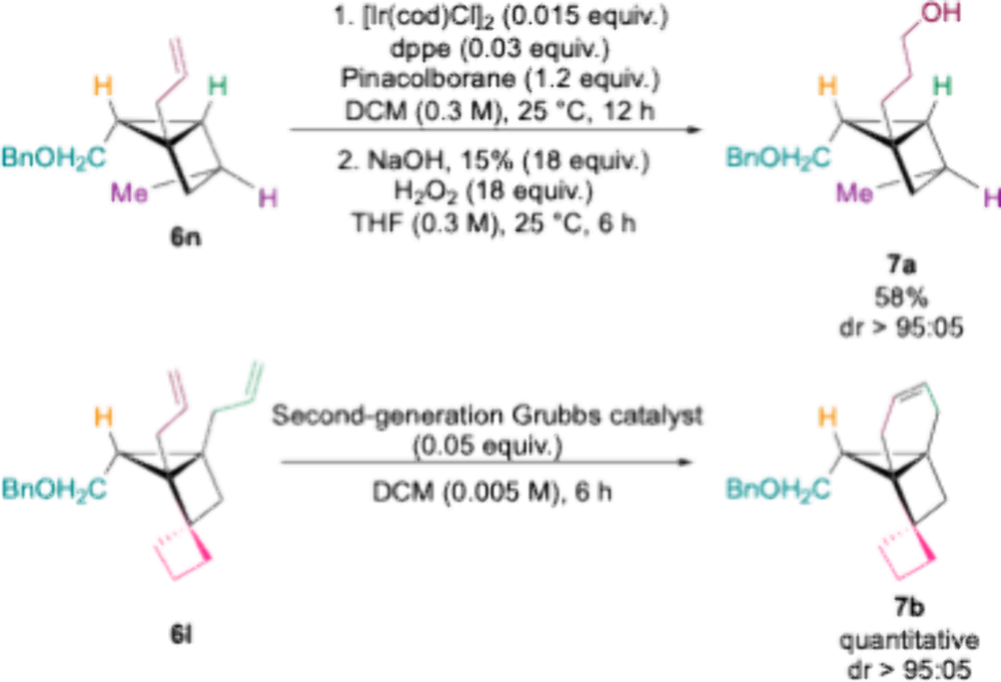
Metal-Catalyzed Functionalization of Polysubstituted Housanes

In conclusion, we have developed a new strategy for the synthesis of polysubstituted, stereodefined housanes via a one-pot sequence comprising metalation, allylzincation, 4-exo-trig cyclization of gem-bismetalated species, and electrophiles trapping. The formation of metalated housane intermediates enables subsequent functionalization with a diverse range of electrophiles, either directly or following transmetalation to copper or palladium intermediates. The protocol is also compatible with secondary iodides, enabling the stereoselective synthesis of housanes bearing either exo- or endo-substituents on the four-membered ring. Importantly, this strategy provides access to both diastereomers of 2,3-disubstituted housanes, highlighting its potential for the synthesis of more complex housane architectures.

## Supplementary Material



## Data Availability

The data underlying this study are available in the published article and its Supporting Information.

## Notes

General procedures, product characterization, and copies of NMR spectra (PDF)
