# Supplementary material for: Stereocontrolled Synthesis of Polysubstituted Housanes via gem-Bismetalated Cyclopropanes
Source: J Am Chem Soc. 2026 Mar 17;148(12):12502–6. doi: 10.1021/jacs.6c02374 (PMC13047527; doi:10.1021/jacs.6c02374)
Supplement: Supplementary file 1 [file ja6c02374_si_001.pdf]

Stereocontrolled Synthesis of Polysubstituted Housanes via Gem-Bismetalated Cyclopropanes

Noam Orbach, Rahul Suresh and Ilan Marek

Schulich Faculty of Chemistry and the Resnick Sustainability Center for Catalysis, Technion–Israel Institute of Technology, Haifa, 3200009, Israel

Contents

1. General information 2
2. Synthesis of starting materials 3
   2.1 General synthesis of cyclopropene esters. 3
   2.2 General procedure for the separation of diastereomeric cyclopropenyl esters. 4
   2.3 General procedure for the synthesis of cyclopropenyl methyl ether..7
   2.4 General procedure for synthesis of iodoethyl cyclopropene derivatives................................12
   2.1 General procedure for synthesis of polysubstituted bicyclo pentanes. 16
   2.2 Functionalization of polysubstituted housanes. 27
   2.3 Determination of the relative configuration of housanes 29
3. References. 42
4. NMR spectra of new compounds 43

1. General information

Unless otherwise stated, reactions were conducted in a flame-dried glassware under a positive pressure of argon. Et_2O and THF were dried from Pure-Solv® Purification System (Innovative Technology©). All organolithium reagents were purchased from Aldrich. All other reagents were purchased from Aldrich, Strem, Acros, and Alfa Aesar, and used as received. Thin-layer chromatography (TLC) was conducted with E. Merck silica gel 60 F254 pre-coated plates, (0.25 mm) and visualized by exposure to UV light (254 nm) or stained with anisaldehyde, CAM (solution of $Mo_7(NH_4)_6O_{24}$) and $(NH_4)_4Ce(SO_4)_4$), phosphomolybdic acid, or potassium permanganate. Column chromatography was performed using Fluka silica gel 60 Å (40- 63 µm, 230-400 mesh). All NMR spectra were recorded on Bruker spectrometers (AVIII 400 and AVII500), and reported relative to deuterated solvent signals and/or $SiMe_4$ as internal standard. Chemical shifts are reported in parts per million (ppm) with respect to the residual solvent signal $CDCl_3$ (^1H NMR: δ = 7.26; ^{13}C NMR: δ = 77.16). Peak multiplicities are reported as follows: s = singlet, bs = broad singlet, d = doublet, t = triplet, q = quartet, quin = quintet, sext = sextet, sep= septet, dd = doublet of doublets, td = triplet of doublets, ddd = doublet of doublets of doublets, m = multiplet. High-resolution mass spectra (HRMS) were obtained by the mass spectrometry facility at the Technion.

2. Synthesis of starting materials

2.1 General synthesis of cyclopropene esters. [1]

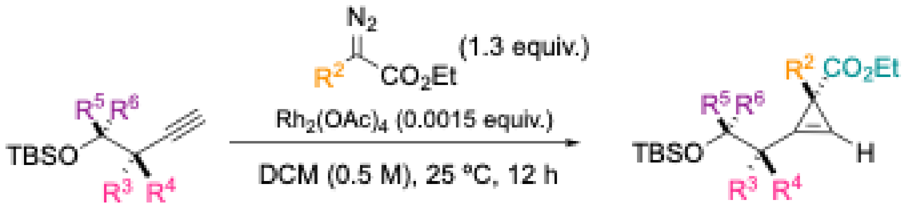

In an oven-dried round-bottom flask, the alkyne (1.0 equiv.) was dissolved in dry DCM (0.5 M). Dirhodium tetraacetate ($Rh_2(OAc)_4$, 0.0015 equiv.) was added to the solution. Diazoacetate (1.3 equiv.; typically as a 30% w/w solution in DCM) was then added over 12 hours using a syringe pump. The reaction progress was monitored by TLC (cyclopropenes show strong staining with $KMnO_4$). After completion, the reaction mixture was concentrated in vacuo. The crude product was purified by column chromatography on silica gel to afford the cyclopropene esters.

Cyclopropenes bearing multiple stereocenters were quickly filtered through a short plug of silica gel and used directly in the next step for diastereomer separation.

(±)-**Ethyl 2-(2-(tosyloxy)ethyl)cycloprop-2-ene-1-carboxylate (8a)**

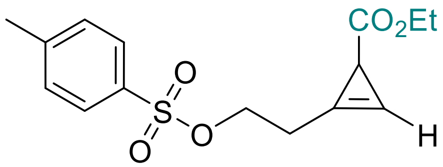

Scale, physical description, yield: 30 mmol, colorless oil, 68% yield.

Rf = 0.50 (PE/Et_2O 1:1).

^{1}H NMR (400 MHz, Chloroform-*d*): δ 7.79 (d, J = 8.2 Hz, 2H), 7.35 (d, J = 7.9 Hz, 2H), 6.45 (s, 1H), 4.22 (tt, J = 6.6, 3.2 Hz, 2H), 4.09 (qd, J = 7.2, 4.3 Hz, 2H), 2.87 (t, J = 6.7 Hz, 2H), 2.45 (s, 3H), 2.11 (s, 1H), 1.23 (t, J = 7.0 Hz, 3H).

^{13}C NMR (101 MHz, Chloroform-*d*): δ 175.93, 145.16, 132.95, 130.06, 128.09, 111.10, 97.69, 66.84, 60.56, 25.47, 21.79, 19.58, 14.43.

HRMS (APCI): m/z calculated for $C_{15}H_{19}O_5S$ $[M+H]^+$: 311.0948, found 311.0947.

(±)-**Ethyl 1-phenyl-2-(2-(tosyloxy)ethyl)cycloprop-2-ene-1-carboxylate (8b)**

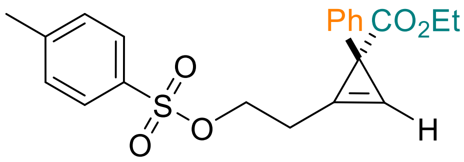

Scale, physical description, yield: 15 mmol, colorless oil, 62% yield.

Rf = 0.50 (PE/Et_2O 1:1).

^{1}H NMR (400 MHz, Chloroform-*d*): δ 7.77 – 7.71 (m, 2H), 7.35 – 7.30 (m, 2H), 7.30 – 7.25 (d, J = 1.0 Hz, 2H), 7.23 – 7.16 (m, 3H), 6.76 (t, J = 1.4 Hz,

1H), 4.23 (td, J = 6.7, 1.4 Hz, 2H), 4.13 (q, J = 7.1 Hz, 2H), 2.92 (tdd, J = 6.7, 3.0, 1.4 Hz, 2H), 2.45 (s, 3H), 1.21 (t, J = 7.1 Hz, 3H).

^{13}C NMR (101 MHz, Chloroform-*d*): δ 174.80, 145.10, 140.99, 132.97, 130.02, 128.18, 128.07, 126.56, 116.70, 100.13, 66.73, 61.04, 33.09, 24.96, 21.79, 14.38.

HRMS (APCI): m/z calculated for $C_{21}H_{23}O_5S$ $[M+H]^+$: 387.1261, found 387.1244.

(±)-Ethyl 2-(1-(((tert-butyldimethylsilyl)oxy)methyl)cyclobutyl)cycloprop-2-ene-1-carboxylate (8c)

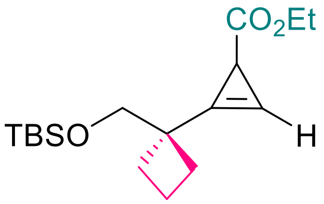

Scale, physical description, yield: 5.3 mmol, yellowish oil, 50% yield.

R*f* = 0.50 (PE/Et_2O 9:1).

^{1}H NMR (400 MHz, Chloroform-*d*): δ 6.34 (s, 1H), 4.21 – 4.04 (m, 2H), 3.66 (s, 2H), 2.21 (t, J = 4.5 Hz, 1H), 2.19 – 2.11 (m, 2H), 2.11 – 1.99 (m, 2H), 1.99 – 1.79 (m, 2H), 1.24 (t, J = 7.2 Hz, 3H), 0.91 (s, 9H), 0.05 (s, 6H).

^{13}C NMR (101 MHz, Chloroform-*d*): 176.79, 119.71, 93.23, 66.67, 60.27, 42.34, 27.99, 27.62, 25.98, 19.90, 18.43, 16.16, 14.50, -5.32.

HRMS (APCI): m/z calculated for $C_{17}H_{30}O_3Si$ $[M+H]^+$: 311.2044, found 311.2037.

2.2 General procedure for the separation of diastereomeric cyclopropenyl esters.

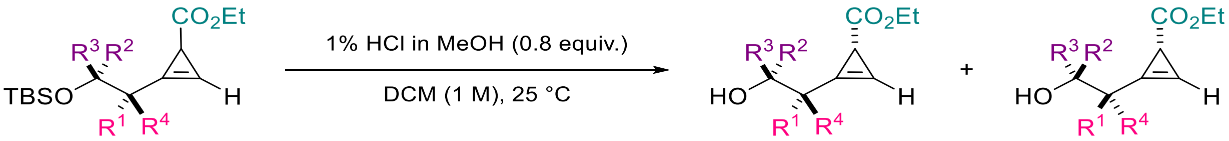

In an open-air flask, 1% solution of HCl in methanol (0.8 equiv.) was added dropwise to a solution of the protected alcohol (1 equiv.) in DCM (1 M). After complete consumption of the starting material (followed by $KMnO_4$ or p-anisaldehyde TLC-staining), solid $NaHCO_3$ was added and the reaction mixture was filtered through a short plug of silica gel. The crude product was further purified by slow silica gel column chromatography, using a gradual gradient up to 20% Et_2O in petroleum ether, to afford diastereoenriched fractions of the desired alcohols.

Before proceeding to the next steps, the alcohols were protected. In a flask open to air, the alcohol (1.0 equiv.) was dissolved in DCM (0.40 M), followed by the addition of Et_3N (3.0 equiv.). TBSCl (3.0 equiv.) was then added dropwise, and the reaction mixture was stirred until complete consumption of the alcohol (as monitored by TLC using $KMnO_4$ staining). Ethylamine was added, and the resulting gel was filtered through a short plug of silica gel. The reaction mixture was

filtered through a short plug of silica gel, and the crude product was used directly in the next step without further purification.

(±)-Ethyl (R)-2-((S)-2-hydroxypropyl)cycloprop-2-ene-1-carboxylate (9a)

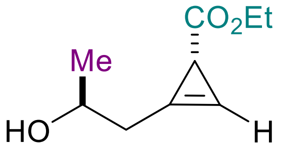

Scale, physical description, yield: 10.5 mmol, colorless oil, 37% yield.

Rf = 0.45 (PE/Et_2O 6:4).

^{1}H NMR (400 MHz, Chloroform-*d*): δ 6.53 (s, 1H), 4.16 (q, J = 7.1 Hz, 2H), 3.99 (dqd, J = 9.3, 6.2, 3.1 Hz, 1H), 3.62 – 3.44 (m, 1H), 2.77 (dd, J = 14.7, 9.1 Hz, 1H), 2.54 (ddd, J = 14.7, 3.2, 1.8 Hz, 1H), 2.17 (d, J = 1.4 Hz, 1H), 1.32 – 1.20 (m, 6H).

^{13}C NMR (101 MHz, Chloroform-*d*): δ 178.35, 112.07, 97.98, 64.69, 61.10, 35.84, 22.33, 19.60, 14.38.

HRMS (APCI): m/z calculated for $C_9H_{15}O_3$ $[M+H]^+$: 171.1016, found 171.1030.

(±)-Ethyl (R)-2-((R)-2-hydroxypropyl)cycloprop-2-ene-1-carboxylate (9b)

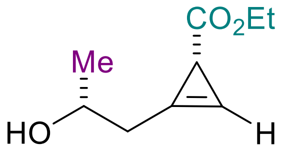

Scale, physical description, yield: 10.5 mmol, colorless oil, 44% yield.

Rf = 0.45 (PE/Et_2O 6:4).

^{1}H NMR (400 MHz, Chloroform-*d*): δ 6.51 (s, 1H), 4.15 (qd, J = 7.1, 1.0 Hz, 2H), 4.07 (dddd, J = 12.4, 6.0, 4.1, 1.4 Hz, 1H), 2.92 (m, 1H), 2.80 (ddt, J = 15.0, 4.0, 1.0 Hz, 1H), 2.59 (ddd, J = 15.0, 7.8, 0.8 Hz, 1H), 2.21 (s, 1H), 1.29 – 1.27 (m, 3H), 1.27 – 1.24 (m, 3H).

^{13}C NMR (101 MHz, Chloroform-*d*): δ 177.89, 112.83, 97.41, 66.14, 60.95, 35.26, 22.84, 20.07, 14.44.

HRMS (APCI): m/z calculated for $C_9H_{15}O_3$ $[M+H]^+$: 171.1016, found 171.1026.

(±)-Ethyl (R)-2-((2R,3S)-3-hydroxybutan-2-yl)cycloprop-2-ene-1-carboxylate (9c)

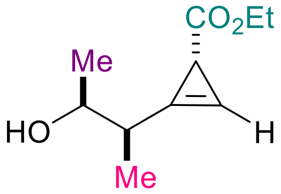

Scale, physical description, yield: 19.1 mmol, colorless oil, 13% yield.

Rf = 0.50 (PE/Et_2O 1:1).

^{1}H NMR (500 MHz, Chloroform-*d*): δ 6.47 (d, J = 1.4 Hz, 1H), 4.16 (qd, J = 7.2, 2.0 Hz, 2H), 3.63 – 3.53 (m, 1H), 2.86 (p, J = 6.9 Hz, 1H), 2.18 (d, J = 1.4 Hz, 1H), 1.30 – 1.23 (m, 6H), 1.08 (d, J = 6.9 Hz, 3H).

^{13}C NMR (126 MHz, Chloroform-*d*): δ 178.44, 115.69, 96.60, 69.44, 61.01, 39.36, 20.17, 18.22, 14.26, 14.25.

HRMS (APCI): m/z calculated for $C_{10}H_{17}O_3$ $[M+H]^+$: 185.1178, found 185.1188.

(±)-Ethyl (R)-2-((2S,3R)-3-hydroxybutan-2-yl)cycloprop-2-ene-1-carboxylate (9d)

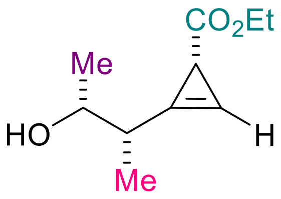

Scale, physical description, yield: 19.1 mmol, colorless oil, 22% yield.

Rf = 0.50 (PE/Et_2O 1:1).

^{1}H NMR (500 MHz, Chloroform-*d*): δ 6.54 (d, J = 1.4 Hz, 1H), 4.16 (qd, J = 7.1, 1.1 Hz, 2H), 3.76 (p, J = 6.2 Hz, 1H), 2.72 – 2.64 (m, 1H), 2.27 (d, J = 1.3 Hz, 1H), 1.29 – 1.26 (m, 3H), 1.26 – 1.23 (m, 6H).

^{13}C NMR (126 MHz, Chloroform-*d*): δ 178.05, 117.24, 97.57, 70.66, 61.01, 39.66, 21.16, 20.96, 16.17, 14.46.

HRMS (APCI): m/z calculated for $C_{10}H_{17}O_3$ $[M+H]^+$: 185.1178, found 185.1185.

2.3 General procedure for the synthesis of cyclopropenyl methyl ether.

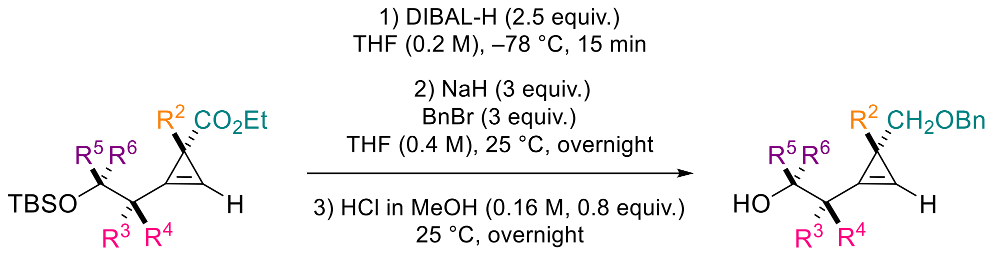

Step 1, Reduction:

In a flame-dried three-neck round-bottom flask, the cyclopropenyl ester (1.0 equiv.) was dissolved in THF (0.2 M). The reaction mixture was cooled to −78 °C (dry ice/acetone bath), and DIBAL-H (2.5 equiv.) was added dropwise. After complete consumption of the ester, as monitored by TLC ($KMnO_4$ staining; typically, 15 min), the reaction mixture was diluted with Et_2O and allowed to warm to 0 °C (ice bath). Water (0.1 equiv.) and 15% aqueous NaOH (0.1 equiv.) were added sequentially, followed by an additional portion of water (0.25 equiv.). The mixture was stirred at room temperature for 15 min, after which solid $MgSO_4$ was added and stirring was continued for 1 h. The resulting gel was filtered through a short plug of silica gel. The organic phase was dried over Na_2SO_4, filtered, and concentrated in vacuo. The crude product was used directly in the next step without further purification.

Step 2, Benzylation:

In a flame-dried three-neck round-bottom flask, the cyclopropenyl alcohol (1.0 equiv.) was dissolved in THF (0.4 M). The reaction mixture was cooled to 0 °C, and sodium hydride (3.0 equiv.) was added portionwise. After stirring for 15 min, benzyl bromide (3.0 equiv.) was added, and the reaction mixture was allowed to warm to room temperature and stirred overnight. After complete consumption of the alcohol, as monitored by TLC ($KMnO_4$ staining), the reaction was quenched with saturated aqueous NH_4Cl and extracted with Et_2O (3×). The combined organic extracts were dried over Na_2SO_4, filtered, and concentrated in vacuo. The crude product was used directly in the next step without further purification.

Step 3, Deprotection:

In a flask open to air, a 1% solution of HCl in methanol (0.8 equiv.) was added dropwise to a solution of the protected alcohol (1.0 equiv.) in DCM (1.0 M). After complete consumption of the starting material, as monitored by TLC ($KMnO_4$ or p-anisaldehyde staining), solid $NaHCO_3$ was added, and the reaction mixture was filtered through a short plug of silica gel. The crude product

was purified by column chromatography on silica gel to afford the desired cyclopropenylmethyl ether.

Note: For several compounds (e.g., **10e** and **10f**), benzyl alcohol co-elutes close to the desired product; therefore, careful column chromatography is required. If residual benzyl alcohol remains after purification, it can be easily removed in the next step, following conversion to the corresponding benzyl iodide.

(±)-2-(3-((Benzyloxy)methyl)cycloprop-1-en-1-yl)ethan-1-ol (10a)

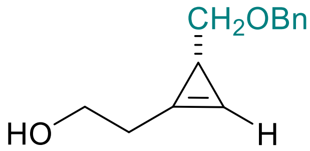

Scale, physical description, yield: 9 mmol, colorless oil, 52% yield (over three steps).

R*f* = 0.55 (PE/Et_2O 6:4).

^{1}H NMR (500 MHz, Chloroform-*d*): δ 7.28 (m, 1H), 7.26 – 7.18 (m, 4H), 6.57 (s, 1H), 4.46 (d, J = 11.9 Hz, 1H), 4.42 (d, J = 12.1 Hz, 1H), 4.06 – 3.97 (m, 1H), 3.77 (dd, J = 9.6, 3.4 Hz, 2H), 3.64 (ddd, J = 11.0, 7.3, 3.4 Hz, 1H), 2.97 (dd, J = 9.4, 6.8 Hz, 1H), 2.77 (ddd, J = 16.2, 7.2, 3.5 Hz, 1H), 2.58 (dddd, J = 16.2, 7.4, 3.8, 1.9 Hz, 1H), 1.70 (ddd, J = 6.8, 3.3, 1.4 Hz, 1H).

^{13}C NMR (126 MHz, Chloroform-*d*): δ 137.88, 128.56, 128.11, 127.91, 123.35, 103.20, 78.32, 73.22, 60.02, 30.11, 17.15.

HRMS (APCI): m/z calculated for $C_{13}H_{17}O_2$ $[M+H]^+$: 205.1229, found 205.1215.

(±)-(1-(3-((Benzyloxy)methyl)cycloprop-1-en-1-yl)cyclobutyl)methanol (10b)

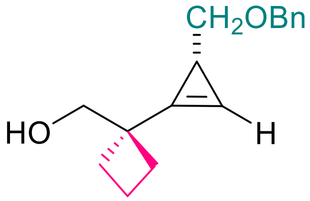

Scale, physical description, yield: 4.6 mmol, colorless oil, 17% yield (over three steps).

R*f* = 0.50 (PE/Et_2O 6:4).

^{1}H NMR (500 MHz, Chloroform-*d*): δ 7.32 – 7.26 (m, 2H), 7.26 – 7.19 (m, 3H), 6.56 (t, J = 1.0 Hz, 1H), 4.45 (d, J = 12.0 Hz, 1H), 4.42 (d, J = 12.0 Hz, 1H), 3.76 (ddd, J = 9.4, 3.4, 0.8 Hz, 1H), 3.67 (d, J = 11.3 Hz, 1H), 3.58 (d, J = 11.1 Hz, 1H), 2.97 (dd, J = 9.3, 6.6 Hz, 1H), 2.20 – 2.09 (m, 1H), 2.06 – 1.95 (m, 2H), 1.90 (ttd, J = 7.5, 4.8, 2.5 Hz, 3H), 1.81 (ddd, J = 6.7, 3.3, 1.4 Hz, 1H).

^{13}C NMR (126 MHz, Chloroform-*d*): δ 137.85, 129.99, 128.55, 128.12, 127.90, 100.89, 78.04, 73.22, 67.78, 42.67, 29.37, 29.33, 18.26, 15.99.

HRMS (APCI): m/z calculated for $C_{16}H_{21}O_2$ $[M+H]^+$: 245.1536, found 245.1535.

(±)-(R)-1-((S)-3-((Benzyloxy)methyl)cycloprop-1-en-1-yl)propan-2-ol (10c)

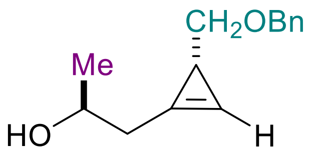

Scale, physical description, yield: 3.6 mmol, colorless oil, 79% yield (over four steps).

R*f* = 0.45 (PE/Et_2O 6:4).

^{1}H NMR (400 MHz, Chloroform-*d*): δ 7.31 – 7.26 (m, 3H), 7.25 – 7.18 (m, 2H), 6.61 (s, 1H), 4.45 (s, 1H), 4.44 (s, 1H), 4.09 – 3.97 (m, 2H), 3.73 (dd, J = 9.5, 3.3 Hz, 1H), 3.04 (dd, J = 9.5, 6.3 Hz, 1H), 2.64 – 2.57 (m, 2H), 1.68 (ddd, J = 6.4, 3.3, 1.4 Hz, 1H), 1.15 (d, J = 6.2 Hz, 3H).

^{13}C NMR (101 MHz, Chloroform-*d*): δ 137.92, 128.52, 128.08, 127.84, 122.50, 103.94, 77.40, 73.09, 65.56, 36.20, 22.82, 17.34.

HRMS (APCI): m/z calculated for $C_{14}H_{19}O_2$ $[M+H]^+$: 219.1380, found 219.1393.

(±)-(R)-1-((R)-3-((Benzyloxy)methyl)cycloprop-1-en-1-yl)propan-2-ol (10d)

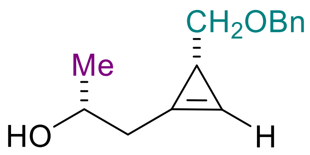

Scale, physical description, yield: 7.6 mmol, colorless oil, 72% yield (over four steps).

R*f* = 0.45 (PE/Et_2O 6:4).

^{1}H NMR (400 MHz, Chloroform-*d*): δ 7.28 – 7.24 (m, 2H), 7.23 – 7.15 (m, 3H), 6.54 (s, 1H), 4.43 (d, J = 1.4 Hz, 2H), 4.22 (d, J = 4.0 Hz, 1H), 3.81 (ddt, J = 9.0, 6.3, 2.7 Hz, 1H), 3.73 (dd, J = 9.4, 3.4 Hz, 1H), 2.89 (dd, J = 9.4, 7.1 Hz, 1H), 2.70 (dd, J = 15.6, 2.9 Hz, 1H), 2.39 (ddd, J = 15.6, 8.5, 1.8 Hz, 1H), 1.69 (ddd, J = 7.3, 3.4, 1.3 Hz, 1H), 1.17 (d, J = 6.3 Hz, 3H).

^{13}C NMR (101 MHz, Chloroform-*d*): δ 138.01, 128.53, 128.05, 127.83, 123.51, 102.95, 78.60, 73.12, 65.54, 36.27, 23.17, 17.60.

HRMS (APCI): m/z calculated for $C_{14}H_{19}O_2$ $[M+H]^+$: 219.1380, found 219.1397.

(±)-(2S,3R)-3-((R)-3-((Benzyloxy)methyl)cycloprop-1-en-1-yl)butan-2-ol (10e)

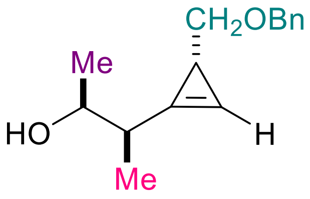

Scale, physical description, yield: 2.5 mmol, colorless oil, 85% yield. (over four steps).

R*f* = 0.50 (PE/Et_2O 6:4).

^{1}H NMR (500 MHz, Chloroform-*d*): δ 7.30 – 7.24 (m, 4H), 7.23 – 7.18 (m, 1H), 6.60 (s, 1H), 4.44 (s, 2H), 3.76 (d, J = 6.4 Hz, 1H), 3.70 (ddd, J = 9.5, 3.4, 0.8 Hz, 1H), 3.66 (p, J = 6.3 Hz, 1H), 3.07 (dd, J = 9.4, 6.2 Hz, 1H), 2.71 (p, J = 6.8 Hz, 1H), 1.70 (ddd, J = 6.2, 3.4, 1.4 Hz, 1H), 1.11 (d, J = 6.3 Hz, 3H), 1.06 (d, J = 7.0 Hz, 3H).

^{13}C NMR (126 MHz, Chloroform-*d*): δ 137.96, 128.52, 128.12, 127.83, 126.63, 103.34, 77.37, 73.10, 70.33, 39.80, 21.46, 16.98, 15.53.

HRMS (APCI): m/z calculated for $C_{15}H_{21}O_2$ $[M+H]^+$: 233.1536, found 233.1559.

(±)-(2R,3S)-3-((R)-3-((Benzyloxy)methyl)cycloprop-1-en-1-yl)butan-2-ol (10f)

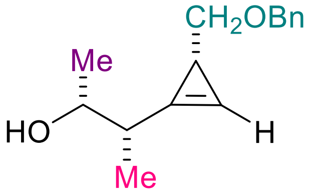

Scale, physical description, yield: 4.2 mmol, colorless oil, 61% yield. (over four steps).

R*f* = 0.50 (PE/Et_2O 6:4).

^{1}H NMR (500 MHz, Chloroform-*d*): δ 7.30 – 7.24 (m, 4H), 7.24 – 7.17 (m, 1H), 6.60 (s, 1H), 4.45 (d, J = 1.9 Hz, 2H), 4.15 (dt, J = 4.7, 2.1 Hz, 1H), 3.72 (ddd, J = 9.4, 3.4, 0.9 Hz, 1H), 3.56 (tdd, J = 6.8, 5.2, 1.7 Hz, 1H), 2.98 (ddd, J = 8.7, 6.7, 1.9 Hz, 1H), 2.55 – 2.44 (m, 1H), 1.79 (ddt, J = 6.5, 3.0, 1.5 Hz, 1H), 1.18 (d, J = 6.3 Hz, 3H), 1.11 (d, J = 7.0 Hz, 3H).

^{13}C NMR (126 MHz, Chloroform-*d*): δ 137.94, 128.46, 128.43, 127.96, 127.73, 103.01, 78.17, 73.05, 70.06, 40.26, 21.12, 18.89, 15.96.

HRMS (APCI): m/z calculated for $C_{15}H_{21}O_2$ $[M+H]^+$: 233.1536, found 233.1525.

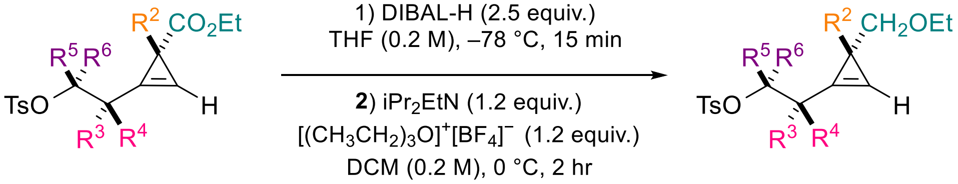

Step 1, Reduction:

In a flame-dried three-neck round-bottom flask, the cyclopropenyl ester (1.0 equiv.) was dissolved in THF (0.2 M). The reaction mixture was cooled to −78 °C (dry ice/acetone bath), and DIBAL-H (2.5 equiv.) was added dropwise. After complete consumption of the ester, as monitored by TLC ($KMnO_4$ staining; typically, 15 min), the reaction mixture was diluted with Et_2O and allowed to warm to 0 °C (ice bath). Water (0.1 equiv.) and 15% aqueous NaOH (0.1 equiv.) were added sequentially, followed by an additional portion of water (0.25 equiv.). The mixture was stirred at room temperature for 15 min, after which solid $MgSO_4$ was added and stirring was continued for 1 hr. The resulting gel was filtered through a short plug of silica gel. The organic phase was dried over Na_2SO_4, filtered, and concentrated in vacuo. The crude product was used directly in the next step without further purification.

Step 2, Ethylation:

In a flame-dried three-neck round-bottom flask, the cyclopropenyl alcohol (1.0 equiv.) was dissolved in dry DCM (0.2 M). The reaction mixture was cooled to 0 °C, and N,N-

diisopropylethylamine (Hünig's base; 1.2 equiv.) was added. After stirring for 10 min, triethyloxonium tetrafluoroborate (Meerwein's salt; 1.2 equiv.) was added portion wise, and the reaction mixture was stirred for 2 h. After complete consumption of the alcohol, as monitored by TLC ($KMnO_4$ staining), the reaction was quenched with saturated aqueous NH_4Cl and extracted with DCM (3×). The combined organic extracts were dried over Na_2SO_4, filtered, and concentrated in vacuo. The crude product was used directly in the next step without further purification.

(±)-2-(3-(Ethoxymethyl)cycloprop-1-en-1-yl)ethyl 4-methylbenzenesulfonate (11a)

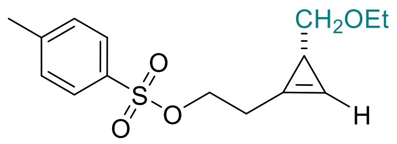

Scale, physical description, yield: 3 mmol, colorless oil, 73% yield.

R*f* = 0.55 (PE/Et_2O 1:1).

^{1}H NMR (500 MHz, Chloroform-*d*): δ 7.79 (d, J = 8.4 Hz, 2H), 7.35 (d, J = 8.0s Hz, 2H), 6.74 (s, 1H), 4.23 (t, J = 6.7 Hz, 2H), 3.40 (qd, J = 7.0, 1.7 Hz, 2H), 3.26 (dd, J = 9.9, 5.0 Hz, 1H), 3.19 (dd, J = 9.9, 5.0 Hz, 1H), 2.85 (tdd, J = 6.5, 4.9, 1.3 Hz, 2H), 2.45 (s, 3H), 1.64 (tt, J = 6.5, 3.3 Hz, 1H), 1.16 (t, J = 7.0 Hz, 3H).

^{13}C NMR (126 MHz, Chloroform-*d*): δ 145.02, 133.13, 130.00, 128.07, 120.69, 106.22, 77.67, 67.73, 65.95, 26.65, 21.79, 17.89, 15.40.

HRMS (APCI): m/z calculated for $C_{15}H_{21}O_4S$ $[M+H]^+$: 297.1155, found 297.1133.

(±)-2-(3-(Ethoxymethyl)-3-phenylcycloprop-1-en-1-yl)ethyl 4-methylbenzenesulfonate (11b)

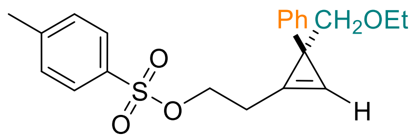

Scale, physical description, yield: 5 mmol, colorless oil, 68% yield.

R*f* = 0.55 (PE/Et_2O 1:1).

^{1}H NMR (400 MHz, Chloroform-*d*): δ 7.76 (d, J = 8.3 Hz, 2H), 7.33 (d, J = 7.7 Hz, 2H), 7.27 – 7.22 (m, 2H), 7.18 – 7.10 (m, 3H), 6.84 (s, 1H), 4.24 (td, J = 6.7, 0.9 Hz, 2H), 3.85 (d, J = 10.1 Hz, 1H), 3.72 (d, J = 10.1 Hz, 1H), 3.47 (qd, J = 7.0, 2.9 Hz, 2H), 2.96 – 2.75 (m, 2H), 2.45 (s, 3H), 1.17 (t, J = 7.0 Hz, 3H).

^{13}C NMR (101 MHz, Chloroform-*d*): δ 146.07, 144.99, 133.16, 130.00, 128.13, 128.06, 126.27, 125.46, 120.21, 105.53, 77.38, 67.59, 66.10, 29.30, 25.28, 21.79, 15.37.

HRMS (APCI): m/z calculated for $C_{21}H_{25}O_4S$ $[M+H]^+$: 373.1468, found 373.1467.

2.4 General procedure for synthesis of iodoethyl cyclopropene derivatives.

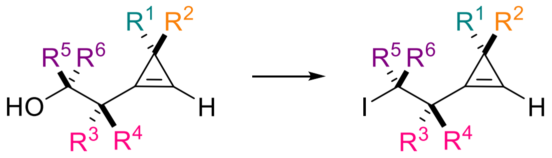

a) Conditon A
1) Et_3N (1.5 equiv.), MsCl (1.5 equiv.), DCM (0.2 M), 1 hr
2) NaI (5 equiv.), Acetone (0.2 M), 65 °C, 5 hr

b) Conditon B
1) Et_3N (1.5 equiv.), MsCl (1.5 equiv.), DCM (0.2 M), 25 °C, 1 hr
2) MeMgI (2 equiv.), PhMe (0.2 M), 0 °C, 10 min

c) Conditon C
1) I_2 (1.5 equiv.), $P(Ph)_3$ (1.5 equiv.), DCM (0.2 M), 0 °C, 1 hr
2) N-methylimidazole (1.5 equiv.), 0 °C, 1 hr

Condition A: [2]

In a flame-dried round-bottom flask, the alcohol (1.0 equiv.) was dissolved in dry DCM (0.10 M). The reaction mixture was cooled to 0 °C, Et_3N (1.5 equiv.) was added, followed by dropwise addition of MsCl (1.5 equiv.). The mixture was stirred at this temperature for 1 h. The reaction was quenched with saturated aqueous $NaHCO_3$ and extracted with DCM (3 ×). The combined organic extracts were dried over Na_2SO_4, filtered, and concentrated under reduced pressure. The crude mesylate was dissolved in acetone (0.20 M), and NaI (5.0 equiv.) was added. The reaction mixture was refluxed for 5 h. Acetone was then removed under reduced pressure, and petroleum ether was added to the resulting residue. The mixture was filtered through a short plug of silica gel to remove triphenylphosphine oxide. The filtrate was concentrated in vacuo and purified by column chromatography on silica gel to afford the corresponding iodide.

Condition B: [3]

In a flame-dried round-bottom flask, Et_3N (1.5 equiv.) was added to a stirred solution of alcohol (1.0 equiv.) in dry DCM (0.2 M), followed by the addition of MsCl (1.5 equiv.). The reaction mixture was stirred at room temperature for 1 h. The mixture was then diluted with dry toluene to a concentration of 0.1 M and cooled to 0 °C. MeMgI (2.0 equiv.) was added at 0 °C, and the reaction mixture was stirred for 10 min. The reaction was quenched by the addition of a few drops of methanol, and the filtered through a short plug of silica gel. The filtrate was concentrated in vacuo and purified by column chromatography on silica gel to afford iodide.

Condition C: [4]

In a flame-dried round-bottom flask, iodine (1.5 equiv.) was dissolved in dry DCM (0.2 M) and cooled to 0 °C. Then triphenylphosphine (1.5 equiv.) was added, and the resulting yellow suspension was stirred at 0 °C for 1 h. N-Methylimidazole (1.5 equiv.) was then added, and the mixture was stirred for an additional 10 min. The alcohol (1 equiv.), dissolved in DCM (0.05 M), was added, and the reaction mixture was stirred at 0 °C for additional 1 h. The reaction was quenched with saturated aqueous $NaHSO_3/Na_2S_2O_5$ solution and extracted with DCM (3×). The combined organic extracts were dried over Na_2SO_4, filtered, and the solvent was removed under reduced pressure. The residue was filtered through a short plug of silica gel to remove triphenylphosphine oxide. The filtrate was concentrated in vacuo and purified by column chromatography on silica gel to afford iodide.

(±)-(((2-(2-Iodoethyl)cycloprop-2-en-1-yl)methoxy)methyl)benzene (1a)

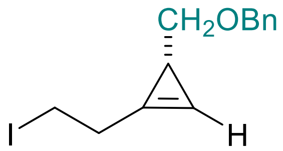

The title compound was prepared according to the condition A.

Scale, physical description, yield: 4.6 mmol, colorless oil, 56% yield.

R*f* = 0.5 (PE/Et_2O 95:5).

^{1}H NMR (400 MHz, Chloroform-*d*): δ 7.27 – 7.16 (m, 5H), 6.77 (s, 1H), 4.43 (s, 2H), 3.39 (dd, J = 9.7, 4.9 Hz, 1H), 3.31 – 3.16 (m, 3H), 3.03 (tt, J = 7.4, 2.0 Hz, 2H), 1.73 (td, J = 5.1, 1.5 Hz, 1H).

^{13}C NMR (101 MHz, Chloroform-*d*): δ 138.84, 128.48, 127.83, 127.60, 124.36, 105.14, 77.66, 72.77, 30.91, 18.25, 0.96.

HRMS (APCI): m/z calculated for $C_{13}H_{16}IO$ $[M+H]^+$: 315.0235, found 315.0240.

(±)-3-(Ethoxymethyl)-1-(2-iodoethyl)cycloprop-1-ene (1b)

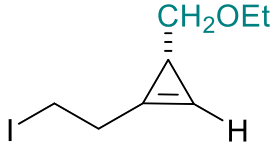

The title compound was prepared according to the condition A, starting from the tosylate **11a**.

Scale, physical description, yield: 3.4 mmol, colorless oil, 80% yield.

R*f* = 0.55 (PE/Et_2O 95:5).

^{1}H NMR (500 MHz, Chloroform-*d*): δ 6.85 (s, 1H), 3.47 (qd, J = 7.0, 1.1 Hz, 2H), 3.39 – 3.34 (m, 2H), 3.34 – 3.27 (m, 2H), 3.17 – 3.04 (m, 2H), 1.76 (td, J = 5.1, 1.5 Hz, 1H), 1.20 (t, J = 7.0 Hz, 3H).

^{13}C NMR (126 MHz, Chloroform-*d*): δ 124.44, 105.36, 78.00, 65.99, 30.96, 18.38, 15.47, 0.95.

HRMS (APCI): m/z calculated for $C_8H_{14}IO$ $[M+H]^+$: 253.0084, found 253.0101.

(±)-(1-(Ethoxymethyl)-2-(2-iodoethyl)cycloprop-2-en-1-yl)benzene (1c)

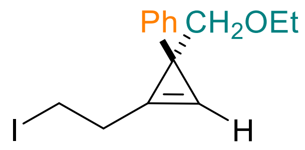

The title compound was prepared according to the condition A, starting from the tosylate **11b**.

Scale, physical description, yield: 1.9 mmol, colorless oil, 81% yield.

Rf = 0.45 (PE/Et_2O 95:5).

^{1}H NMR (500 MHz, Chloroform-*d*): δ 7.28 (m, 2H), 7.25 – 7.22 (m, 2H), 7.18 – 7.13 (m, 1H), 6.95 (s, 1H), 3.97 (d, J = 10.1 Hz, 1H), 3.81 (d, J = 10.1 Hz, 1H), 3.53 (qd, J = 7.0, 2.1 Hz, 2H), 3.39 – 3.29 (m, 2H), 3.12 (tdd, J = 6.6, 2.2, 1.2 Hz, 2H), 1.22 (t, J = 7.0 Hz, 3H).

^{13}C NMR (126 MHz, Chloroform-*d*): δ 146.27, 128.13, 126.30, 125.43, 123.66, 104.61, 77.81, 66.14, 29.75, 29.67, 15.45, 0.71.

HRMS (APCI): m/z calculated for $C_{14}H_{18}IO$ $[M+H]^+$: 329.0403, found 329.0397.

(±)-(((2-(1-(Iodomethyl)cyclobutyl)cycloprop-2-en-1-yl)methoxy)methyl)benzene (1d)

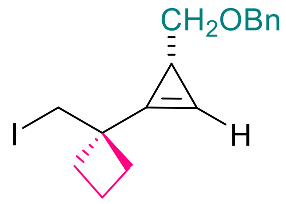

The title compound was prepared according to the condition A.

Scale, physical description, yield: 1.7 mmol, colorless oil, 22% yield.

Rf = 0.5 (PE/Et_2O 95:5).

^{1}H NMR (500 MHz, Chloroform-*d*): 7.30-7.25 (m, 3H), 7.25 – 7.15 (m, 2H), 6.71 (s, 1H), 4.51 – 4.39 (m, 2H), 3.45 (q, J = 9.5 Hz, 2H), 3.38 (m, 2H), 2.18 – 2.02 (m, 2H), 1.98 (m, 2H), 1.94 – 1.83 (m, 2H), 1.79 (m, 1H).

^{13}C NMR (126 MHz, Chloroform-*d*): 138.92, 129.78, 128.43, 127.86, 127.54, 103.12, 78.05, 72.88, 42.01, 32.24, 32.18, 19.11, 17.22, 14.32.

HRMS (APCI): m/z calculated for $C_{16}H_{20}IO$ $[M+H]^+$: 355.0559, found 355.0592.

(±)-((((R)-2-((R)-2-Iodopropyl)cycloprop-2-en-1-yl)methoxy)methyl)benzene (1e)

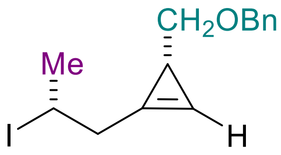

The title compound was prepared according to the condition B.

Scale, physical description, yield: 1.8 mmol, colorless oil, 48% yield.

Rf = 0.45 (PE/Et_2O 95:5).

^{1}H NMR (400 MHz, Chloroform-*d*): δ 7.27– 7.15 (m, 5H), 6.78 (s, 1H), 4.48 – 4.39 (m, 2H), 4.28 (h, *J* = 6.8 Hz, 1H), 3.36 (dd, *J* = 9.8, 5.0 Hz, 1H), 3.30 (dd, *J* = 9.8, 5.0 Hz, 1H), 3.09 (ddd, *J* = 16.9, 6.9, 1.1 Hz, 1H), 2.98 (ddd, *J* = 16.9, 6.5, 1.4 Hz, 1H), 1.87 (d, *J* = 6.8 Hz, 3H), 1.75 (td, *J* = 5.0, 1.5 Hz, 1H).

^{13}C NMR (101 MHz, Chloroform-*d*): δ 138.85, 128.46, 127.78, 127.58, 123.61, 105.35, 77.47, 72.71, 39.92, 28.56, 22.92, 18.17.

HRMS (APCI): m/z calculated for $C_{14}H_{18}IO$ $[M+H]^+$: 329.0397, found 329.0402.

(±)-((((R)-2-((S)-2-Iodopropyl)cycloprop-2-en-1-yl)methoxy)methyl)benzene (1f)

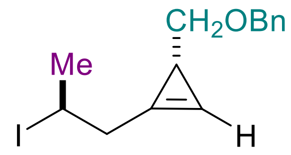

The title compound was prepared according to the condition B.

Scale, physical description, yield: 3 mmol, colorless oil, 43% yield.

R*f* = 0.45 (PE/Et_2O 95:5).

^{1}H NMR (400 MHz, Chloroform-*d*): δ 7.27– 7.15 (m, 5H), 6.78 (s, 1H), 4.43 (s, 2H), 4.36 – 4.23 (m, 1H), 3.39 (dd, *J* = 9.8, 4.9 Hz, 1H), 3.30 (dd, *J* = 9.8, 5.2 Hz, 1H), 3.07 (ddd, *J* = 16.8, 7.1, 1.1 Hz, 1H), 2.94 (ddd, *J* = 16.8, 6.3, 1.3 Hz, 1H), 1.87 (d, *J* = 6.9 Hz, 3H), 1.71 (td, *J* = 5.1, 1.5 Hz, 1H).

^{13}C NMR (101 MHz, Chloroform-*d*): δ 138.85, 128.47, 127.86, 127.59, 123.70, 105.34, 77.67, 72.78, 40.07, 28.74, 23.22, 18.30.

HRMS (APCI): m/z calculated for $C_{14}H_{18}IO$ $[M+H]^+$: 329.0397, found 329.0396.

(±)-((((R)-2-((2R,3R)-3-iodobutan-2-yl)cycloprop-2-en-1-yl)methoxy)methyl)benzene (1g)

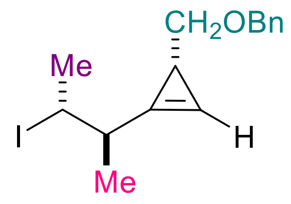

The title compound was prepared according to the condition C.

Scale, physical description, yield: 1.1 mmol, colorless oil, 39% yield.

R*f* = 0.4 (PE/Et_2O 95:5).

^{1}H NMR (500 MHz, Chloroform-*d*): δ 7.34 (m, 4H), 7.28 (m, 1H), 6.82 (d, J = 1.9 Hz, 1H), 4.52 (qd, J = 11.9, 1.6 Hz, 2H), 4.47 – 4.42 (m, 1H), 3.40 (qdd, J = 9.9, 5.1, 1.6 Hz, 2H), 2.62 (pd, J = 6.4, 3.1 Hz, 1H), 1.93 (d, J = 6.9 Hz, 3H), 1.87 (td, J = 5.1, 1.5 Hz, 1H), 1.25 (d, J = 6.8 Hz, 3H).

^{13}C NMR (126 MHz, Chloroform-*d*): δ 138.91, 128.46, 127.79, 127.57, 127.44, 104.65, 77.66, 72.77, 40.58, 33.21, 26.36, 18.70, 17.83.

HRMS (APCI): m/z calculated for $C_{15}H_{20}IO$ $[M+H]^+$: 343.0553, found 343.0528.

(±)-((((R)-2-((2S,3S)-3-Iodobutan-2-yl)cycloprop-2-en-1-yl)methoxy)methyl)benzene (1h)

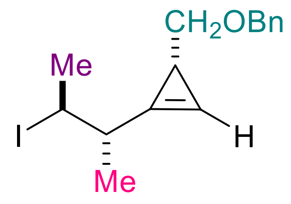

The title compound was prepared according to the condition C.

Scale, physical description, yield: 1 mmol, colorless oil, 34% yield.

R*f* = 0.4 (PE/Et_2O 95:5).

^{1}H NMR (500 MHz, Chloroform-*d*): δ 7.38 – 7.31 (m, 4H), 7.30 – 7.26 (m, 1H), 6.81 (s, 1H), 4.55 – 4.47 (m, 3H), 3.48 (dt, J = 7.8, 3.9 Hz, 1H), 3.41 (ddd, J = 9.9, 5.5, 2.4 Hz, 1H), 2.62 – 2.52 (m, 1H), 1.94 (d, J = 7.0 Hz, 3H), 1.80 (tt, J = 5.0, 1.5 Hz, 1H), 1.25 (d, J = 6.8 Hz, 3H).

^{13}C NMR (126 MHz, Chloroform-*d*): δ 138.90, 128.47, 127.94, 127.60, 127.55, 104.56, 78.04, 72.90, 40.88, 33.87, 26.63, 18.84, 17.73.

HRMS (APCI): m/z calculated for $C_{15}H_{20}IO$ $[M+H]^+$: 343.0553, found 343.0527.

(±)-2-(3-((benzyloxy)methyl)cycloprop-1-en-1-yl)ethyl diethyl phosphate (1i)

The title compound was prepared according to previously reported procedure.[5]

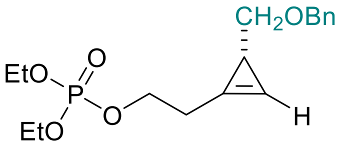

Scale, physical description, yield: 15 mmol, colorless oil, 51% yield.

Rf = 0.5 (PE/EtOAc 35:65).

^{1}H NMR (500 MHz, Chloroform-*d*): δ 7.33 (d, J = 4.4 Hz, 4H), 7.28 (m, 1H), 6.83 (d, J = 1.9 Hz, 1H), 4.50 (s, 2H), 4.24 (qd, J = 6.9, 2.2 Hz, 2H), 4.09 (p, J = 7.3 Hz, 4H), 3.37 (d, J = 5.0 Hz, 2H), 2.91 – 2.84 (m, 2H), 1.77 (td, J = 5.0, 1.5 Hz, 1H), 1.32 (td, J = 7.1, 1.1 Hz, 6H).

^{13}C NMR (126 MHz, Chloroform-*d*): δ 138.89, 128.46, 127.78, 127.58, 121.28, 105.65, 77.63, 72.72, δ 64.95 (d, *J* = 5.8 Hz), 63.94 (d, *J* = 5.9 Hz), 28.00 (d, *J* = 7.2 Hz), 17.77, 16.27 (d, *J* = 6.8 Hz).

HRMS (APCI): m/z calculated for $C_{17}H_{26}O_5P$ $[M+H]^+$: 341.1518, found 343.1419.

2.1 General procedure for synthesis of polysubstituted bicyclo pentanes.

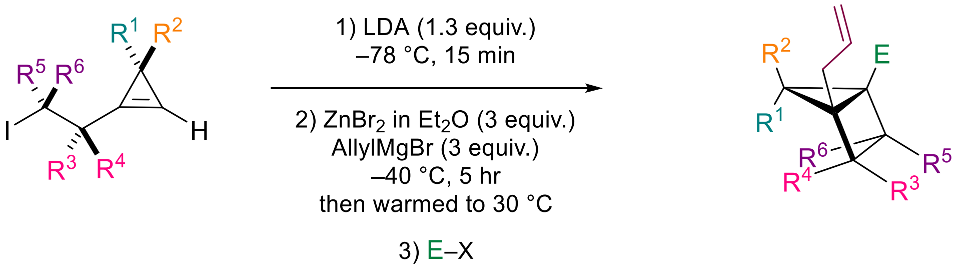

Step 1, Preparation of lithium diisopropylamide:

In a flame-dried Schlenk tube, a solution of diisopropylamine (1.5 equiv.) in dry THF (0.4 M, based on cyclopropene) was cooled to 0 °C, and n-BuLi (1.3 equiv.) was added dropwise. The resulting mixture was stirred at 0 °C for 30 min.

Step 2, Synthesis of Housane:[6]

Cyclopropene (1.0 equiv.) in dry Et_2O (0.05 M) was cooled to −78 °C (dry ice/acetone bath), and freshly prepared LDA (see above) was added dropwise. After stirring for 15 min, $ZnBr_2$ (2.0 equiv., 1.0 M in Et_2O) and allylmagnesium bromide (2.0 equiv.) were added sequentially. The reaction mixture was allowed to warm gradually to −40 °C and maintained at this temperature for 5 h. The mixture was then heated to 30 °C and stirred overnight.

For compounds **1f–1h**, transmetallation to copper was required for efficient housane formation. Accordingly, CuCN·2LiCl was added to the reaction mixture after allylzincation step was complete, as indicated by TLC.
In a flame-dried Schlenk tube, lithium chloride (6.0 equiv.) was flame-dried under vacuum. Dry THF (0.10 M, based on cyclopropene) was added, and the suspension was cooled to 0 °C. CuCN (3.0 equiv.) was then added, and the resulting mixture was stirred at 0 °C for 30 min to generate the CuCN·2LiCl complex. This solution was added to the reaction mixture, which was then heated to 30 °C and stirred for overnight.

Note: The use of freshly prepared allylmagnesium bromide is recommended, as commercially available solutions may lead to reduced yields. Allylmagnesium bromide should be stored for no longer than one month.

Step 3, Electrophile trapping:

Condition D, proton quench:

The reaction was quenched by the addition of 1 M HCl, and the mixture was transferred to a separatory funnel and extracted with Et_2O (3 ×). The combined organic extracts were dried over Na_2SO_4, filtered, and concentrated in vacuo. The crude product was purified by column chromatography on silica gel (typically eluting with 0.5–1.5% Et_2O in pentane) to afford the corresponding housane.

Condition E, allyl bromide or methyl iodide trapping:

In a flame-dried Schlenk tube, lithium chloride (6.0 equiv.) was flame-dried under vacuum. Dry THF (0.10 M, based on cyclopropene) was added, and the suspension was cooled to 0 °C. CuCN (3.0 equiv.) was then added, and the resulting mixture was stirred at 0 °C for 30 min to generate the CuCN·2LiCl complex. Separately, the housane reaction mixture (see above) was cooled to 0 °C, and the freshly prepared CuCN·2LiCl solution was added. Allyl bromide or methyl iodide (4.0 equiv.) was then added, and the reaction mixture was allowed to warm to 30 °C and stirred for 5–10 h, with reaction progress monitored by TLC. The reaction was quenched by the sequential addition of aqueous NH_4Cl and NH_4OH. The mixture was transferred to a separatory funnel and extracted with Et_2O (3 ×). The combined organic extracts were dried over Na_2SO_4, filtered, and concentrated in vacuo. The crude product was purified by column chromatography on silica gel (typically eluting with 0.5–1.5% Et_2O in pentane) to afford the corresponding housane.

Condition F, iodine quench:

In a flame-dried Schlenk tube, iodine (4.0 equiv.) was dissolved in dry THF (0.10 M, based on cyclopropene). This solution was added to the reaction mixture, and the resulting mixture was stirred for 5 h, with reaction progress monitored by TLC. The reaction was quenched by the addition of 1 M HCl. The mixture was transferred to a separatory funnel and extracted with Et_2O (3 ×). The combined organic extracts were dried over Na_2SO_4, filtered, and concentrated in vacuo. The crude product was purified by column chromatography on silica gel (typically eluting with 0.5–1.5% Et_2O in pentane) to afford the corresponding housane.

Condition G, cross coupling:

The reaction mixture was diluted with THF to a concentration of 0.10 M (based on cyclopropene), followed by the addition of $Pd(PPh_3)_4$ (0.15 equiv.) and the corresponding aryl iodide (5.0 equiv.). The resulting mixture was stirred for 5–10 h, with reaction progress monitored by TLC. The reaction was quenched by the addition of 1 M HCl. The mixture was transferred to a separatory funnel and extracted with Et_2O (3 ×). The combined organic extracts were dried over Na_2SO_4, filtered, and concentrated in vacuo. The crude product was purified by column chromatography on silica gel (typically eluting with 0.5–1.5% Et_2O in pentane) to afford the corresponding housane.

Condition H, tosyl cyanide trapping:

To the reaction mixture already containing CuCN·2LiCl for the cyclization step (see above), tosyl cyanide (5.0 equiv.) was added. The resulting mixture was stirred for 10 h, with reaction progress monitored by TLC. The reaction was quenched by the addition of 1 M HCl. The mixture was transferred to a separatory funnel and extracted with Et_2O (3 ×). The combined organic extracts were dried over Na_2SO_4, filtered, and concentrated in vacuo. The crude product was purified by column chromatography on silica gel (typically eluting with 0.5–1.5% Et_2O in pentane) to afford the corresponding housane.

(±)-(1R,4S,5R)-1-Allyl-5-((benzyloxy)methyl)bicyclo[2.1.0]pentane (6a)

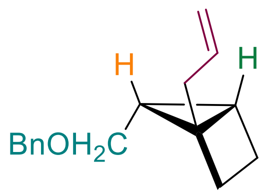

The title compound was prepared according to the condition D.

Scale, physical description, yield: 0.32 mmol, colorless oil, (38 mg, 0.17 mmol, 52% yield).

R*f* = 0.6 (PE/Et_2O 96:4).

^{1}H NMR (500 MHz, Chloroform-d): 7.32 (dd, J = 14.1, 6.9 Hz, 4H), 7.25 – 7.21 (m, 1H), 5.72 (ddt, J = 16.9, 10.1, 6.7 Hz, 1H), 5.06 – 4.90 (m, 2H), 4.57 (s, 2H), 3.76 – 3.61 (m, 2H), 2.31 (dd, J = 15.0, 6.8 Hz, 1H), 2.15 (dd, J = 15.0, 6.6 Hz, 1H), 2.01 (dddd, J = 10.9, 8.3, 4.4, 4.2 Hz, 1H), 1.86 (ddd, J = 11.1, 3.9 Hz, 1H), 1.62 (dd, J = 6.5, 5.4 Hz, 1H), 1.49 – 1.41 (m, 1H), 1.30 (dd, J = 11.1, 5.2 Hz, 1H), 1.03 (td, J = 7.4, 6.7 Hz, 1H).

^{13}C NMR (126 MHz, Chloroform-*d*): δ 138.97, 136.19, 128.46, 127.82, 127.59, 115.70, 72.73, 64.38, 38.73, 28.98, 26.25, 22.24, 20.85, 17.23.

HRMS (APCI): m/z calculated for $C_{16}H_{21}O$ $[M+H]^+$: 229.1587, found 229.1583.

(±)-(1R,4S,5R)-1-Allyl-5-(ethoxymethyl)bicyclo[2.1.0]pentane (6b)

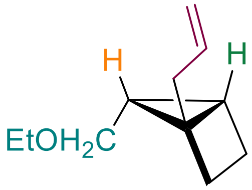

The title compound was prepared according to the condition D.

Scale, physical description, yield: 0.2 mmol, colorless oil, (19 mg, 0.11 mmol, 57% yield). Yield is approximate due to minor impurities.

Rf = 0.6 (PE/Et_2O 96:4).

^{1}H NMR (500 MHz, Chloroform-*d*): δ 5.74 (ddt, J = 16.9, 10.1, 6.7 Hz, 1H), 5.07 – 4.94 (m, 2H), 3.66 (qd, J = 10.6, 7.2 Hz, 2H), 3.56 (q, J = 7.0 Hz, 2H), 2.36 – 2.28 (m, 1H), 2.17 (ddt, J = 14.7, 6.5, 1.4 Hz, 1H), 2.09 – 2.01 (m, 1H), 1.89 (td, J = 11.1, 3.9 Hz, 1H), 1.64 (ddd, J = 6.1, 4.7, 1.7 Hz, 1H), 1.51 (dddd, J = 11.3, 5.6, 3.8, 1.7 Hz, 1H), 1.36 (ddd, J = 11.3, 5.7, 3.9 Hz, 1H), 1.24 (t, J = 7.0 Hz, 3H), 1.04 – 0.97 (m, 1H).

^{13}C NMR (126 MHz, Chloroform-*d*): δ 136.22, 115.65, 65.99, 64.53, 38.74, 28.81, 26.23, 22.24, 20.78, 17.22, 15.53.

HRMS (APCI): m/z calculated for $C_{10}H_{15}O$ $[M-CH_3]^+$: 151.1117, found 151.1114.

(±)-(1S,4R,5R)-1-Allyl-5-(ethoxymethyl)-4-iodobicyclo[2.1.0]pentane (6c)

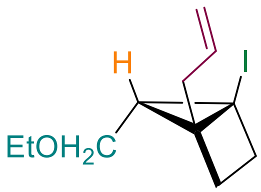

The title compound was prepared according to the condition F.

Scale, physical description, yield: 0.2 mmol, colorless oil, (37 mg, 0.23 mmol, 63% yield.

Rf = 0.5 (PE/Et_2O 96:4).

^{1}H NMR (500 MHz, Chloroform-*d*): δ 5.87 (ddt, J = 16.9, 10.1, 6.6 Hz, 1H), 5.22 – 5.06 (m, 2H), 3.70 (dd, J = 11.2, 6.9 Hz, 1H), 3.65 – 3.59 (m, 1H), 3.59 – 3.50 (m, 2H), 2.48 (td, J = 11.7, 11.1, 3.6 Hz, 1H), 2.33 (ddd, J = 15.3, 6.4, 1.6 Hz, 1H), 2.21 – 2.07 (m, 3H), 1.77 (ddd, J = 12.1, 6.0, 3.6 Hz, 1H), 1.39 (t, J = 7.0 Hz, 1H), 1.23 (m, 4H).

^{13}C NMR (126 MHz, Chloroform-*d*): δ 134.20, 116.88, 66.10, 63.49, 38.77, 37.43, 35.41, 30.59, 23.18, 15.49, 8.20.

HRMS (APCI): m/z calculated for $C_{11}H_{18}IO$ $[M+H]^+$: 293.0397, found 293.0393.

(±)-(1R,4S,5s)-1,4-Diallyl-5-((benzyloxy)methyl)bicyclo[2.1.0]pentane (6d)

The title compound was prepared according to the condition D.

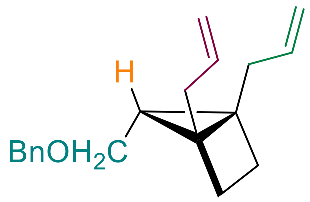

The title compound was prepared according to the condition E.

Scale, physical description, yield: 0.32 mmol, colorless oil, (49 mg, 0.18 mmol, 56% yield).

R*f* = 0.55 (PE/Et_2O 96:4).

^{1}H NMR (500 MHz, Chloroform-d): δ 7.40 – 7.32 (m, 4H), 7.30 – 7.26 (m, 1H), 5.83 (ddtd, J = 16.9, 10.2, 6.7, 1.0 Hz, 2H), 5.09 – 4.97 (m, 4H), 4.59 (s, 2H), 3.72 (dd, J = 7.2, 1.0 Hz, 2H), 2.38 – 2.30 (m, 2H), 2.19 (ddt, J = 15.1, 6.7, 1.4 Hz, 2H), 1.92 – 1.83 (m, 2H), 1.49 – 1.39 (m, 2H), 0.94 (t, J = 7.2 Hz, 1H).

^{13}C NMR (126 MHz, Chloroform-*d*): δ 139.11, 136.50, 128.43, 127.66, 127.52, 115.58, 72.52, 64.70, 36.74, 31.93, 31.90, 21.53.

HRMS (APCI): m/z calculated for $C_{19}H_{25}O$ $[M+H]^+$: 269.1900, found 269.1904.

(±)-(1S,4S,5R)-1-Allyl-5-((Benzyloxy)methyl)-4-methylbicyclo[2.1.0]pentane (6e)

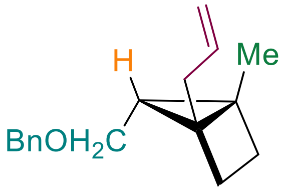

The title compound was prepared according to the condition E.

Scale, physical description, yield: 0.26 mmol, colorless oil, (12 mg, 0.05 mmol, 19% yield).

R*f* = 0.6 (PE/Et_2O 96:4).

^{1}H NMR (500 MHz, Chloroform-d): δ 7.40 – 7.32 (m, 4H), 7.30 – 7.26 (m, 1H), 5.82 (ddt, J = 16.9, 10.2, 6.6 Hz, 1H), 5.07 – 4.96 (m, 2H), 4.58 (d, J = 2.7 Hz, 2H), 3.75 – 3.64 (m, 2H), 2.29 – 2.22 (m, 1H), 2.18 – 2.10 (m, 1H), 1.88 (td, J = 10.9, 3.6 Hz, 1H), 1.79 (td, J = 10.9, 3.7 Hz, 1H), 1.50 (ddd, J = 11.2, 5.7, 3.7 Hz, 1H), 1.45 – 1.38 (m, 1H), 1.23 (s, 3H), 0.79 (t, J = 7.2 Hz, 1H).

^{13}C NMR (126 MHz, Chloroform-*d*): δ 139.12, 136.66, 128.46, 127.74, 127.55, 115.44, 72.45, 64.78, 36.56, 32.50, 31.45, 28.40, 23.82, 21.64, 17.53.

HRMS (APCI): m/z calculated for $C_{17}H_{23}O$ $[M+H]^+$: 243.1743, found 243.1747.

(±)-(1R,4S,5R)-5-((Benzyloxy)methyl)-1-((R/S)-but-3-en-2-yl)bicyclo[2.1.0]pentane (6f)

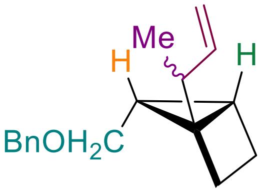

The title compound was prepared according to the condition D.

Scale, physical description, yield: 0.32 mmol, colorless oil, (34 mg, 0.14 mmol, 44% yield), as an inseparable mixture of diastereomers (*d.r* = 65:35).

R*f* = 0.55 (PE/Et_2O 96:4).

^{1}H NMR (500 MHz, Chloroform-d): δ 7.36 (dd, J = 14.0, 6.9 Hz, 4H), 7.32 – 7.23 (m, 1H), 5.88 – 5.59 (m, 1H), 5.04 – 4.88 (m, 2H), 4.61 (t, J = 4.4 Hz, 2H), 3.77 – 3.62 (m, 2H), 2.20 (h, J = 6.6 Hz, 1H), 2.07 – 1.97 (m, 1H), 1.90 (dtd, J = 22.4, 11.0, 3.8 Hz, 1H), 1.68 (dt, J = 16.9, 5.3 Hz, 1H), 1.41 (ddt, J = 11.4, 5.6, 2.9 Hz, 1H), 1.33 (dt, J = 10.6, 4.9 Hz, 1H), 1.12 (dt, J = 13.8, 7.2 Hz, 1H), 0.98 (ddd, J = 69.3, 6.8, 1.2 Hz, 3H).

^{13}C NMR (126 MHz, Chloroform-*d*): δ 142.19, 141.10, 139.01, 128.45, 127.76, 127.58, 127.56, 113.52, 113.14, 72.80, 72.75, 64.41, 64.35, 40.51, 40.20, 33.95, 24.52, 24.48, 20.41, 20.39, 20.30, 20.25, 17.13, 17.00, 16.74, 15.99.

HRMS (APCI): m/z calculated for $C_{17}H_{23}O$ $[M+H]^+$: 243.1743, found 243.1749.

(±)-(1S,4R,5S)-1-Allyl-5-(ethoxymethyl)-4-phenylbicyclo[2.1.0]pentane (6g)

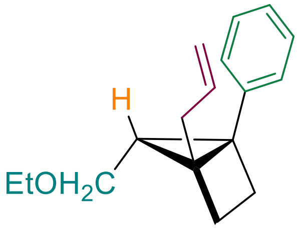

The title compound was prepared according to the condition G.

Scale, physical description, yield: 0.2 mmol, colorless oil, (34 mg, 0.14 mmol, 70% yield). Yield is approximate due to minor impurities.

R*f* = 0.5 (PE/Et_2O 96:4).

^{1}H NMR (500 MHz, Chloroform-*d*): δ 7.29 (t, J = 7.7 Hz, 2H), 7.23 – 7.18 (m, 2H), 7.15 (td, J = 7.1, 1.4 Hz, 1H), 5.74 (ddt, J = 16.9, 10.1, 6.6 Hz, 1H), 5.07 – 4.93 (m, 2H), 3.80 (d, J = 7.0 Hz, 2H), 3.59 (qt, J = 7.0, 3.3 Hz, 2H), 2.48 (td, J = 11.2, 3.8 Hz, 1H), 2.35 (ddd, J = 15.2, 6.6, 1.5 Hz, 1H), 2.23 (ddt, J = 15.3, 6.8, 1.5 Hz, 1H), 2.03 (td, J = 11.1, 3.9 Hz, 1H), 1.83 (ddd, J = 11.5, 5.9, 4.0 Hz, 1H), 1.69 – 1.62 (m, 2H), 1.24 (m, 4H).

^{13}C NMR (126 MHz, Chloroform-*d*): δ 141.56, 135.84, 128.25, 126.96, 125.46, 115.91, 65.92, 64.39, 37.78, 36.50, 35.55, 33.55, 21.73, 21.31, 15.54.

HRMS (APCI): m/z calculated for $C_{17}H_{21}O$ $[M-H]^+$: 241.1587, found 241.1591.

(±)-(1S,4R,5S)-1-Allyl-5-((benzyloxy)methyl)-4-phenylbicyclo[2.1.0]pentane (6h)

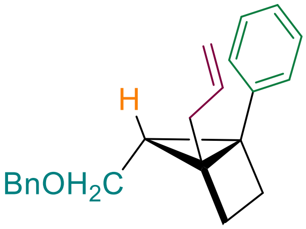

The title compound was prepared according to the condition G.

Scale, physical description, yield: 0.32 mmol, colorless oil, (38 mg, 0.125 mmol, 39% yield). Yield is approximate due to minor impurities.

R*f* = 0.5 (PE/Et_2O 96:4).

^{1}H NMR (500 MHz, Chloroform-*d*): δ 7.42 – 7.33 (m, 4H), 7.30 (d, J = 7.5 Hz, 3H), 7.22 (d, J = 7.6 Hz, 2H), 7.17 (t, J = 7.4 Hz, 1H), 5.77 (ddt, J = 16.9, 10.2, 6.7 Hz, 1H), 5.02 (dd, J = 23.6, 13.6 Hz, 2H), 4.63 (s, 2H), 3.86 (d, J = 7.1 Hz, 2H), 2.47 (td, J = 11.2, 3.8 Hz, 1H), 2.38 (dd, J = 15.2, 6.6 Hz, 1H), 2.25 (dd, J = 15.2, 6.8 Hz, 1H), 2.02 (td, J = 11.2, 4.0 Hz, 1H), 1.80 (dt, J = 10.9, 4.9 Hz, 1H), 1.72 (t, J = 7.1 Hz, 1H), 1.66 – 1.57 (m, 1H).

^{13}C NMR (126 MHz, Chloroform-*d*): 141.51, 138.93, 135.84, 128.47, 128.28, 127.72, 127.60, 126.98, 125.52, 115.98, 72.62, 64.27, 37.87, 36.54, 35.67, 33.60, 21.78, 21.37.

HRMS (APCI): m/z calculated for $C_{22}H_{25}O$ $[M+H]^+$: 305.1900, found 305.1881.

(±)-4-((1R,4S,5S)-4-Allyl-5-((benzyloxy)methyl)bicyclo[2.1.0]pentan-1-yl)benzonitrile (6i)

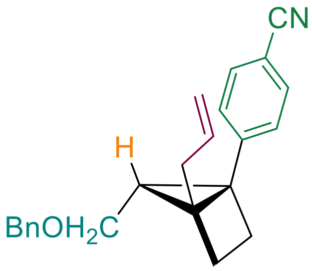

The title compound was prepared according to the condition G.

Scale, physical description, yield: 0.32 mmol, colorless oil, (32 mg, 0.097 mmol, 30% yield). Yield is approximate due to minor impurities.

R*f* = 0.5 (PE/Et_2O 9:1).

^{1}H NMR (500 MHz, Chloroform-*d*): δ 7.57 – 7.52 (m, 2H), 7.34 (d, J = 4.4 Hz, 4H), 7.29 (q, J = 4.1 Hz, 1H), 7.24 – 7.21 (m, 2H), 5.68 (ddt, J = 17.0, 10.3, 6.7 Hz, 1H), 5.05 – 4.93 (m, 2H), 4.59 (s, 2H), 3.88 – 3.77 (m, 2H), 2.45 (td, J = 11.1, 3.7 Hz, 1H), 2.39 – 2.23 (m, 2H), 2.03 (td, J = 11.3, 4.0 Hz, 1H), 1.80 (m, 2H), 1.63 (dd, J = 11.4, 5.2 Hz, 1H).

^{13}C NMR (126 MHz, Chloroform-*d*): δ 147.76, 138.61, 134.93, 132.06, 128.53, 127.75, 127.72, 127.01, 119.40, 116.65, 108.66, 72.86, 63.96, 40.71, 36.22, 35.70, 35.42, 21.15, 20.94.

HRMS (APCI): m/z calculated for $C_{23}H_{24}NO$ $[M+H]^+$: 330.1852, found 330.1832.

(±)-(1R,4R,5R)-1-Allyl-5-(ethoxymethyl)-5-phenylbicyclo[2.1.0]pentane (6j)

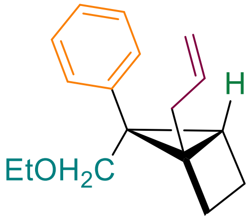

The title compound was prepared according to the condition D.

Scale, physical description, yield: 0.6 mmol, colorless oil, (80 mg, 0.33 mmol, 55% yield).

R*f* = 0.5 (PE/Et_2O 96:4).

^{1}H NMR (500 MHz, Chloroform-*d*): δ 7.31 – 7.27 (m, 2H), 7.26 – 7.21 (m, 2H), 7.18 – 7.13 (m, 1H), 5.71 (ddt, J = 16.9, 10.3, 6.3 Hz, 1H), 5.02 – 4.92 (m, 2H), 3.94 (d, J = 10.6 Hz, 1H), 3.88 (d, J = 10.6 Hz, 1H), 3.50 – 3.36 (m, 2H), 2.22 – 2.12 (m, 2H), 2.08 (td, J = 11.2, 3.8 Hz, 1H), 1.99 – 1.91 (m, 1H), 1.83 (ddt, J = 15.7, 6.7, 1.5 Hz, 1H), 1.74 (dddd, J = 11.5, 5.7, 3.9, 1.6 Hz, 1H), 1.59 (ddd, J = 11.1, 5.7, 3.7 Hz, 1H), 1.09 (t, J = 7.0 Hz, 3H).

^{13}C NMR (126 MHz, Chloroform-*d*): δ 141.82, 136.46, 130.27, 128.02, 126.28, 115.56, 69.02, 66.20, 37.65, 35.11, 34.19, 26.34, 23.64, 17.97, 15.31.

HRMS (APCI): m/z calculated for $C_{17}H_{23}O$ $[M+H]^+$: 243.1743, found 243.1742.

(±)-(1S,4S,5R)-1-Allyl-5-((benzyloxy)methyl)spiro[bicyclo[2.1.0]pentane-2,1'-cyclobutane] (6k)

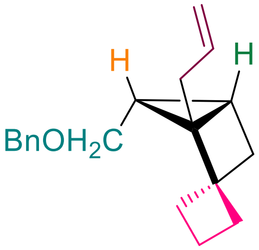

The title compound was prepared according to the condition D.

Scale, physical description, yield: 0.38 mmol, colorless oil (49 mg, 0.18 mmol, 50% yield).

R*f* = 0.5 (PE/Et_2O 96:4).

^{1}H NMR (500 MHz, Chloroform-*d*): δ 7.40 – 7.32 (m, 4H), 7.31 – 7.26 (m, 1H), 5.80 (dddd, J = 17.0, 10.1, 7.7, 5.7 Hz, 1H), 5.11 (dq, J = 17.0, 1.7 Hz,

1H), 5.04 – 4.96 (m, 1H), 4.57 (s, 2H), 3.67 (dd, J = 10.3, 6.1 Hz, 1H), 3.59 (dd, J = 10.3, 8.5 Hz, 1H), 2.71 (ddt, J = 14.9, 7.8, 1.1 Hz, 1H), 2.44 (dddd, J = 10.7, 8.6, 6.1, 1.7 Hz, 1H), 1.99 – 1.89 (m, 2H), 1.87 – 1.67 (m, 4H), 1.66 – 1.60 (m, 3H), 1.19 (dt, J = 8.5, 5.9 Hz, 1H).

^{13}C NMR (126 MHz, Chloroform-*d*): δ 138.90, 136.95, 128.44, 127.88, 127.59, 115.62, 72.78, 65.73, 43.82, 34.82, 34.32, 33.95, 33.16, 31.97, 28.78, 18.70, 16.72.

HRMS (APCI): m/z calculated for $C_{19}H_{25}O$ $[M+H]^+$: 269.1905, found 269.1905.

(±)-(1S,4S,5R)-1,4-Diallyl-5-((benzyloxy)methyl)spiro[bicyclo[2.1.0]pentane-2,1'-cyclobutane] (6l)

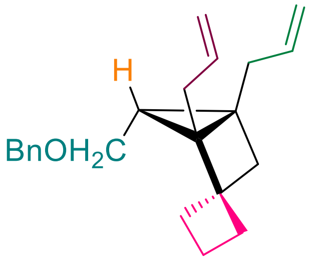

The title compound was prepared according to the condition E.

Scale, physical description, yield: 0.19 mmol, colorless oil (17 mg, 0.05 mmol, 29% yield).

R*f* = 0.45 (PE/Et_2O 96:4).

^{1}H NMR (500 MHz, Chloroform-*d*): δ 7.39 – 7.31 (m, 4H), 7.28 (dt, J = 6.6, 2.1 Hz, 1H), 5.91 (dddd, J = 17.4, 10.2, 7.5, 5.7 Hz, 1H), 5.77 (dddd, J = 15.2, 10.2, 7.5, 5.8 Hz, 1H), 5.15 (dq, J = 17.1, 1.8 Hz, 1H), 5.06 – 4.94 (m, 3H), 4.54 (bs, 2H), 3.71 – 3.54 (m, 2H), 2.70 (ddd, J = 15.3, 7.8, 1.5 Hz, 1H), 2.48 – 2.36 (m, 1H), 2.22 (dq, J = 6.9, 1.5 Hz, 2H), 1.90 – 1.68 (m, 7H), 1.65 (dtd, J = 11.1, 5.6, 2.8 Hz, 1H), 1.05 (dd, J = 8.2, 6.2 Hz, 1H).

^{13}C NMR (126 MHz, Chloroform-*d*): δ 139.07, 137.51, 136.70, 128.41, 127.69, 127.50, 115.58, 115.37, 72.54, 66.29, 43.39, 37.72, 37.07, 36.25, 34.87, 33.07, 32.99, 31.82, 30.46, 16.70.

HRMS (APCI): m/z calculated for $C_{22}H_{27}O$ $[M-H]^+$: 307.2062, found 307.2079.

(±)-(1S,3S,4S,5R)-1-Allyl-5-((benzyloxy)methyl)-3-methylbicyclo[2.1.0]pentane (6m)

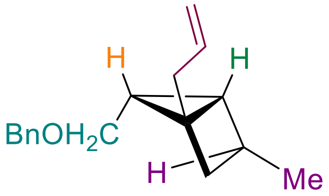

The title compound was prepared according to the condition D.

Scale, physical description, yield: 0.31 mmol, colorless oil, (43mg, 0.18 mmol, 58% yield).

R*f* = 0.55 (PE/Et_2O 96:4).

^{1}H NMR (500 MHz, Chloroform-*d*): δ 7.41 – 7.33 (m, 4H), 7.29 (t, J = 7.1 Hz, 1H), 5.78 (ddt, J = 17.0, 10.4, 6.9 Hz, 1H), 5.02 (t, J = 13.8 Hz, 2H), 4.60 (m, 2H), 3.77 – 3.63 (m, 2H), 2.29 (dd, J = 14.8, 6.9 Hz, 1H), 2.23 (dd, J = 14.9, 6.8 Hz, 1H), 1.69 (td, J = 16.5, 7.3 Hz, 2H), 1.48 (dd, J = 5.9, 0.9 Hz, 1H), 1.30 – 1.24 (m, 1H), 1.17 (m, 4H).

^{13}C NMR (126 MHz, Chloroform-*d*): δ 138.94, 136.40, 128.45, 127.81, 127.59, 115.76, 72.72, 65.07, 39.31, 31.07, 28.49, 28.09, 27.08, 25.00, 21.81.

HRMS (APCI): m/z calculated for $C_{17}H_{23}O$ $[M+H]^+$: 243.1743, found 243.1741.

(±)-(1S,3R,4S,5R)-1-Allyl-5-((benzyloxy)methyl)-3-methylbicyclo[2.1.0]pentane (6n)

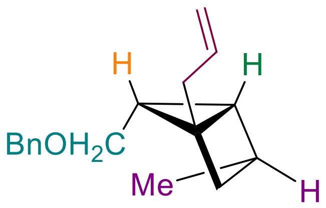

The title compound was prepared according to the condition D.

Scale, physical description, yield: 0.24 mmol, colorless oil (36 mg, 0.14 mmol, 61% yield). Yield is approximate due to minor impurities.

R*f* = 0.55 (PE/Et2O 96:4).

^{1}H NMR (500 MHz, Chloroform-*d*): δ 7.42 – 7.32 (m, 5H), 7.32 – 7.26 (m, 1H), 5.78 (ddt, J = 17.0, 10.3, 6.6 Hz, 1H), 5.11 – 4.91 (m, 3H), 4.60 (d, J = 1.6 Hz, 2H), 3.89 – 3.84 (m, 1H), 3.80 (dd, J = 10.3, 6.7 Hz, 1H), 2.58 (ddt, J = 10.2, 7.4, 4.7 Hz, 1H), 2.33 (ddd, J = 14.8, 6.8, 1.4 Hz, 1H), 2.23 (dd, J = 14.9, 6.9 Hz, 1H), 2.02 (dd, J = 11.0, 11.0 Hz, 1H), 1.61 – 1.56 (dd, J = 6.3, 5.4 Hz, 1H), 1.34 (dd, J = 11.8, 4.7 Hz, 1H), 1.05 (dt, J = 8.4, 6.4 Hz, 1H), 0.83 (d, J = 7.2 Hz, 3H).

^{13}C NMR (126 MHz, Chloroform-*d*): δ 138.84, 136.38, 128.44, 127.91, 127.60, 115.64, 72.74, 66.86, 38.33, 27.98, 27.86, 26.04, 25.47, 24.76, 17.58.

HRMS (APCI): m/z calculated for $C_{17}H_{23}O$ $[M+H]^+$: 243.1743, found 243.1749.

(±)-(1R,4R,5S)-4-Allyl-5-((benzyloxy)methyl)-2-methylbicyclo[2.1.0]pentane-1-carbonitrile (6o)

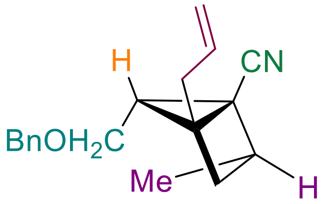

The title compound was prepared according to the condition H.

Scale, physical description, yield: 0.3 mmol, colorless oil (17 mg, 0.06 mmol, 21% yield). Yield is approximate due to minor impurities.

R*f* = 0.45 (PE/Et2O 9:1).

^{1}H NMR (500 MHz, Chloroform-*d*): δ 7.39 – 7.27 (m, 5H), 5.82 (ddt, J = 17.1, 10.2, 6.8 Hz, 1H), 5.21 – 5.06 (m, 2H), 4.58 (s, 2H), 3.88 – 3.71 (m, 2H), 2.84 – 2.74 (m, 1H), 2.52 – 2.37 (m, 2H), 2.15 (dd, J = 12.3, 10.6 Hz, 1H), 1.74 (dd, J = 8.0, 6.3 Hz, 1H), 1.37 (dd, J = 12.2, 5.0 Hz, 1H), 0.93 (d, J = 7.2 Hz, 3H).

^{13}C NMR (126 MHz, Chloroform-*d*): δ 138.04, 133.31, 128.61, 127.96, 127.84, 120.83, 118.13, 73.04, 64.69, 37.26, 36.77, 35.83, 28.79, 26.56, 18.20, 16.63.

HRMS (APCI): m/z calculated for $C_{18}H_{22}NO$ $[M+H]^+$: 268.1696, found 268.1720.

(±)-(1S,2S,3S,4S,5R)-1-Allyl-5-((benzyloxy)methyl)-2,3-dimethylbicyclo[2.1.0]pentane (6p)

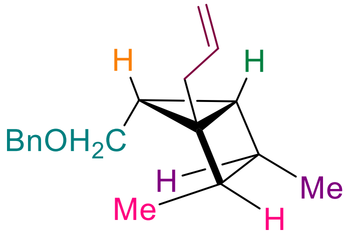

The title compound was prepared according to the condition D.

Scale, physical description, yield: 0.34 mmol, colorless oil (48 mg, 0.19 mmol, 55% yield). Yield is approximate due to minor impurities.

R*f* = 0.4 (PE/Et2O 96:4).

^{1}H NMR (500 MHz, Chloroform-*d*): δ 7.36 (qt, J = 7.9, 2.8 Hz, 5H), 7.31 – 7.26 (m, 1H), 5.77 (ddt, J = 17.0, 10.1, 6.9 Hz, 1H), 5.06 – 5.00 (m, 1H), 4.97 (ddt, J = 10.1,

2.2, 1.2 Hz, 1H), 4.60 (q, J = 12.0 Hz, 2H), 3.81 – 3.77 (m, 2H), 2.31 – 2.25 (m, 1H), 2.21 (ddt, J = 14.7, 6.9, 1.4 Hz, 1H), 1.75 – 1.68 (m, 1H), 1.47 (qd, J = 6.8, 4.3 Hz, 1H), 1.44 – 1.40 (m, 1H), 1.17 – 1.09 (m, 4H), 0.81 (d, J = 7.2 Hz, 3H).

^{13}C NMR (126 MHz, Chloroform-*d*): δ 138.88, 136.88, 128.46, 127.88, 127.61, 115.67, 72.80, 66.65, 41.31, 39.73, 31.82, 30.00, 29.94, 25.56, 20.83, 15.34.

HRMS (APCI): m/z calculated for $C_{18}H_{24}O$ $[M+H]^+$: 257.1900, found 257.1921.

(±)-(1S,2R,3R,4S,5R)-1-Allyl-5-((benzyloxy)methyl)-2,3-dimethylbicyclo[2.1.0]pentane (6q)

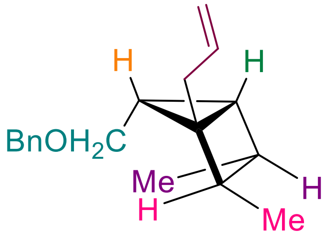

The title compound was prepared according to the condition D.

Scale, physical description, yield: 0.33 mmol, colorless oil (35 mg, 0.14 mmol, 42% yield).

R*f* = 0.4 (PE/Et$_2$O 96:4).

^{1}H NMR (500 MHz, Chloroform-*d*): δ 7.40 – 7.32 (m, 4H), 7.31 – 7.25 (m, 1H), 5.75 (dddd, J = 17.4, 10.2, 7.3, 5.6 Hz, 1H), 5.04 (dq, J = 17.2, 1.8 Hz, 1H), 5.00 – 4.91 (m, 1H), 4.58 (s, 2H), 3.87 (dd, J = 10.2, 7.8 Hz, 1H), 3.78 (dd, J = 10.3, 7.0 Hz, 1H), 2.68 (ddt, J = 15.2, 7.3, 1.3 Hz, 1H), 1.92 – 1.82 (m, 2H), 1.68 (qd, J = 6.9, 4.3 Hz, 1H), 1.60 (dd, J = 5.8, 4.7, 1H), 1.14 (d, J = 6.9 Hz, 3H), 1.11 (m, 1H), 0.83 (d, J = 7.2 Hz, 3H).

^{13}C NMR (126 MHz, Chloroform-*d*): δ 138.88, 137.14, 128.44, 127.93, 127.60, 115.33, 72.84, 66.97, 37.04, 34.54, 33.90, 29.29, 28.94, 24.02, 18.05, 16.31.

HRMS (APCI): m/z calculated for $C_{18}H_{24}O$ $[M-H]^+$: 257.1900, found 257.1883.

(±)-(1R,2S,3S,4S,5S)-1,4-Diallyl-5-((benzyloxy)methyl)-2,3-dimethylbicyclo[2.1.0]pentane (6r)

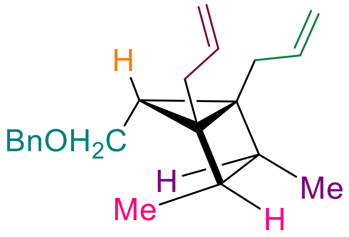

The title compound was prepared according to the condition E.

Scale, physical description, yield: 0.38 mmol, colorless oil (74 mg, 0.25 mmol, 65% yield).

R*f* = 0.4 (PE/Et$_2$O 96:4).

^{1}H NMR (500 MHz, Chloroform-*d*): δ 7.41 – 7.32 (m, 4H), 7.31 – 7.26 (m, 1H), 5.84 (tdt, J = 17.1, 10.1, 7.0 Hz, 2H), 5.11 – 4.91 (m, 4H), 4.58 (d, J = 1.6 Hz, 2H), 3.83 (qd, J = 10.3, 7.2 Hz, 2H), 2.65 (dd, J = 15.4, 7.1 Hz, 1H), 2.29 – 2.16 (m, 2H), 1.81 (ddt, J = 15.4, 5.7, 1.8 Hz, 1H), 1.72 (qd, J = 7.1, 4.1 Hz, 1H), 1.65 (tt, J = 6.9, 3.4 Hz, 1H), 1.11 (d, J = 6.8 Hz, 3H), 1.02 (t, J = 7.3 Hz, 1H), 0.82 (d, J = 7.2 Hz, 3H).

^{13}C NMR (126 MHz, Chloroform-*d*): δ 139.07, 137.62, 137.29, 128.40, 127.67, 127.48, 115.54, 115.05, 72.67, 66.67, 41.28, 37.54, 35.19, 34.94, 34.42, 33.00, 31.96, 17.98, 14.95.

HRMS (APCI): m/z calculated for $C_{21}H_{27}O$ $[M-H]^+$: 295.2056, found 295.2035.

(±)-2-((1R,2R)-1-Allyl-2-((benzyloxy)methyl)cyclopropyl)ethyl diethyl phosphate (4)

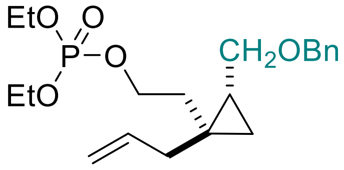

The title compound was prepared accoring to the standard procedure and quenced at -40 °C prior to heating.

Scale, physical description, yield: 0.23 mmol, colorless oil (51 mg, 0.13 mmol, 56% yield).

R*f* = 0.6 (PE/EtOAc 35:65).

^{1}H NMR (500 MHz, Chloroform-*d*): δ 7.33 (d, J = 4.4 Hz, 4H), 7.31 – 7.27 (m, 1H), 5.78 (ddt, J = 17.1, 10.1, 7.0 Hz, 1H), 5.13 – 4.96 (m, 2H), 4.51 (d, J = 2.0 Hz, 2H), 4.18 (tt, J = 9.6, 6.5 Hz, 1H), 4.09 (dqd, J = 14.8, 8.2, 1.9 Hz, 5H), 3.58 (dd, J = 10.4, 6.2 Hz, 1H), 3.36 (dd, J = 10.5, 8.4 Hz, 1H), 2.05 (dd, J = 14.7, 7.1 Hz, 1H), 1.97 (dd, J = 14.7, 6.8 Hz, 1H), 1.75 (dddd, J = 39.7, 14.5, 8.8, 6.1 Hz, 2H), 1.31 (tt, J = 7.0, 1.1 Hz, 6H), 1.04 (tt, J = 8.5, 5.9 Hz, 1H), 0.61 (dd, J = 8.8, 4.8 Hz, 1H), 0.25 (t, J = 5.2 Hz, 1H).

^{13}C NMR (126 MHz, Chloroform-*d*): δ 138.57, 135.69, 128.47, 127.78, 127.64, 116.95, 72.79, 70.56, δ 65.92 (d, *J* = 6.0 Hz), 63.74 (d, *J* = 5.9 Hz), 41.86, 31.55 (d, *J* = 6.9 Hz), 22.63, 20.98, 16.28 (d, *J* = 6.8 Hz), 15.98.

HRMS (APCI): m/z calculated for $C_{20}H_{32}O_5P$ $[M+H]^+$: 383.1987, found 383.1962.

2.2 Functionalization of polysubstituted housanes.

Iridium-catalyzed hydroboration oxidation:[7]

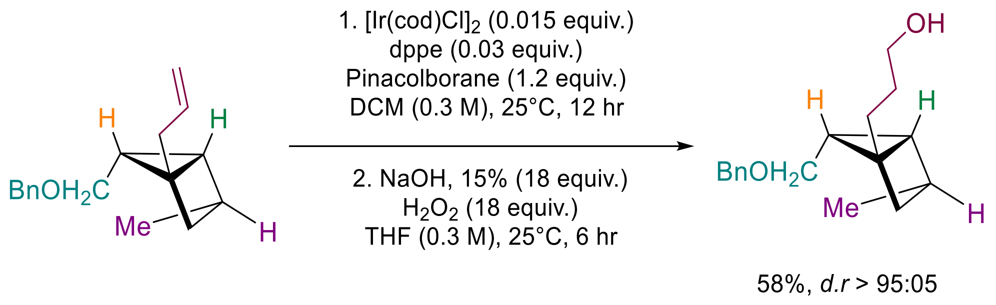

In a flame-dried Schlenk tube, $[Ir(cod)Cl]_2$ (0.015 equiv.) and dppe (0.03 equiv.) were added, and the flask was purged with argon. DCM (0.3 M), pinacolborane (1.2 equiv.), and housane **6n** (1.0 equiv.) were added successively at room temperature. The reaction mixture was stirred at room temperature overnight.

Upon completion, the solvent was removed under reduced pressure, and THF (0.3 M) was added to dissolve the residue. To this solution, 15% aqueous NaOH (18 equiv.) and H_2O_2 (18 equiv., 30% w/w) were added, and the mixture was stirred at room temperature for 6 h. The reaction was quenched by addition of saturated aqueous NH_4Cl, and the mixture was extracted with Et_2O (3 ×). The combined organic layers were dried over Na_2SO_4, filtered, and concentrated in vacuo. The crude product was purified by column chromatography on silica gel (eluting with 25% Et_2O in pentane) to afford the corresponding alcohol.

(±)-3-(1S,3R,4S,5R)-5-((Benzyloxy)methyl)-3-methylbicyclo[2.1.0]pentan-1-yl)propan-1-ol (7a)

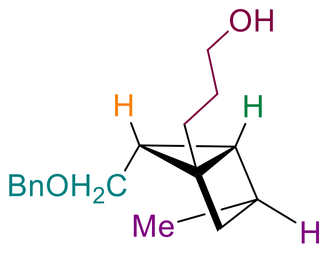

Scale, physical description, yield: 0.7 mmol, colorless oil, 58% yield.

Rf = 0.5 (PE/Et_2O 6:4).

^{1}H NMR (500 MHz, Chloroform-*d*): δ 7.29 – 7.22 (m, 4H), 7.20 – 7.15 (m, 1H), 4.48 (s, 2H), 3.74 – 3.64 (m, 2H), 3.52 (qt, J = 10.7, 6.3 Hz, 2H), 2.46 (dqt, J = 9.8, 7.2, 4.8 Hz, 1H), 1.89 (dd, J = 11.7, 10.4 Hz, 1H), 1.68 (ddd, J = 13.8, 8.2, 5.6 Hz, 1H), 1.57 – 1.44 (m, 2H), 1.42 (ddd, J = 5.9, 4.6, 1.1 Hz, 1H), 1.34 – 1.26 (m, 1H), 1.22 (ddd, J = 11.7, 4.9, 1.2 Hz, 1H), 0.89 (dt, J = 8.6, 6.0 Hz, 1H), 0.68 (d, J = 7.2 Hz, 3H).

^{13}C NMR (126 MHz, Chloroform-*d*): 138.55, 128.50, 127.99, 127.73, 73.04, 66.93, 62.50, 30.49, 30.45, 28.79, 28.07, 26.04, 25.97, 25.48, 17.79.

HRMS (APCI): m/z calculated for $C_{18}H_{27}O_2$ $[M+CH_3]^+$: 275.2006, found 275.2003.

Metathesis reaction:[8]

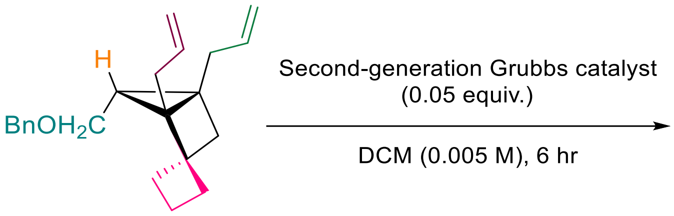

quantitative, *d.r* > 95:05

In an oven-dried, open-air flask, housane **6l** (1.0 equiv.) was dissolved in DCM (0.005 M). Grubbs' second-generation catalyst (0.05 equiv.) was added, and the reaction mixture was stirred at room temperature for 5 h. Upon completion, the solvent was removed under reduced pressure, and the crude product was purified by column chromatography on silica gel (eluting with 0.5% Et_2O in pentane) to afford the corresponding tricyclo[4.2.1]nonane.

(±)-(1'S,6'S,9'R)-9'-((Benzyloxy)methyl)spiro[cyclobutane-1,7'-tricyclo[4.2.1.01,6]nonan]-3'-ene (7b)

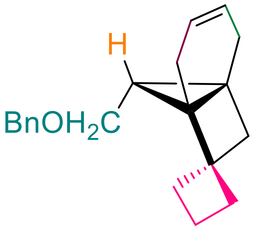

Scale, physical description, yield: 0.04 mmol, colorless oil, 100% yield.

R*f* = 0.55 (PE/Et_2O 96:4).

^{1}H NMR (500 MHz, Chloroform-*d*): δ 7.30 – 7.21 (m, 4H), 7.20 – 7.15 (m, 1H), 5.41 (ddt, J = 9.0, 4.3, 2.1 Hz, 1H), 5.35 (ddt, J = 10.4, 4.6, 2.3 Hz, 1H), 4.46 (s, 2H), 3.65 – 3.54 (m, 2H), 2.39 – 2.29 (m, 1H), 2.29 – 2.21 (m, 1H), 2.14 (m, 1H), 2.06 (m, 1H), 1.97 – 1.88 (m, 1H), 1.77 – 1.54 (m, 6H), 1.24 – 1.08 (m, 2H).

^{13}C NMR (126 MHz, Chloroform-*d*): δ 138.97, 128.45, 127.93, 127.59, 123.87, 123.37, 72.57, 66.14, 44.51, 41.02, 32.76, 32.33, 30.57, 27.49, 27.15, 22.28, 18.35, 16.80.

HRMS (APCI): m/z calculated for $C_{20}H_{23}O$ [M-H]$^+$: 279.1749, found 279.1738.

2.3 Determination of the relative configuration of housanes

Previous studies indicate that the relative configuration of housane backbones can be determined from ^{1}H NMR chemical shifts and J-couplings, as these parameters depend primarily on the relative orientation rather than on the electronic nature of the substituents.[9]

Key considerations for interpreting chemical shifts and J-couplings are as follows:

- Exo-substituents has higher chemical shift than endo-substituents due to the shielding effect of the cyclopropyl ring (H^3 and H^4, respectively).
- $J(H^1\text{-}H^3) \approx$ 4-5 Hz, while $J(H^1\text{-}H^4) \approx$ 0 Hz.
- $J(H^1\text{-}H^2) \approx$ 5-6 Hz, while $J(H^1\text{-}H^5) \approx$ 1-1.5 Hz.

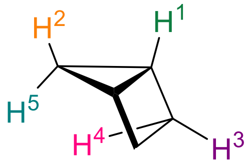

For each of the substrates below, the chemical shifts and J-couplings of all protons were analyzed based on ^{1}H NMR, ^{13}C NMR, COSY (and HSQC) spectra, and the relative configuration was determined accordingly. For several compounds, indicative signals observed in NOESY spectra provided additional support for the assignments.

- Compund **6a**:

  The chemical shifts and J-couplings of the relevant protons are summarized in Table S1. Proton H^1 appears as doublet of doublets with coupling constants of J = 6.5, 5.4 Hz. This splitting pattern corresponds to J(H^1–H^2) = 6.5 Hz and J(H^1–H^8) = 5.4 Hz, indicating a syn relationship between H^1 and H^2. In agreement with this assignment, proton H^2 displays a triplet of doublets with coupling constants of J = 7.4 Hz and 6.7 Hz, corresponding to coupling constants J(H^5–H^2) = 7.4 Hz and J(H^1–H^2) = 6.7 Hz. Accordingly, the relative configuration of **6a** was assigned as shown in Figure S2.

  The proton assignments were established by analysis of the COSY spectrum (Figure S2).

Table S1: ^{1}H NMR chemical shifts and J-couplings for the relative configuration assignment of housane **6a**

Proton	Chemical Shift	Multiplicity	Integration
H^1	1.62	Doublet of doublets, J=6.5, 5.4 Hz	1H
H^2	1.03	Triplet of doublets, J=7.4, 6.7 Hz	1H
H^3	1.30	Doublet of doublets, J=11.1, 5.2 Hz	1H
H^4	1.49-1.41	Multiplet	1H
H^7	1.86	Doublet of doublet of doublets, J=11.1, 11.1, 3.9 Hz	1H
H^8	2.01	Doublet of doublet of doublet of doublets, J=10.9, 8.3, 4.4, 4.2 Hz	1H
H^5	3.76-3.61	Multiplet	2H
H^6	2.31<br>2.15	Doublet of doublets, J=15.0, 6.8 Hz<br>Doublet of doublets, J=15.0, 6.6 Hz	1H<br>1H

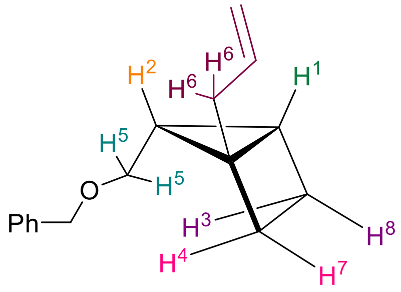

Figure S1: Relative configuration of housane **6a**.

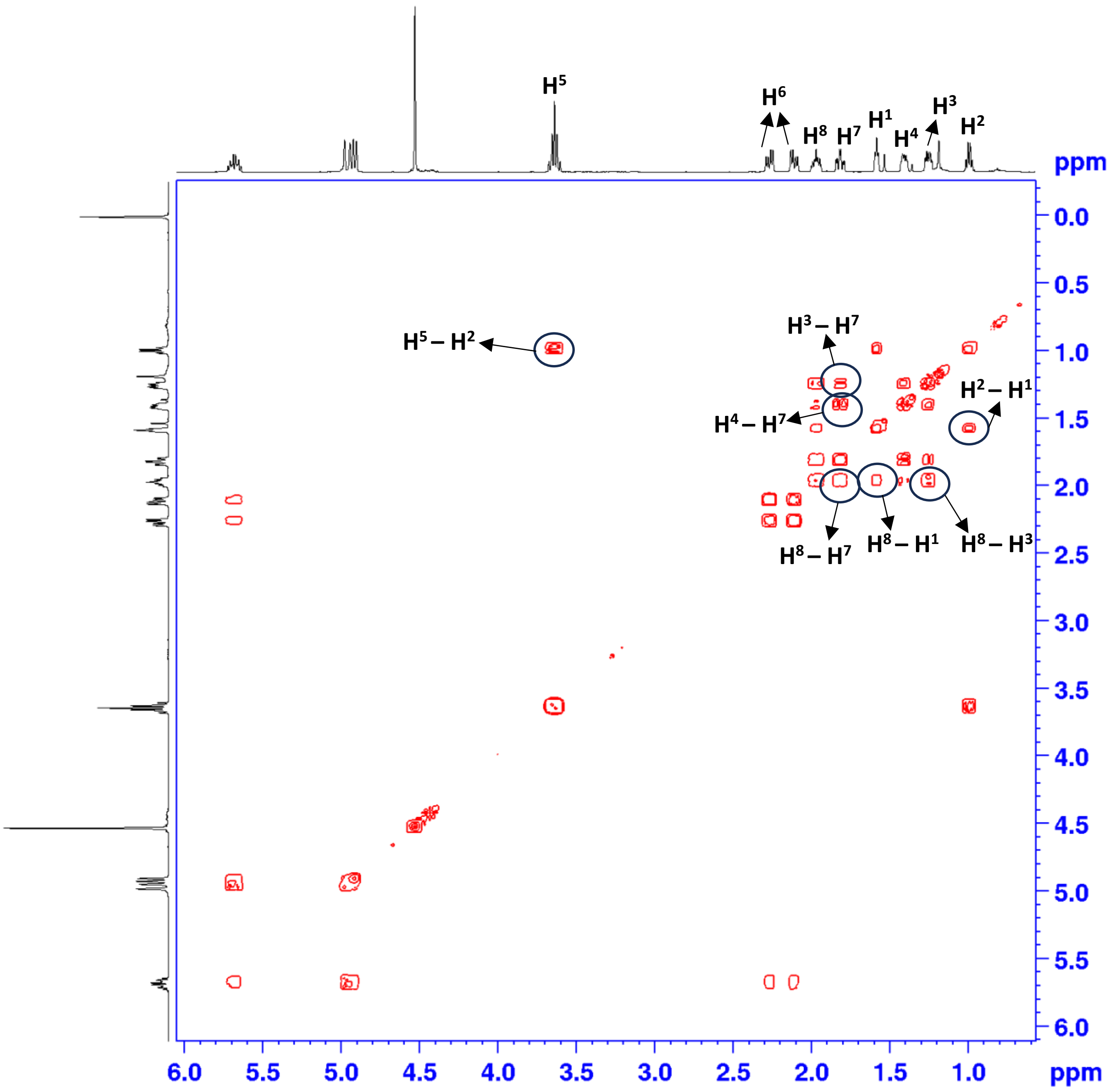

Figure S2: COSY spectrum for compound **6a**.

- Compund **6m**:

  The chemical shifts and J-couplings of the relevant protons are summarized in Table S2. Proton H^1 displays as doublet of doublets with coupling constants of J = 5.9, 0.9 Hz. This splitting pattern corresponds to $J(H^1–H^2)$ = 5.9 Hz and $J(H^1–H^3)$ = 0.9 Hz, indicating that H^3 adopts an endo orientation. Accordingly, the relative configuration of **6m** was assigned as shown in Figure S3. Moreover, comparison with compound **6n** reveals a downfield shift of the methyl group, consistent with an exo orientation.

  The proton assignments were established by analysis of the COSY spectrum (Figure S4).

Table S2: ^{1}H NMR chemical shifts and J-couplings for the relative configuration assignment of housane **6m**

Proton	Chemical Shift	Multiplicity	Integration
H^1	1.48	Doublet of doublets, J=5.9, 0.9 Hz	1H
H^2 (+Me)	1.17	Multiplet	4H
H^3 (+H^7)	1.69	Triplet of doublets, J=16.5, 7.3 Hz	2H
H^4	1.30-1.24	Multiplet	1H
H^7 (+H^3)	1.69	Triplet of doublets, J=16.5, 7.3 Hz	2H
Me (+H^2)	1.17	Multiplet	4H
H^5	3.77-3.63	Multiplet	2H
H^6	2.29<br>2.23	Doublet of doublets, J=14.8, 6.9 Hz<br>Doublet of doublets, J=14.9, 6.8 Hz	1H<br>1H

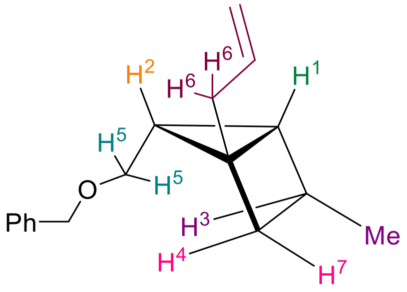

Figure S3: Relative configuration of housane **6m**.

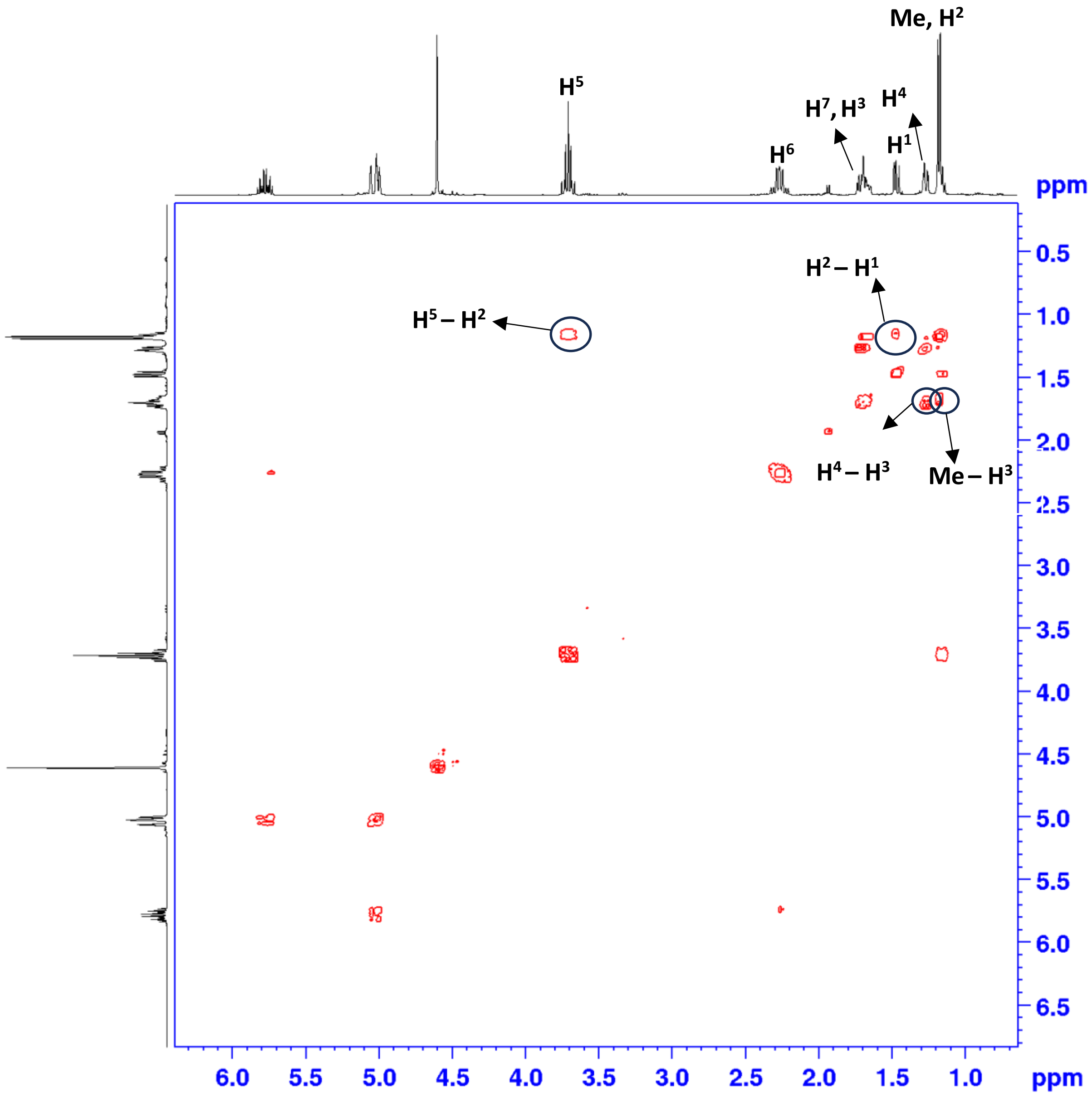

Figure S4: COSY spectrum for compound **6m**.

- Compund **6n**:

The chemical shifts and J-couplings of the relevant protons are summarized in Table S3. Proton H^1 displays a doublet of doublets with coupling constants of J = 6.3, 5.4 Hz. This splitting pattern corresponds to $J(H^1–H^2)$ = 6.3 Hz and $J(H^1–H^3)$ = 5.4 Hz, indicating that H^3 adopts an exo orientation. Accordingly, the relative configuration of **6n** was assigned as shown in Figure S5.

Moreover, comparison with compound **6m** reveals an upfield shift of the methyl group, consistent with an endo orientation.

The proton assignments were established by analysis of the COSY spectrum (Figure S6).

Table S3: ^{1}H NMR chemical shifts and J-couplings for the relative configuration assignment of housane **6n**

Proton	Chemical Shift	Multiplicity	Integration
H^1	1.61-1.56	Doublet of doublets, J=6.3, 5.4 Hz	1H
H^2	1.05	Doublet of triplets, J=8.4, 6.4 Hz	1H
H^3	2.58	Doublet of doublet of triplets, J=10.2, 7.4, 4.7 Hz	1H
H^4	1.34	Doublet of doublets, J=11.8, 4.7 Hz	1H
H^7	2.02	Doublet of doublets, J=11.0, 11.0 Hz	1H
Me	0.83	Doublet, J=7.2 Hz	3H
H^5	3.89-3.84	Multiplet	1H
	3.80	Doublet of doublets, J=10.3, 6.7 Hz	1H
H^6	2.33	Doublet of doublets, J=14.8, 6.8 Hz	1H
	2.23	Doublet of doublets, J=14.9, 6.9 Hz	1H

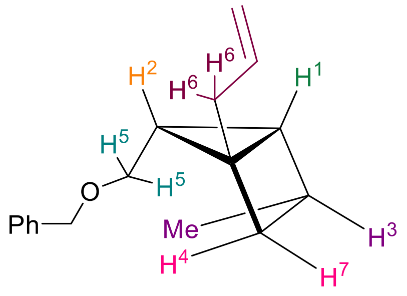

Figure S5: Relative configuration of housane **6n**.

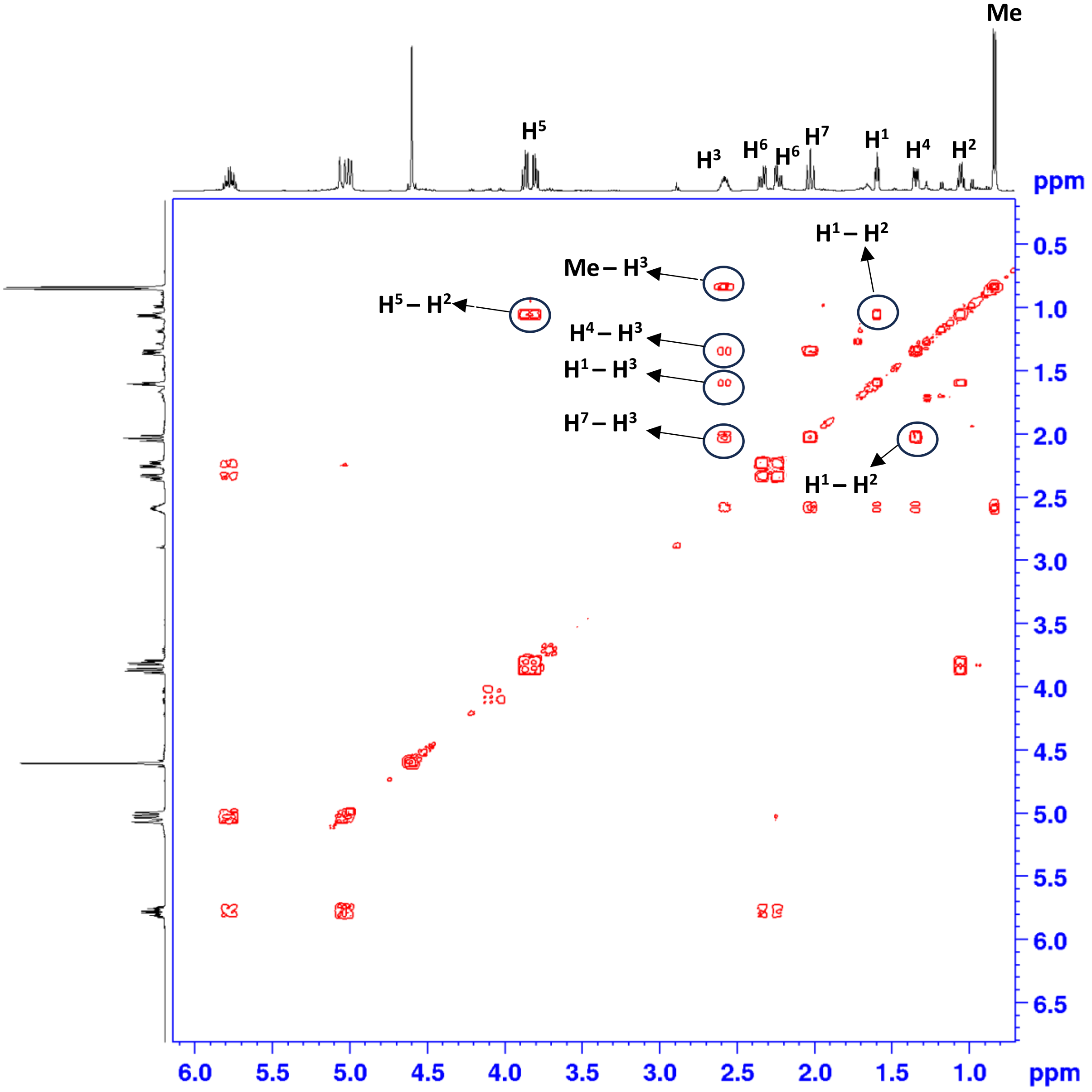

Figure S6: COSY spectrum for compound **6n**.

- Compund **6p**:

  The chemical shifts and J-couplings of the relevant protons are summarized in Table S4. In this case, the coupling constants for H^1 could not be determined unambiguously. Nevertheless, comparison of the chemical shifts of Me^1 and Me^2, as well as H^3 and H^4, suggests that Me^1 and H^4 are exo-substituted, whereas Me^2 and H^3 are endo-substituted.

  Additional support for this assignment is provided by the NOESY spectrum, which shows correlations between the ether protons H^5 and Me^2, as well as H^3 (Figure S9). Furthermore, comparison with **6q**, that unambiguously determined as 2,3-exo, endo, supports that **6p** has 2,3-endo, exo configuration.

  The proton assignments were established by analysis of the COSY spectrum (Figure S8).

Table S4: ^{1}H NMR chemical shifts and J-couplings for the relative configuration assignment of housane **6p**

Proton	Chemical Shift	Multiplicity	Integration
H^2 (+Me^1)	1.17-1.09	Multiplet	4H
H^1	1.44-1.40	Multiplet	1H
H^3	1.47	Quartet of doublets, J=6.8, 4.3 Hz	1H
H^4	1.75-1.68	Multiplet	1H
Me^1 (+H^2)	1.17-1.09	Multiplet	4H
Me^2	0.81	Doublet, J=7.2 Hz	3H
H^5	3.81-3.77	Multiplet	2H
H^6	2.31-2.25	Multiplet	1H
	2.21	Doublet of double of triplets, J=14.7, 6.9, 1.4 Hz	1H

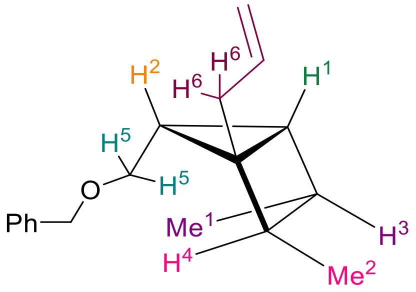

Figure S7: Relative configuration of housane **6p**.

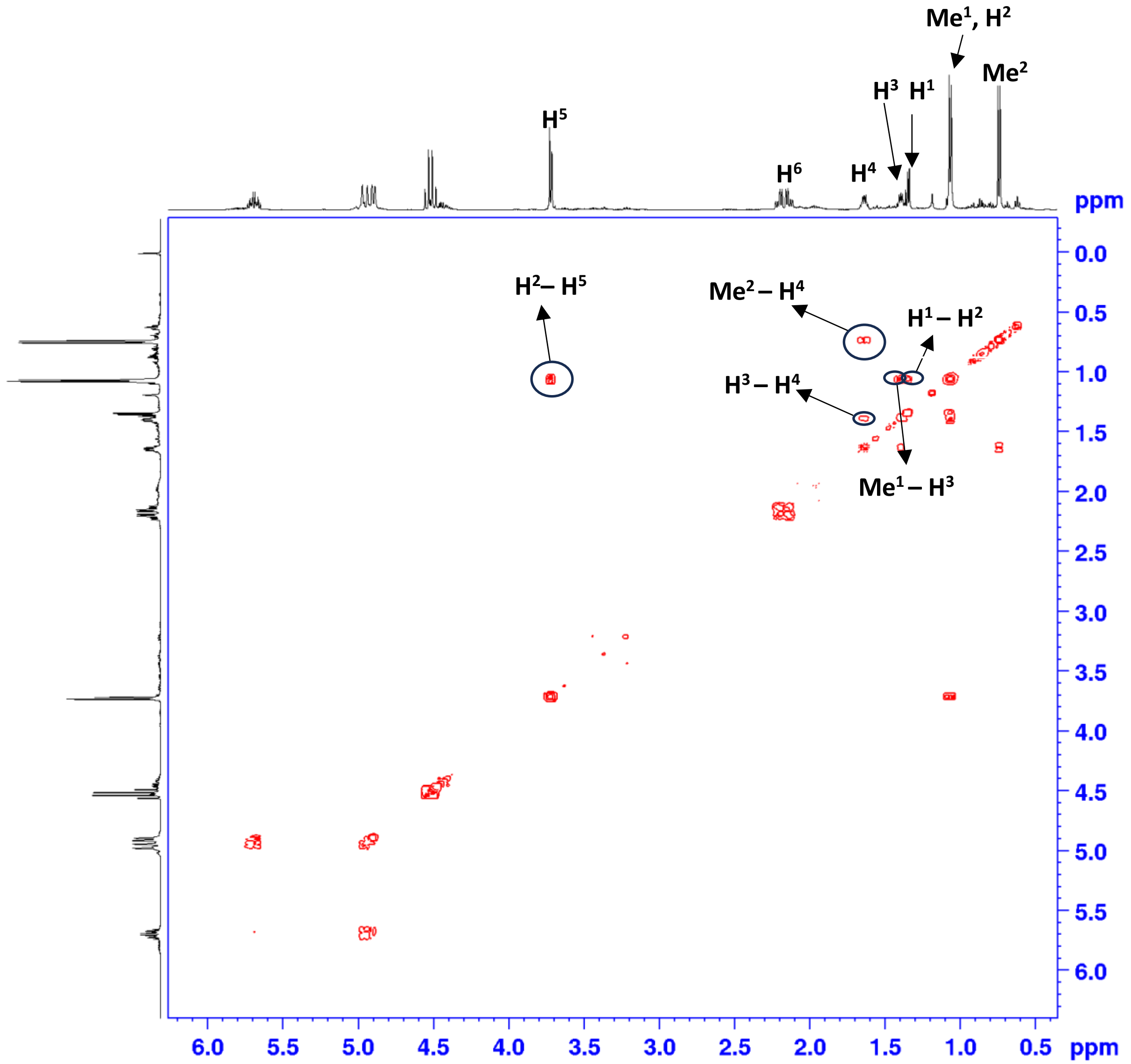

Figure S8: COSY spectrum for compound **6p**.

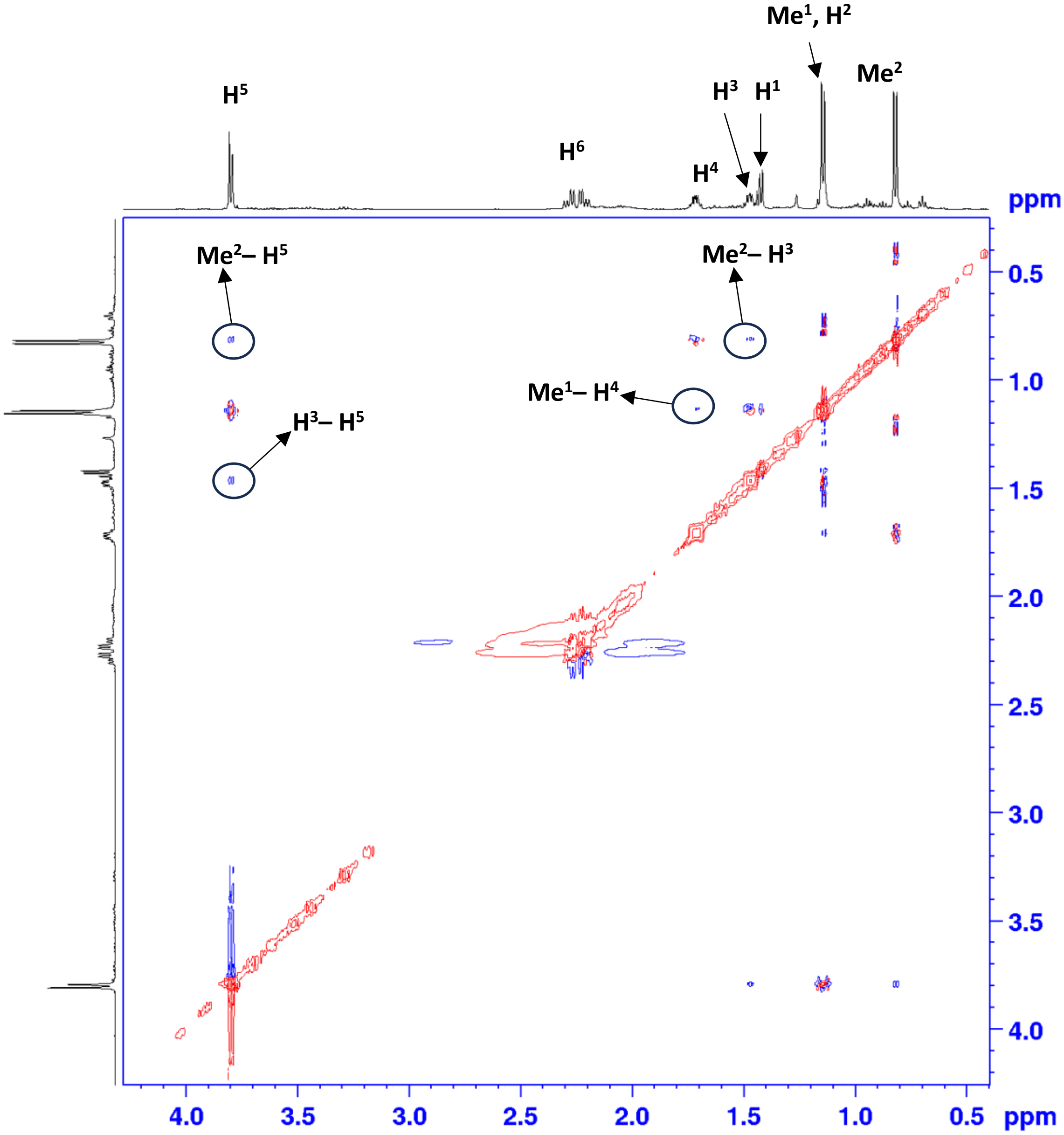

Figure S9: NOESY spectrum for compound **6p**.

- Compund **6q**:

The chemical shifts and J-couplings of the relevant protons are summarized in Figure S7. Proton H^1 displays a doublet of doublets with coupling constants of J=5.8, 4.7 Hz. This splitting pattern corresponds to $J(H^1–H^2)$ = 5.8 Hz and $J(H^1–H^3)$ = 4.7 Hz, indicating that H^3 adopts an exo orientation. Accordingly, the relative configuration of **6q** was assigned as shown in Figure S10. Moreover, comparison between the chemical shifts of Me^1 and Me^2, as well as H^3 and H^4, suggest that Me^1 and H^4 are endo-substituents, while Me^2 and H^3 are exo-substituents. Additional support for this assignment is provided by the NOESY spectrum, which shows correlations between the ether protons H^5 and Me^1, as well as H^4 (Figure S12).

The proton assignments were established by analysis of the COSY spectrum (Figure S11).

Table S5: ^{1}H NMR chemical shifts and J-couplings for the relative configuration assignment of housane **6q**

Substituent	Chemical Shift	Multiplicity	Integration
H^1	1.60	Doublet of doublets, J=5.8, 4.7 Hz	1H
H^2	1.11	Multiplet	1H
H^3 (+H^6)	1.92-1.82	Multiplet	2H
H^4	1.68	Quartet of doublets, J=6.9, 4.3 Hz	1H
Me^1	0.83	Doublet, J=7.2 Hz	3H
Me^2	1.14	Doublet, J=6.9 Hz	3H
H^5	3.87	Doublet of doublets, J=10.2, 7.8 Hz	1H
	3.78	Doublet of doublets, J=10.3, 7.0 Hz	1H
H^6 (+H^3)	1.92-1.82	Multiplet	2H
	2.68	Doublet of double of triplets, J=15.2, 7.3, 1.3 Hz	1H

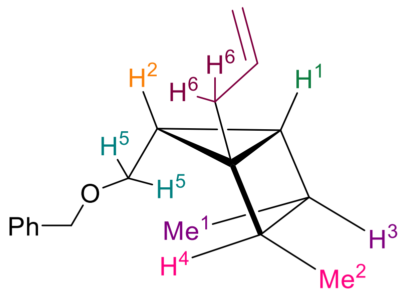

Figure S10: Relative configuration of housane **6q**.

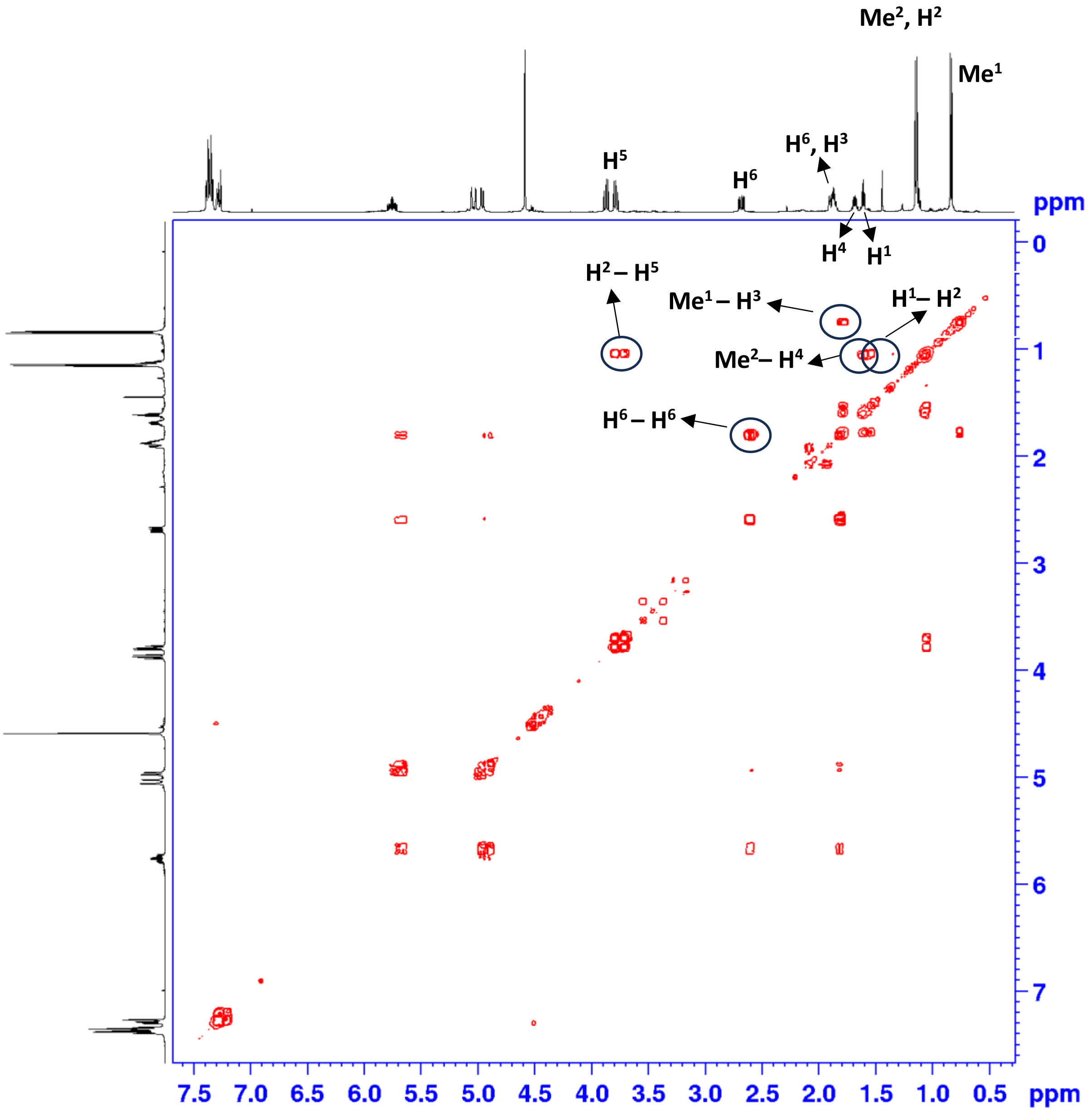

Figure S11: COSY spectrum for compound **6q**.

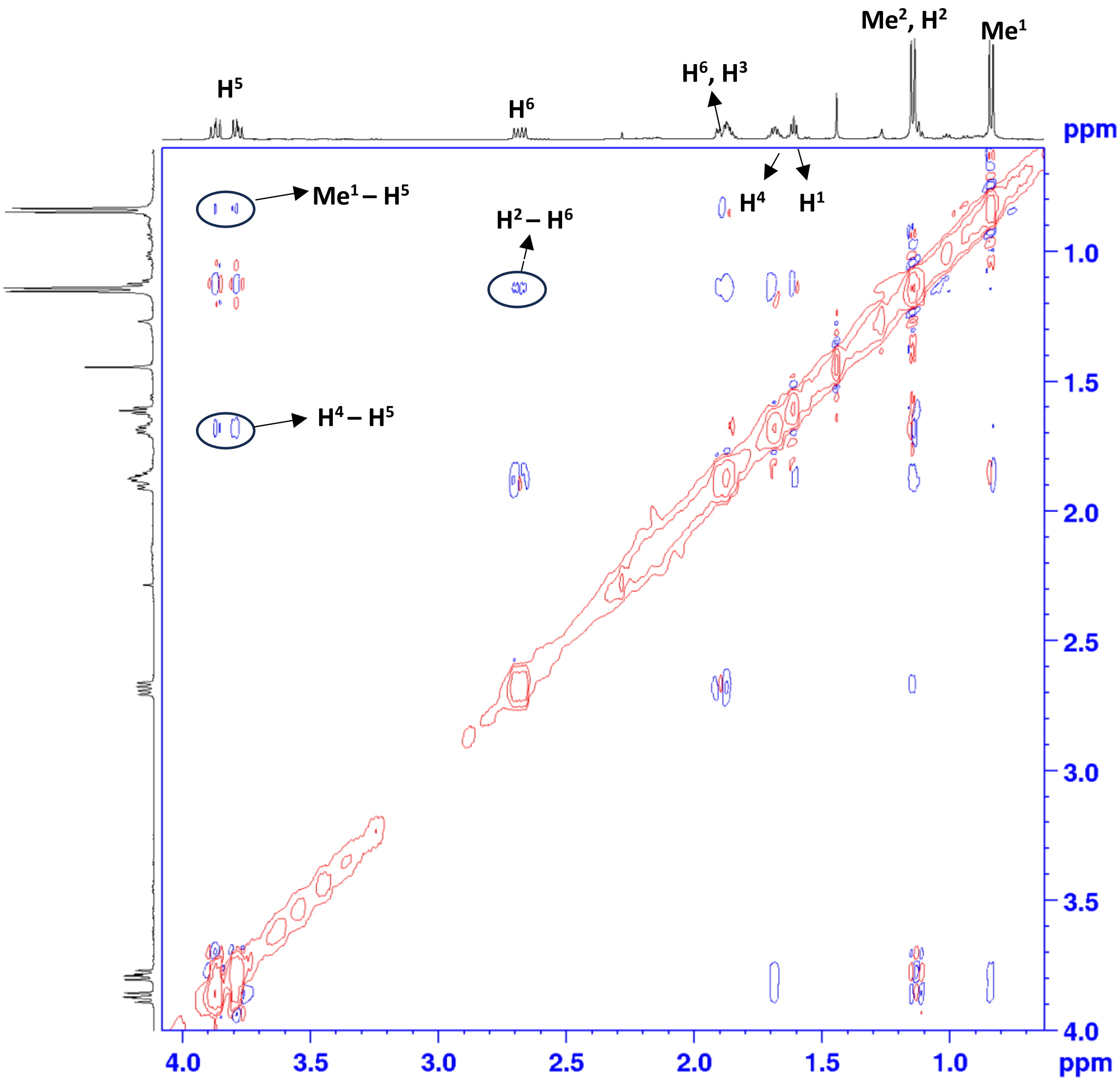

Figure S12: NOESY spectrum for compound **6q**.

3. References.

1. Cohen, Y.; Augustin, A. U.; Levy, L.; Jones, P. G.; Werz, D. B.; Marek, I. Regio- and Diastereoselective Copper-Catalyzed Carbomagnesiation for the Synthesis of Penta- and Hexa-Substituted Cyclopropanes. *Angew. Chem. Int. Ed.* **2021**, *60* , 11804-11808.
2. Pavlickova, T.; Orbach, N.; Kaushansky, A.; Marek, I. Vicinal Stereocenter Construction via α-Boryl Carbanions from Borylated Cyclopropanes. *J. Am. Chem. Soc.* **2025***, 147,* 41204–41209.
3. Hirbawi, N.; Lin, P. C.; Jarvo, E. R. Halogenation Reactions of Alkyl Alcohols Employing Methyl Grignard Reagents. *J. Org. Chem.* **2022**, *87*, 12352–12369.
4. Block, E.; Orf, H. W.; Winter, R. E. K. The nuclear magnetic resonance spectra of bicyclo[2.1.0]pentanes: A comparative study. *Tetrahedron* **1972**, *28*, 4483–4496.
5. Augustin, A. U.; Di Silvio, S.; Marek, I. Borylated cyclopropanes as spring-loaded entities: Access to vicinal tertiary and quaternary carbon stereocenters in acyclic systems. *J. Am. Chem. Soc.* **2022**, *144*, 16298-16302.
6. Levin, A.; Marek, I. Cyclopropenyllithiums as a new source of 1, 1-bismetalated cyclopropyl derivatives. *Chem. Commun.* **2008**, *36*, 4300–4302.
7. Kesavulu, G.; Silambarasan, K.; Prasad, K. R. Total synthesis of the macrolactone AKML-B. *Tetrahedron* **2024**, *166*, 134205.
8. Yamamoto, Y.; Fujikawa, R.; Umemoto, T.; Miyaura, N. Iridium-catalyzed hydroboration of alkenes with pinacolborane. *Tetrahedron* **2004**, *60*, 10695–10700.
9. Block, E.; Orf, H. W.; Winter, R. E. K. The nuclear magnetic resonance spectra of bicyclo[2.1.0]pentanes: A comparative study. *Tetrahedron* **1972**, *28*, 4483–4496.

4. NMR spectra of new compounds

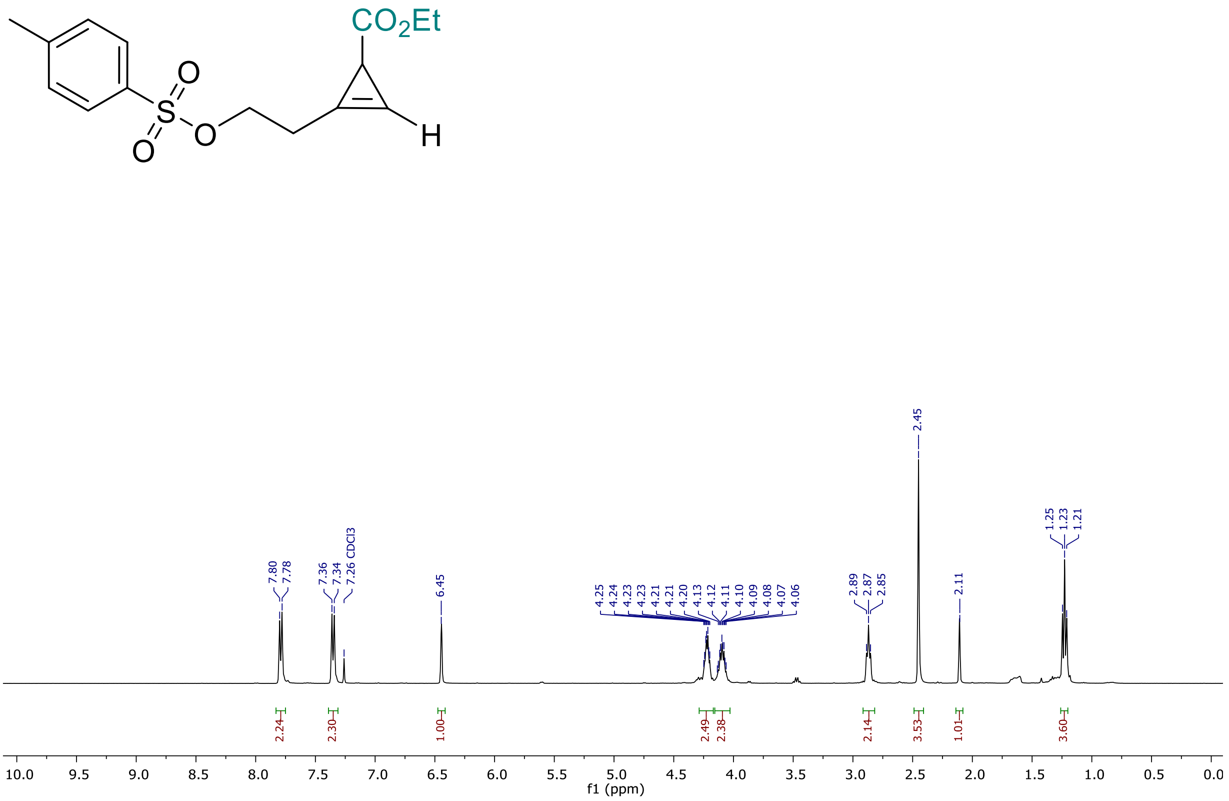
CO2Et
O
S
O
O
H
7.80
7.78
7.36
7.34
7.26 CDCl3
6.45
4.25
4.24
4.23
4.23
4.21
4.21
4.20
4.13
4.12
4.11
4.10
4.09
4.08
4.07
4.06
2.89
2.87
2.85
2.45
2.11
1.25
1.23
1.21
2.24
2.30
1.00
2.49
2.38
2.14
3.53
1.01
3.60
10.0 9.5 9.0 8.5 8.0 7.5 7.0 6.5 6.0 5.5 5.0 4.5 4.0 3.5 3.0 2.5 2.0 1.5 1.0 0.5 0.0
f1 (ppm)

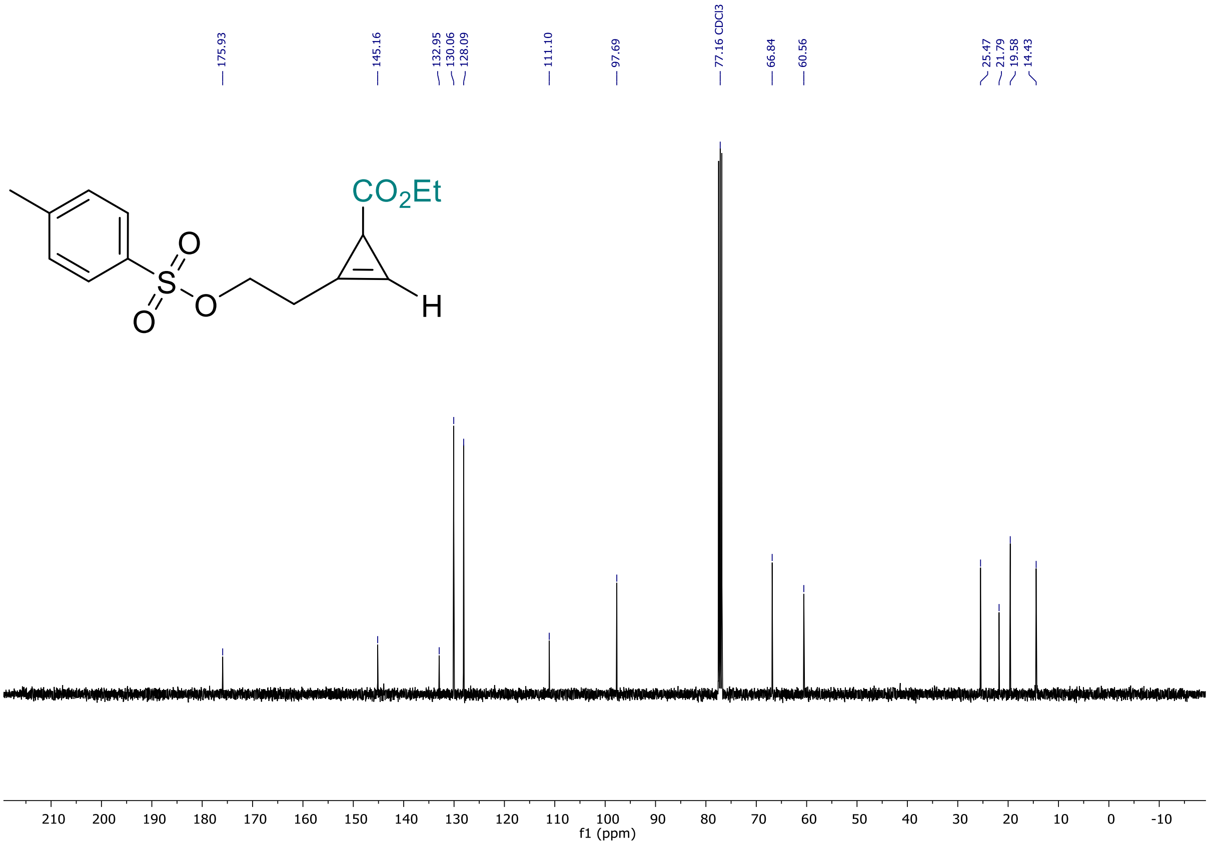
175.93
145.16
132.95
130.06
128.09
111.10
97.69
77.16 CDCl3
66.84
60.56
25.47
21.79
19.58
14.43
CO2Et
O
S
O
O
H
210 200 190 180 170 160 150 140 130 120 110 100 90 80 70 60 50 40 30 20 10 0 -10
f1 (ppm)

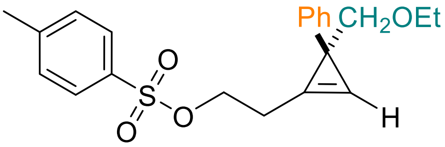
Ph
CH2OEt
O
S
O
O
H

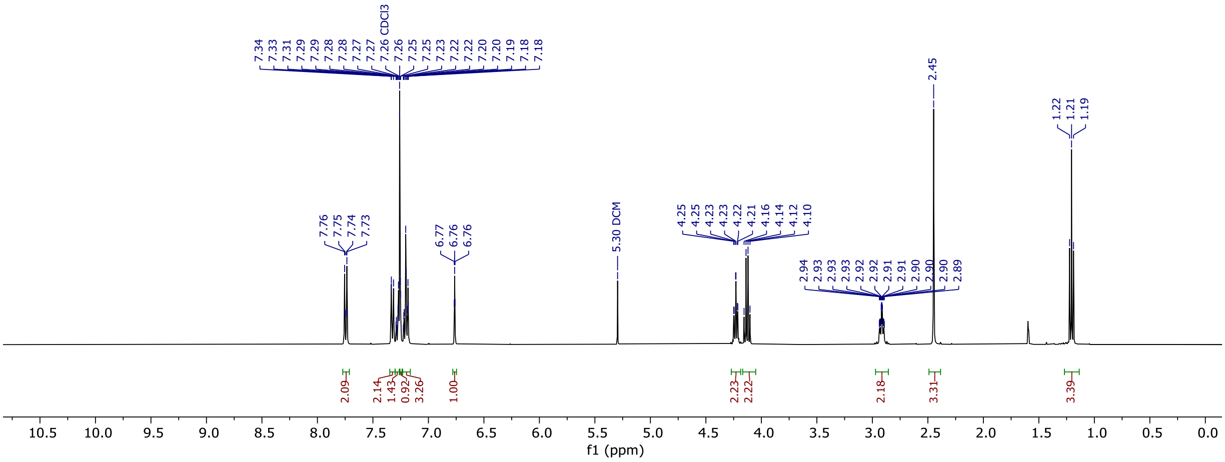
7.34
7.33
7.31
7.29
7.29
7.28
7.28
7.27
7.27
7.26 CDCl3
7.26
7.25
7.25
7.23
7.22
7.22
7.20
7.20
7.19
7.18
7.18
2.45
1.22
1.21
1.19
7.76
7.75
7.74
7.73
6.77
6.76
6.76
5.30 DCM
4.25
4.25
4.23
4.23
4.22
4.21
4.16
4.14
4.12
4.10
2.94
2.93
2.93
2.93
2.92
2.92
2.91
2.91
2.90
2.90
2.90
2.89
2.09
2.14
1.43
0.92
3.26
1.00
2.23
2.22
2.18
3.31
3.39
f1 (ppm)

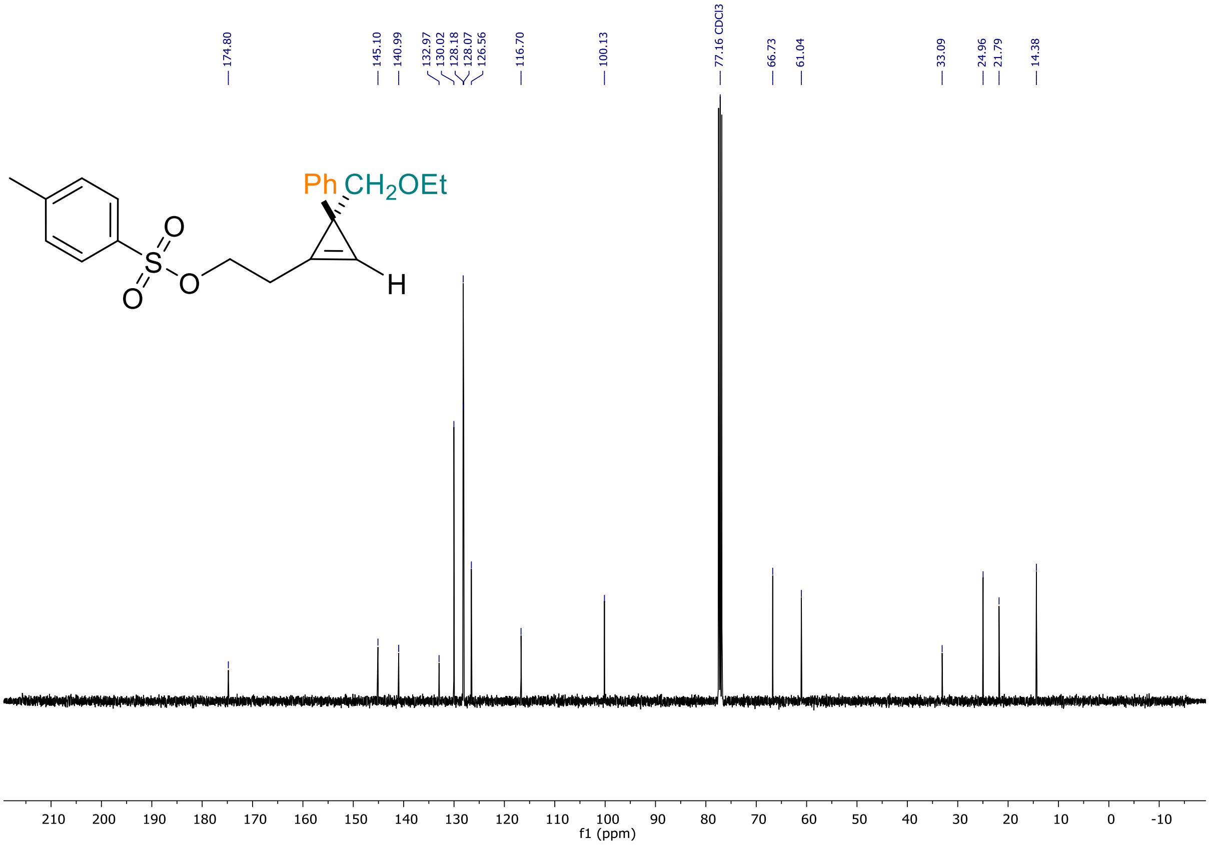
174.80
145.10
140.99
132.97
130.02
128.18
128.07
126.56
116.70
100.13
77.16 CDCl3
66.73
61.04
33.09
24.96
21.79
14.38
Ph
CH2OEt
O
S
O
O
H
f1 (ppm)

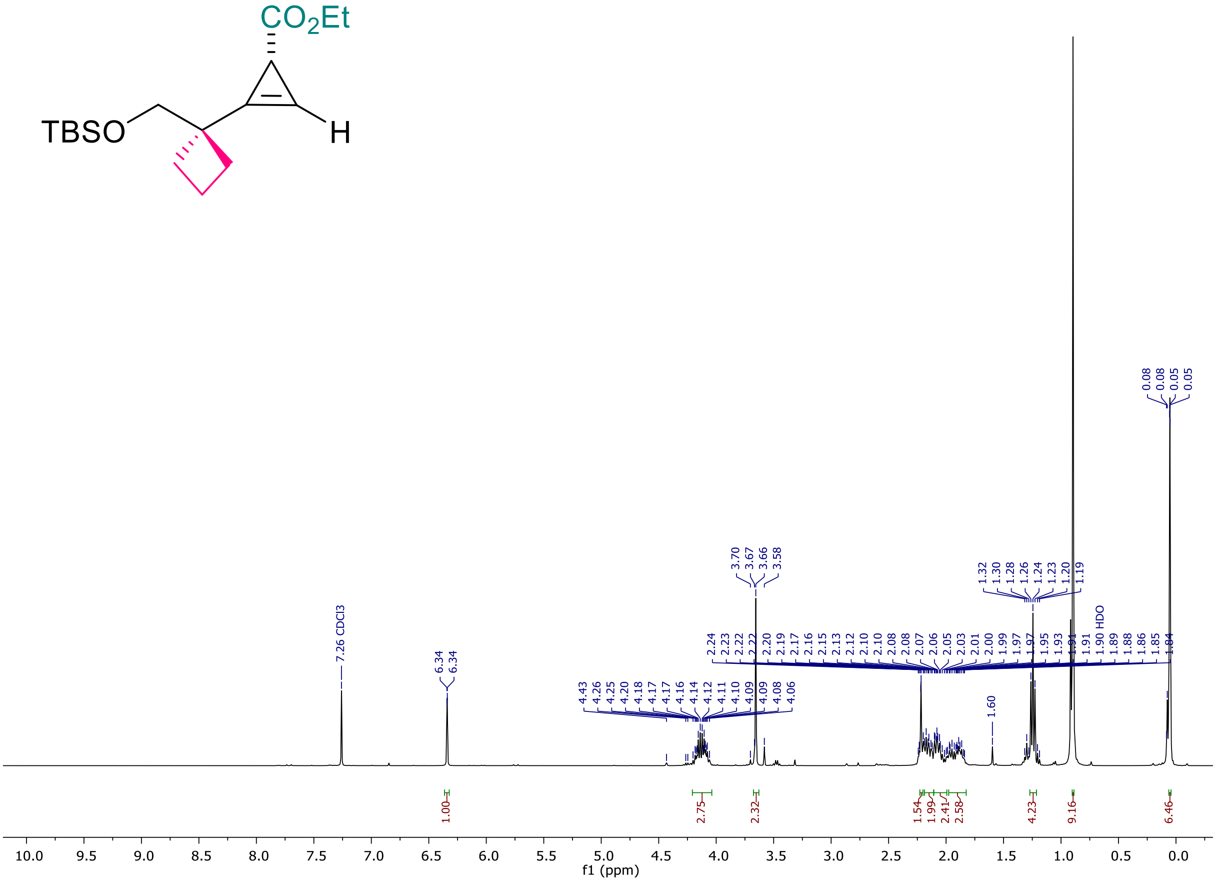
CO2Et
TBSO
H
7.26 CDCl3
6.34
6.34
3.70
3.67
3.66
3.58
1.60
1.90 HDO
1.00
2.75
2.32
1.54
1.99
2.41
2.58
4.23
9.16
6.46
10.0 9.5 9.0 8.5 8.0 7.5 7.0 6.5 6.0 5.5 5.0 4.5 4.0 3.5 3.0 2.5 2.0 1.5 1.0 0.5 0.0
f1 (ppm)

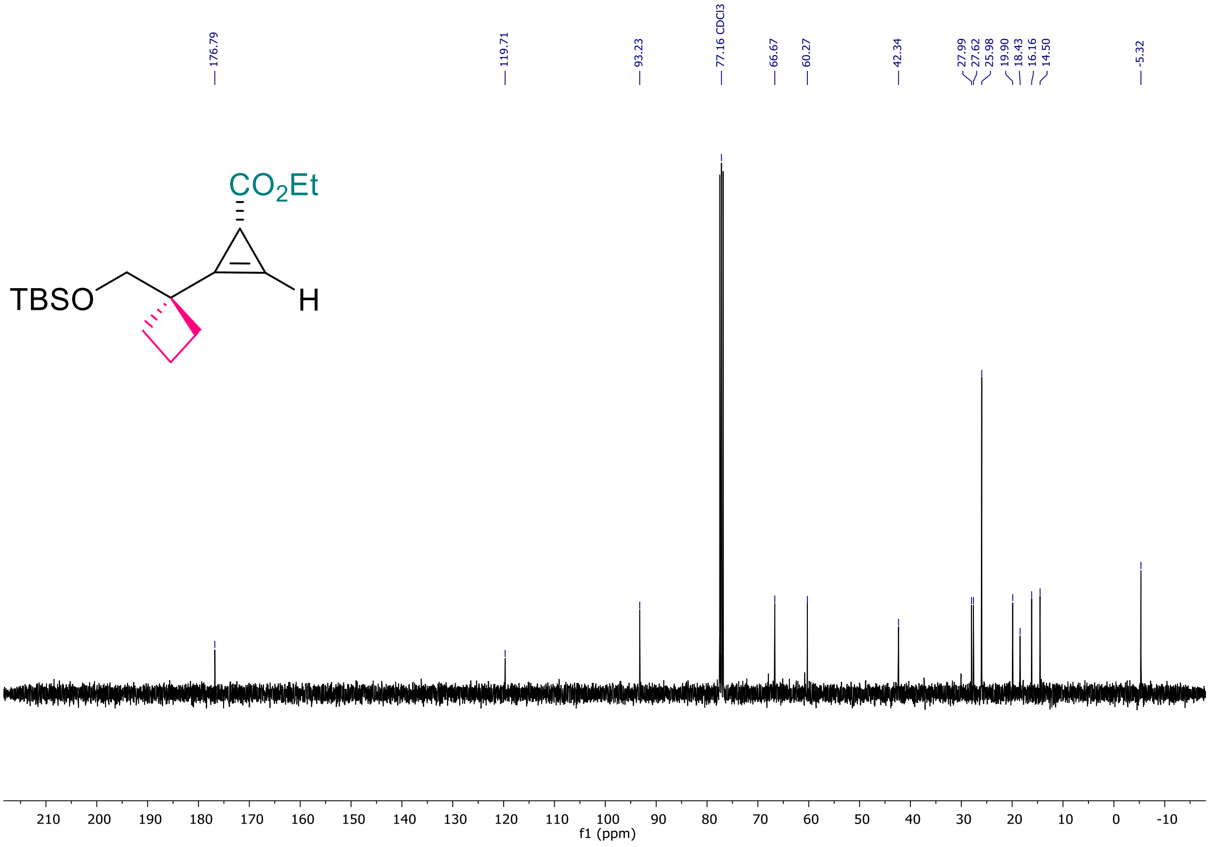
176.79
119.71
93.23
77.16 CDCl3
66.67
60.27
42.34
27.99
27.62
25.98
19.90
18.43
16.16
14.50
-5.32
CO2Et
TBSO
H
210 200 190 180 170 160 150 140 130 120 110 100 90 80 70 60 50 40 30 20 10 0 -10
f1 (ppm)

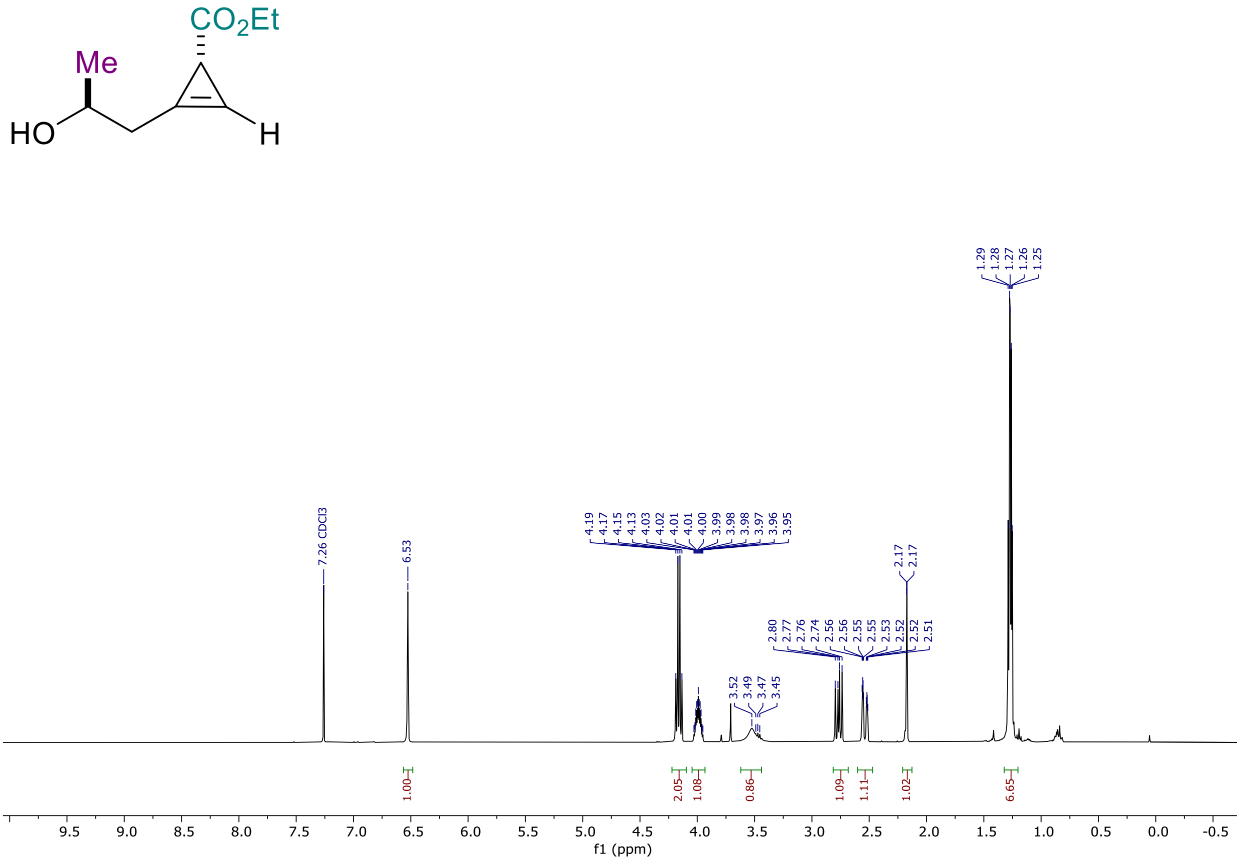
CO2Et
Me
HO
H
7.26 CDCl3
6.53
4.19 4.17 4.15 4.13 4.03 4.02 4.01 4.01 4.00 3.99 3.98 3.98 3.97 3.96 3.95
3.52 3.49 3.47 3.45
2.80 2.77 2.76 2.74 2.56 2.56 2.55 2.55 2.53 2.52 2.52 2.51
2.17 2.17
1.29 1.28 1.27 1.26 1.25
1.00 2.05 1.08 0.86 1.09 1.11 1.02 6.65
f1 (ppm)

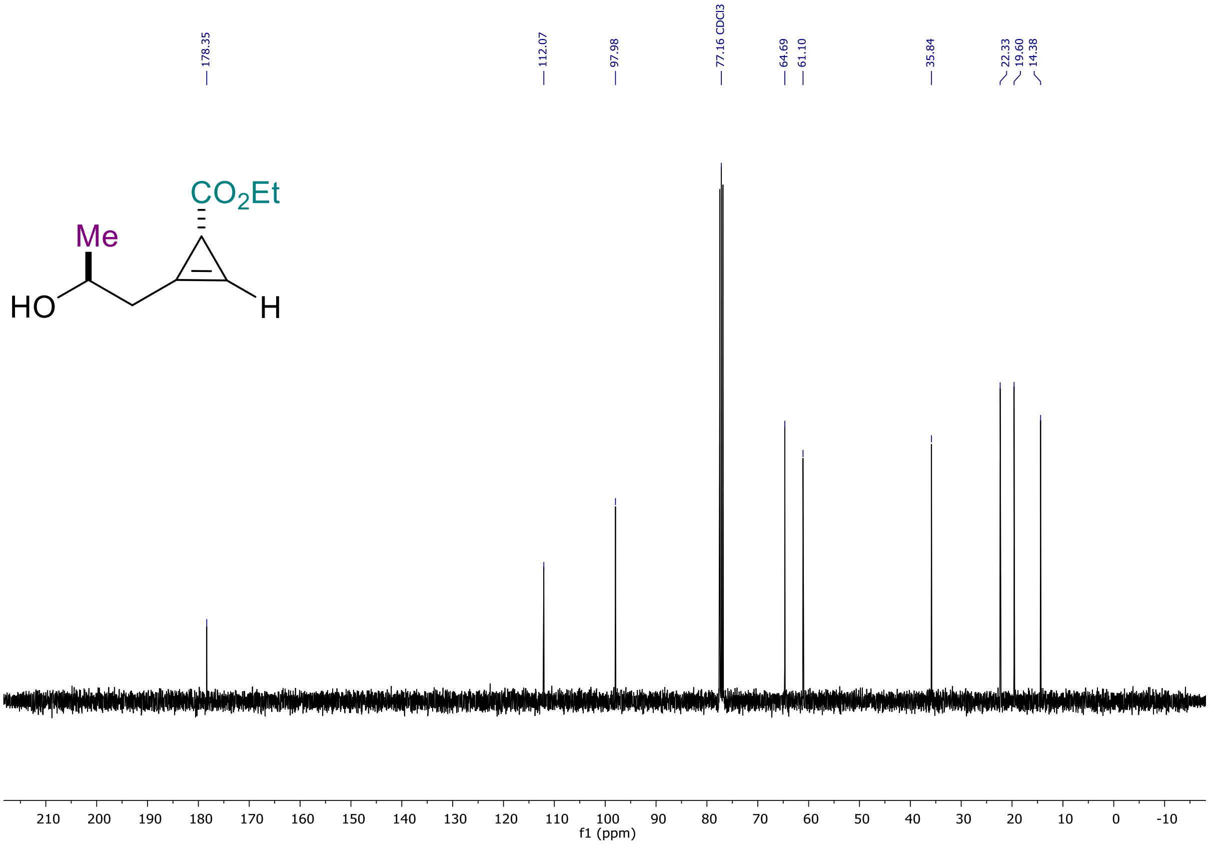
178.35
112.07
97.98
77.16 CDCl3
64.69
61.10
35.84
22.33 19.60 14.38
CO2Et
Me
HO
H
f1 (ppm)

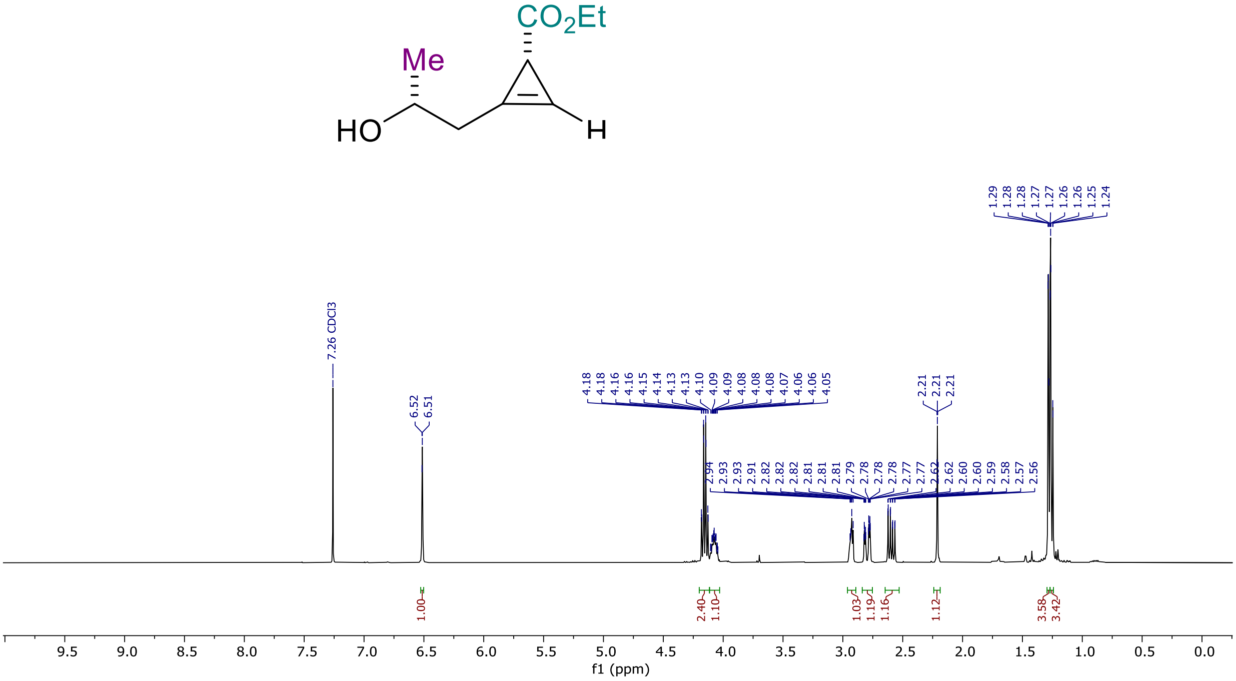
CO2Et
Me
HO
H
7.26 CDCl3
6.52
6.51
4.18 4.18 4.16 4.16 4.15 4.14 4.13 4.13 4.10 4.09 4.09 4.08 4.08 4.08 4.07 4.06 4.06 4.05
2.94 2.93 2.93 2.91 2.82 2.82 2.81 2.81 2.81 2.79 2.78 2.78 2.78 2.77 2.77 2.62 2.62 2.60 2.60 2.59 2.58 2.57 2.56
2.21 2.21 2.21
1.29 1.28 1.28 1.27 1.27 1.26 1.26 1.25 1.24
1.00
2.40
1.10
1.03
1.19
1.16
1.12
3.58
3.42
9.5 9.0 8.5 8.0 7.5 7.0 6.5 6.0 5.5 5.0 4.5 4.0 3.5 3.0 2.5 2.0 1.5 1.0 0.5 0.0
f1 (ppm)

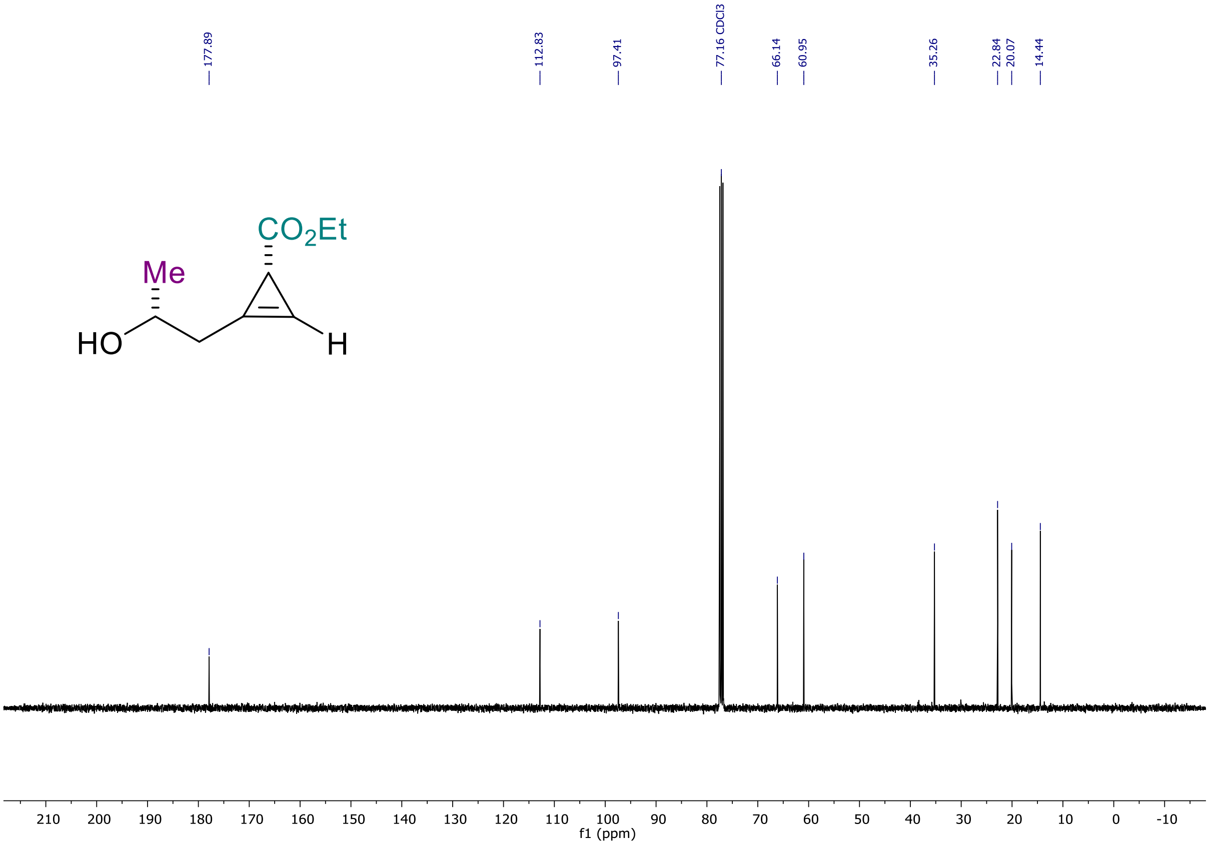
177.89
112.83
97.41
77.16 CDCl3
66.14
60.95
35.26
22.84
20.07
14.44
CO2Et
Me
HO
H
210 200 190 180 170 160 150 140 130 120 110 100 90 80 70 60 50 40 30 20 10 0 -10
f1 (ppm)

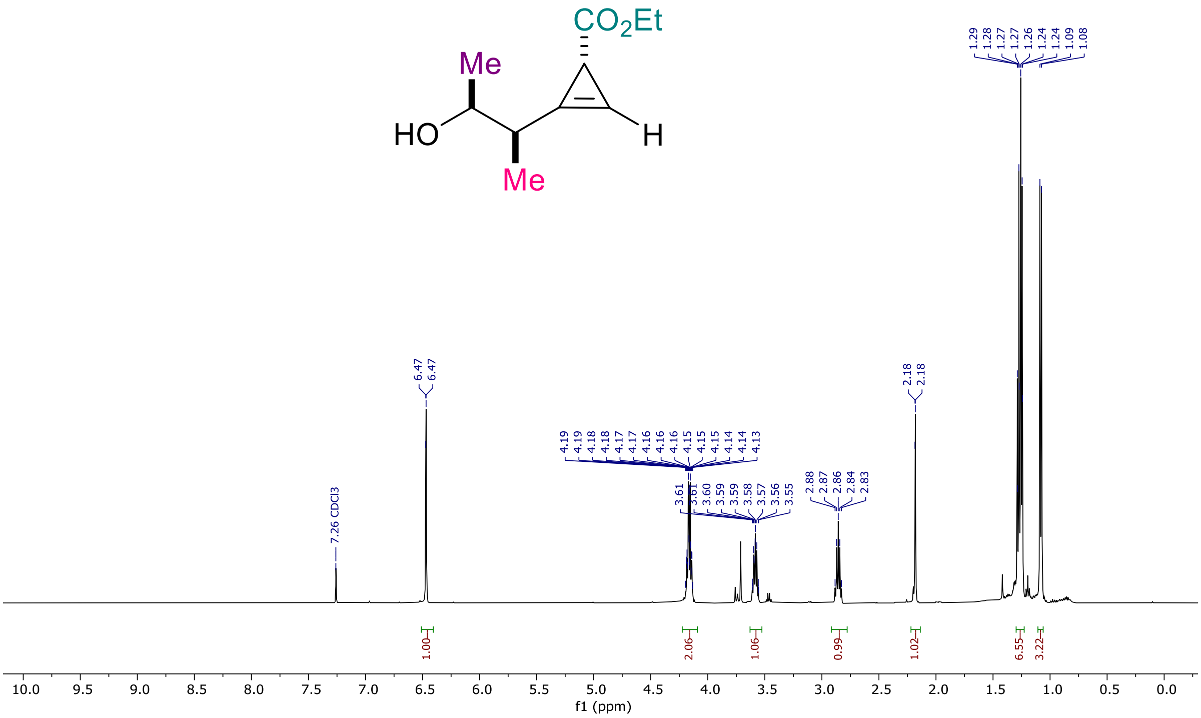
CO2Et
Me
HO
H
Me
7.26 CDCl3
6.47
6.47
2.18
2.18
1.00
2.06
1.06
0.99
1.02
6.55
3.22
f1 (ppm)

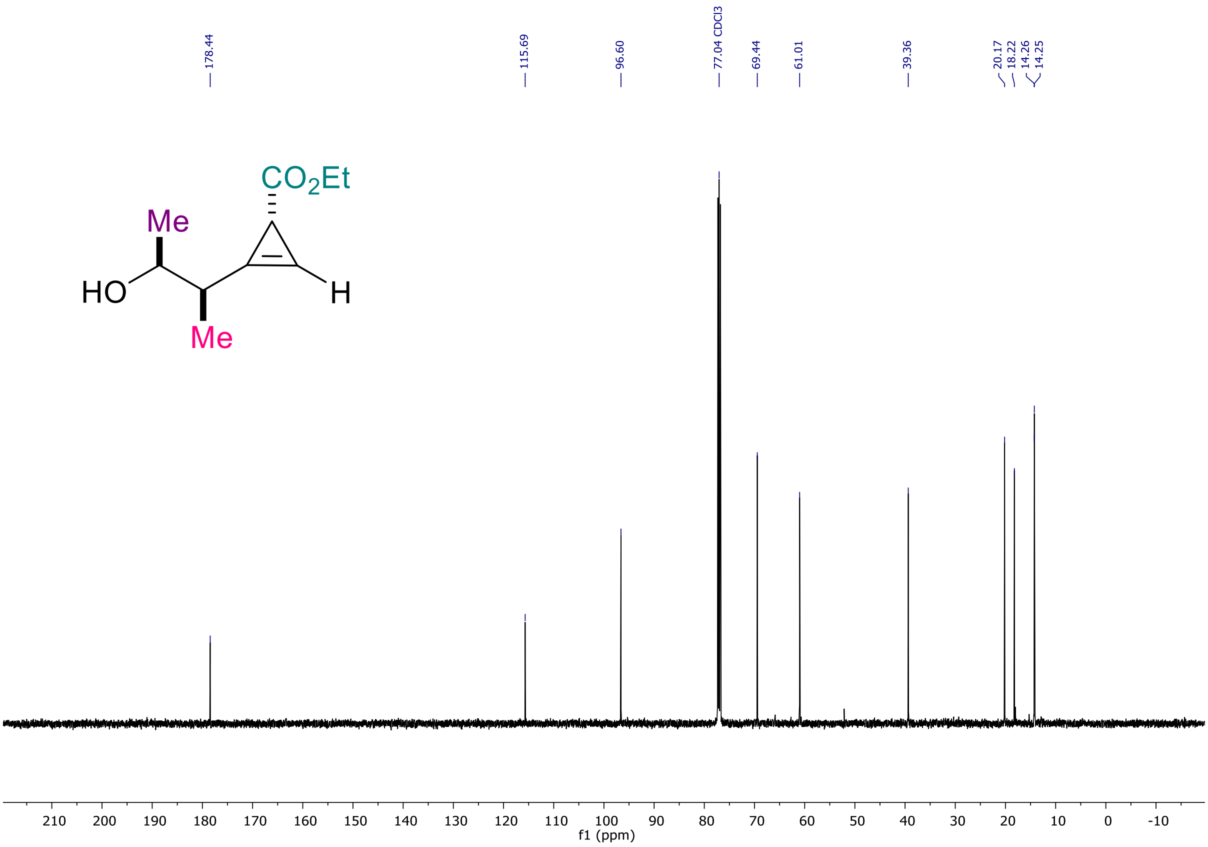
178.44
115.69
96.60
77.04 CDCl3
69.44
61.01
39.36
20.17
18.22
14.26
14.25
CO2Et
Me
HO
H
Me
f1 (ppm)

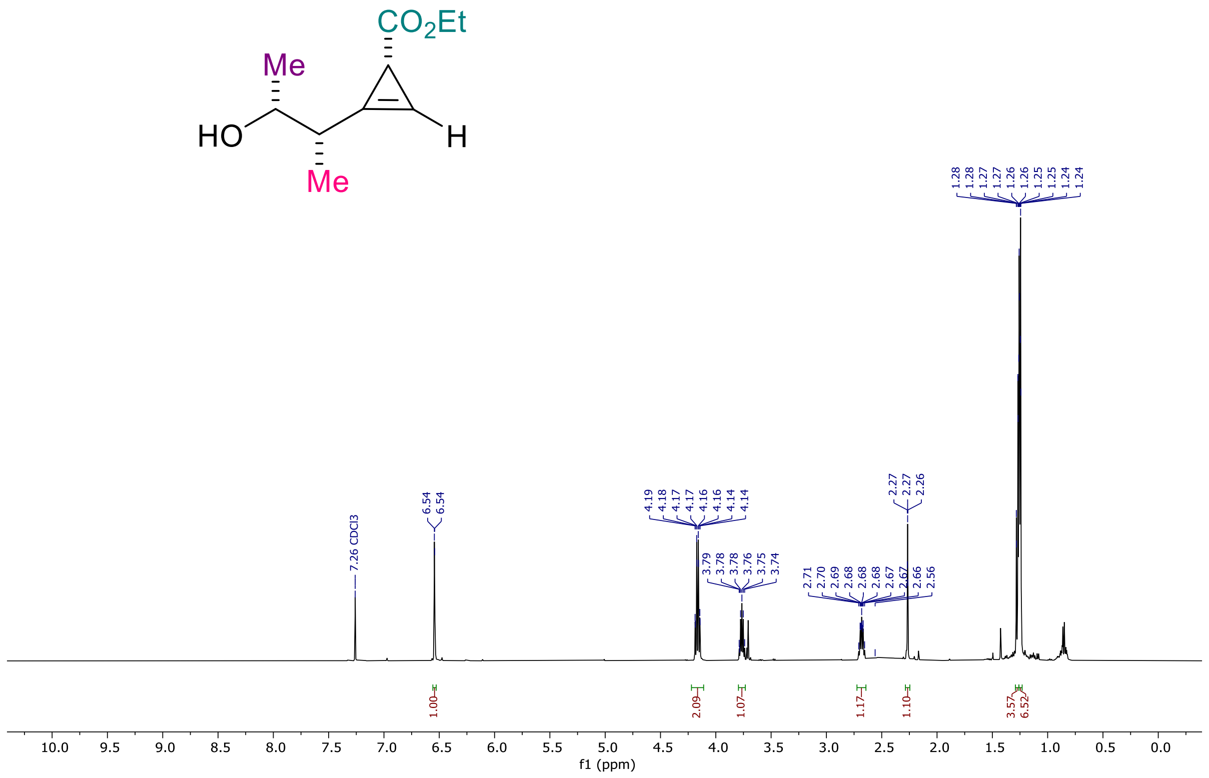
CO2Et
Me
HO
H
Me
7.26 CDCl3
6.54
6.54
4.19
4.18
4.17
4.17
4.16
4.16
4.14
4.14
3.79
3.78
3.78
3.76
3.75
3.74
2.71
2.70
2.69
2.68
2.68
2.68
2.67
2.67
2.66
2.56
2.27
2.27
2.26
1.28
1.28
1.27
1.27
1.26
1.26
1.25
1.25
1.24
1.24
1.00
2.09
1.07
1.17
1.10
3.57
6.52
10.0 9.5 9.0 8.5 8.0 7.5 7.0 6.5 6.0 5.5 5.0 4.5 4.0 3.5 3.0 2.5 2.0 1.5 1.0 0.5 0.0
f1 (ppm)

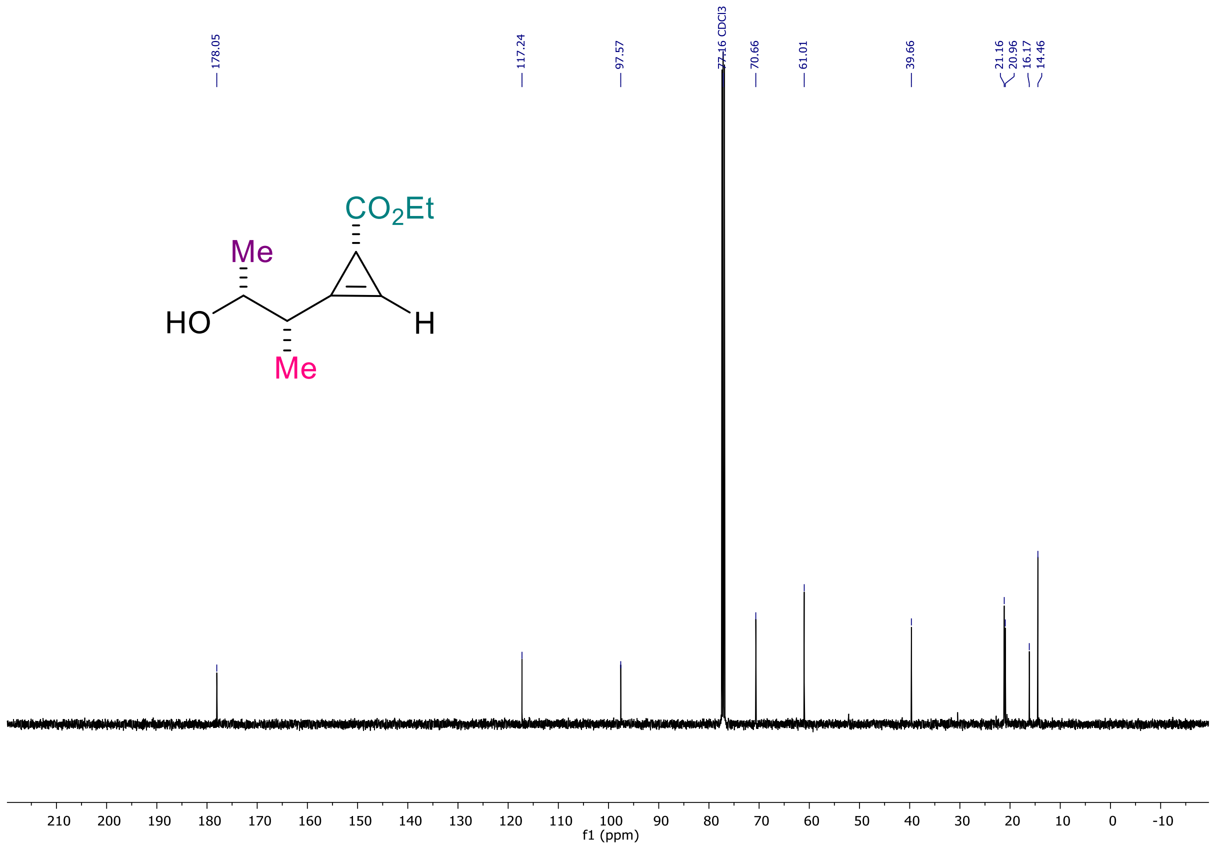
178.05
117.24
97.57
77.16 CDCl3
70.66
61.01
39.66
21.16
20.96
16.17
14.46
CO2Et
Me
HO
H
Me
210 200 190 180 170 160 150 140 130 120 110 100 90 80 70 60 50 40 30 20 10 0 -10
f1 (ppm)

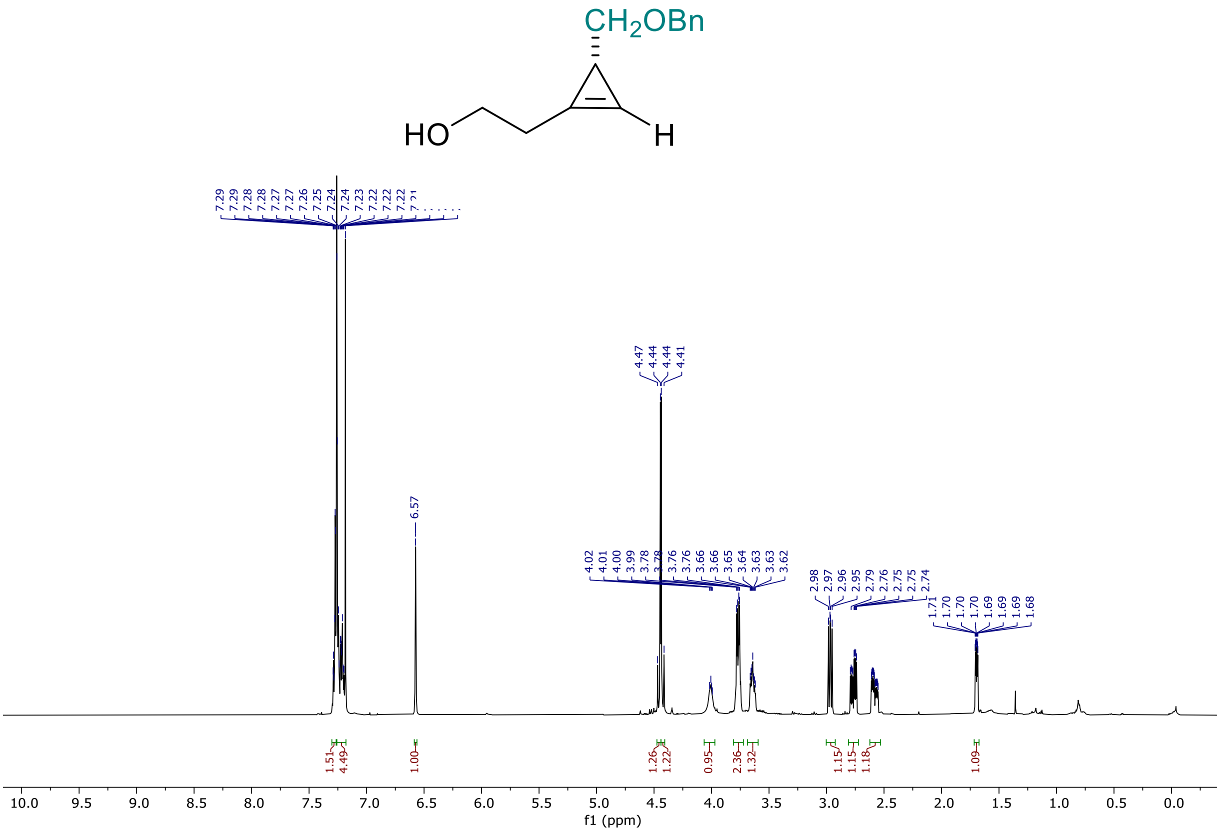

CH2OBn
HO
H
6.57
4.47
4.44
4.44
4.41
1.51
4.49
1.00
1.26
1.22
0.95
2.36
1.32
1.15
1.15
1.18
1.09
f1 (ppm)

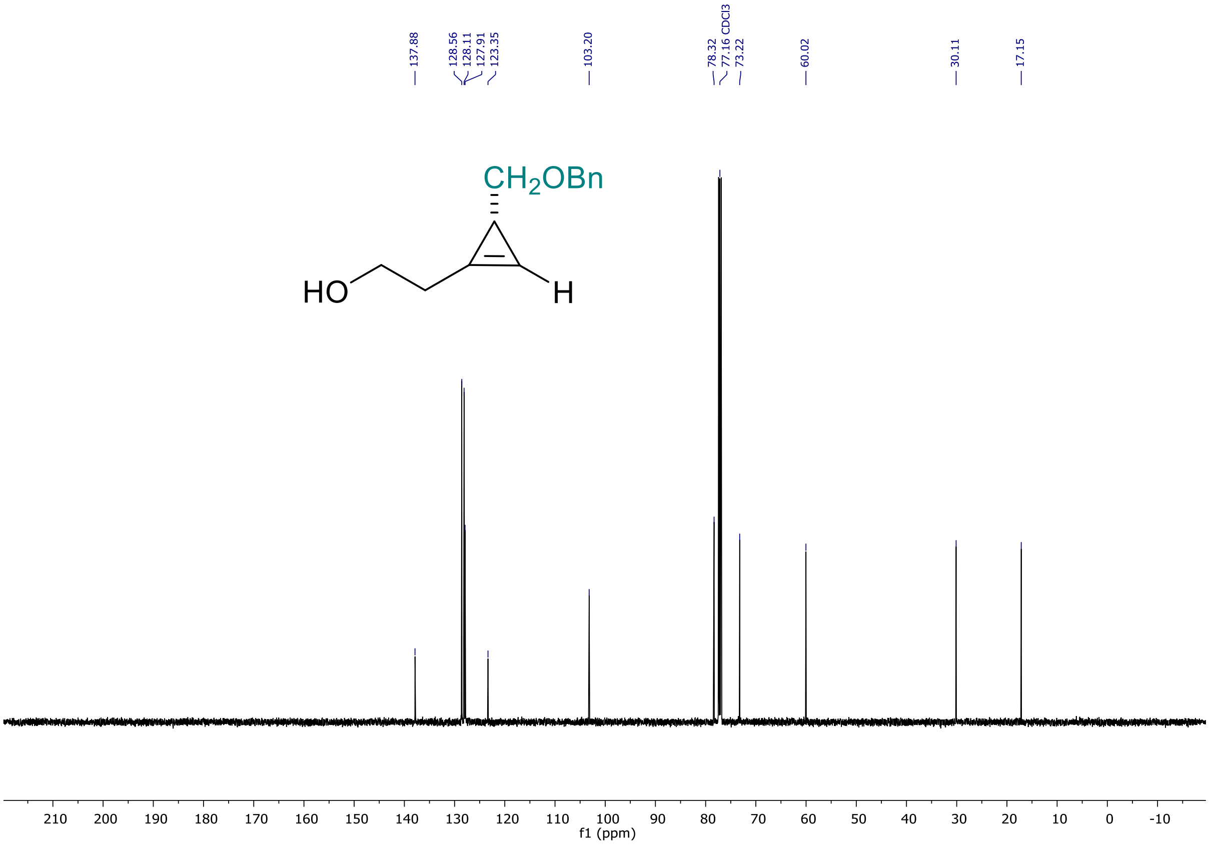

137.88
128.56
128.11
127.91
123.35
103.20
78.32
77.16 CDCl3
73.22
60.02
30.11
17.15
CH2OBn
HO
H
f1 (ppm)

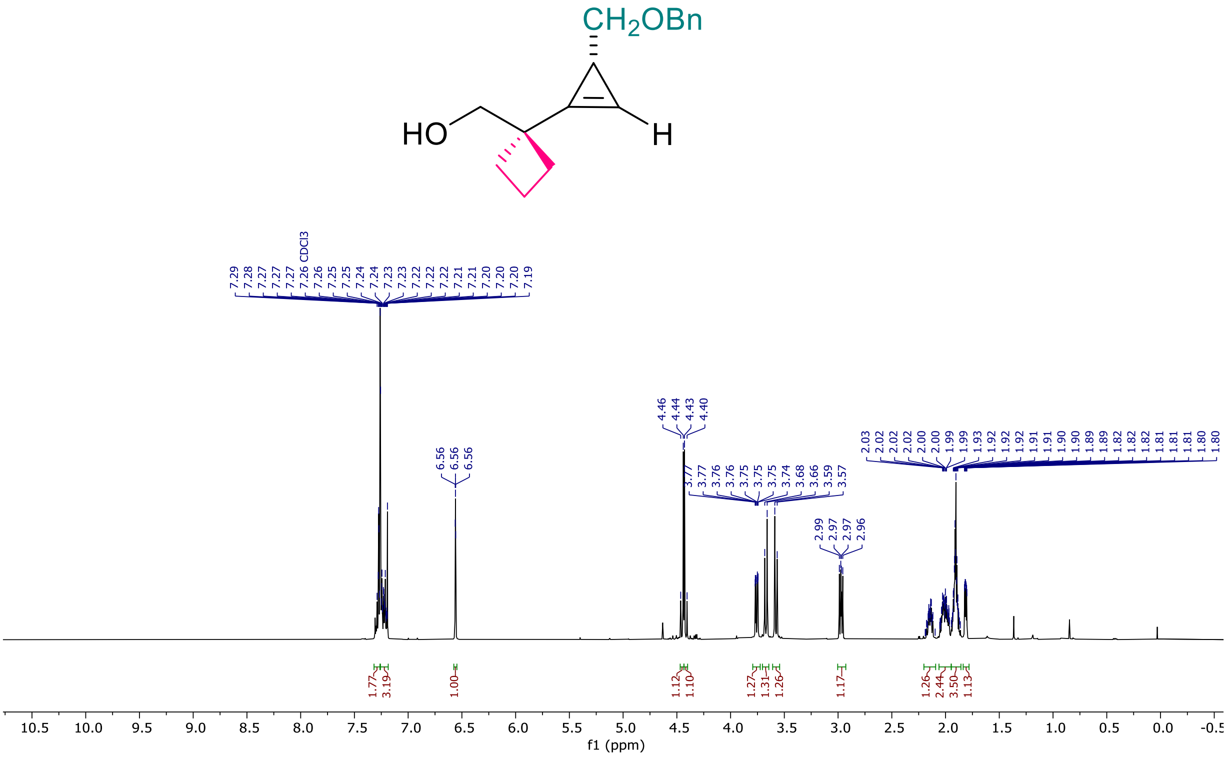
CH2OBn
HO
H
7.29 7.28 7.27 7.27 7.27 7.26 CDCl3 7.26 7.25 7.25 7.24 7.24 7.23 7.23 7.22 7.22 7.22 7.21 7.21 7.20 7.20 7.20 7.19
6.56 6.56 6.56
4.46 4.44 4.43 4.40
3.77 3.77 3.76 3.76 3.75 3.75 3.75 3.75 3.74 3.68 3.66 3.59 3.57
2.99 2.97 2.97 2.96
2.03 2.02 2.02 2.02 2.00 2.00 1.99 1.99 1.93 1.92 1.92 1.91 1.91 1.90 1.90 1.89 1.82 1.82 1.81 1.81 1.81 1.80
1.77 3.19 1.00 1.12 1.10 1.27 1.31 1.26 1.17 1.26 2.44 3.50 1.13
f1 (ppm)

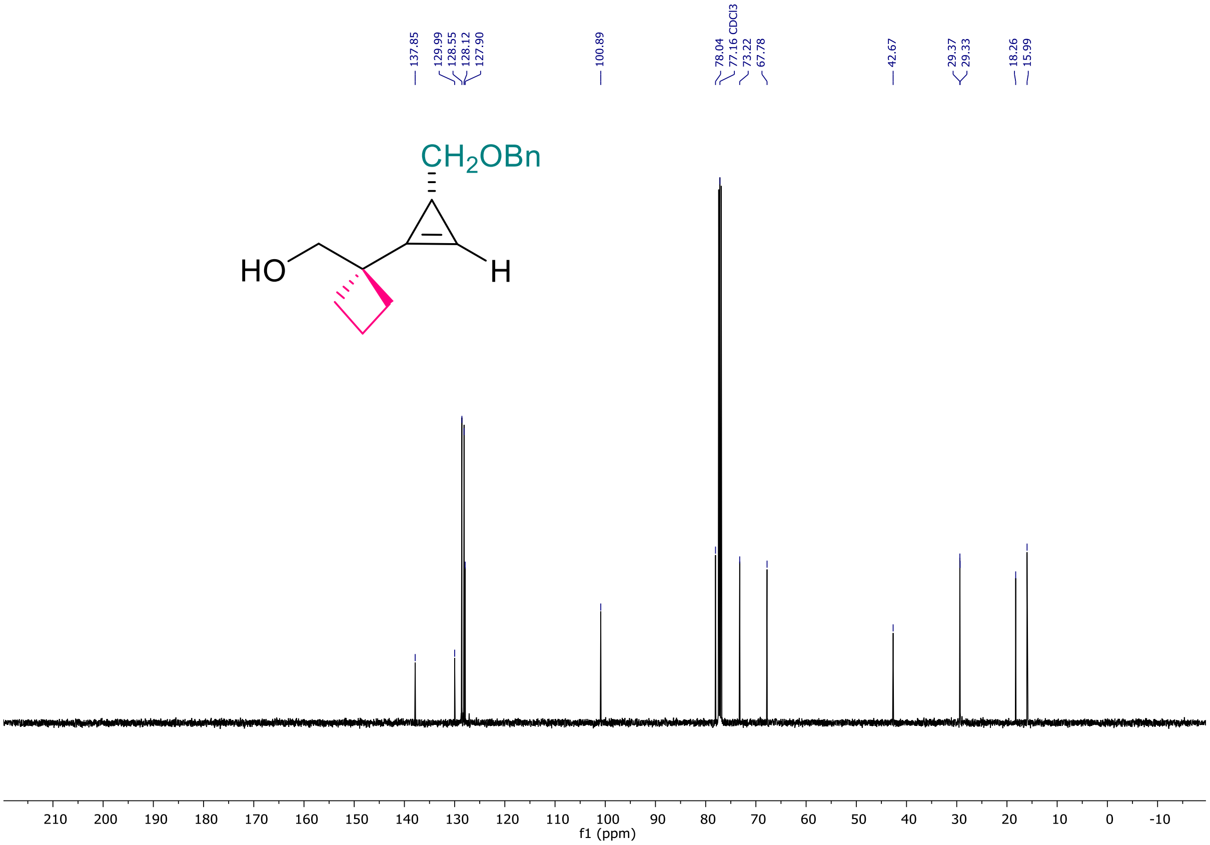
137.85
129.99 128.55 128.12 127.90
100.89
78.04 77.16 CDCl3 73.22 67.78
42.67
29.37 29.33
18.26 15.99
CH2OBn
HO
H
f1 (ppm)

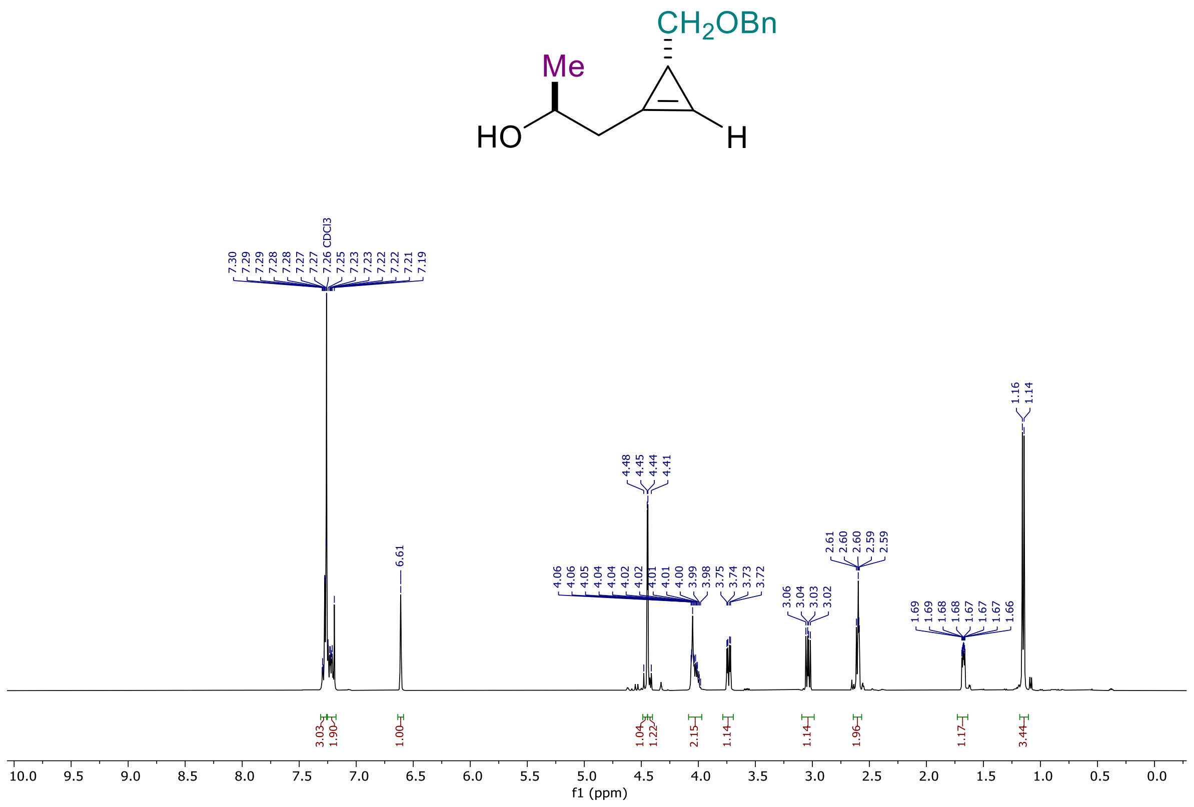
CH2OBn
Me
HO
H
f1 (ppm)

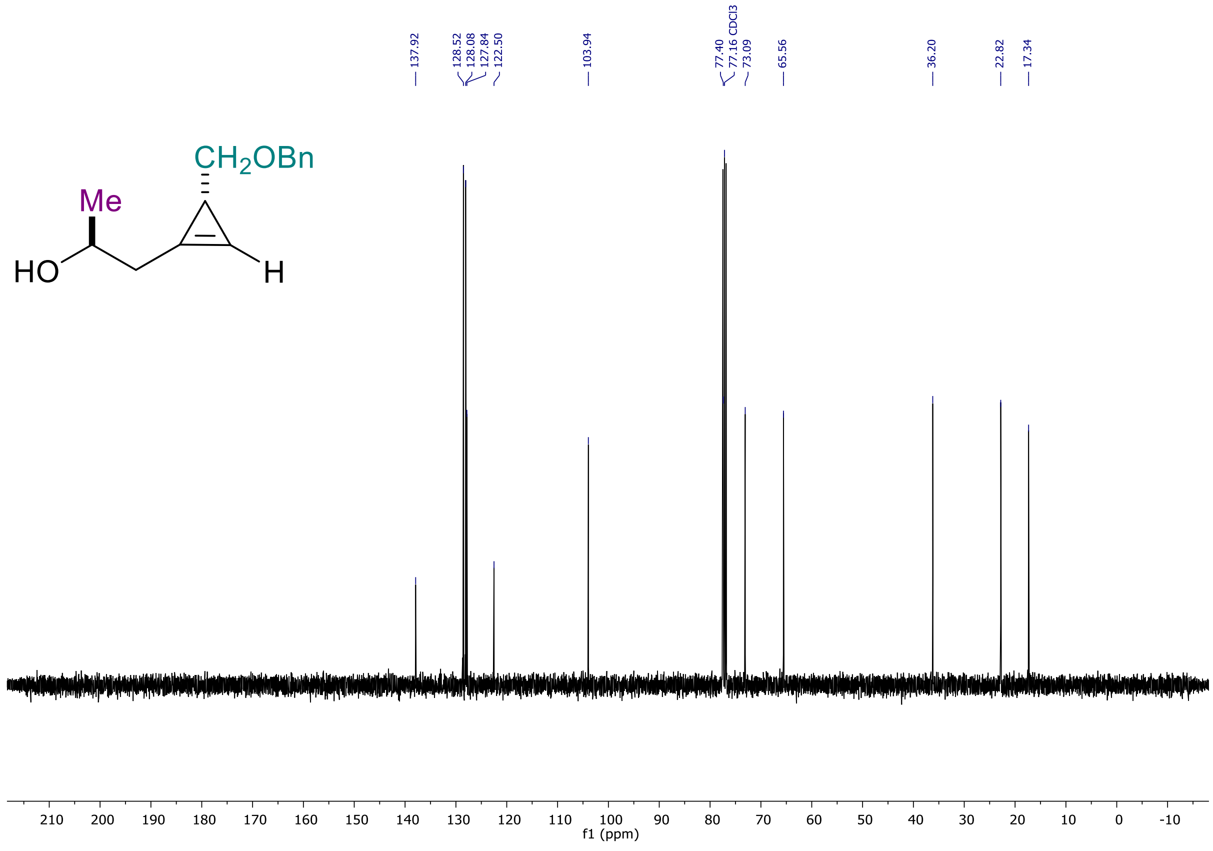
CH2OBn
Me
HO
H
f1 (ppm)

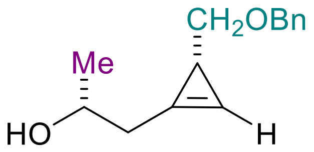
CH2OBn
Me
HO
H

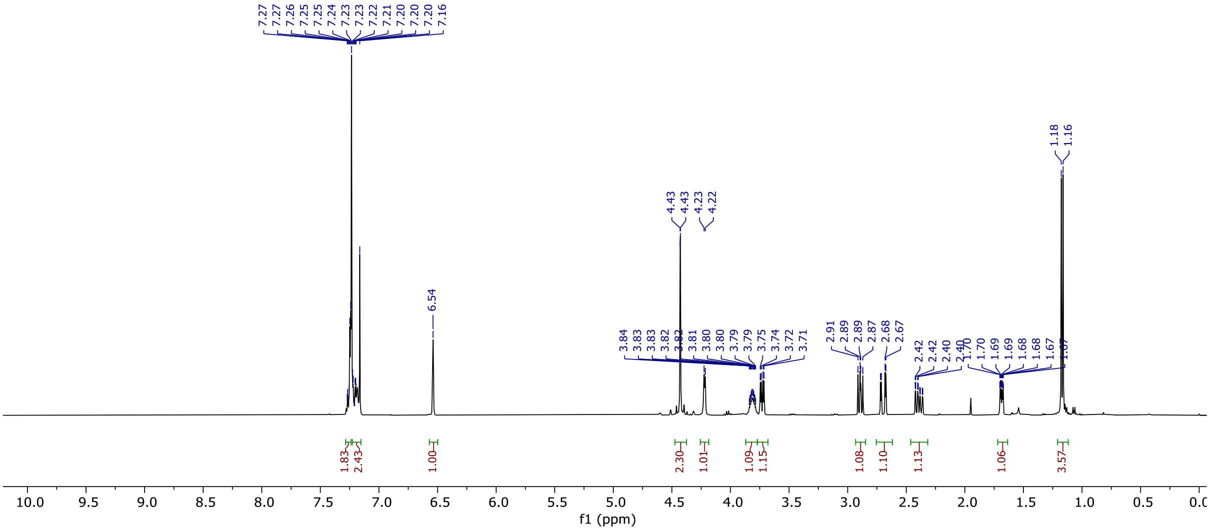
7.27 7.27 7.26 7.25 7.25 7.24 7.23 7.23 7.22 7.21 7.20 7.20 7.20 7.16
6.54
4.43 4.43 4.23 4.22
3.84 3.83 3.83 3.82 3.82 3.81 3.80 3.80 3.79 3.79 3.75 3.74 3.72 3.71
2.91 2.89 2.89 2.87 2.68 2.67
2.42 2.42 2.40 2.40 1.70 1.70 1.69 1.69 1.68 1.68 1.67 1.67
1.18 1.16
1.83 2.43 1.00 2.30 1.01 1.09 1.15 1.08 1.10 1.13 1.06 3.57
f1 (ppm)

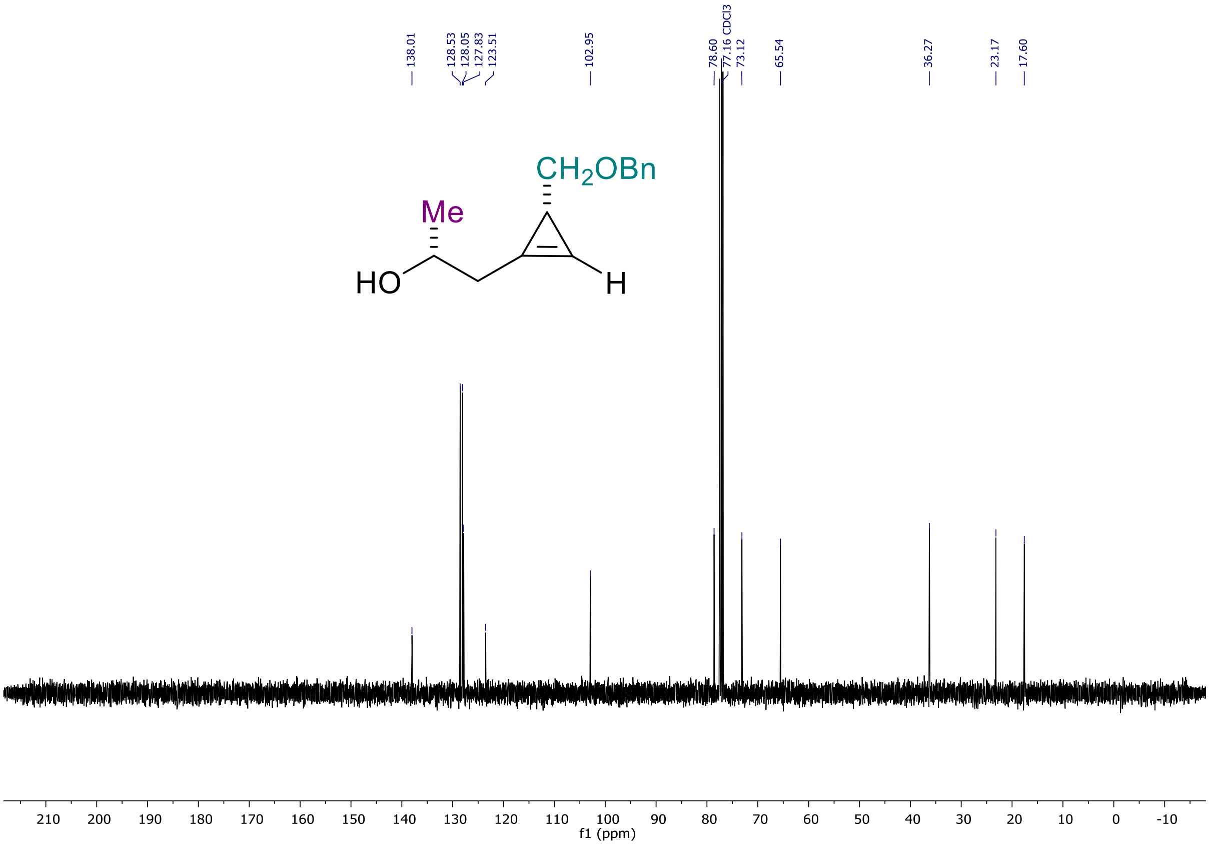
138.01
128.53 128.05 127.83 123.51
102.95
78.60 77.16 CDCl3 73.12
65.54
36.27
23.17 17.60
CH2OBn
Me
HO
H
f1 (ppm)

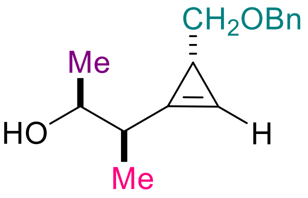
CH2OBn
Me
HO
H
Me

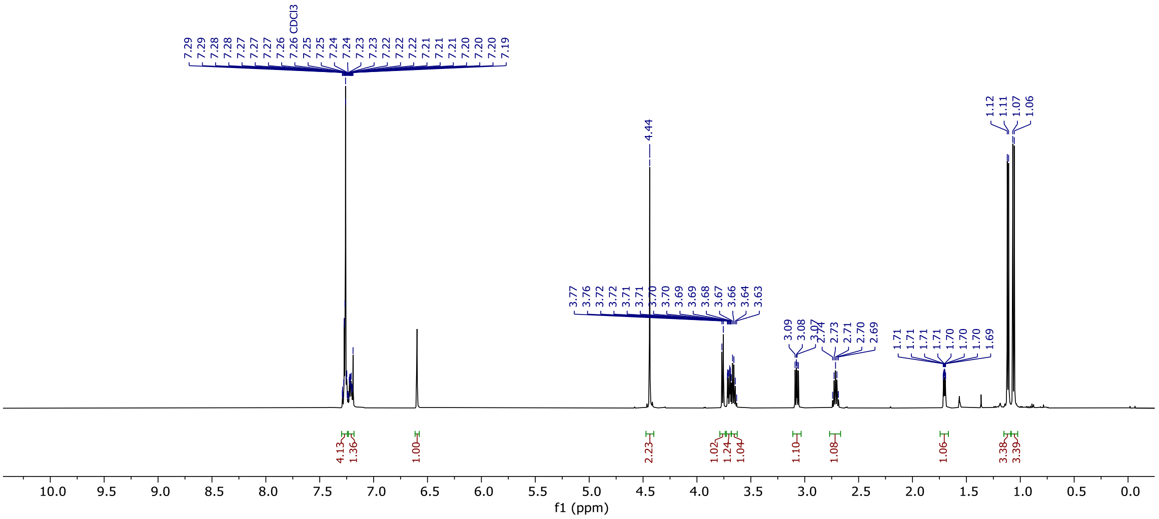
7.29 7.29 7.28 7.28 7.27 7.27 7.27 7.26 CDCl3 7.25 7.25 7.25 7.24 7.24 7.23 7.23 7.22 7.22 7.22 7.21 7.21 7.21 7.20 7.20 7.20 7.19
4.44
3.77 3.76 3.72 3.72 3.71 3.71 3.70 3.70 3.69 3.69 3.68 3.67 3.66 3.64 3.63
3.09 3.08 3.07 2.74 2.73 2.71 2.70 2.69
1.71 1.71 1.71 1.71 1.70 1.70 1.70 1.69
1.12 1.11 1.07 1.06
4.13 1.36 1.00 2.23 1.02 1.24 1.04 1.10 1.08 1.06 3.38 3.39
10.0 9.5 9.0 8.5 8.0 7.5 7.0 6.5 6.0 5.5 5.0 4.5 4.0 3.5 3.0 2.5 2.0 1.5 1.0 0.5 0.0
f1 (ppm)

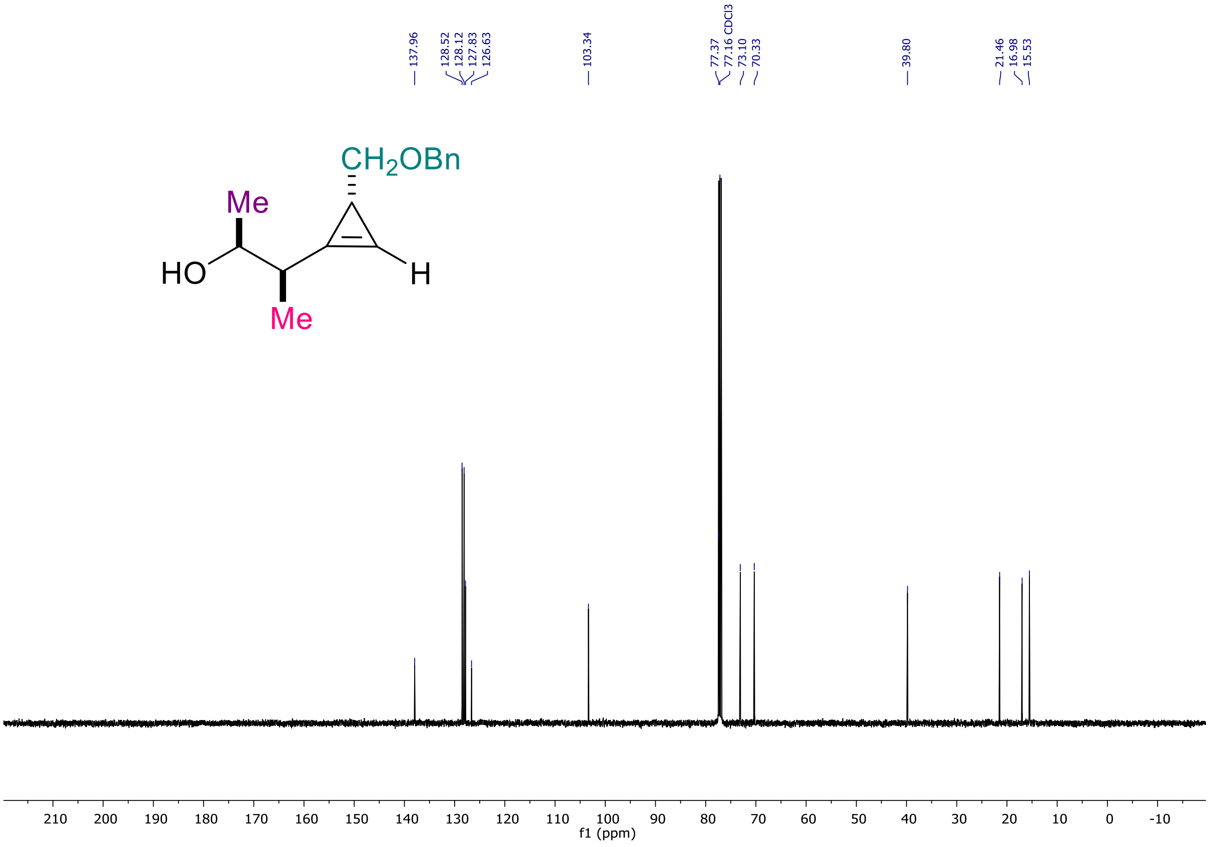
137.96
128.52 128.12 127.83 126.63
103.34
77.37 77.16 CDCl3 73.10 70.33
39.80
21.46 16.98 15.53
CH2OBn
Me
HO
H
Me
210 200 190 180 170 160 150 140 130 120 110 100 90 80 70 60 50 40 30 20 10 0 -10
f1 (ppm)

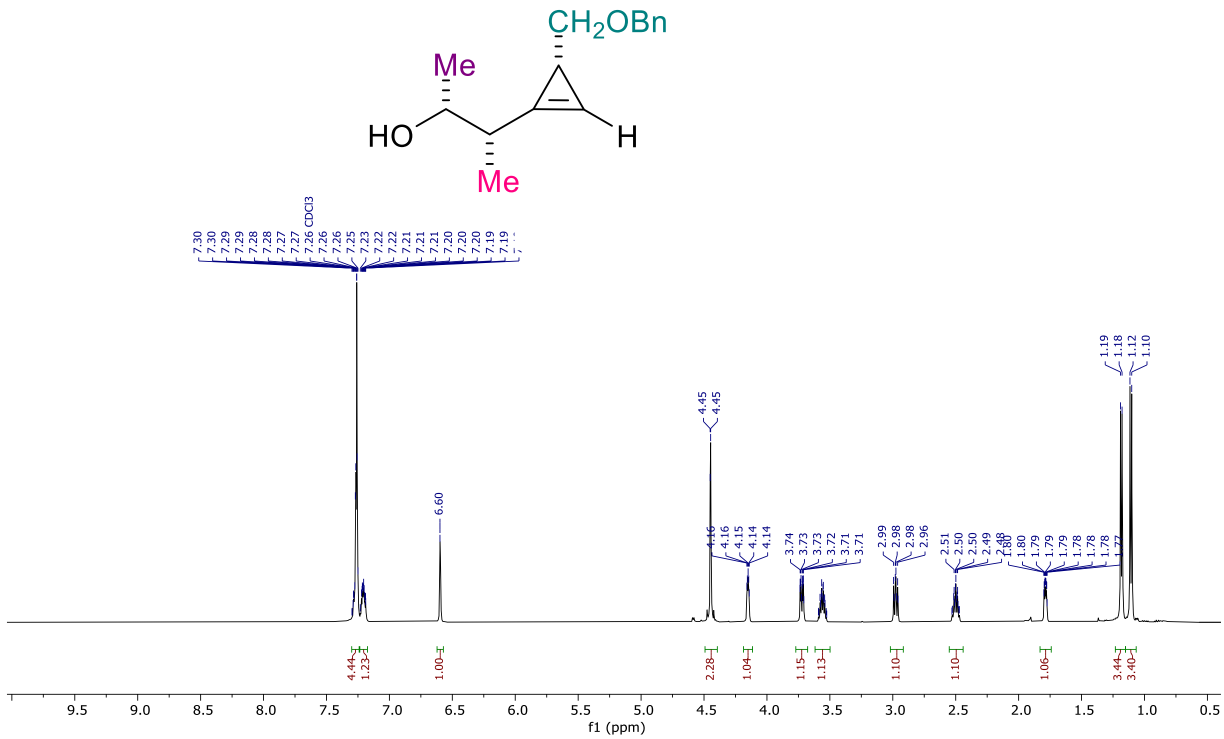
CH2OBn
Me
HO
H
Me
CDCl3
6.60
4.45
f1 (ppm)

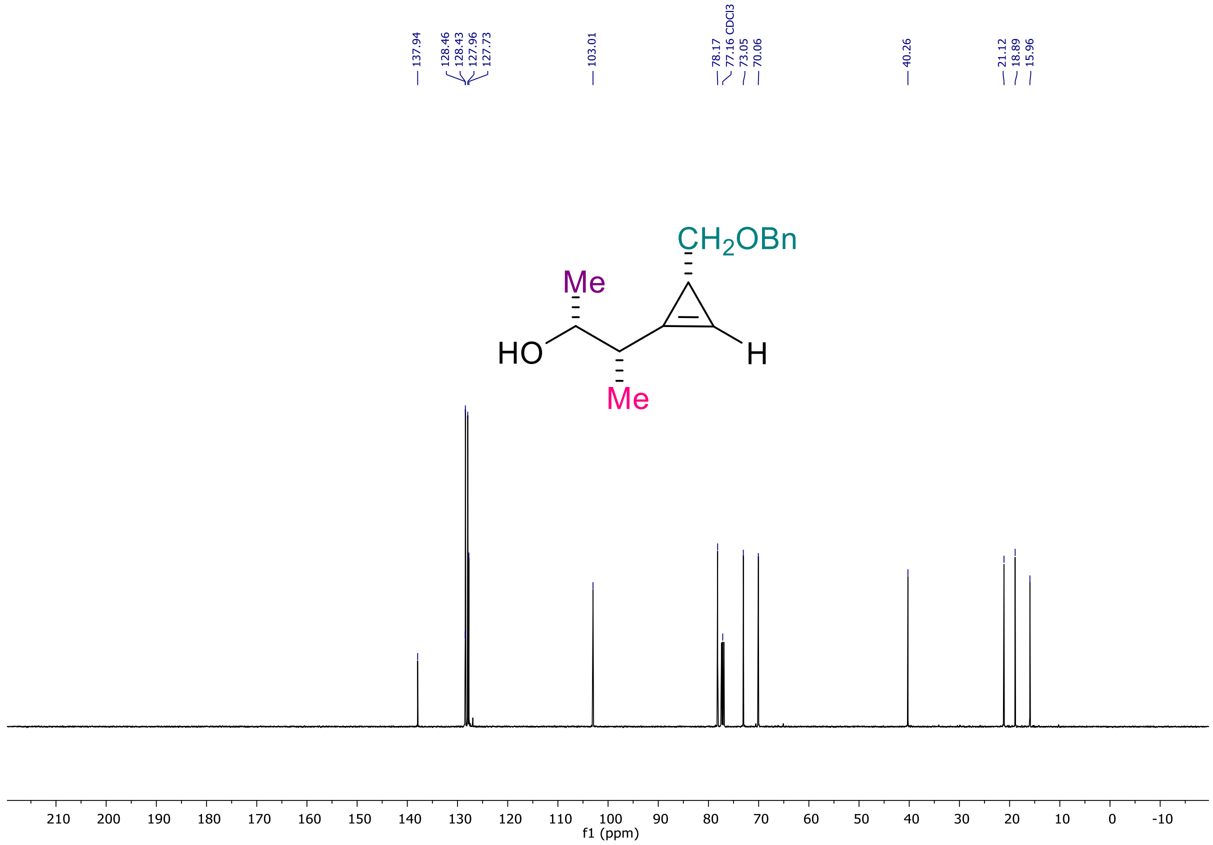
137.94
128.46
128.43
127.96
127.73
103.01
78.17
77.16 CDCl3
73.05
70.06
40.26
21.12
18.89
15.96
CH2OBn
Me
HO
H
Me
f1 (ppm)

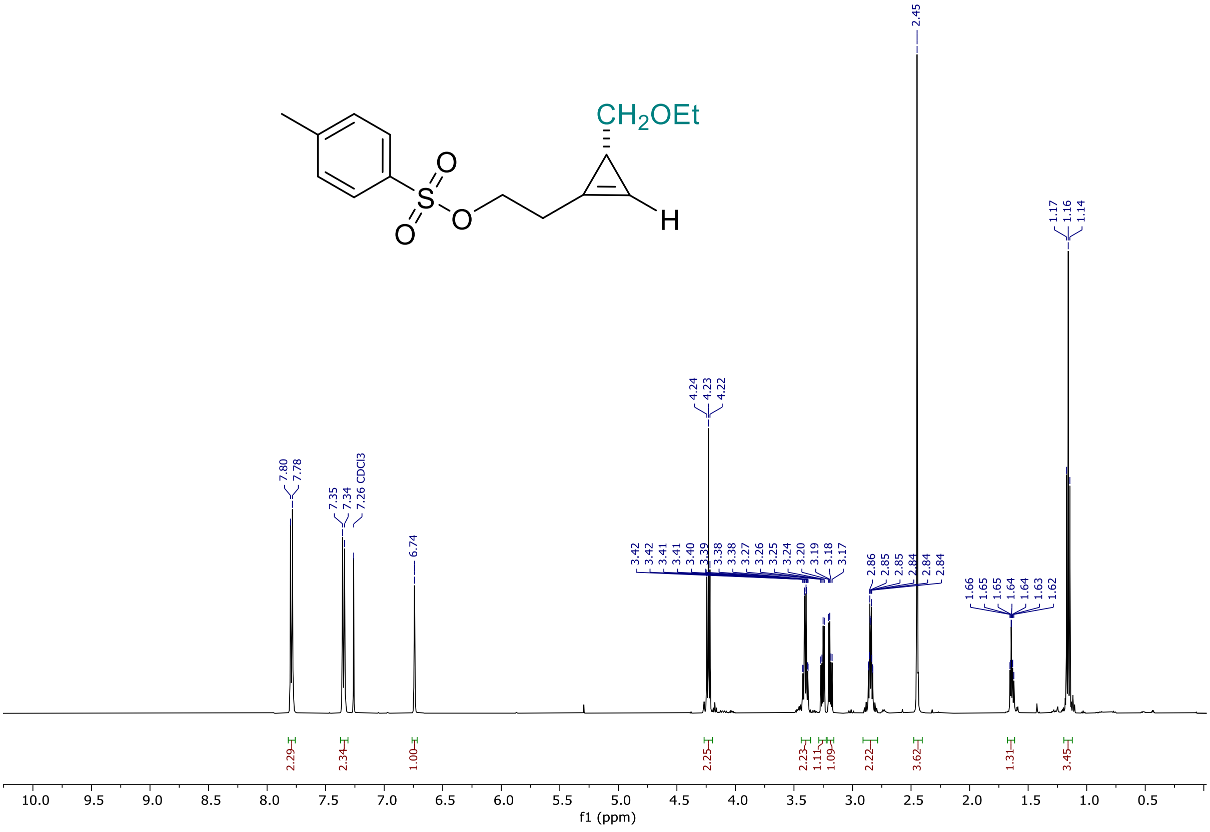
CH2OEt
H
O
S
O
O
7.80
7.78
7.35
7.34
7.26 CDCl3
6.74
4.24
4.23
4.22
3.42
3.42
3.41
3.41
3.40
3.39
3.38
3.38
3.27
3.26
3.25
3.24
3.20
3.19
3.18
3.17
2.86
2.85
2.85
2.84
2.84
2.84
2.45
1.66
1.65
1.65
1.64
1.64
1.63
1.62
1.17
1.16
1.14
2.29
2.34
1.00
2.25
2.23
1.11
1.09
2.22
3.62
1.31
3.45
10.0 9.5 9.0 8.5 8.0 7.5 7.0 6.5 6.0 5.5 5.0 4.5 4.0 3.5 3.0 2.5 2.0 1.5 1.0 0.5
f1 (ppm)
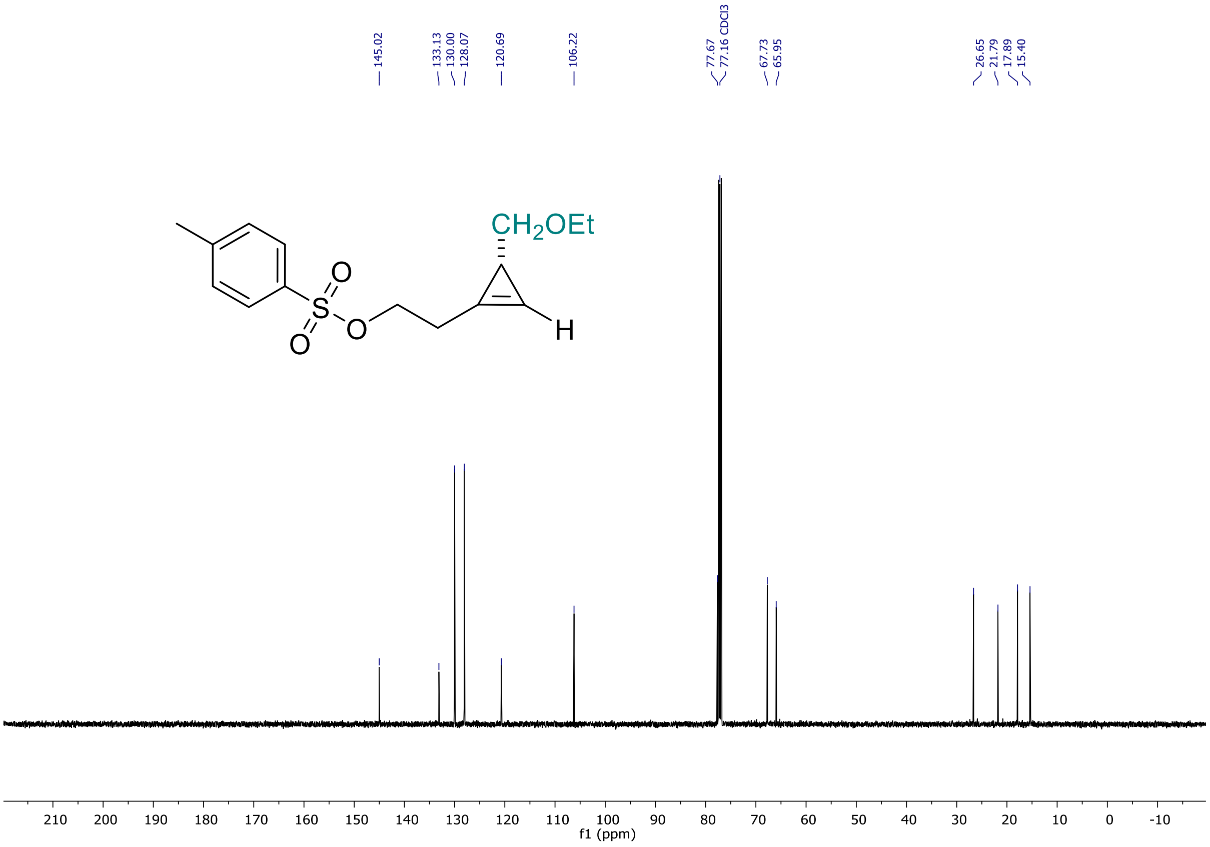
145.02
133.13
130.00
128.07
120.69
106.22
77.67
77.16 CDCl3
67.73
65.95
26.65
21.79
17.89
15.40
CH2OEt
H
O
S
O
O
210 200 190 180 170 160 150 140 130 120 110 100 90 80 70 60 50 40 30 20 10 0 -10
f1 (ppm)

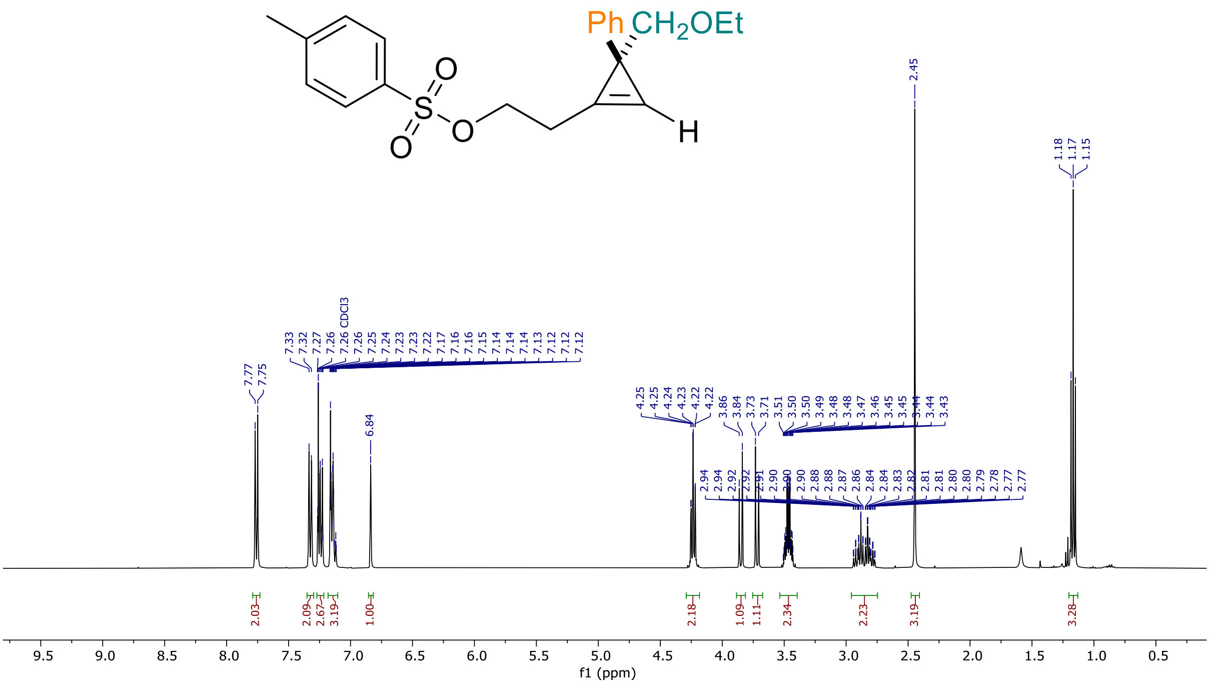
Ph CH2OEt
H
f1 (ppm)

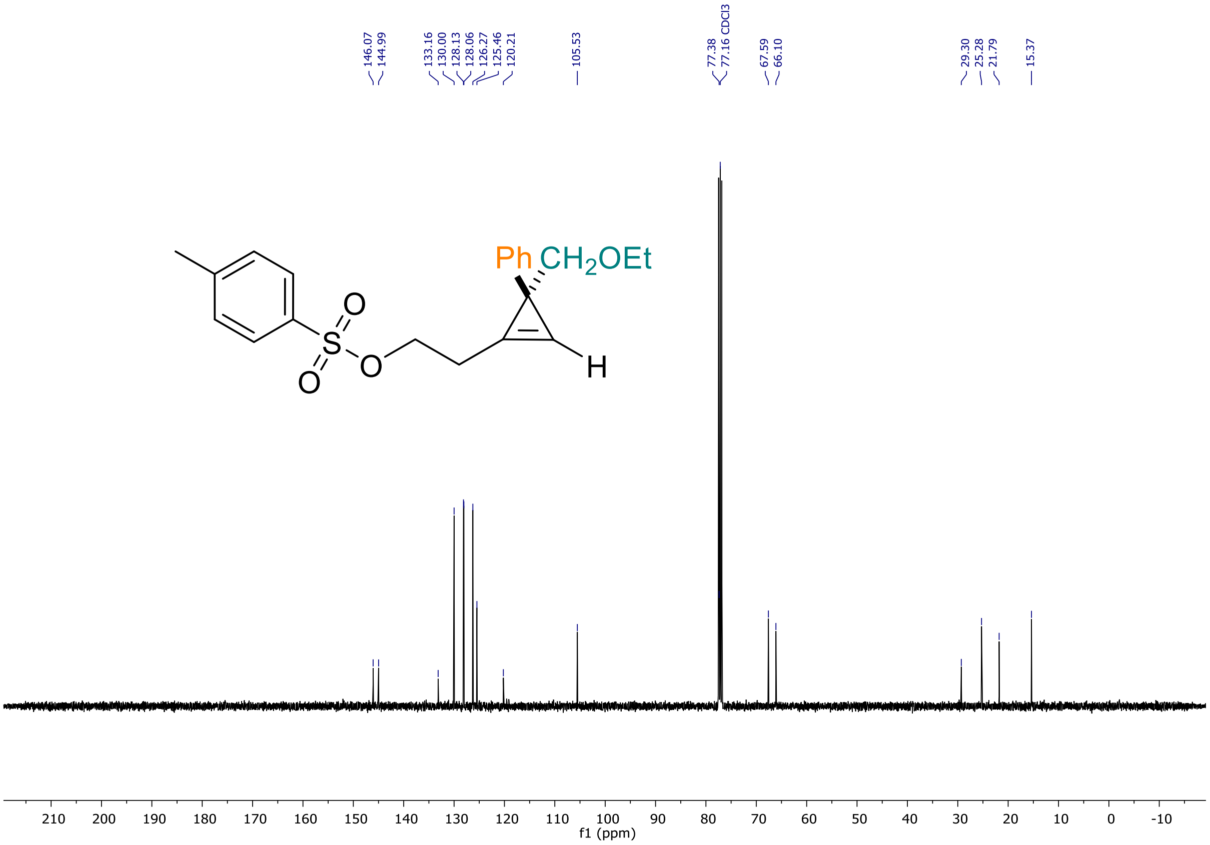
Ph CH2OEt
H
f1 (ppm)

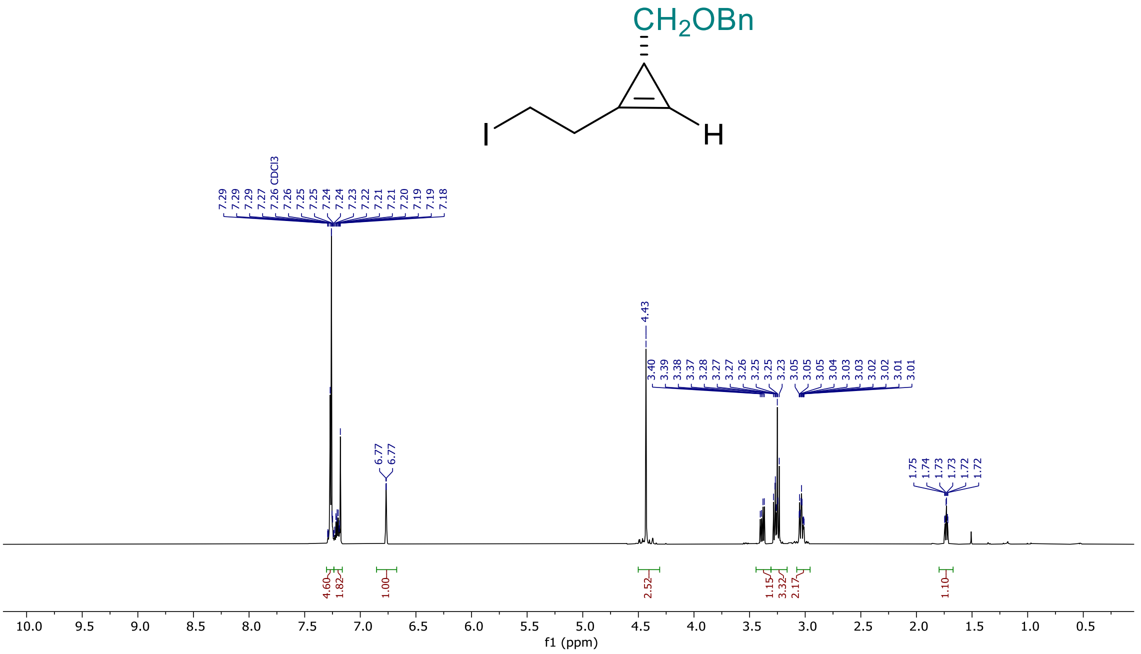

CH2OBn
I
H
7.29
7.29
7.29
7.27
7.26 CDCl3
7.26
7.25
7.25
7.24
7.24
7.23
7.22
7.21
7.21
7.20
7.19
7.19
7.18
6.77
6.77
4.43
3.40
3.39
3.38
3.37
3.28
3.27
3.27
3.26
3.25
3.25
3.23
3.05
3.05
3.04
3.03
3.03
3.02
3.02
3.01
3.01
1.75
1.74
1.73
1.73
1.72
1.72
4.60
1.82
1.00
2.52
1.15
3.32
2.17
1.10
10.0 9.5 9.0 8.5 8.0 7.5 7.0 6.5 6.0 5.5 5.0 4.5 4.0 3.5 3.0 2.5 2.0 1.5 1.0 0.5
f1 (ppm)

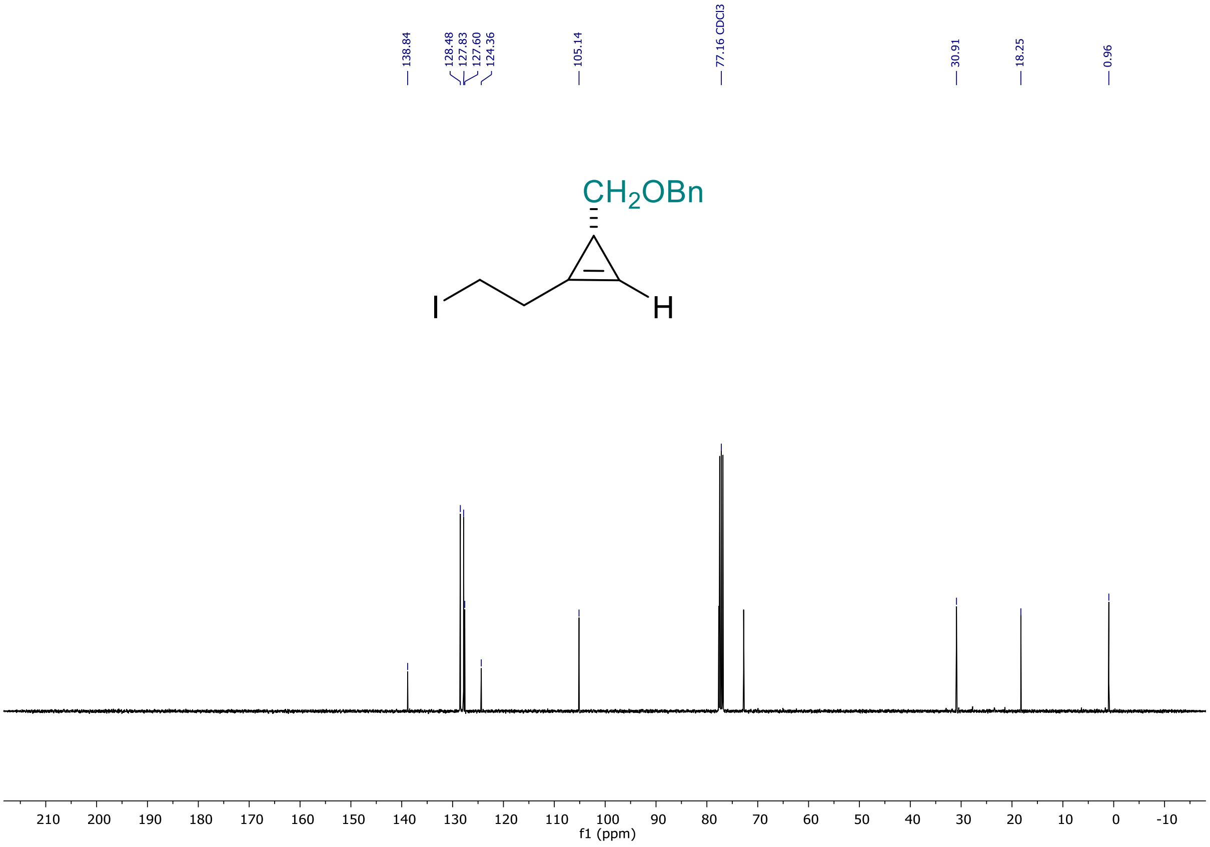

138.84
128.48
127.83
127.60
124.36
105.14
77.16 CDCl3
30.91
18.25
0.96
CH2OBn
I
H
210 200 190 180 170 160 150 140 130 120 110 100 90 80 70 60 50 40 30 20 10 0 -10
f1 (ppm)

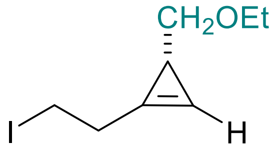
CH2OEt
I
H

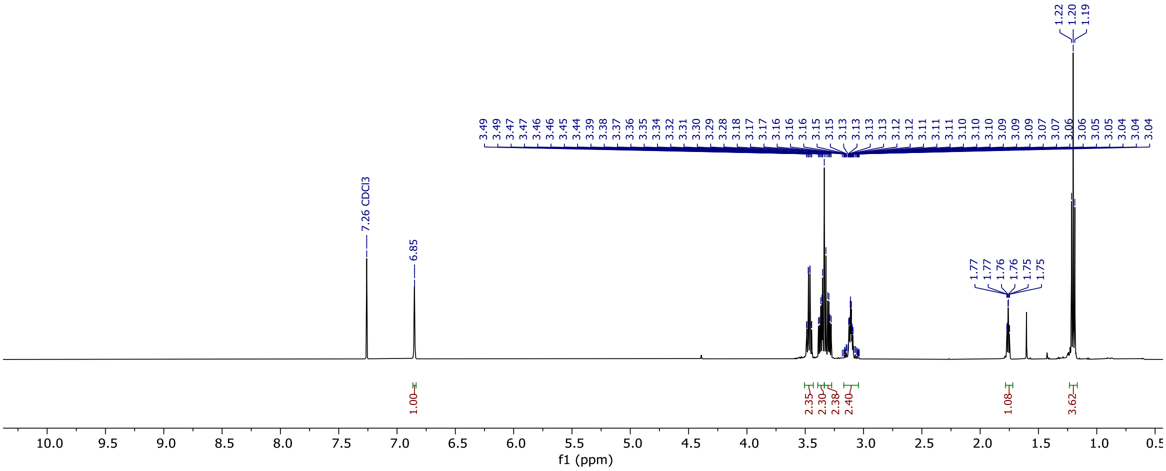
7.26 CDCl3
6.85
1.77
1.77
1.76
1.76
1.75
1.75
1.22
1.20
1.19
1.00
2.35
2.30
2.38
2.40
1.08
3.62
f1 (ppm)

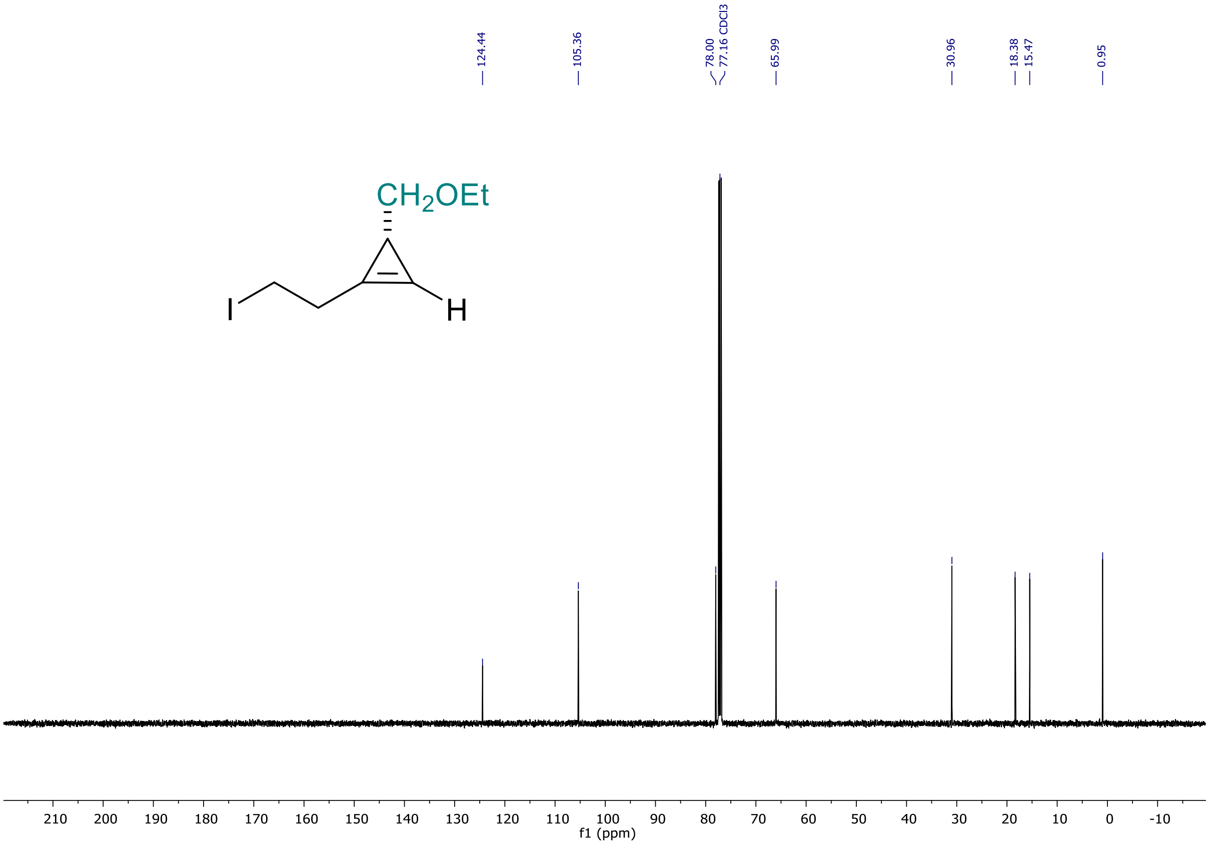
124.44
105.36
78.00
77.16 CDCl3
65.99
30.96
18.38
15.47
0.95
CH2OEt
I
H
f1 (ppm)

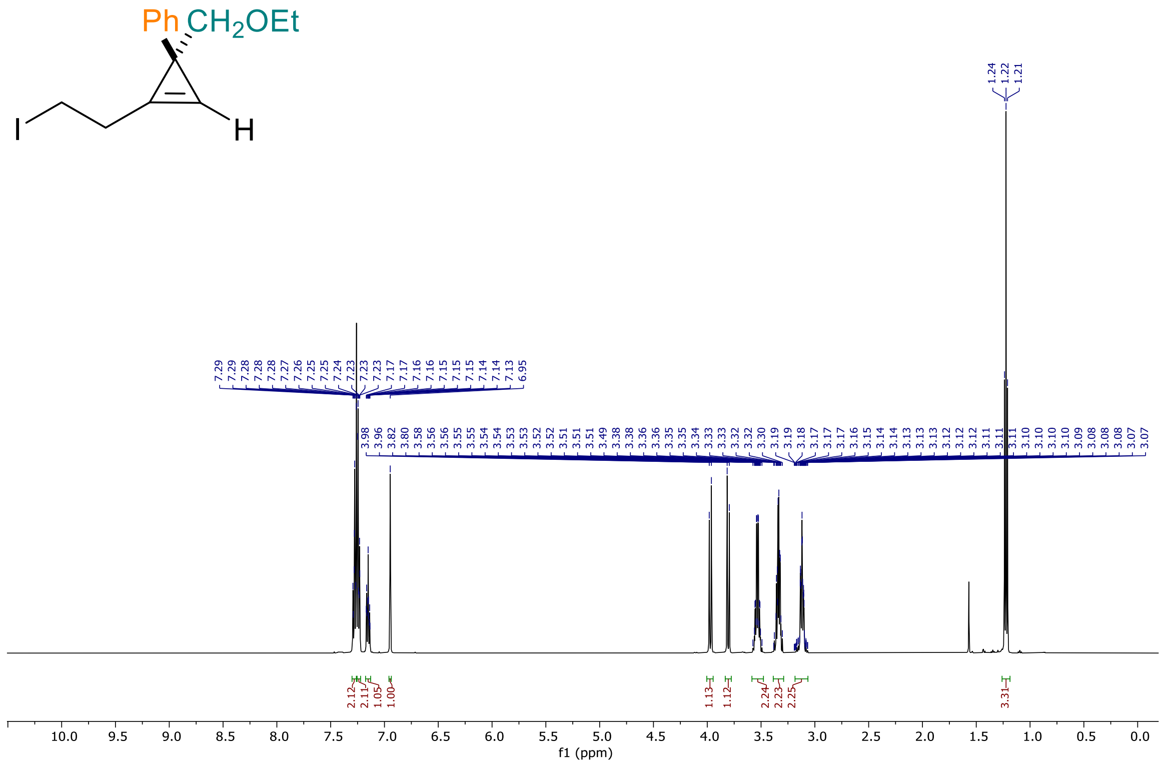
Ph
CH2OEt
I
H
2.12
2.11
1.05
1.00
1.13
1.12
2.24
2.23
2.25
3.31
f1 (ppm)

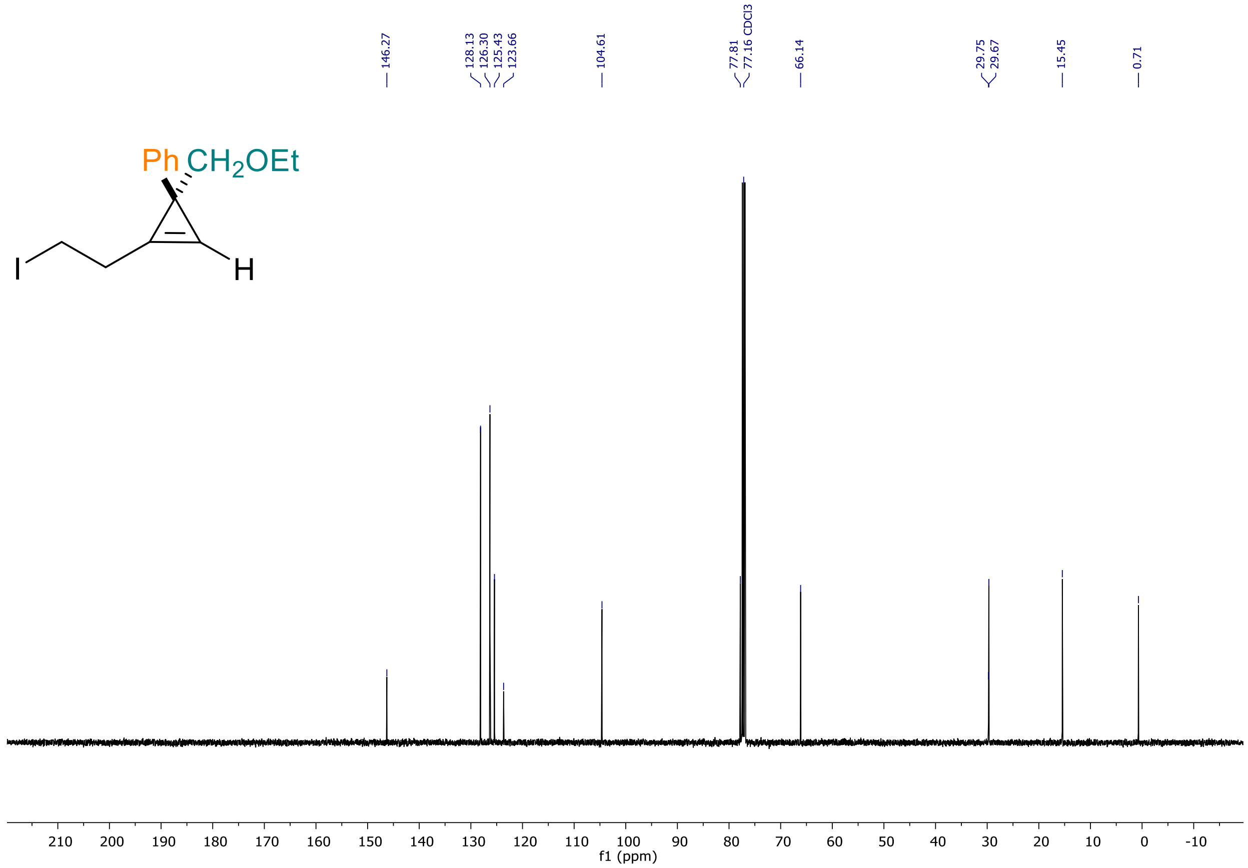
146.27
128.13
126.30
125.43
123.66
104.61
77.81
77.16 CDCl3
66.14
29.75
29.67
15.45
0.71
Ph
CH2OEt
I
H
f1 (ppm)

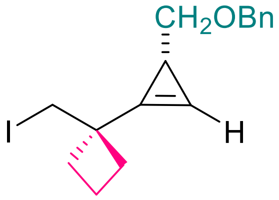
CH2OBn
I
H

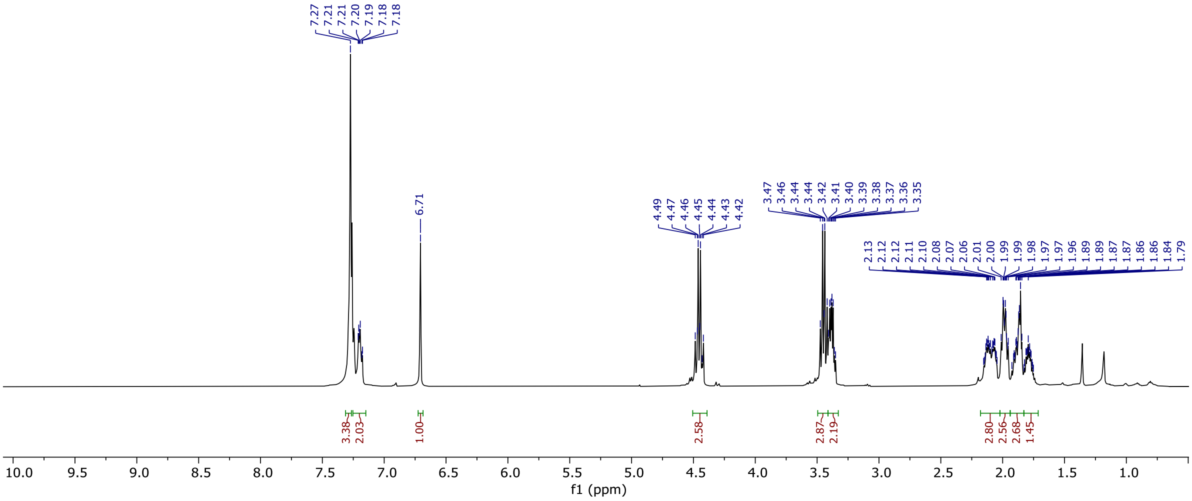
7.27
7.21
7.21
7.20
7.19
7.18
7.18
6.71
4.49
4.47
4.46
4.45
4.44
4.43
4.42
3.47
3.46
3.44
3.42
3.41
3.40
3.39
3.38
3.37
3.36
3.35
2.13
2.12
2.12
2.11
2.10
2.08
2.07
2.06
2.01
2.00
1.99
1.99
1.98
1.97
1.97
1.96
1.89
1.89
1.87
1.87
1.86
1.86
1.84
1.79
3.38
2.03
1.00
2.58
2.87
2.19
2.80
2.56
2.68
1.45
10.0 9.5 9.0 8.5 8.0 7.5 7.0 6.5 6.0 5.5 5.0 4.5 4.0 3.5 3.0 2.5 2.0 1.5 1.0
f1 (ppm)

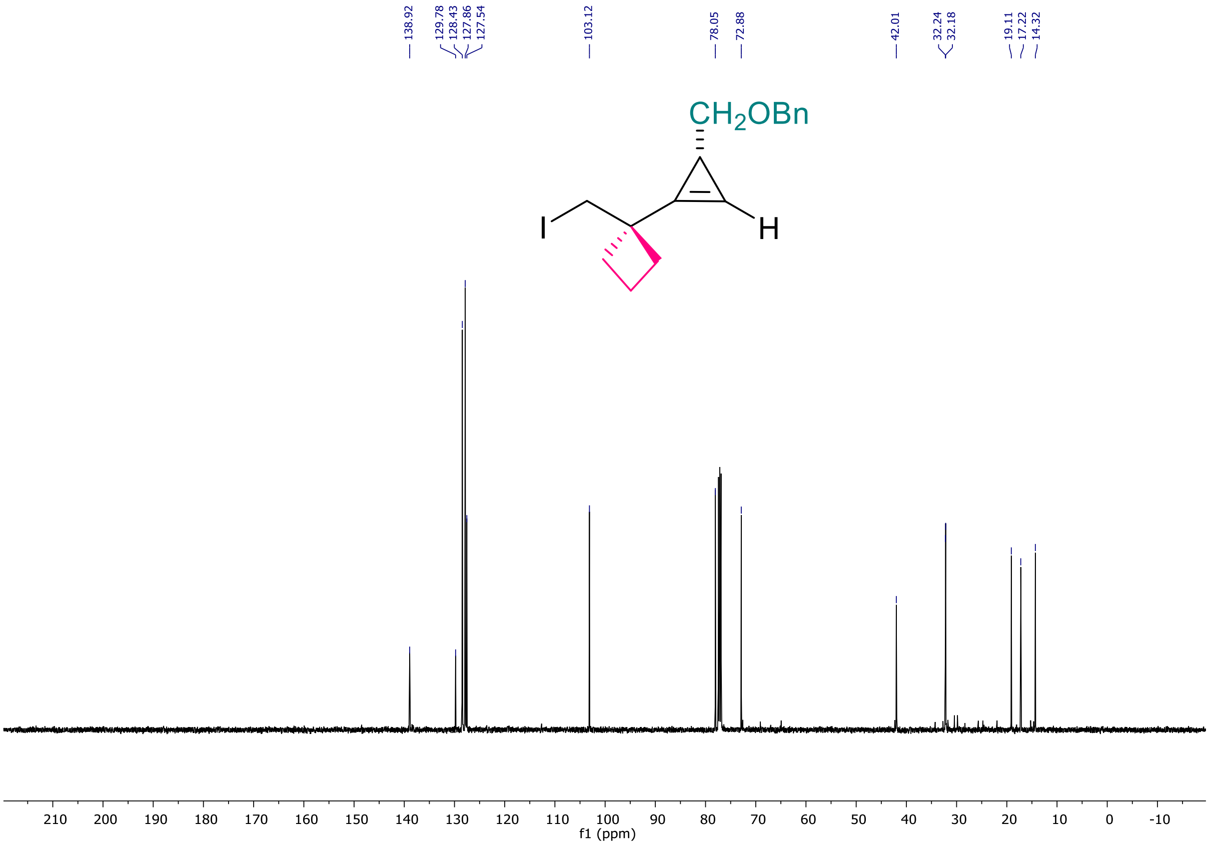
138.92
129.78
128.43
127.86
127.54
103.12
78.05
72.88
42.01
32.24
32.18
19.11
17.22
14.32
CH2OBn
I
H
210 200 190 180 170 160 150 140 130 120 110 100 90 80 70 60 50 40 30 20 10 0 -10
f1 (ppm)

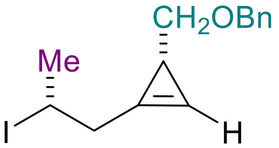
CH2OBn
Me
I
H

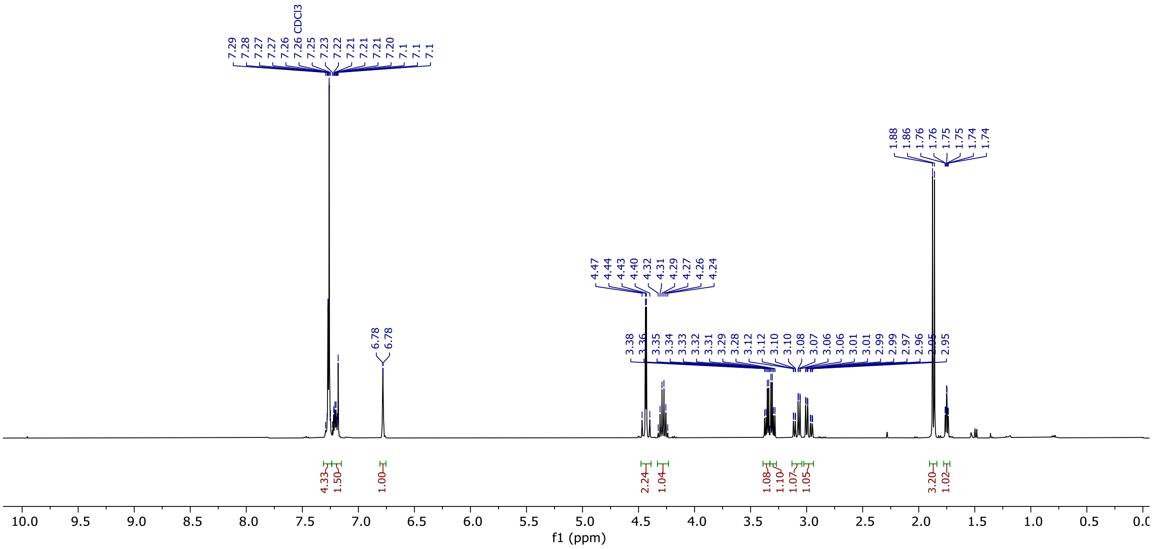
7.29
7.28
7.27
7.27
7.26
7.26 CDCl3
7.25
7.23
7.22
7.21
7.21
7.21
7.20
7.1
7.1
7.1
6.78
6.78
4.47
4.44
4.43
4.40
4.32
4.31
4.29
4.27
4.26
4.24
3.38
3.36
3.35
3.34
3.33
3.32
3.31
3.29
3.28
3.12
3.12
3.10
3.10
3.08
3.07
3.06
3.06
3.01
3.01
2.99
2.99
2.97
2.96
2.95
2.95
1.88
1.86
1.76
1.76
1.75
1.75
1.74
1.74
4.33
1.50
1.00
2.24
1.04
1.08
1.10
1.07
1.05
3.20
1.02
f1 (ppm)

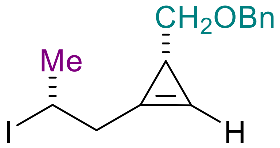
CH2OBn
Me
I
H

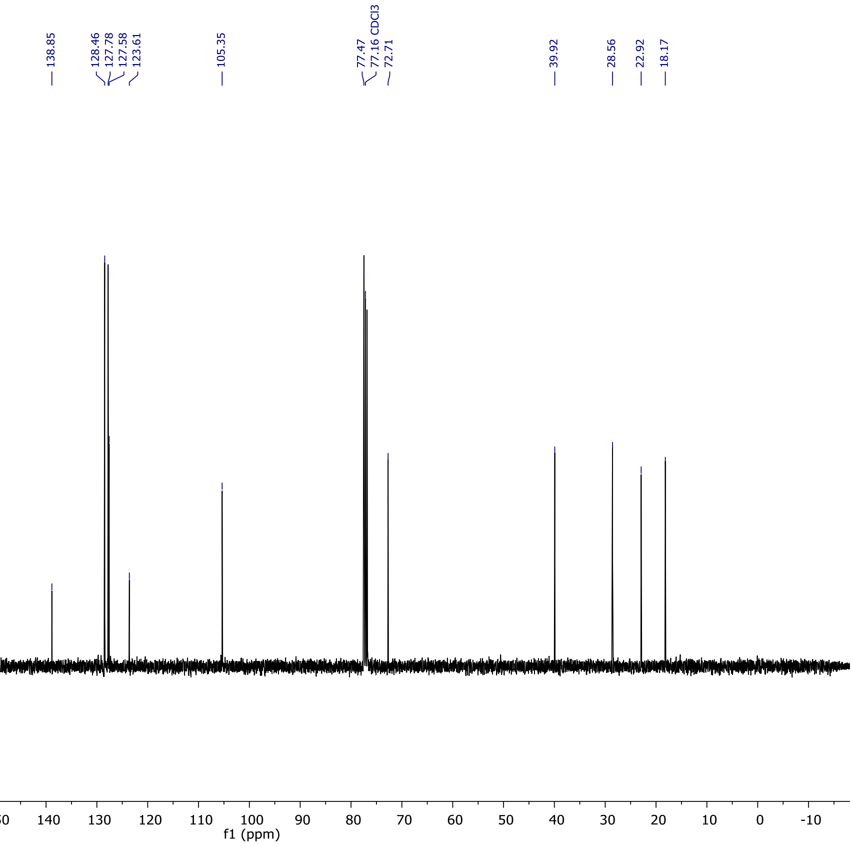
138.85
128.46
127.78
127.58
123.61
105.35
77.47
77.16 CDCl3
72.71
39.92
28.56
22.92
18.17
f1 (ppm)

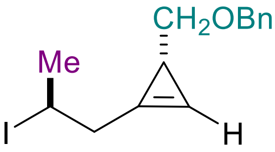
CH2OBn
Me
I
H

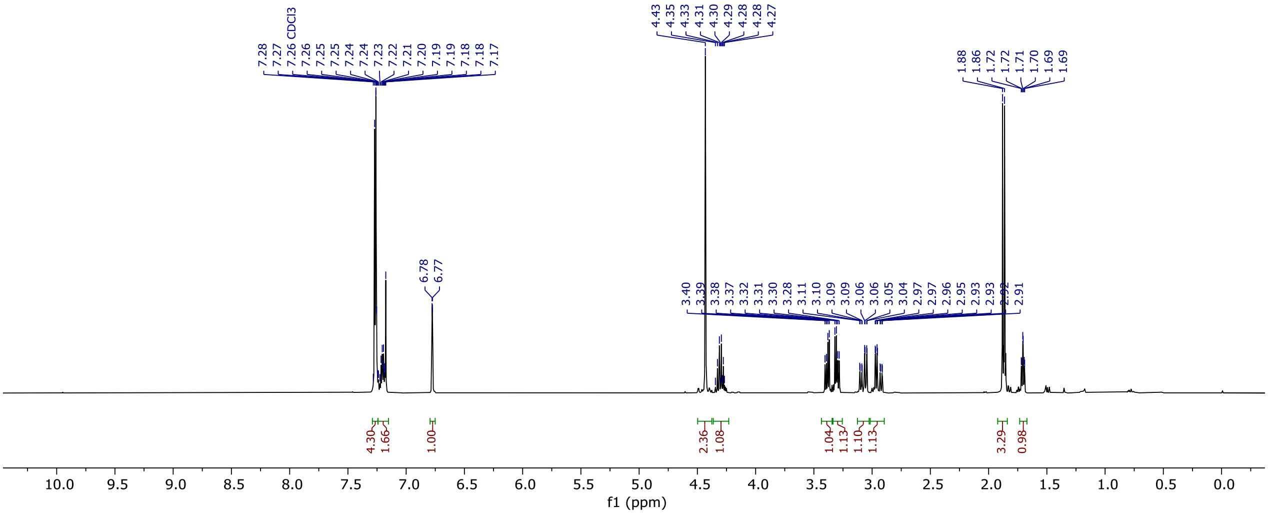
7.28 7.27 7.26 CDCl3 7.26 7.25 7.25 7.24 7.24 7.23 7.22 7.21 7.20 7.19 7.19 7.18 7.18 7.17
6.78 6.77
4.43 4.35 4.33 4.31 4.30 4.29 4.28 4.28 4.27
3.40 3.39 3.38 3.37 3.32 3.31 3.30 3.28 3.11 3.10 3.09 3.09 3.06 3.06 3.05 3.04 2.97 2.97 2.96 2.95 2.93 2.93 2.92 2.91
1.88 1.86 1.72 1.72 1.71 1.70 1.69 1.69
4.30 1.66 1.00 2.36 1.08 1.04 1.13 1.10 1.13 3.29 0.98
10.0 9.5 9.0 8.5 8.0 7.5 7.0 6.5 6.0 5.5 5.0 4.5 4.0 3.5 3.0 2.5 2.0 1.5 1.0 0.5 0.0
f1 (ppm)

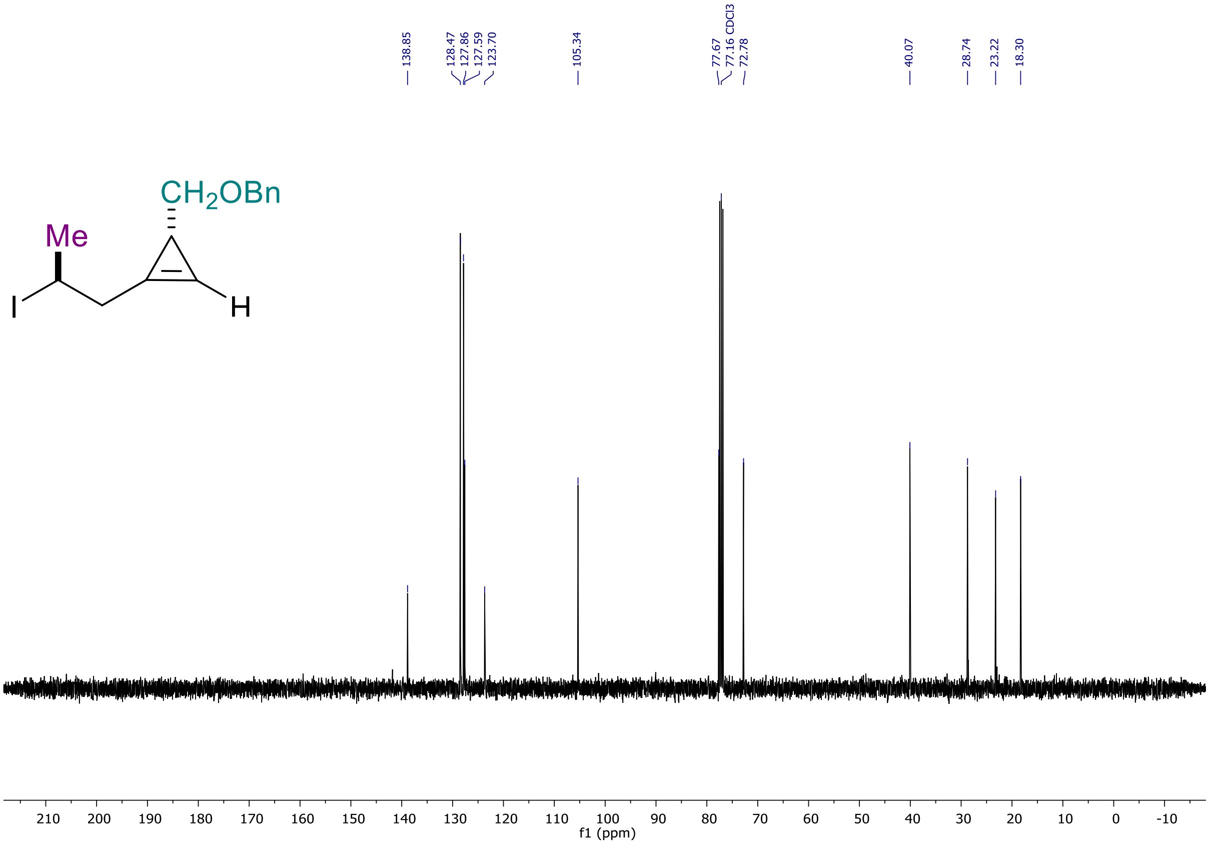
138.85
128.47 127.86 127.59 123.70
105.34
77.67 77.16 CDCl3 72.78
40.07
28.74
23.22
18.30
CH2OBn
Me
I
H
210 200 190 180 170 160 150 140 130 120 110 100 90 80 70 60 50 40 30 20 10 0 -10
f1 (ppm)

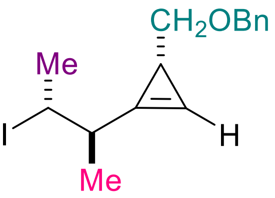
CH2OBn
Me
I
H
Me

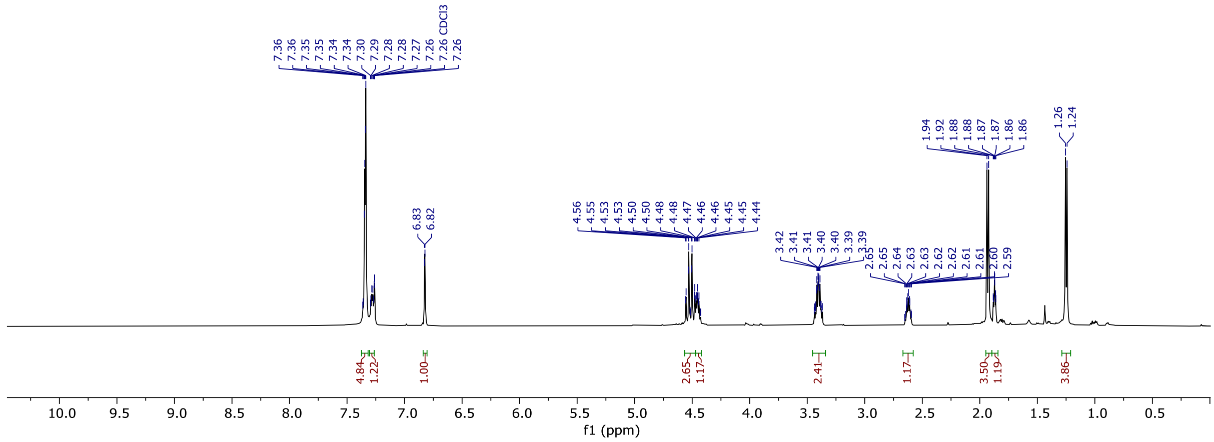
7.36 7.36 7.35 7.35 7.34 7.34 7.30 7.29 7.28 7.28 7.27 7.26 7.26 CDCl3 7.26
6.83 6.82
4.56 4.55 4.53 4.53 4.50 4.50 4.48 4.48 4.47 4.46 4.46 4.45 4.45 4.44
3.42 3.41 3.41 3.40 3.40 3.39 3.39
2.65 2.65 2.64 2.63 2.63 2.62 2.62 2.61 2.61 2.60 2.59
1.94 1.92 1.88 1.88 1.87 1.87 1.87 1.86 1.86
1.26 1.24
4.84 1.22 1.00 2.65 1.17 2.41 1.17 3.50 1.19 3.86
10.0 9.5 9.0 8.5 8.0 7.5 7.0 6.5 6.0 5.5 5.0 4.5 4.0 3.5 3.0 2.5 2.0 1.5 1.0 0.5
f1 (ppm)

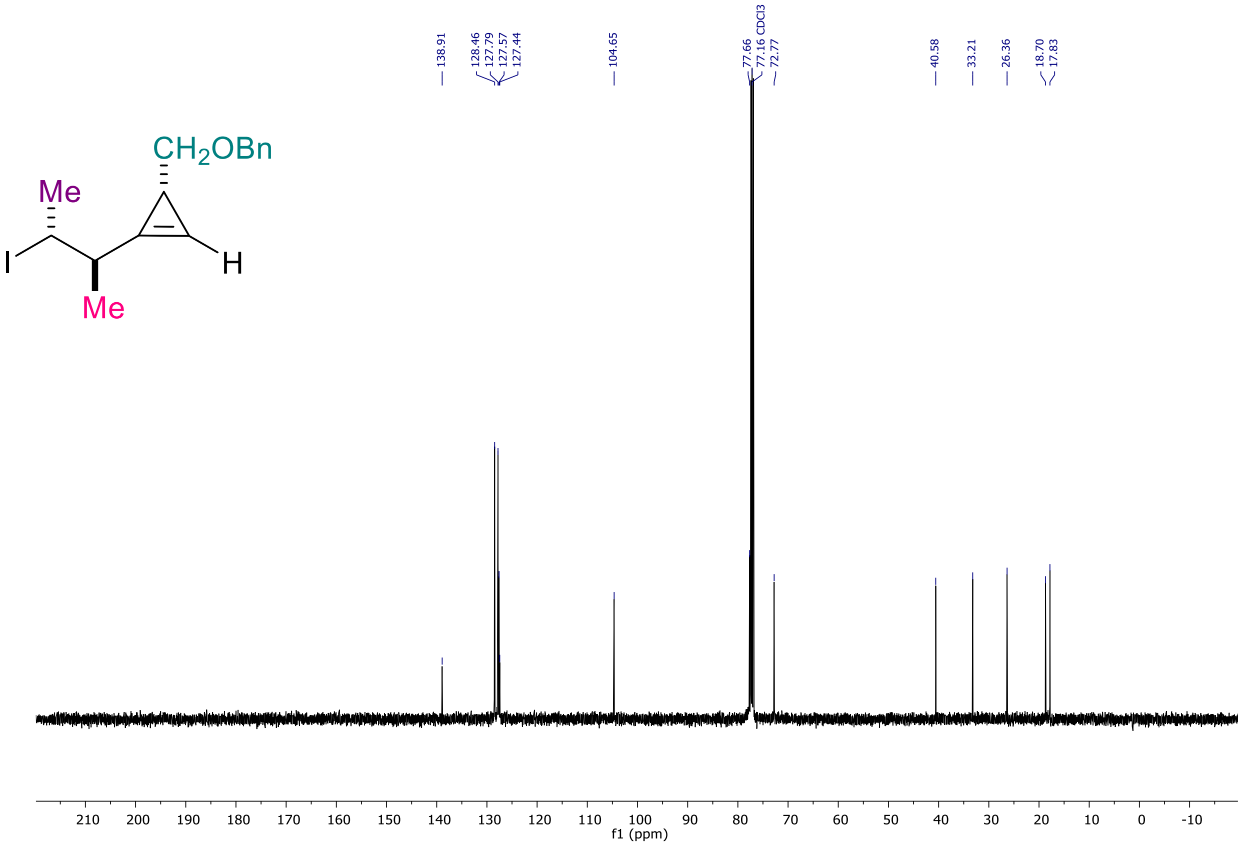
138.91
128.46 127.79 127.57 127.44
104.65
77.66 77.16 CDCl3 72.77
40.58
33.21
26.36
18.70 17.83
CH2OBn
Me
I
H
Me
210 200 190 180 170 160 150 140 130 120 110 100 90 80 70 60 50 40 30 20 10 0 -10
f1 (ppm)

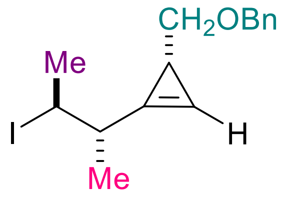
CH2OBn
Me
I
H
Me

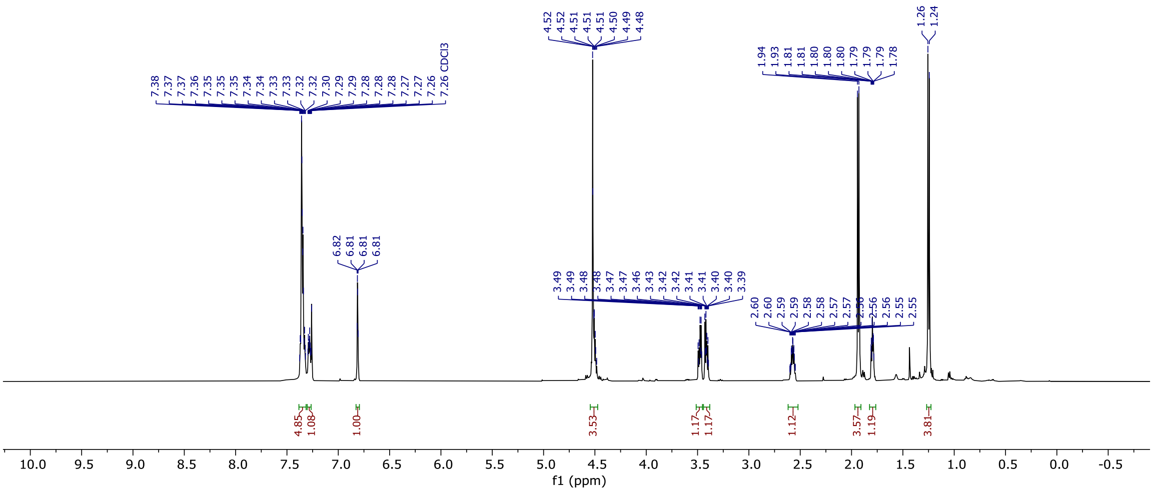
7.38 7.37 7.37 7.36 7.35 7.35 7.35 7.34 7.34 7.33 7.33 7.32 7.32 7.30 7.29 7.29 7.28 7.28 7.28 7.27 7.27 7.26 CDCl3
6.82 6.81 6.81 6.81
4.52 4.52 4.51 4.51 4.51 4.50 4.49 4.48
3.49 3.49 3.48 3.48 3.47 3.47 3.46 3.43 3.42 3.42 3.41 3.41 3.40 3.40 3.39
2.60 2.60 2.59 2.59 2.58 2.58 2.57 2.57 2.56 2.56 2.56 2.55 2.55
1.94 1.93 1.81 1.81 1.80 1.80 1.80 1.79 1.79 1.79 1.79 1.78
1.26 1.24
4.85 1.08 1.00 3.53 1.17 1.17 1.12 3.57 1.19 3.81
10.0 9.5 9.0 8.5 8.0 7.5 7.0 6.5 6.0 5.5 5.0 4.5 4.0 3.5 3.0 2.5 2.0 1.5 1.0 0.5 0.0 -0.5
f1 (ppm)

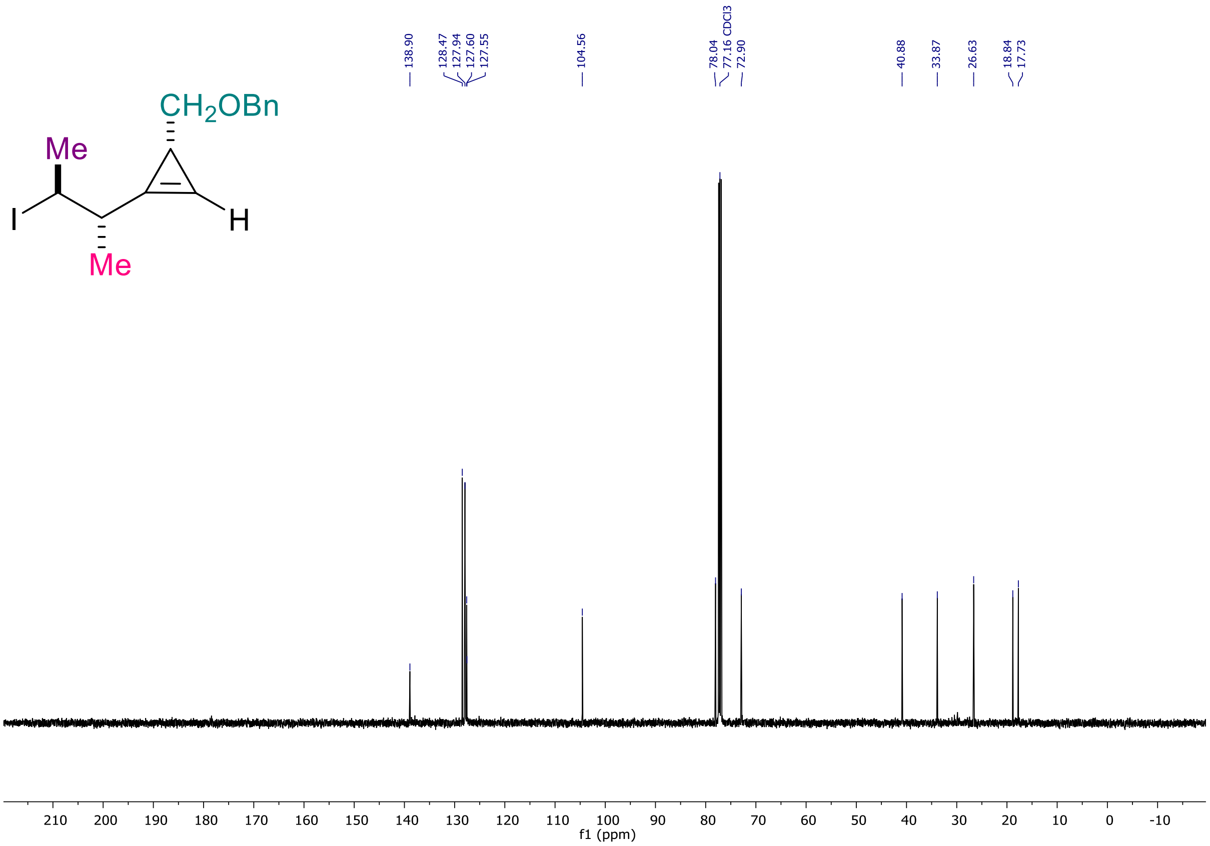
138.90
128.47 127.94 127.60 127.55
104.56
78.04 77.16 CDCl3 72.90
40.88
33.87
26.63
18.84 17.73
CH2OBn
Me
I
H
Me
210 200 190 180 170 160 150 140 130 120 110 100 90 80 70 60 50 40 30 20 10 0 -10
f1 (ppm)

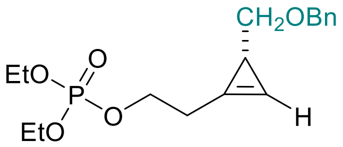
CH2OBn
EtO
EtO
P
O
O
H

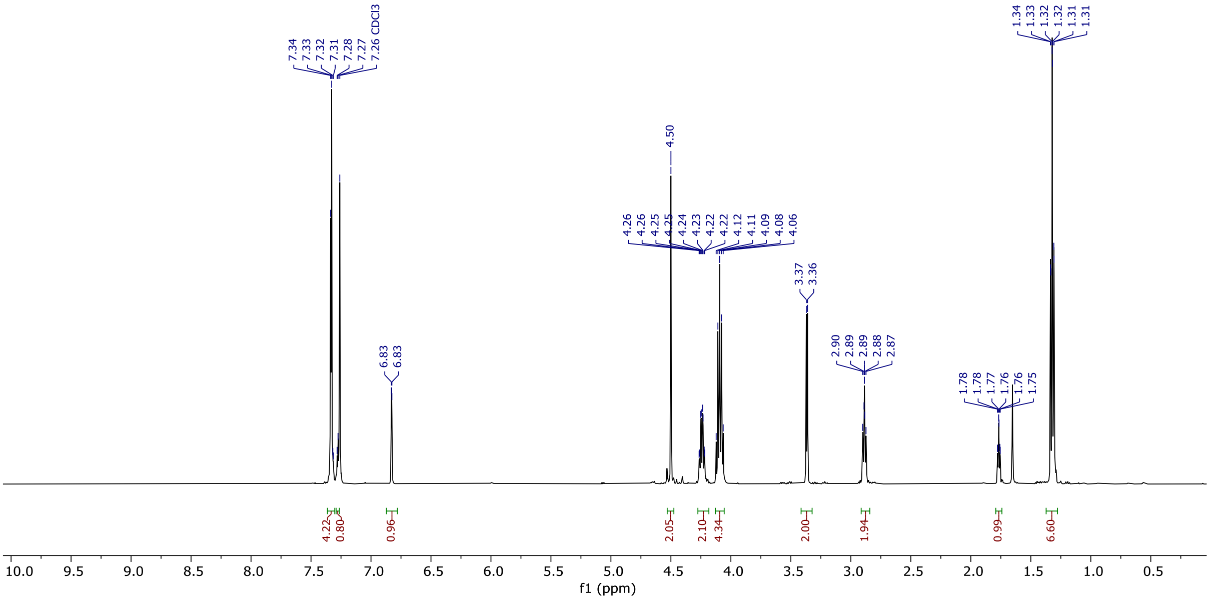
7.34
7.33
7.32
7.31
7.28
7.27
7.26 CDCl3
6.83
6.83
4.50
4.26
4.26
4.25
4.25
4.24
4.23
4.22
4.22
4.12
4.11
4.09
4.08
4.06
3.37
3.36
2.90
2.89
2.89
2.88
2.87
1.78
1.78
1.77
1.76
1.76
1.75
1.34
1.33
1.32
1.32
1.31
1.31
4.22
0.80
0.96
2.05
2.10
4.34
2.00
1.94
0.99
6.60
10.0 9.5 9.0 8.5 8.0 7.5 7.0 6.5 6.0 5.5 5.0 4.5 4.0 3.5 3.0 2.5 2.0 1.5 1.0 0.5
f1 (ppm)

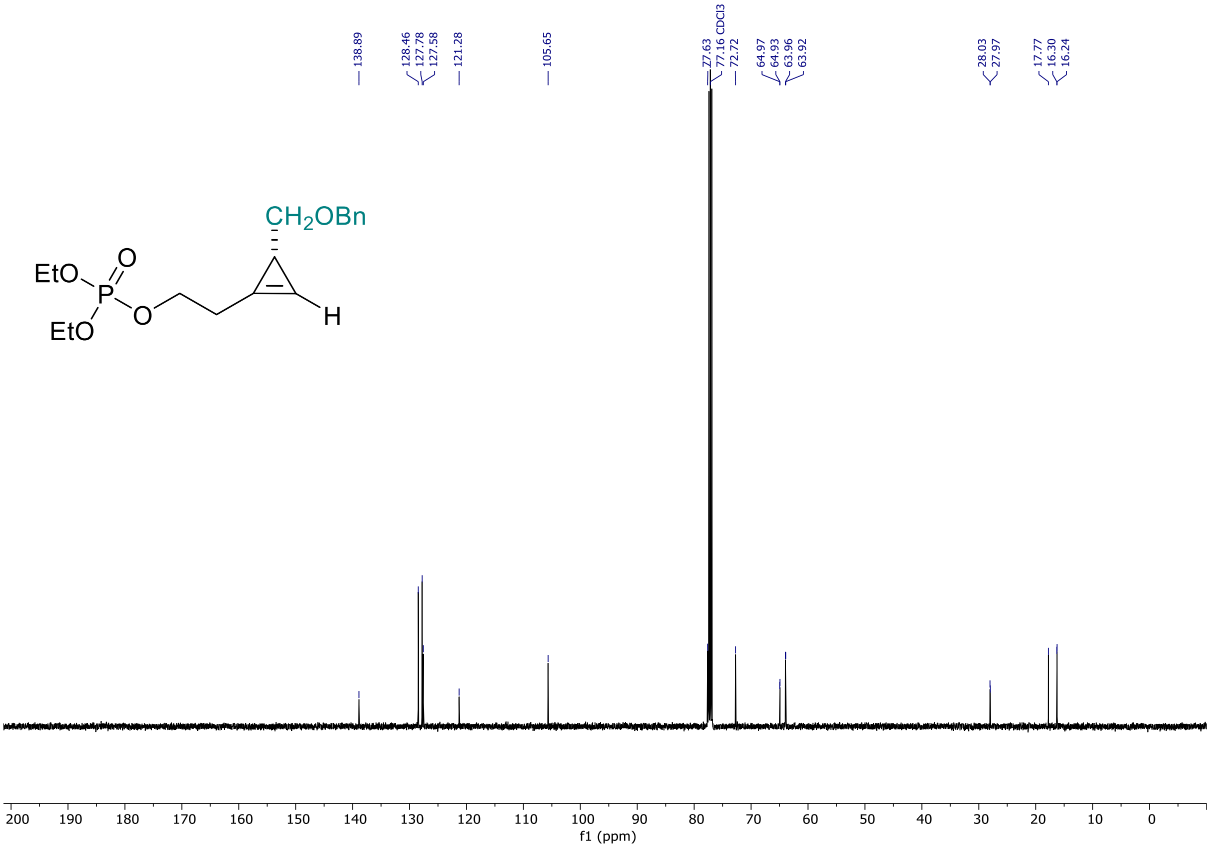
138.89
128.46
127.78
127.58
121.28
105.65
77.63
77.16 CDCl3
72.72
64.97
64.93
63.96
63.92
28.03
27.97
17.77
16.30
16.24
CH2OBn
EtO
EtO
P
O
O
H
200 190 180 170 160 150 140 130 120 110 100 90 80 70 60 50 40 30 20 10 0
f1 (ppm)

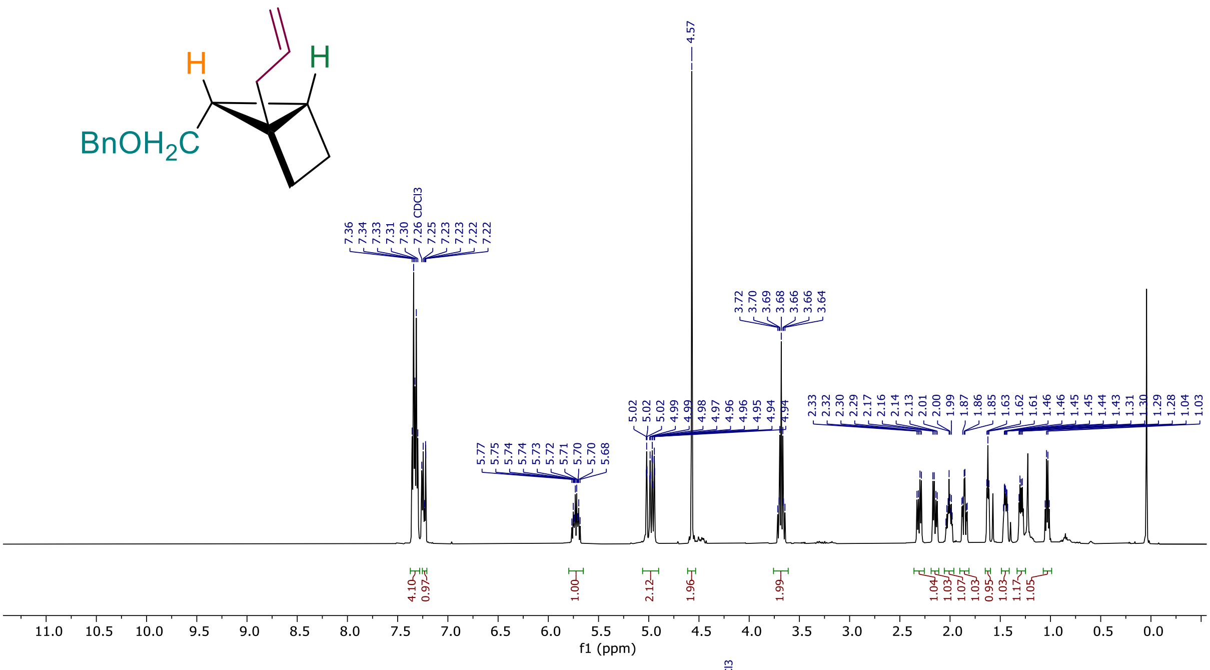
BnOH2C
H
H
f1 (ppm)
4.57
7.36
7.34
7.33
7.31
7.30
7.26 CDCl3
7.25
7.23
7.23
7.23
7.22
7.22
3.72
3.70
3.69
3.68
3.66
3.66
3.64
4.10
0.97
1.00
2.12
1.96
1.99

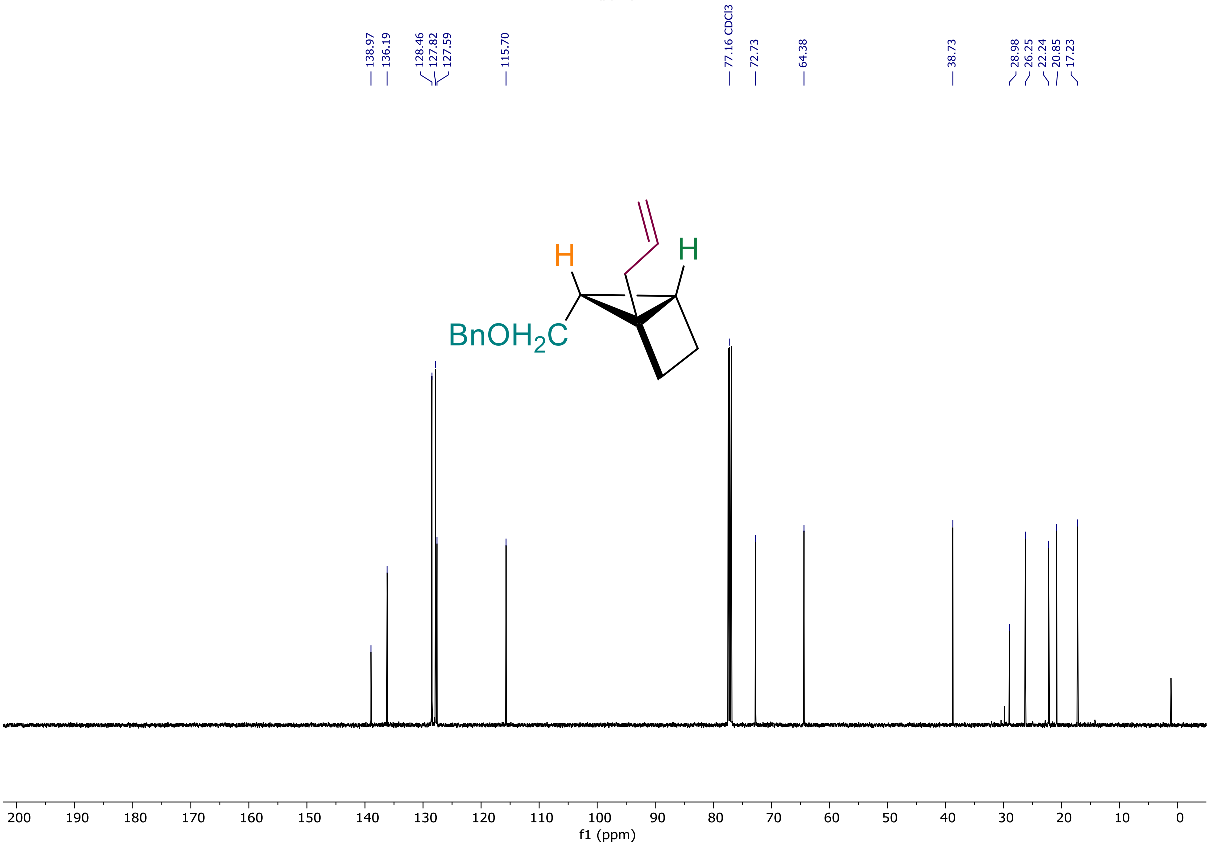
138.97
136.19
128.46
127.82
127.59
115.70
77.16 CDCl3
72.73
64.38
38.73
28.98
26.25
22.24
20.85
17.23
BnOH2C
H
H
f1 (ppm)

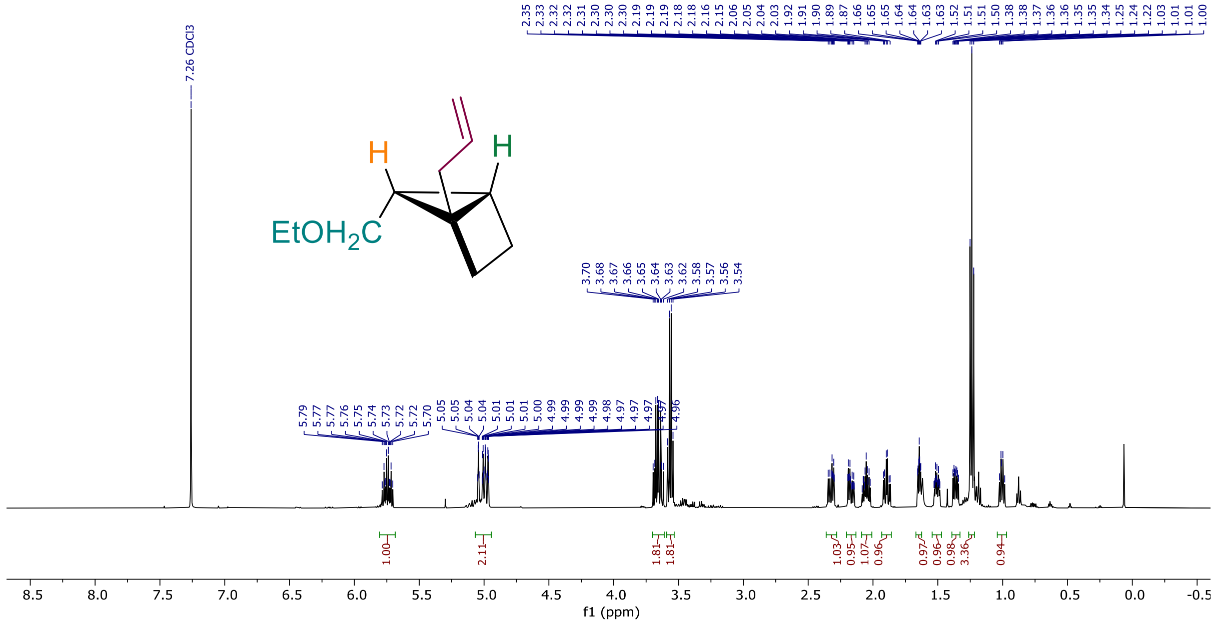
EtOH2C
H
H
7.26 CDCl3
f1 (ppm)

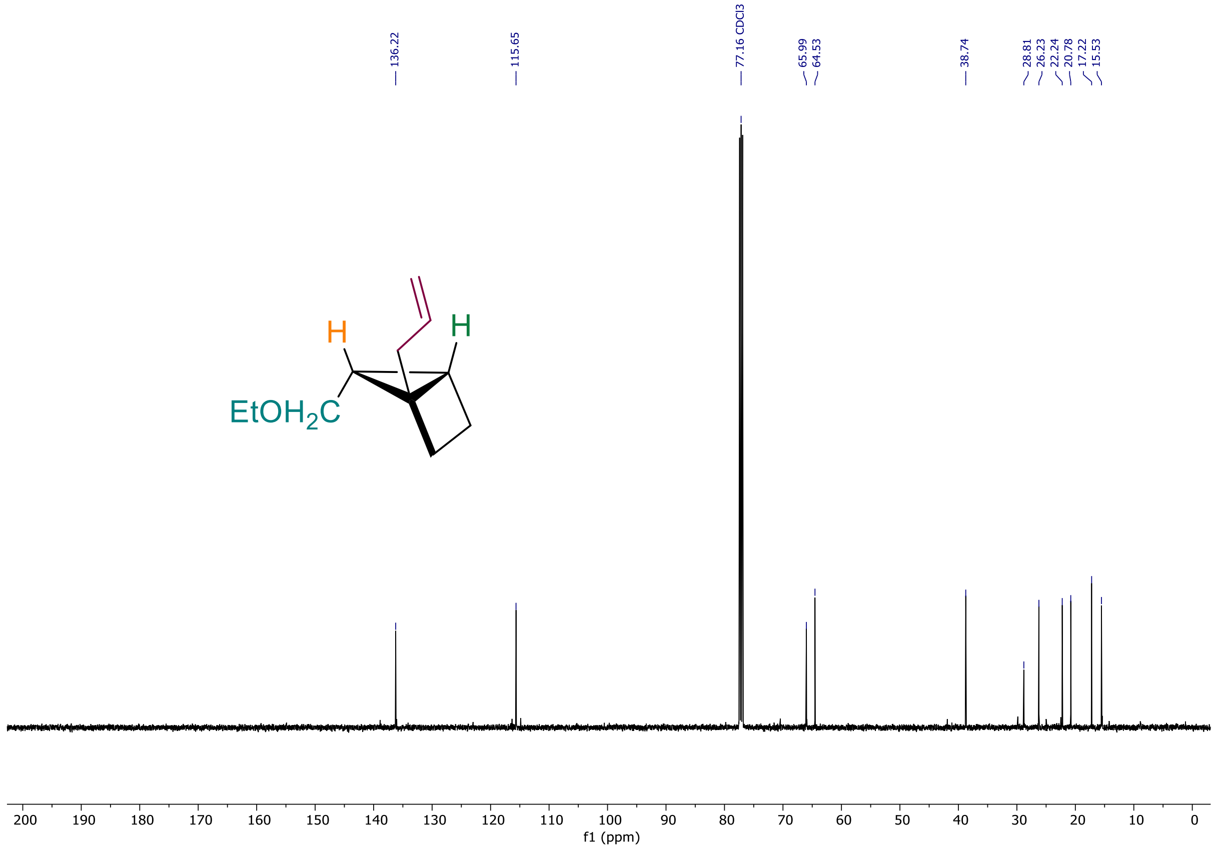
EtOH2C
H
H
136.22
115.65
77.16 CDCl3
65.99
64.53
38.74
28.81
26.23
22.24
20.78
17.22
15.53
f1 (ppm)

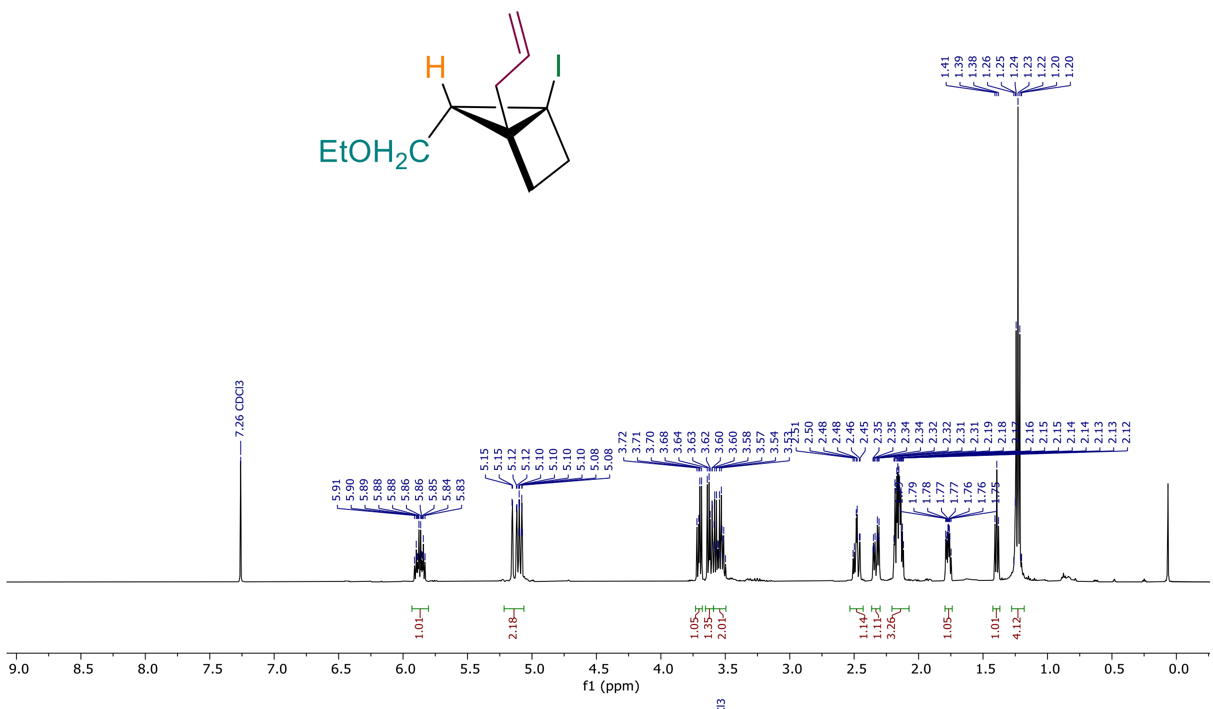
EtOH2C
H
I
7.26 CDCl3
5.91 5.90 5.89 5.88 5.88 5.86 5.86 5.85 5.84 5.83
5.15 5.15 5.12 5.12 5.10 5.10 5.10 5.10 5.08 5.08
3.72 3.71 3.70 3.68 3.64 3.63 3.62 3.60 3.60 3.58 3.57 3.54 3.53
2.51 2.50 2.48 2.46 2.45 2.35 2.35 2.34 2.34 2.32 2.32 2.31 2.19 2.18 2.17 2.16 2.15 2.15 2.14 2.14 2.13 2.13 2.12
1.79 1.78 1.77 1.77 1.76 1.76 1.75
1.41 1.39 1.38 1.26 1.25 1.24 1.23 1.22 1.20 1.20
1.01 2.18 1.05 1.35 2.01 1.14 1.11 3.26 1.05 1.01 4.12
9.0 8.5 8.0 7.5 7.0 6.5 6.0 5.5 5.0 4.5 4.0 3.5 3.0 2.5 2.0 1.5 1.0 0.5 0.0
f1 (ppm)

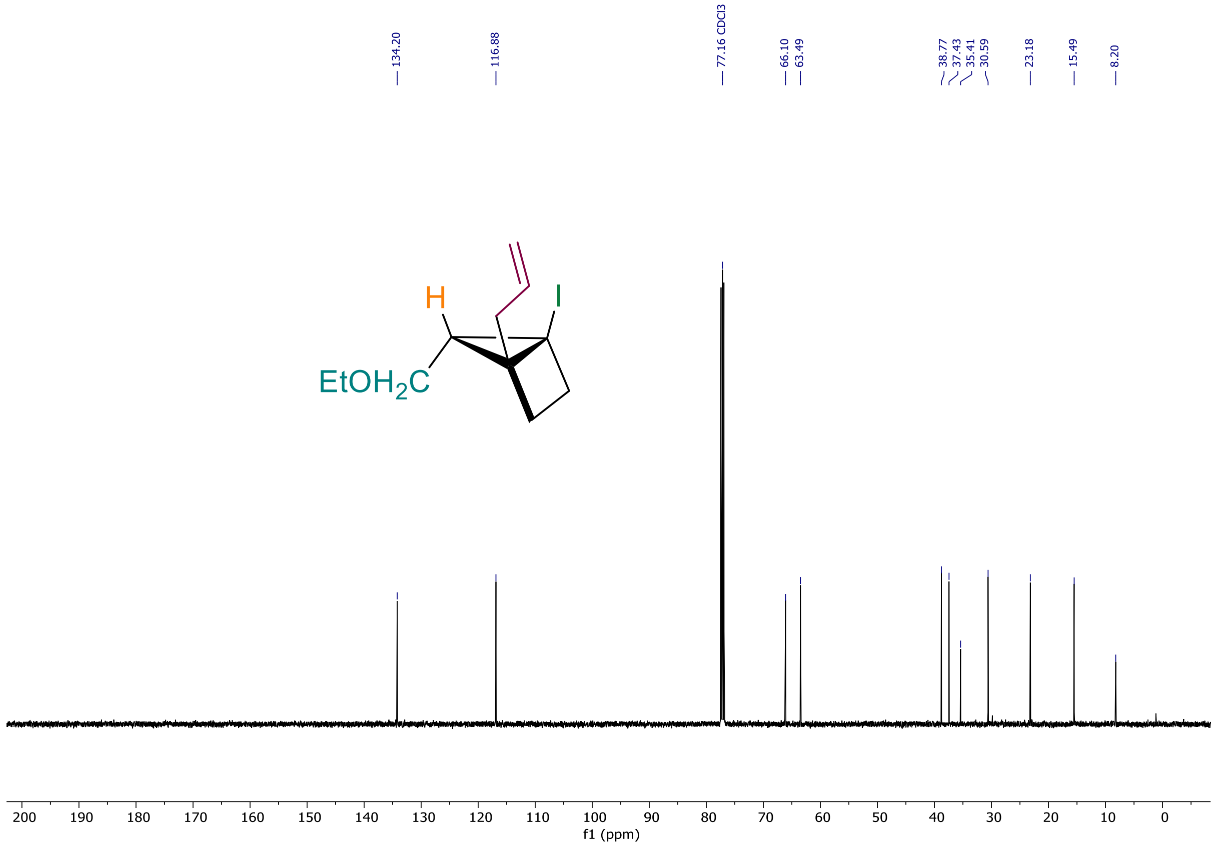
134.20
116.88
77.16 CDCl3
66.10
63.49
38.77
37.43
35.41
30.59
23.18
15.49
8.20
EtOH2C
H
I
200 190 180 170 160 150 140 130 120 110 100 90 80 70 60 50 40 30 20 10 0
f1 (ppm)

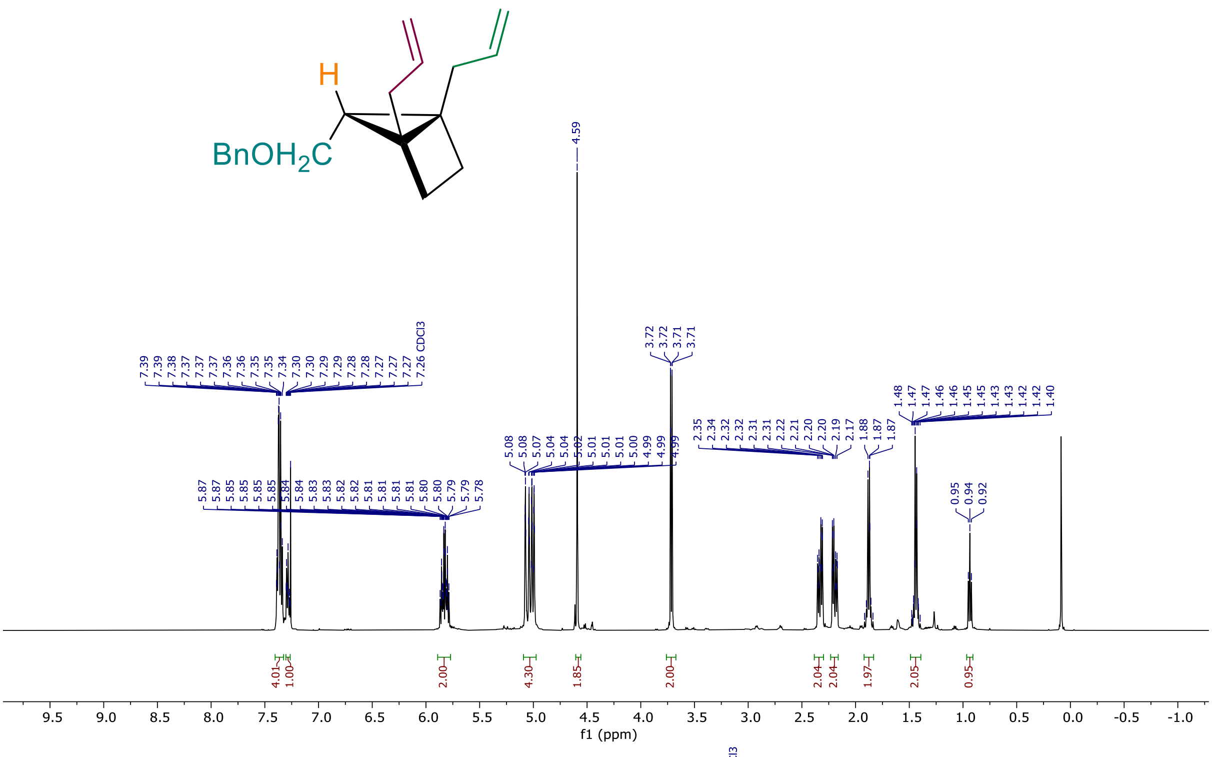
BnOH2C
H
4.59
3.72
3.72
3.71
3.71
7.26 CDCl3
f1 (ppm)

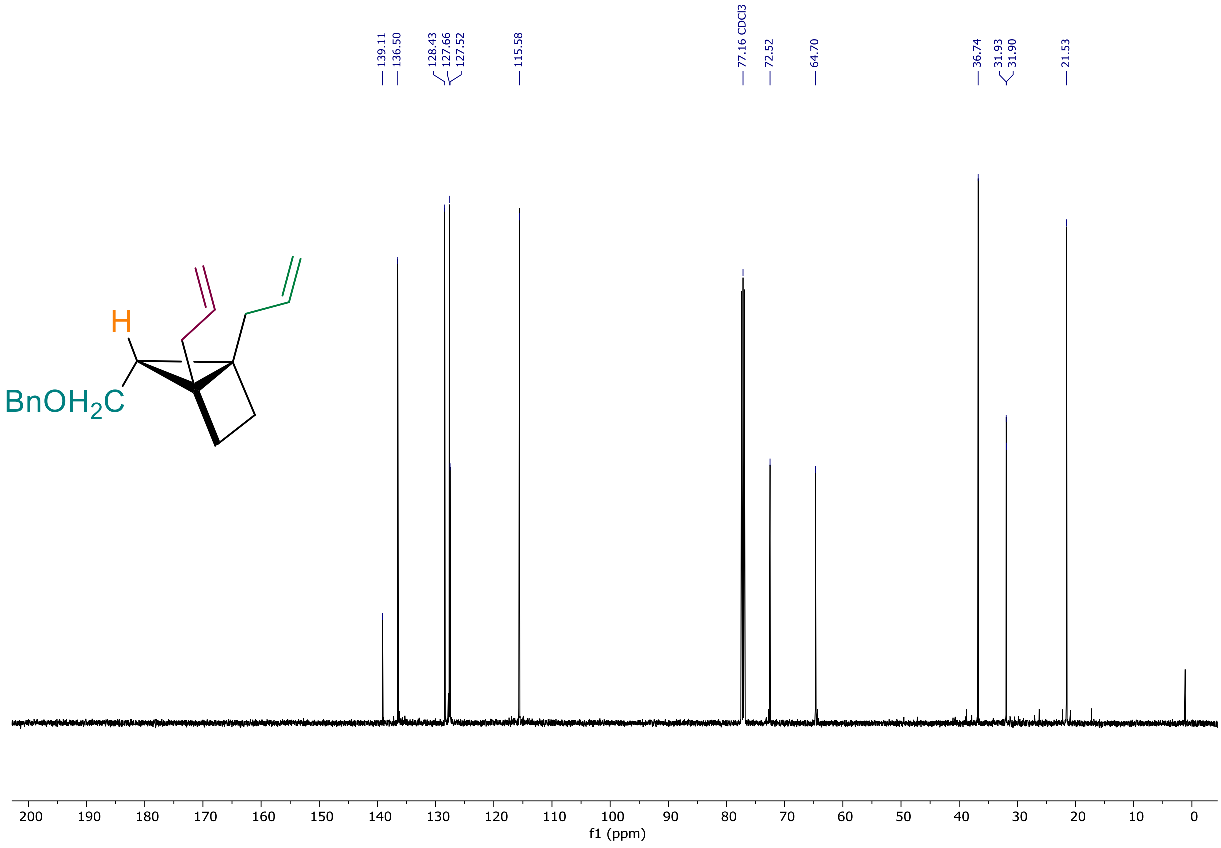
BnOH2C
H
139.11
136.50
128.43
127.66
127.52
115.58
77.16 CDCl3
72.52
64.70
36.74
31.93
31.90
21.53
f1 (ppm)

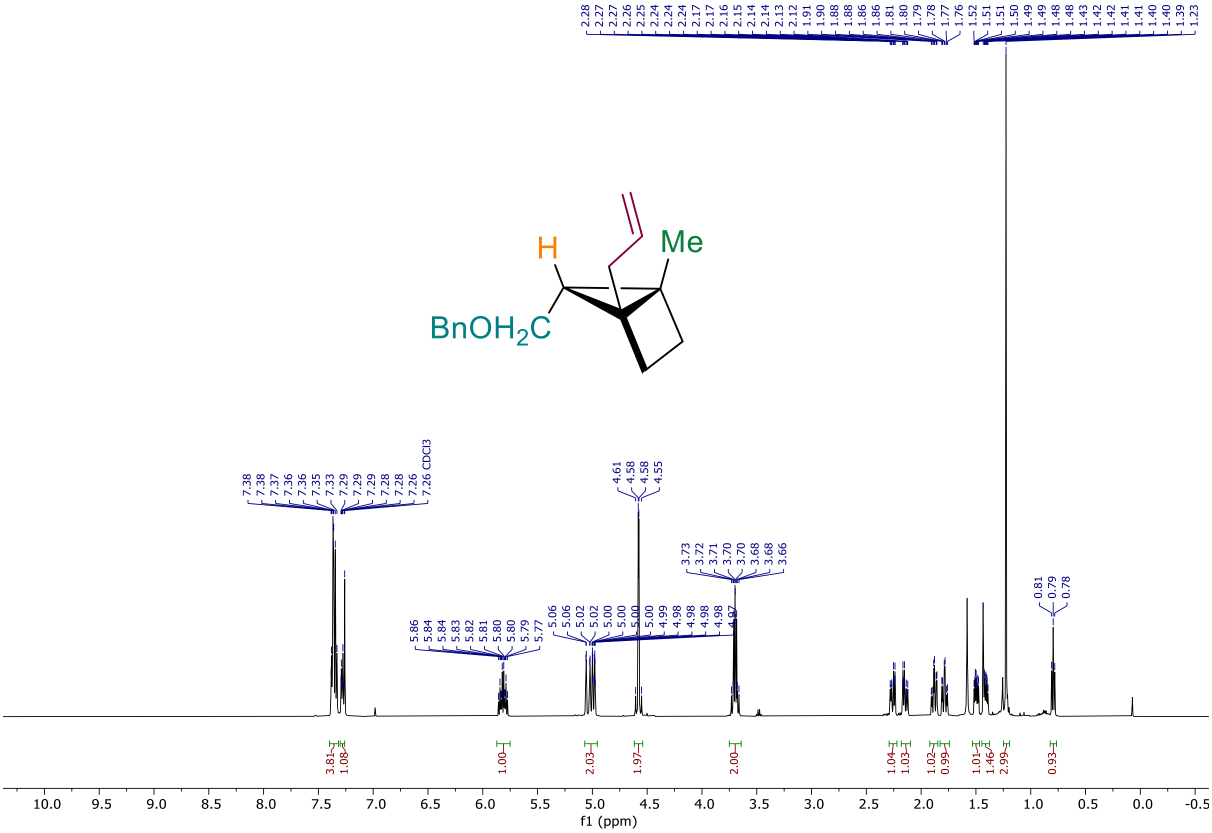

2.28 2.27 2.27 2.26 2.25 2.24 2.24 2.24 2.17 2.17 2.16 2.15 2.14 2.14 2.13 2.12 1.91 1.90 1.88 1.88 1.86 1.81 1.80 1.79 1.78 1.77 1.76 1.52 1.51 1.51 1.50 1.49 1.49 1.48 1.48 1.43 1.42 1.42 1.41 1.41 1.40 1.39 1.23
H
Me
BnOH2C
7.38 7.38 7.37 7.36 7.36 7.35 7.33 7.29 7.29 7.29 7.28 7.28 7.26 7.26 CDCl3
5.86 5.84 5.84 5.83 5.82 5.81 5.80 5.80 5.79 5.77
5.06 5.06 5.02 5.02 5.00 5.00 5.00 5.00 4.99 4.98 4.98 4.98 4.98 4.98 4.97
4.61 4.58 4.58 4.55
3.73 3.72 3.71 3.70 3.70 3.68 3.68 3.66
0.81 0.79 0.78
3.81 1.08 1.00 2.03 1.97 2.00 1.04 1.03 1.02 0.99 1.01 1.46 2.99 0.93
10.0 9.5 9.0 8.5 8.0 7.5 7.0 6.5 6.0 5.5 5.0 4.5 4.0 3.5 3.0 2.5 2.0 1.5 1.0 0.5 0.0 -0.5
f1 (ppm)

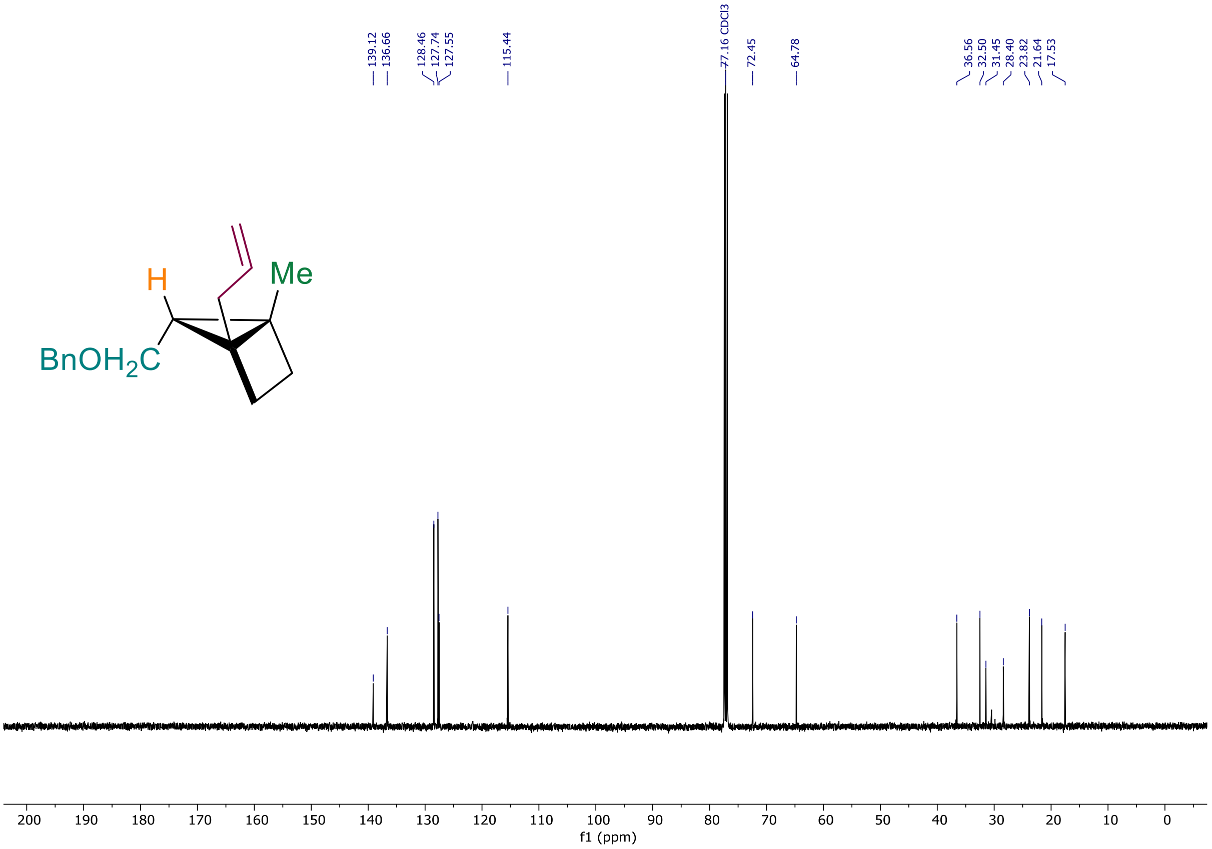

139.12 136.66 128.46 127.74 127.55 115.44
77.16 CDCl3
72.45 64.78
36.56 32.50 31.45 28.40 23.82 21.64 17.53
H
Me
BnOH2C
200 190 180 170 160 150 140 130 120 110 100 90 80 70 60 50 40 30 20 10 0
f1 (ppm)

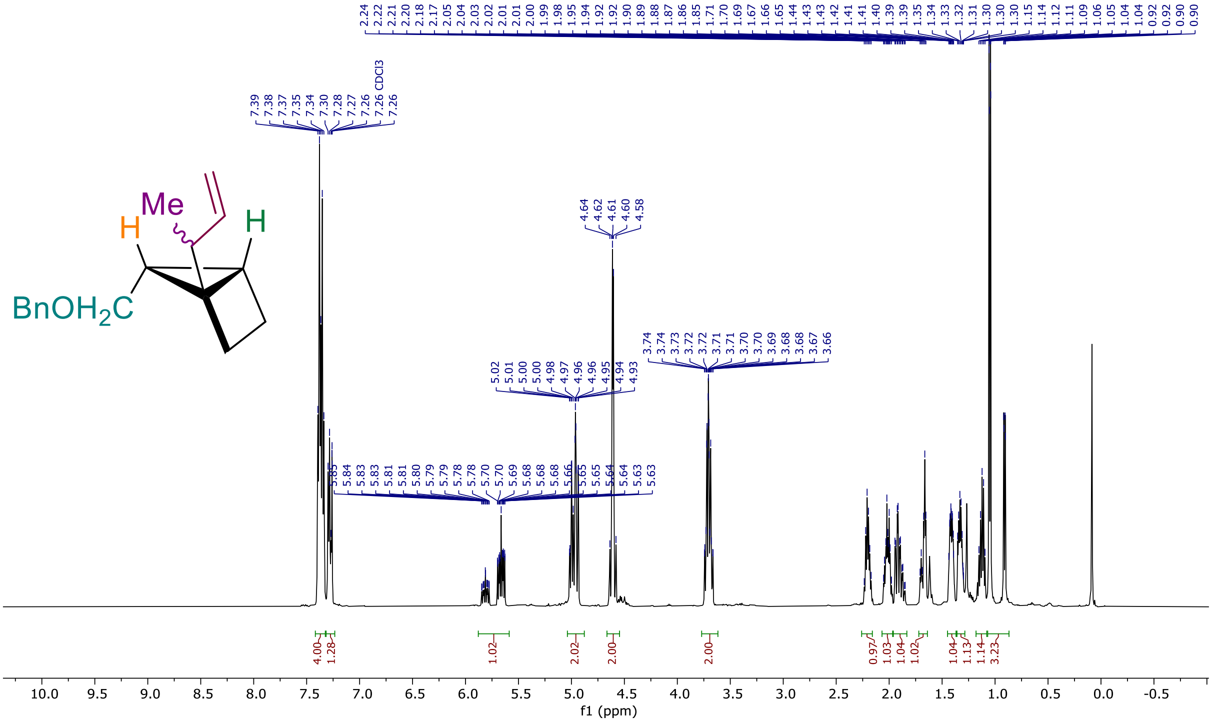
2.24 2.22 2.21 2.20 2.18 2.17 2.05 2.04 2.03 2.02 2.01 2.01 2.00 1.99 1.98 1.95 1.94 1.92 1.92 1.90 1.89 1.88 1.87 1.86 1.85 1.71 1.70 1.69 1.67 1.66 1.65 1.44 1.43 1.43 1.42 1.41 1.40 1.39 1.39 1.35 1.34 1.33 1.32 1.31 1.30 1.30 1.30 1.15 1.14 1.12 1.11 1.09 1.06 1.05 1.04 1.04 0.92 0.92 0.90 0.90
7.39 7.38 7.37 7.35 7.34 7.30 7.28 7.27 7.26 7.26 CDCl3 7.26
4.64 4.62 4.61 4.60 4.58
5.02 5.01 5.00 5.00 4.98 4.97 4.96 4.96 4.95 4.94 4.93
3.74 3.74 3.73 3.72 3.72 3.71 3.71 3.70 3.70 3.69 3.68 3.68 3.67 3.66
5.85 5.84 5.83 5.83 5.81 5.81 5.80 5.79 5.79 5.78 5.78 5.70 5.70 5.69 5.68 5.68 5.68 5.66 5.65 5.65 5.64 5.64 5.63 5.63
Me
H
H
BnOH2C
4.00 1.28 1.02 2.02 2.00 2.00 0.97 1.03 1.04 1.02 1.04 1.13 1.14 3.23
10.0 9.5 9.0 8.5 8.0 7.5 7.0 6.5 6.0 5.5 5.0 4.5 4.0 3.5 3.0 2.5 2.0 1.5 1.0 0.5 0.0 -0.5
f1 (ppm)

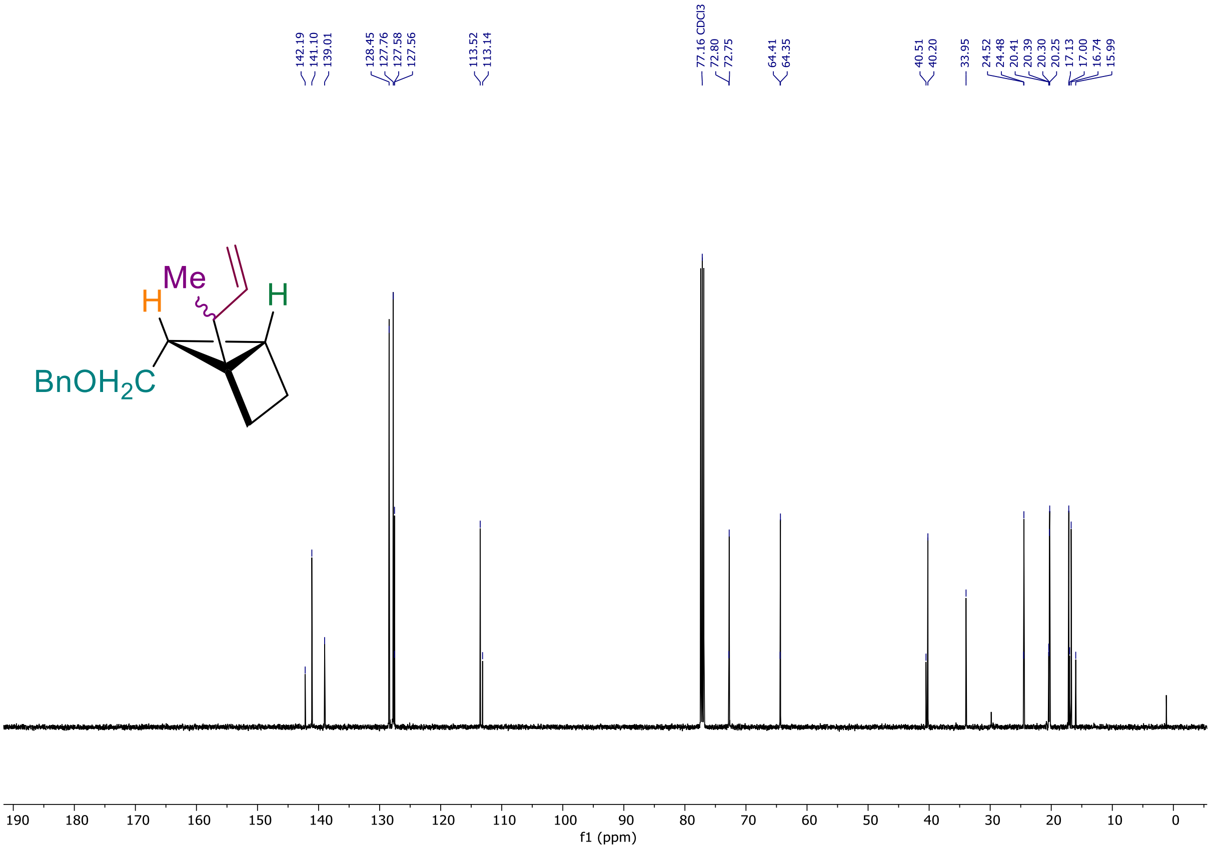
142.19 141.10 139.01
128.45 127.76 127.58 127.56
113.52 113.14
77.16 CDCl3 72.80 72.75
64.41 64.35
40.51 40.20
33.95
24.52 24.48 20.41 20.39 20.30 20.25 17.13 17.00 16.74 15.99
Me
H
H
BnOH2C
190 180 170 160 150 140 130 120 110 100 90 80 70 60 50 40 30 20 10 0
f1 (ppm)

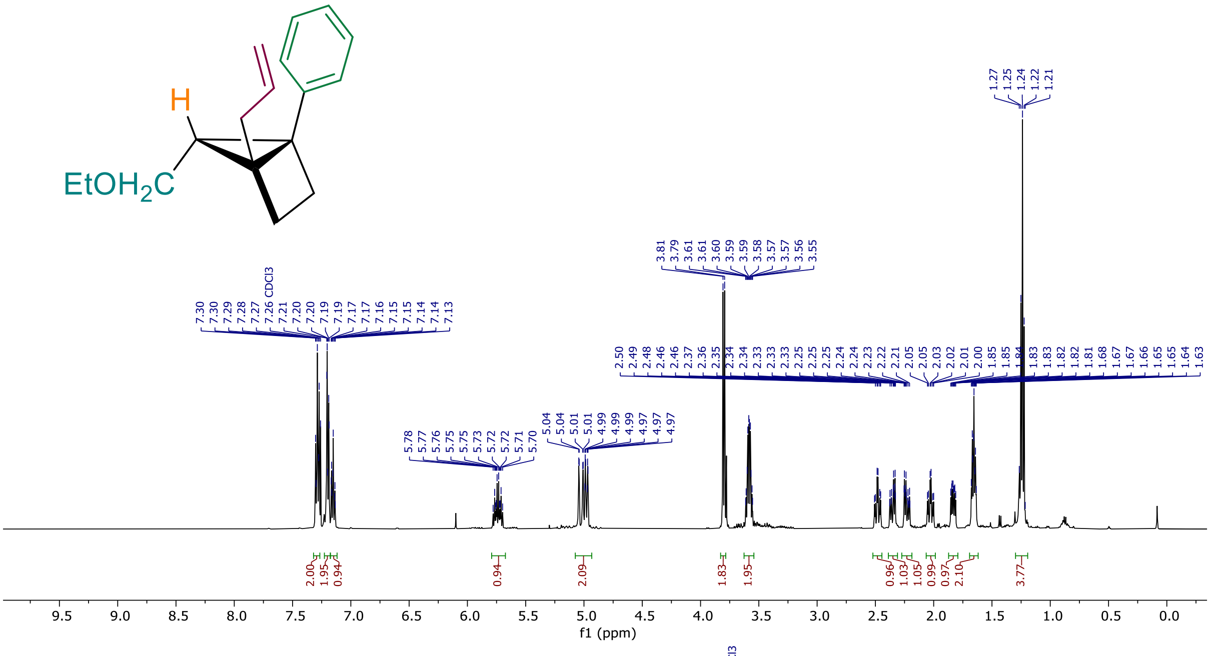
H
EtOH2C
f1 (ppm)
7.30 7.30 7.29 7.28 7.27 7.26 CDCl3 7.21 7.20 7.20 7.19 7.19 7.17 7.17 7.16 7.15 7.15 7.14 7.14 7.13
5.78 5.77 5.76 5.75 5.75 5.73 5.72 5.72 5.71 5.70
5.04 5.04 5.01 5.01 4.99 4.99 4.99 4.97 4.97
3.81 3.79 3.61 3.61 3.60 3.59 3.59 3.58 3.57 3.57 3.56 3.55
1.27 1.25 1.24 1.22 1.21
2.00 1.95 0.94 0.94 2.09 1.83 1.95 0.96 1.03 1.05 0.99 0.97 2.10 3.77

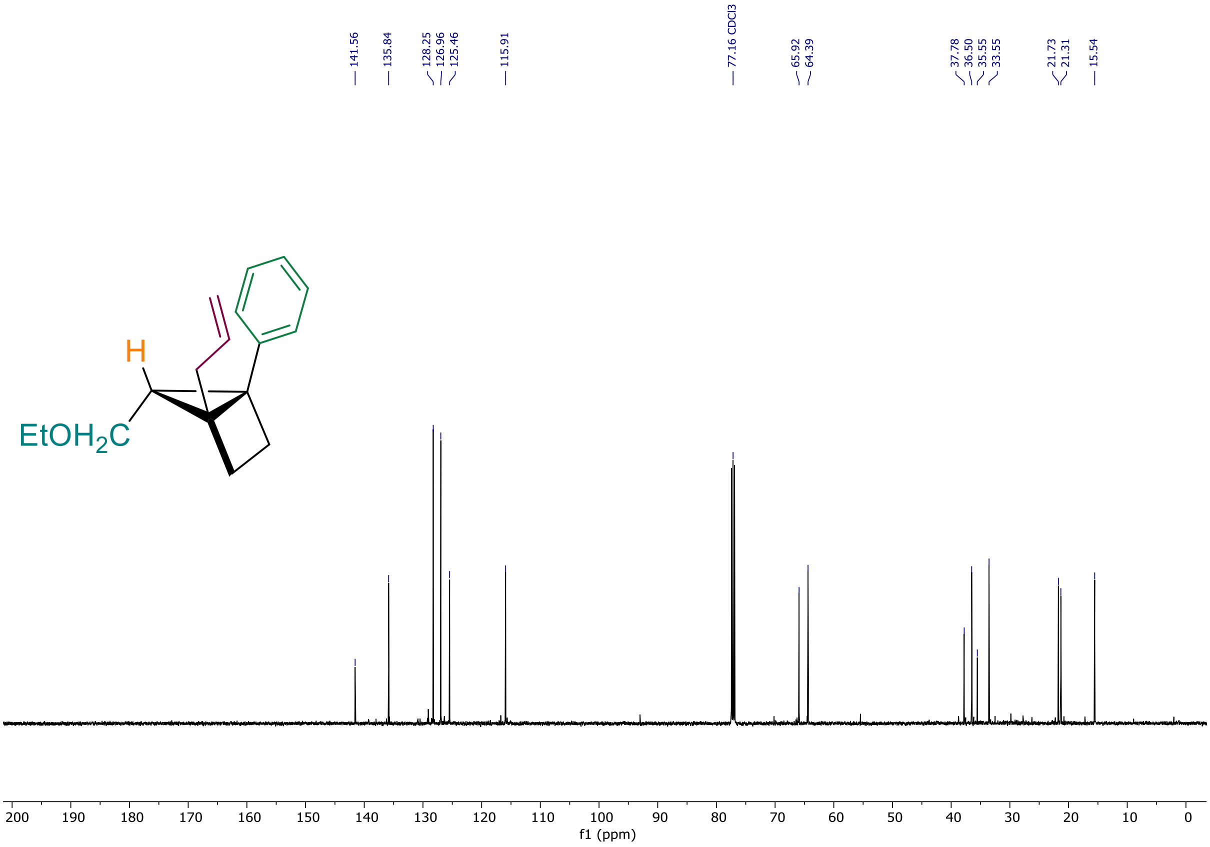
141.56 135.84 128.25 126.96 125.46 115.91 77.16 CDCl3 65.92 64.39 37.78 36.50 35.55 33.55 21.73 21.31 15.54
H
EtOH2C
f1 (ppm)

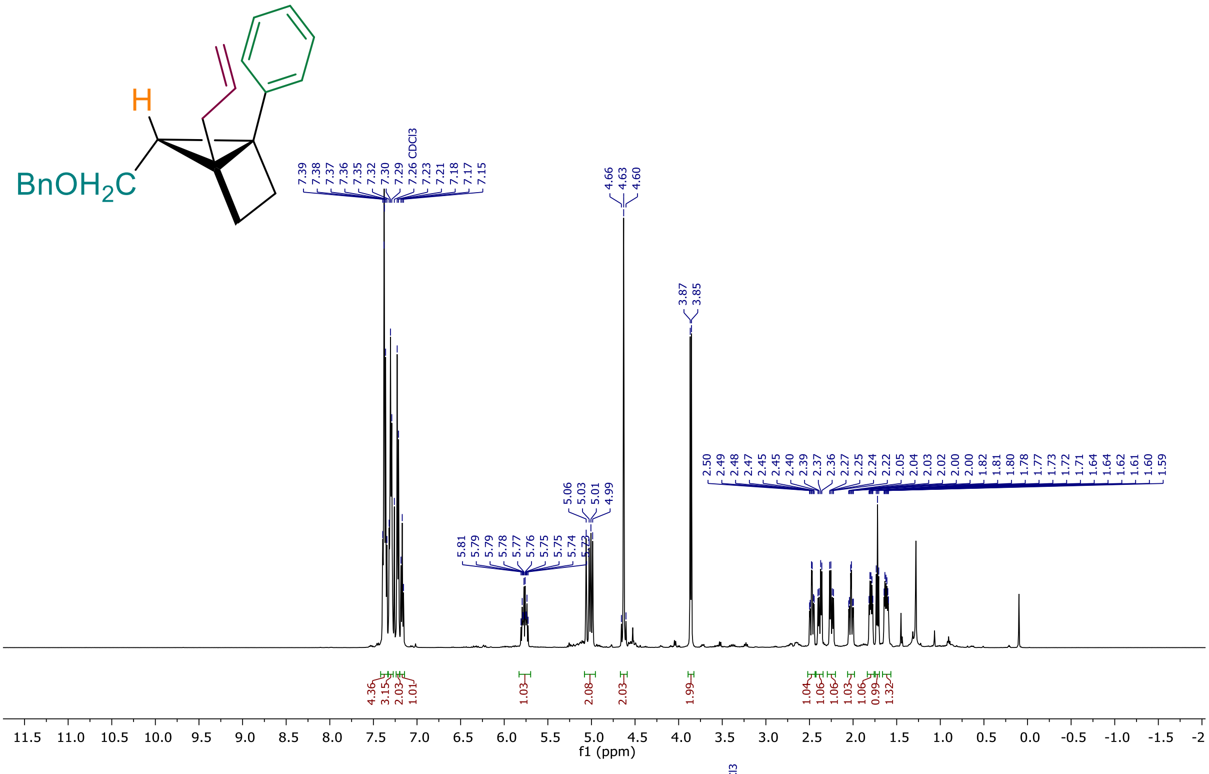
H
BnOH2C
7.39 7.38 7.37 7.36 7.35 7.32 7.30 7.29 7.26 CDCl3 7.23 7.21 7.18 7.17 7.15
4.66 4.63 4.60
3.87 3.85
5.81 5.79 5.79 5.78 5.77 5.76 5.75 5.75 5.74 5.72
5.06 5.03 5.01 4.99
2.50 2.49 2.48 2.47 2.45 2.45 2.40 2.39 2.37 2.36 2.27 2.25 2.24 2.22 2.05 2.04 2.03 2.02 2.00 2.00 1.82 1.81 1.80 1.78 1.77 1.73 1.72 1.71 1.64 1.64 1.62 1.61 1.60 1.59
4.36 3.15 2.03 1.01 1.03 2.08 2.03 1.99 1.04 1.06 1.06 1.03 1.06 0.99 1.32
11.5 11.0 10.5 10.0 9.5 9.0 8.5 8.0 7.5 7.0 6.5 6.0 5.5 5.0 4.5 4.0 3.5 3.0 2.5 2.0 1.5 1.0 0.5 0.0 -0.5 -1.0 -1.5 -2
f1 (ppm)

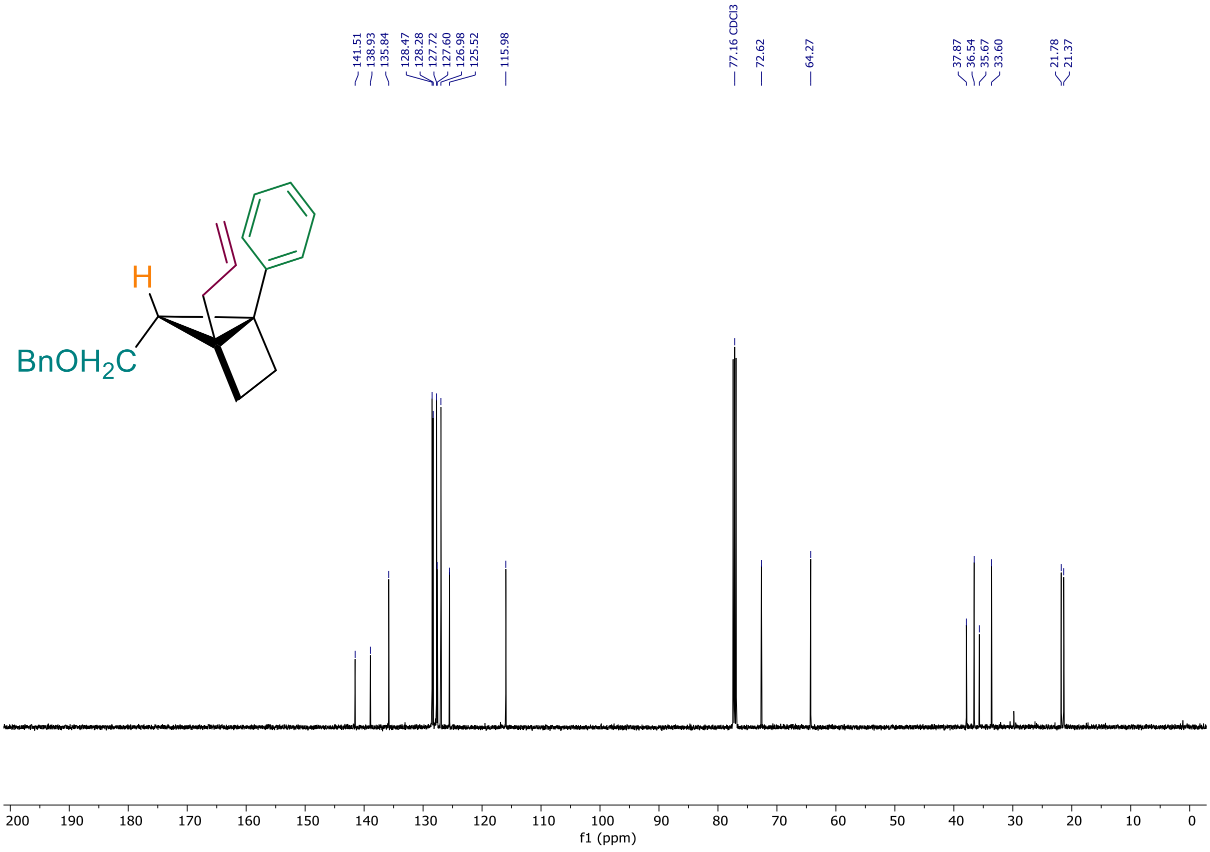
141.51 138.93 135.84 128.47 128.28 127.72 127.60 126.98 125.52 115.98
77.16 CDCl3
72.62
64.27
37.87 36.54 35.67 33.60
21.78 21.37
H
BnOH2C
200 190 180 170 160 150 140 130 120 110 100 90 80 70 60 50 40 30 20 10 0
f1 (ppm)

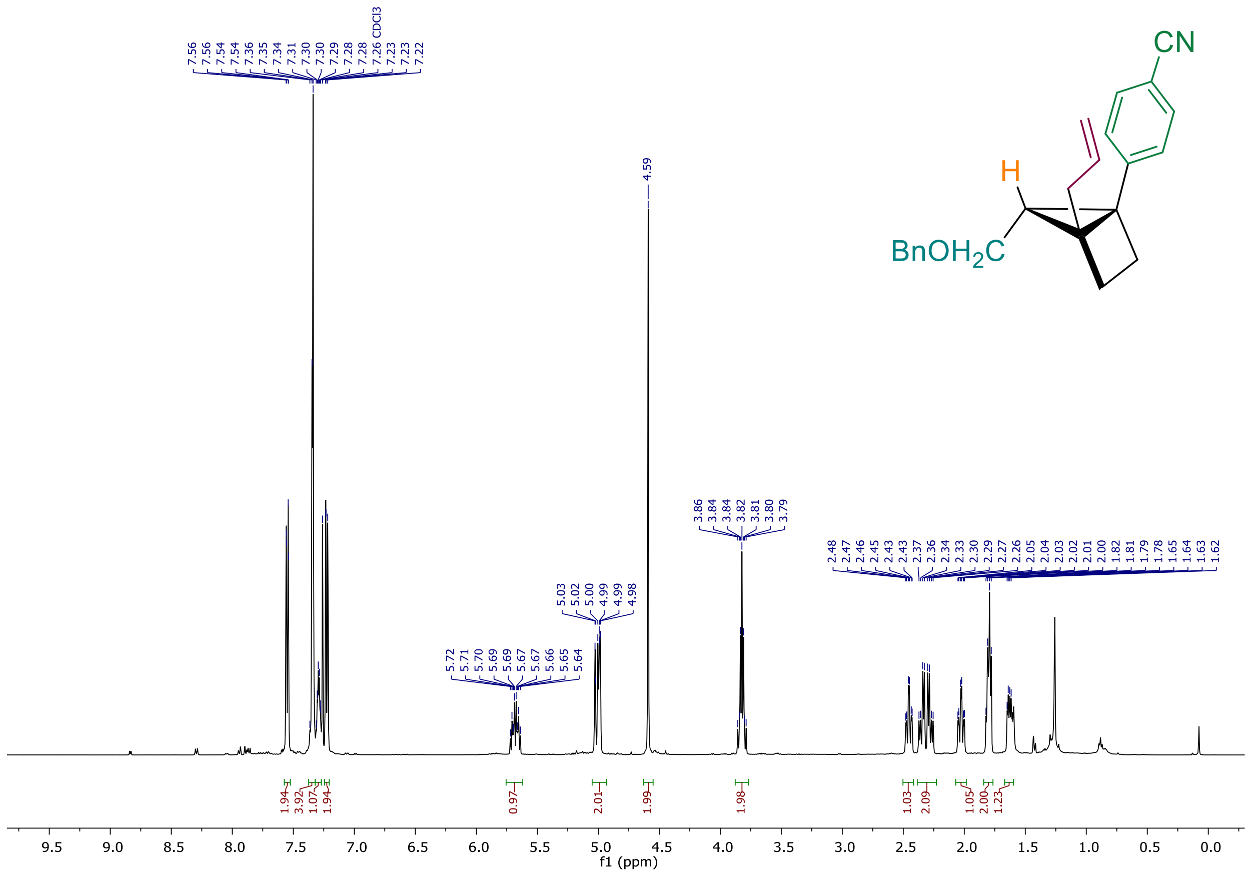
7.56 7.56 7.54 7.54 7.36 7.35 7.34 7.31 7.30 7.30 7.29 7.28 7.28 7.26 CDCl3 7.23 7.23 7.22
4.59
5.72 5.71 5.70 5.69 5.69 5.67 5.67 5.66 5.65 5.64
5.03 5.02 5.00 4.99 4.99 4.98
3.86 3.84 3.84 3.82 3.81 3.80 3.79
2.48 2.47 2.46 2.45 2.43 2.43 2.37 2.36 2.34 2.33 2.30 2.29 2.27 2.26 2.05 2.04 2.03 2.02 2.01 2.00 1.82 1.81 1.79 1.78 1.65 1.64 1.63 1.62
1.94 3.92 1.07 1.94 0.97 2.01 1.99 1.98 1.03 2.09 1.05 2.00 1.23
9.5 9.0 8.5 8.0 7.5 7.0 6.5 6.0 5.5 5.0 4.5 4.0 3.5 3.0 2.5 2.0 1.5 1.0 0.5 0.0
f1 (ppm)
CN
H
BnOH2C

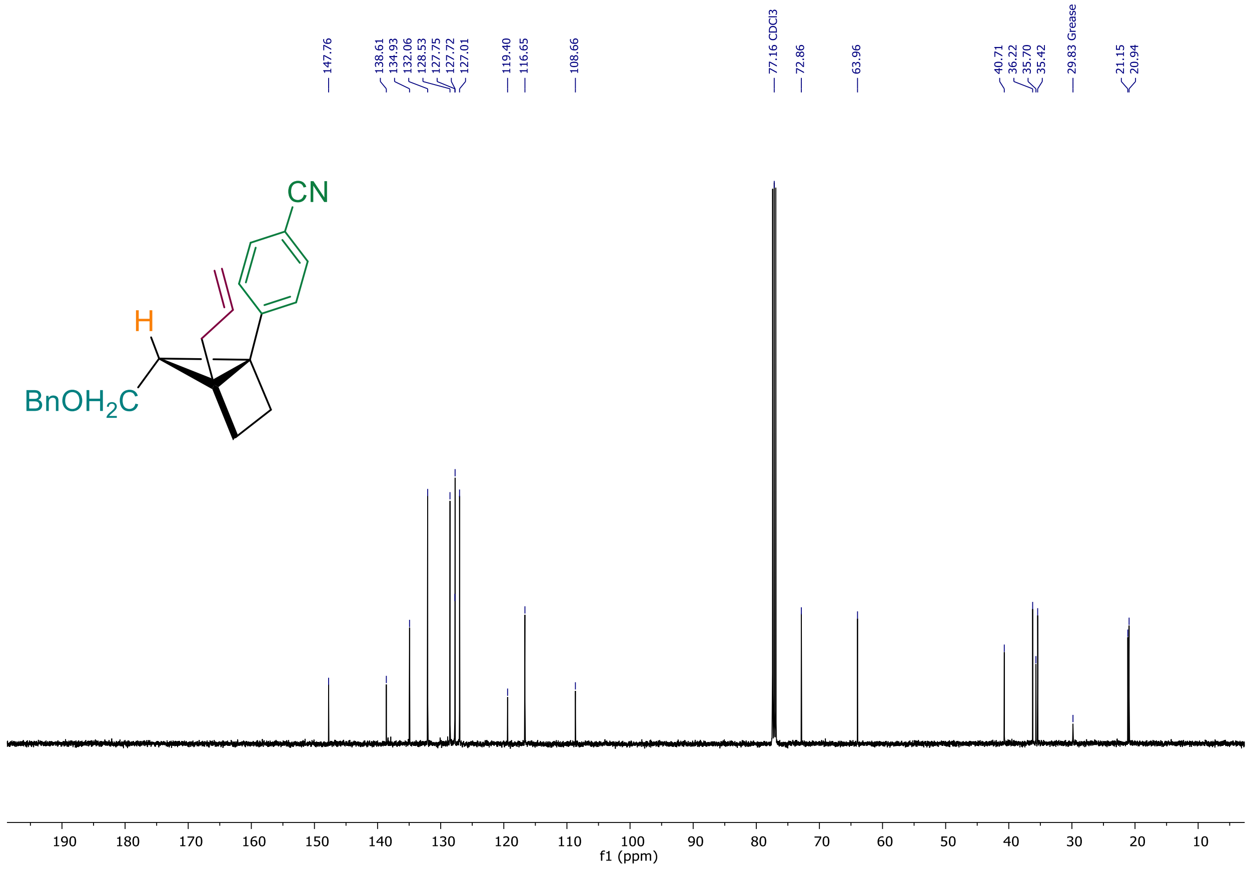
147.76
138.61 134.93 132.06 128.53 127.75 127.72 127.01
119.40 116.65
108.66
77.16 CDCl3
72.86
63.96
40.71 36.22 35.70 35.42
29.83 Grease
21.15 20.94
CN
H
BnOH2C
190 180 170 160 150 140 130 120 110 100 90 80 70 60 50 40 30 20 10
f1 (ppm)

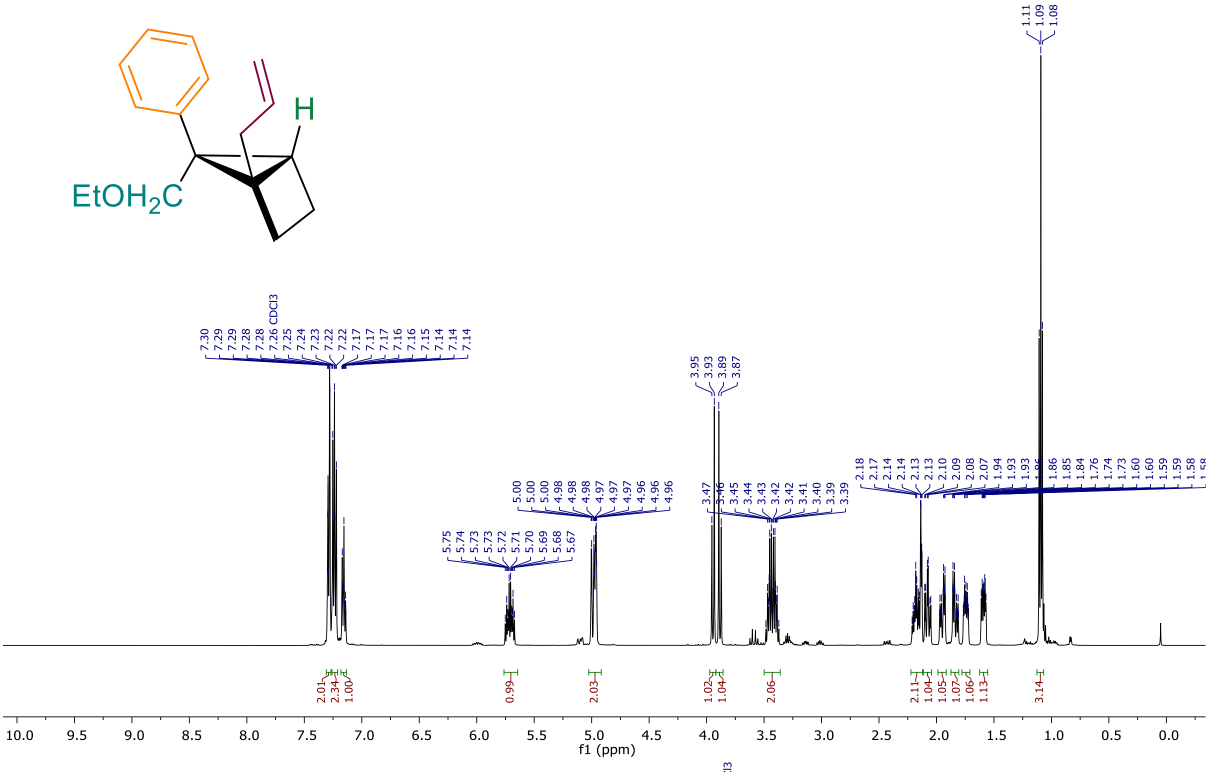
EtOH2C
H
CDCl3
f1 (ppm)

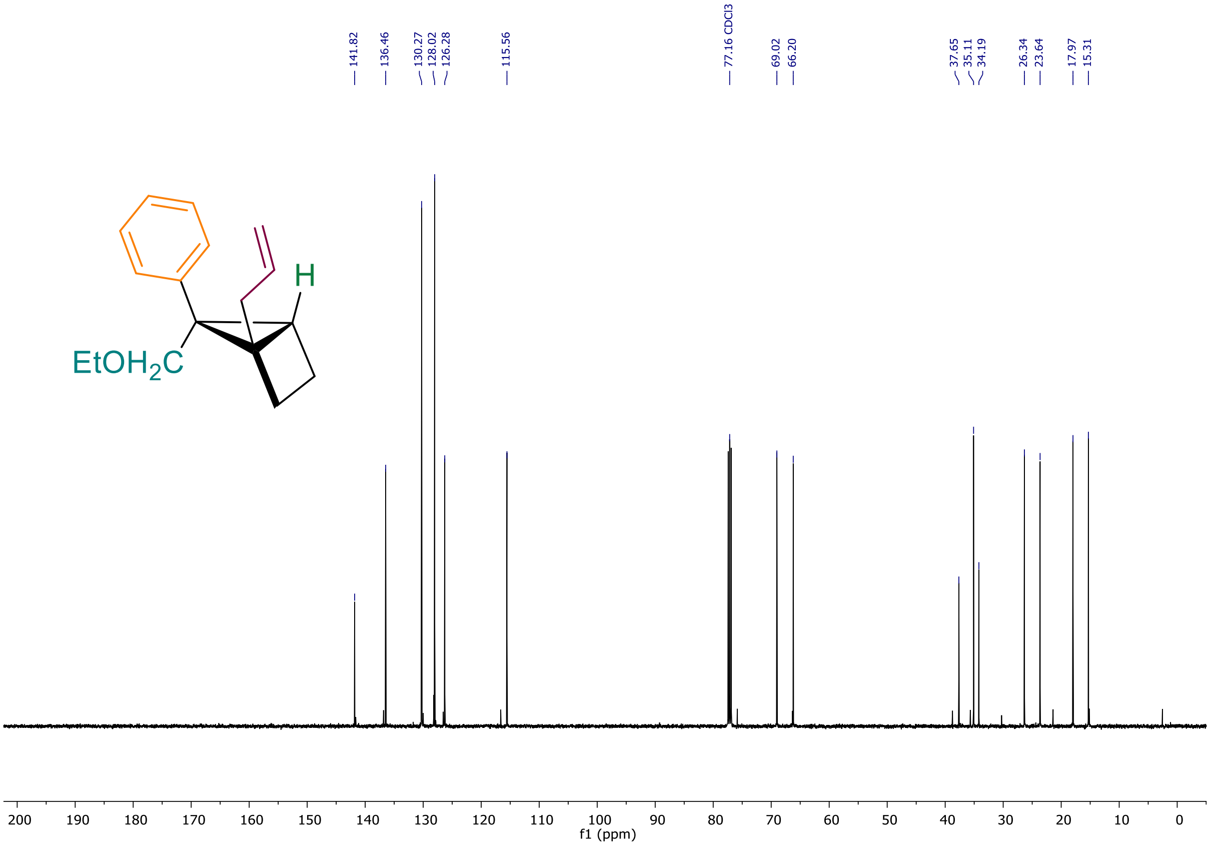
141.82
136.46
130.27
128.02
126.28
115.56
77.16 CDCl3
69.02
66.20
37.65
35.11
34.19
26.34
23.64
17.97
15.31
EtOH2C
H
f1 (ppm)

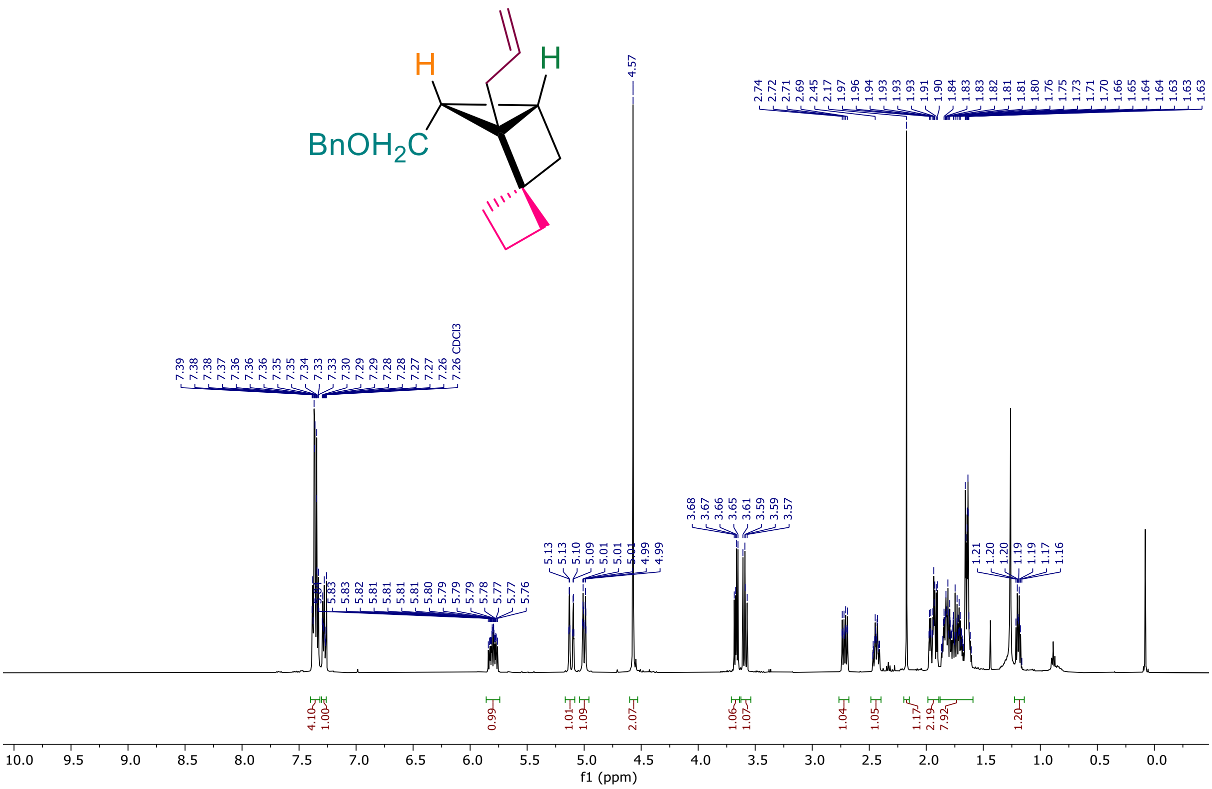
H
H
BnOH2C
7.39 7.38 7.38 7.37 7.36 7.36 7.35 7.35 7.34 7.33 7.33 7.33 7.30 7.29 7.29 7.28 7.28 7.27 7.27 7.26 7.26 CDCl3
5.83 5.83 5.82 5.81 5.81 5.81 5.81 5.80 5.79 5.79 5.79 5.79 5.78 5.77 5.77 5.76
5.13 5.13 5.10 5.09 5.01 5.01 5.01 4.99 4.99
4.57
3.68 3.67 3.66 3.65 3.61 3.59 3.59 3.57
2.74 2.72 2.71 2.69 2.45 2.17 1.97 1.96 1.94 1.93 1.93 1.93 1.91 1.90 1.84 1.83 1.83 1.82 1.81 1.81 1.80 1.76 1.75 1.73 1.71 1.70 1.66 1.65 1.64 1.63 1.63 1.63
1.21 1.20 1.20 1.19 1.19 1.17 1.16
4.10 1.00 0.99 1.01 1.09 2.07 1.06 1.07 1.04 1.05 1.17 2.19 7.92 1.20
10.0 9.5 9.0 8.5 8.0 7.5 7.0 6.5 6.0 5.5 5.0 4.5 4.0 3.5 3.0 2.5 2.0 1.5 1.0 0.5 0.0
f1 (ppm)

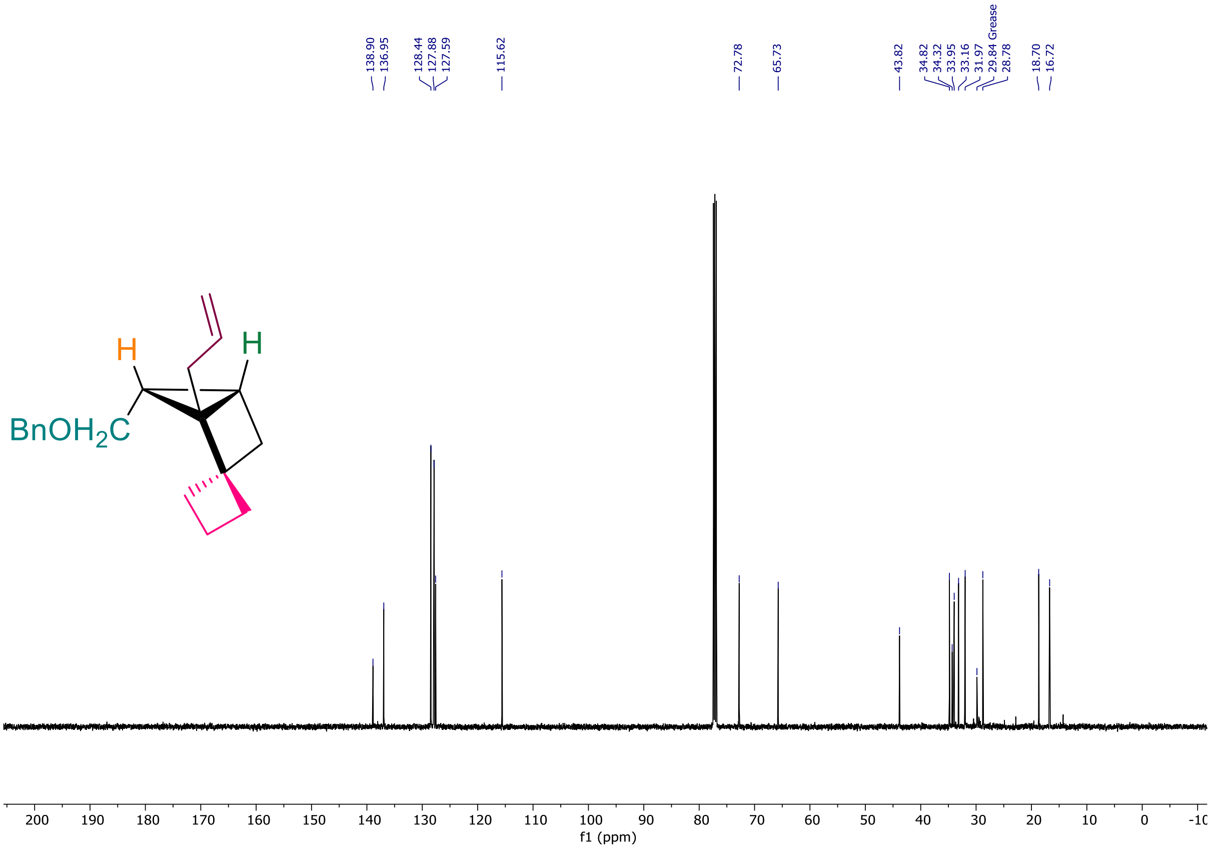
138.90 136.95 128.44 127.88 127.59 115.62 72.78 65.73 43.82 34.82 34.32 33.95 33.16 31.97 29.84 Grease 28.78 18.70 16.72
H
H
BnOH2C
200 190 180 170 160 150 140 130 120 110 100 90 80 70 60 50 40 30 20 10 0 -10
f1 (ppm)

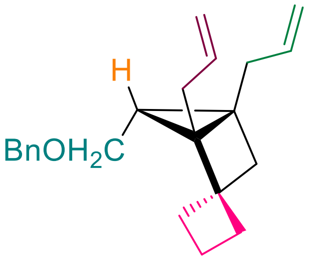
H
BnOH$_2$C

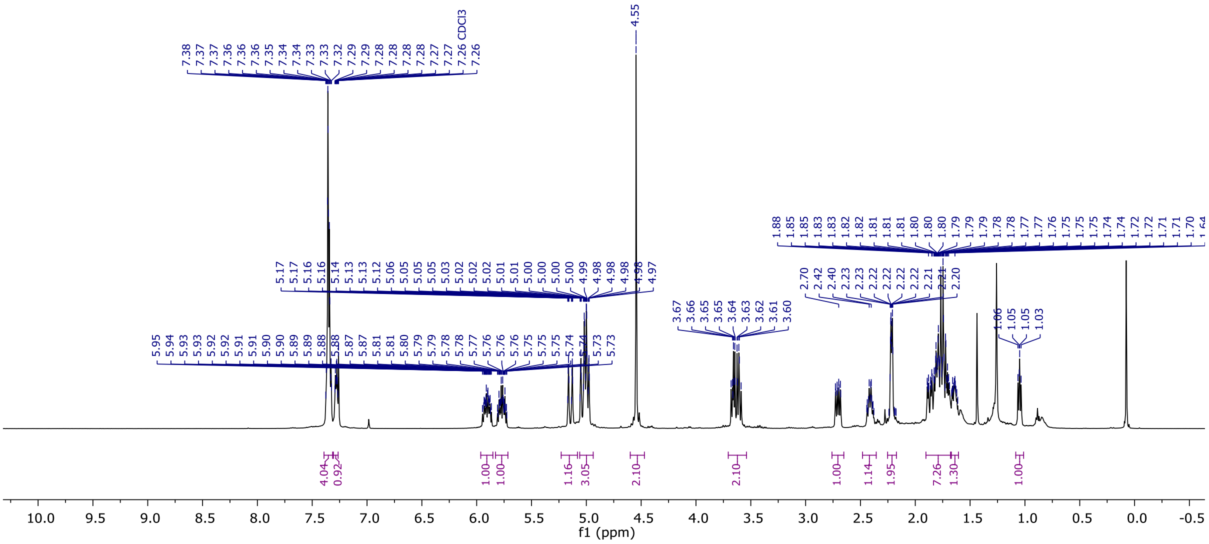
7.38 7.37 7.37 7.36 7.36 7.36 7.35 7.34 7.34 7.33 7.33 7.32 7.29 7.29 7.28 7.28 7.28 7.28 7.27 7.26 CDCl3 7.26
4.55
5.17 5.17 5.16 5.16 5.14 5.13 5.13 5.12 5.06 5.05 5.05 5.05 5.03 5.02 5.02 5.02 5.01 5.01 5.00 5.00 5.00 5.00 4.99 4.98 4.98 4.98 4.98 4.97
5.95 5.94 5.93 5.93 5.92 5.92 5.91 5.91 5.90 5.90 5.89 5.89 5.88 5.88 5.87 5.87 5.81 5.81 5.80 5.79 5.79 5.78 5.78 5.77 5.76 5.76 5.76 5.75 5.75 5.75 5.74 5.74 5.73 5.73
3.67 3.66 3.65 3.65 3.64 3.63 3.62 3.61 3.60
2.70 2.42 2.40 2.23 2.23 2.22 2.22 2.22 2.22 2.22 2.21 2.21 2.20
1.88 1.85 1.85 1.83 1.83 1.82 1.82 1.81 1.81 1.81 1.80 1.80 1.80 1.79 1.79 1.78 1.78 1.77 1.77 1.76 1.75 1.75 1.74 1.74 1.72 1.72 1.71 1.70
1.06 1.05 1.05 1.03
4.04 0.92 1.00 1.00 1.16 3.05 2.10 2.10 1.00 1.14 1.95 7.26 1.30 1.00
10.0 9.5 9.0 8.5 8.0 7.5 7.0 6.5 6.0 5.5 5.0 4.5 4.0 3.5 3.0 2.5 2.0 1.5 1.0 0.5 0.0 -0.5
f1 (ppm)

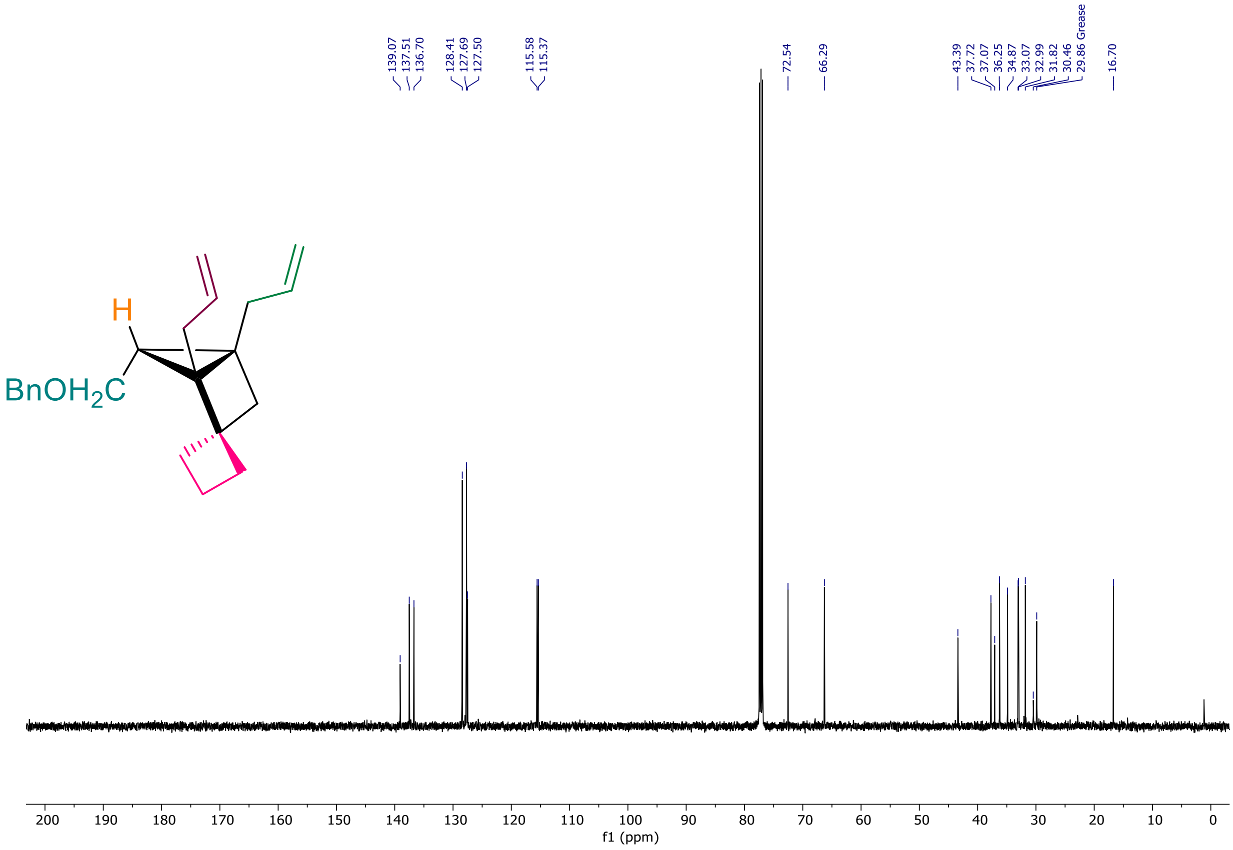
139.07 137.51 136.70
128.41 127.69 127.50
115.58 115.37
72.54
66.29
43.39 37.72 37.07 36.25 34.87 33.07 32.99 31.82 30.46 29.86 Grease
16.70
H
BnOH$_2$C
200 190 180 170 160 150 140 130 120 110 100 90 80 70 60 50 40 30 20 10 0
f1 (ppm)

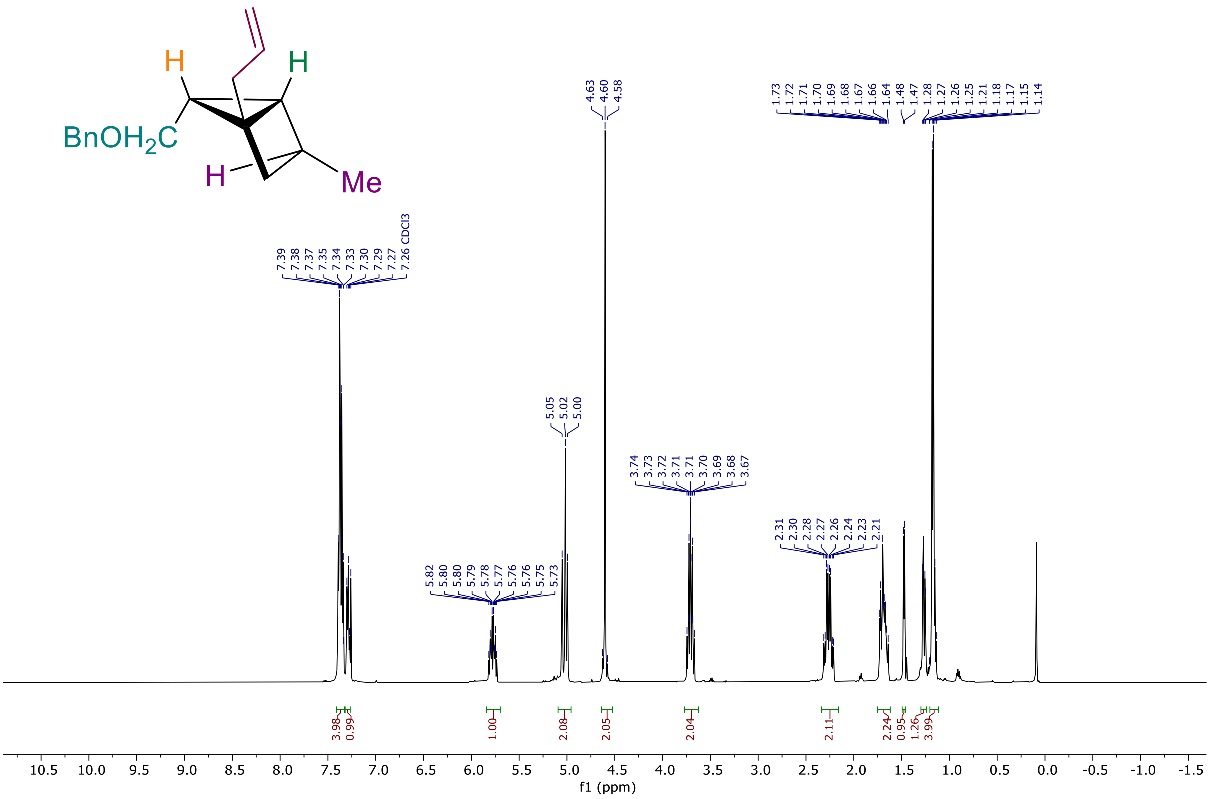
H
H
BnOH$_2$C
H
Me
7.39 7.38 7.37 7.35 7.34 7.33 7.30 7.29 7.27 7.26 CDCl3
5.82 5.80 5.80 5.79 5.78 5.77 5.76 5.76 5.75 5.73
5.05 5.02 5.00
4.63 4.60 4.58
3.74 3.73 3.72 3.71 3.71 3.70 3.69 3.68 3.67
2.31 2.30 2.28 2.27 2.26 2.24 2.23 2.21
1.73 1.72 1.71 1.70 1.69 1.68 1.67 1.66 1.64 1.48 1.47 1.28 1.27 1.26 1.25 1.21 1.18 1.17 1.15 1.14
3.98 0.99 1.00 2.08 2.05 2.04 2.11 2.24 0.95 1.26 3.99
10.5 10.0 9.5 9.0 8.5 8.0 7.5 7.0 6.5 6.0 5.5 5.0 4.5 4.0 3.5 3.0 2.5 2.0 1.5 1.0 0.5 0.0 -0.5 -1.0 -1.5
f1 (ppm)

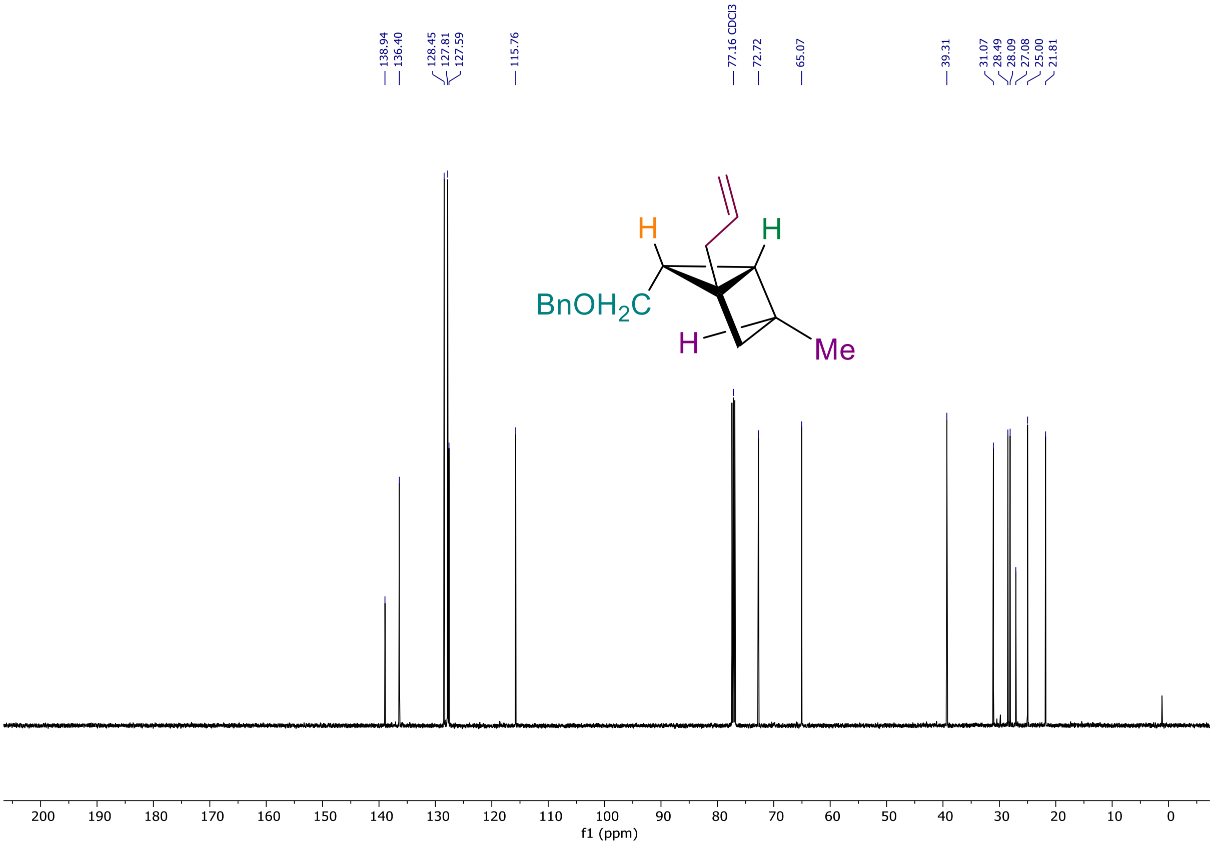
138.94
136.40
128.45 127.81 127.59
115.76
77.16 CDCl3
72.72
65.07
39.31
31.07 28.49 28.09 27.08 25.00 21.81
H
H
BnOH$_2$C
H
Me
200 190 180 170 160 150 140 130 120 110 100 90 80 70 60 50 40 30 20 10 0
f1 (ppm)

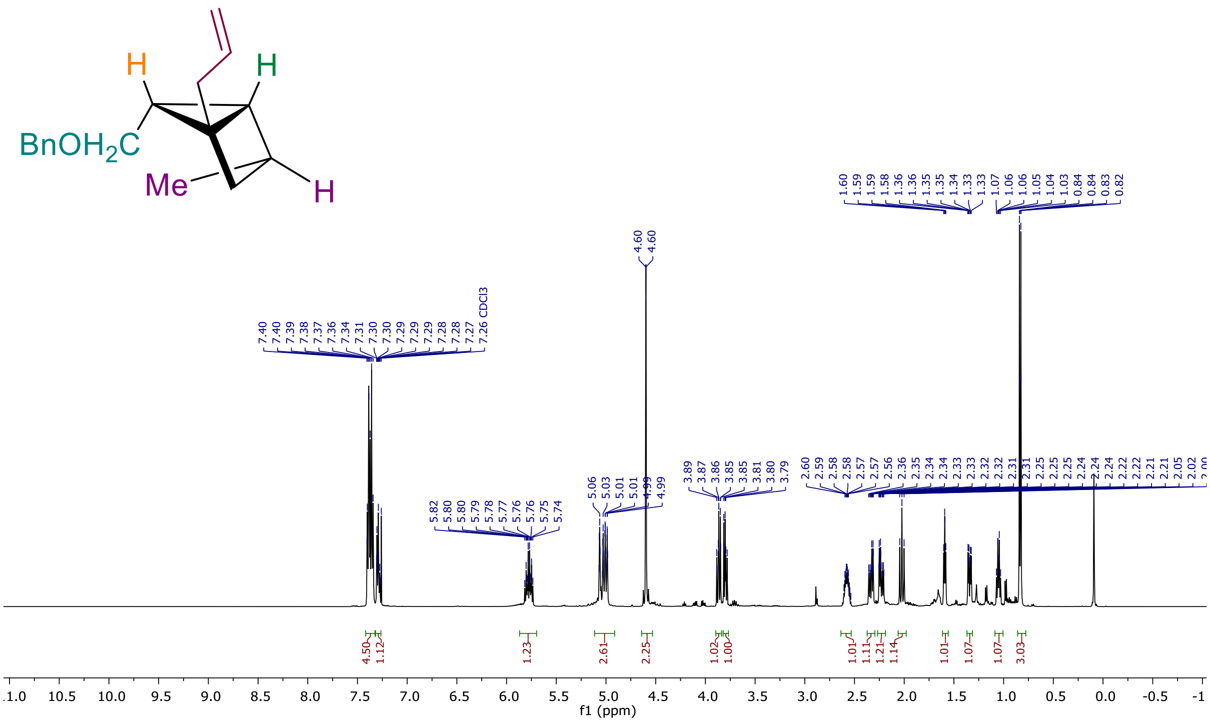
H
H
BnOH$_2$C
Me
H
7.26 CDCl3
4.50
1.12
1.23
2.61
2.25
1.02
1.00
1.01
1.11
1.21
1.14
1.01
1.07
1.07
3.03
f1 (ppm)

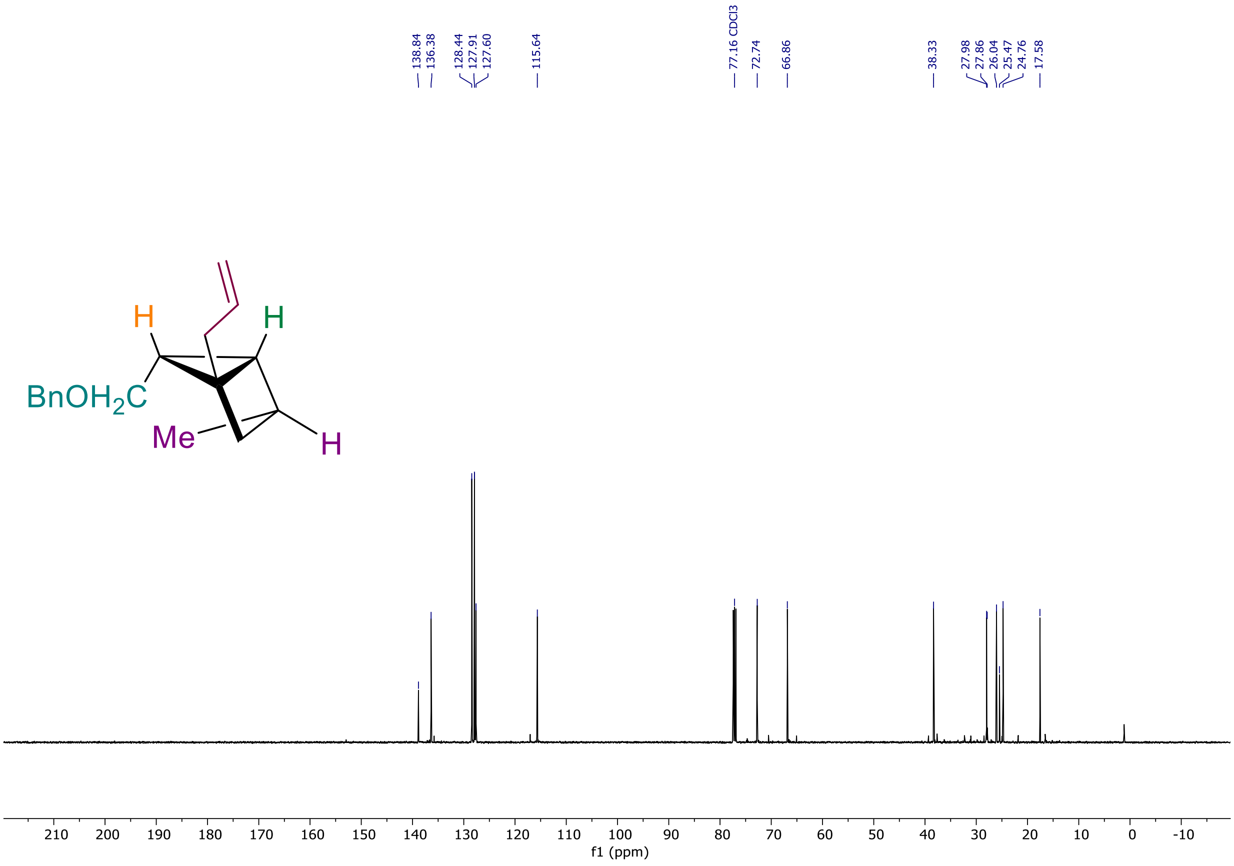
138.84
136.38
128.44
127.91
127.60
115.64
77.16 CDCl3
72.74
66.86
38.33
27.98
27.86
26.04
25.47
24.76
17.58
H
H
BnOH$_2$C
Me
H
f1 (ppm)

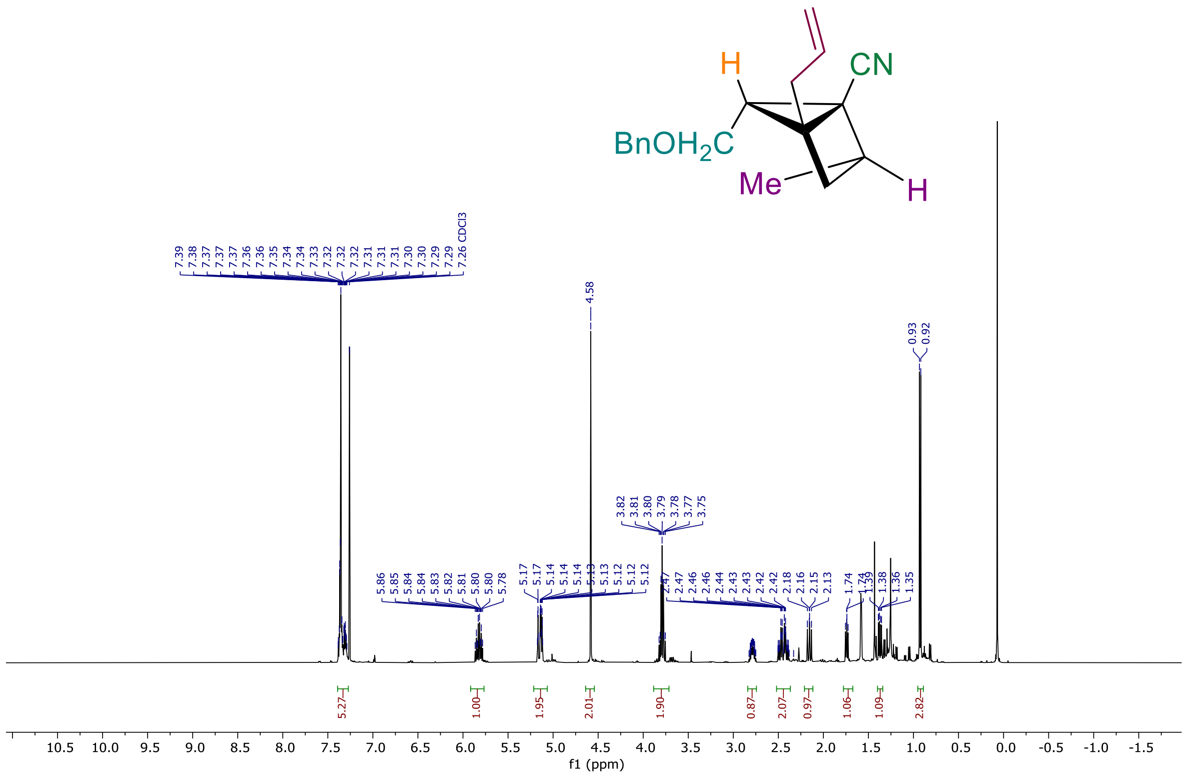
H
CN
BnOH2C
Me
H
7.39 7.38 7.37 7.37 7.37 7.36 7.36 7.35 7.34 7.34 7.33 7.32 7.32 7.32 7.31 7.31 7.31 7.30 7.30 7.29 7.29 7.26 CDCl3
4.58
0.93 0.92
5.86 5.85 5.84 5.84 5.83 5.82 5.81 5.80 5.80 5.78
5.17 5.17 5.14 5.14 5.14 5.13 5.13 5.12 5.12 5.12
3.82 3.81 3.80 3.79 3.78 3.77 3.75
2.47 2.47 2.46 2.44 2.43 2.43 2.42 2.42 2.18 2.16 2.15 2.13
1.74 1.74 1.39 1.38 1.36 1.35
5.27 1.00 1.95 2.01 1.90 0.87 2.07 0.97 1.06 1.09 2.82
10.5 10.0 9.5 9.0 8.5 8.0 7.5 7.0 6.5 6.0 5.5 5.0 4.5 4.0 3.5 3.0 2.5 2.0 1.5 1.0 0.5 0.0 -0.5 -1.0 -1.5
f1 (ppm)

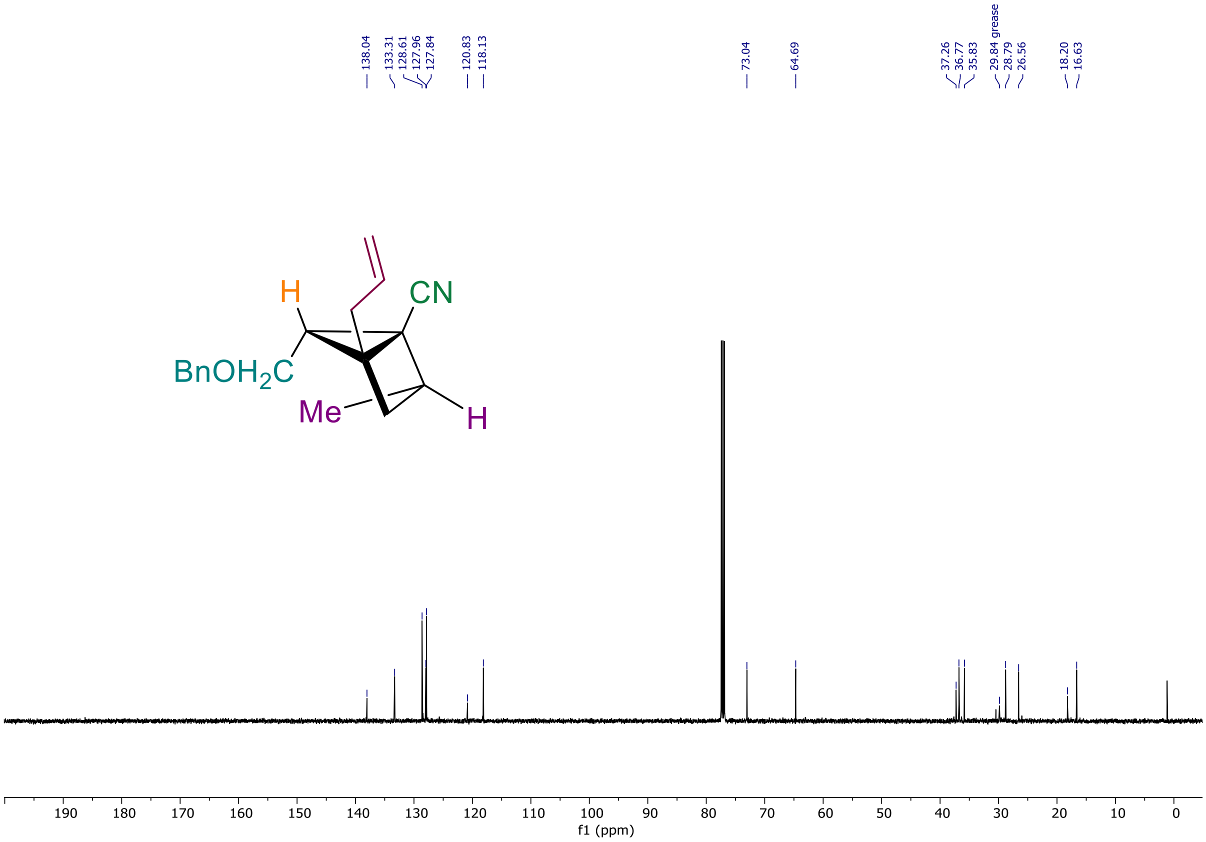
138.04 133.31 128.61 127.96 127.84 120.83 118.13
73.04 64.69
37.26 36.77 35.83 29.84 grease 28.79 26.56
18.20 16.63
H
CN
BnOH2C
Me
H
190 180 170 160 150 140 130 120 110 100 90 80 70 60 50 40 30 20 10 0
f1 (ppm)

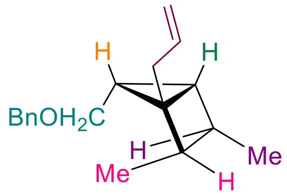
H
H
BnOH2C
H
Me
Me
H

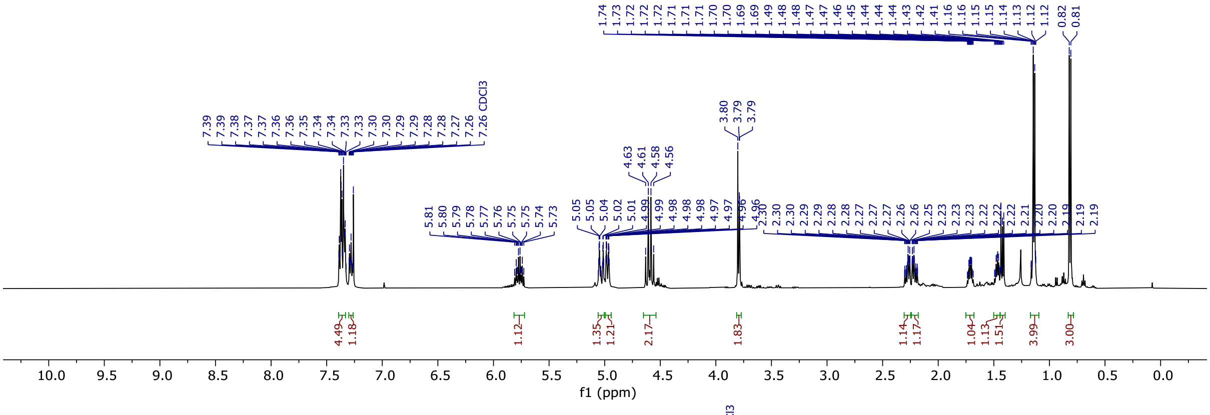
7.39 7.39 7.38 7.37 7.37 7.36 7.36 7.35 7.34 7.34 7.33 7.33 7.30 7.30 7.29 7.29 7.28 7.28 7.27 7.26 7.26 CDCl3
5.81 5.80 5.79 5.78 5.77 5.76 5.75 5.75 5.74 5.73
5.05 5.05 5.04 5.02 5.01 4.99 4.99 4.98 4.98 4.98 4.97 4.97 4.96 4.96
4.63 4.61 4.58 4.56
3.80 3.79 3.79
2.30 2.30 2.30 2.29 2.29 2.28 2.28 2.27 2.27 2.27 2.26 2.26 2.25 2.23 2.23 2.23 2.22 2.22 2.22 2.21 2.20 2.20 2.19 2.19 2.19
1.74 1.73 1.72 1.72 1.72 1.71 1.71 1.71 1.70 1.70 1.69 1.69 1.49 1.48 1.48 1.47 1.47 1.46 1.45 1.44 1.44 1.44 1.43 1.42 1.41 1.16 1.16 1.15 1.15 1.14 1.13 1.13 1.12 1.12
0.82 0.81
4.49 1.18 1.12 1.35 1.21 2.17 1.83 1.14 1.17 1.04 1.13 1.51 3.99 3.00
10.0 9.5 9.0 8.5 8.0 7.5 7.0 6.5 6.0 5.5 5.0 4.5 4.0 3.5 3.0 2.5 2.0 1.5 1.0 0.5 0.0
f1 (ppm)

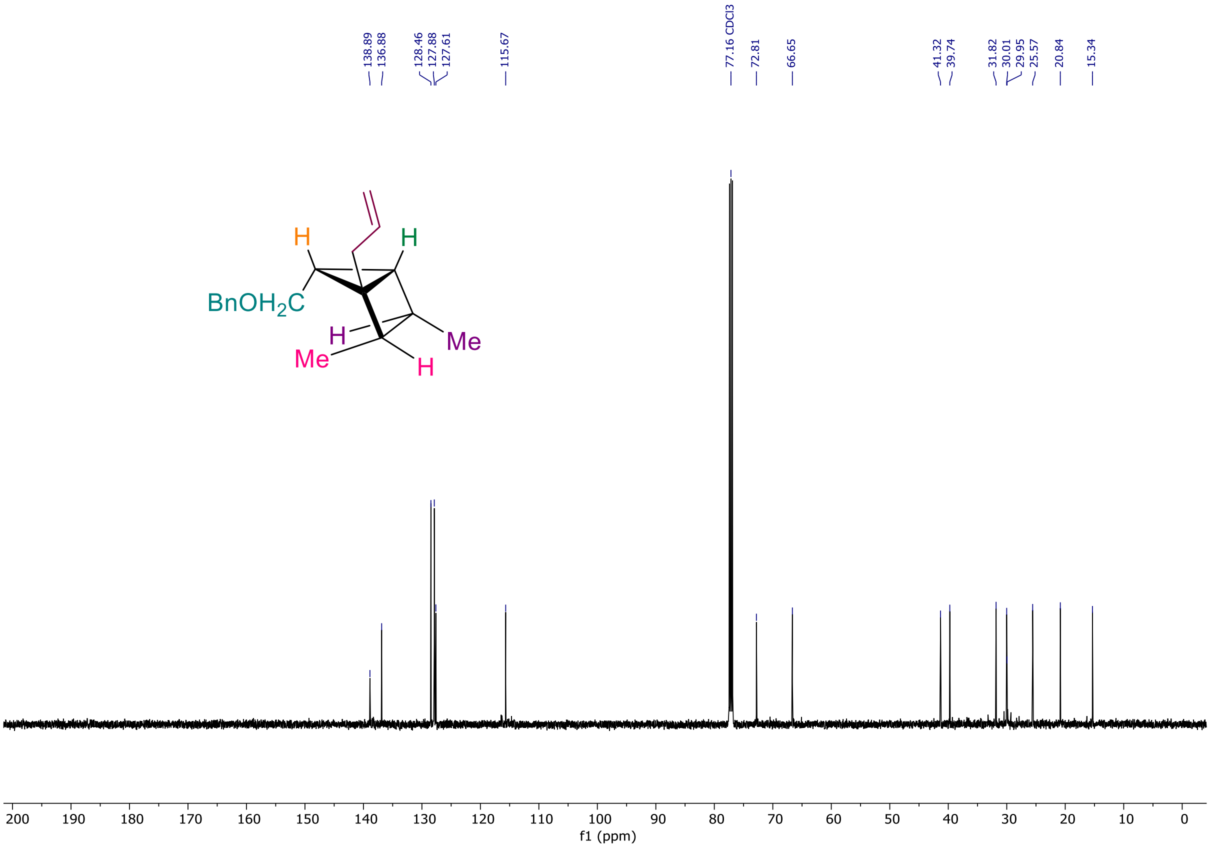
138.89 136.88 128.46 127.88 127.61 115.67 77.16 CDCl3 72.81 66.65 41.32 39.74 31.82 30.01 29.95 25.57 20.84 15.34
H
H
BnOH2C
H
Me
Me
H
200 190 180 170 160 150 140 130 120 110 100 90 80 70 60 50 40 30 20 10 0
f1 (ppm)

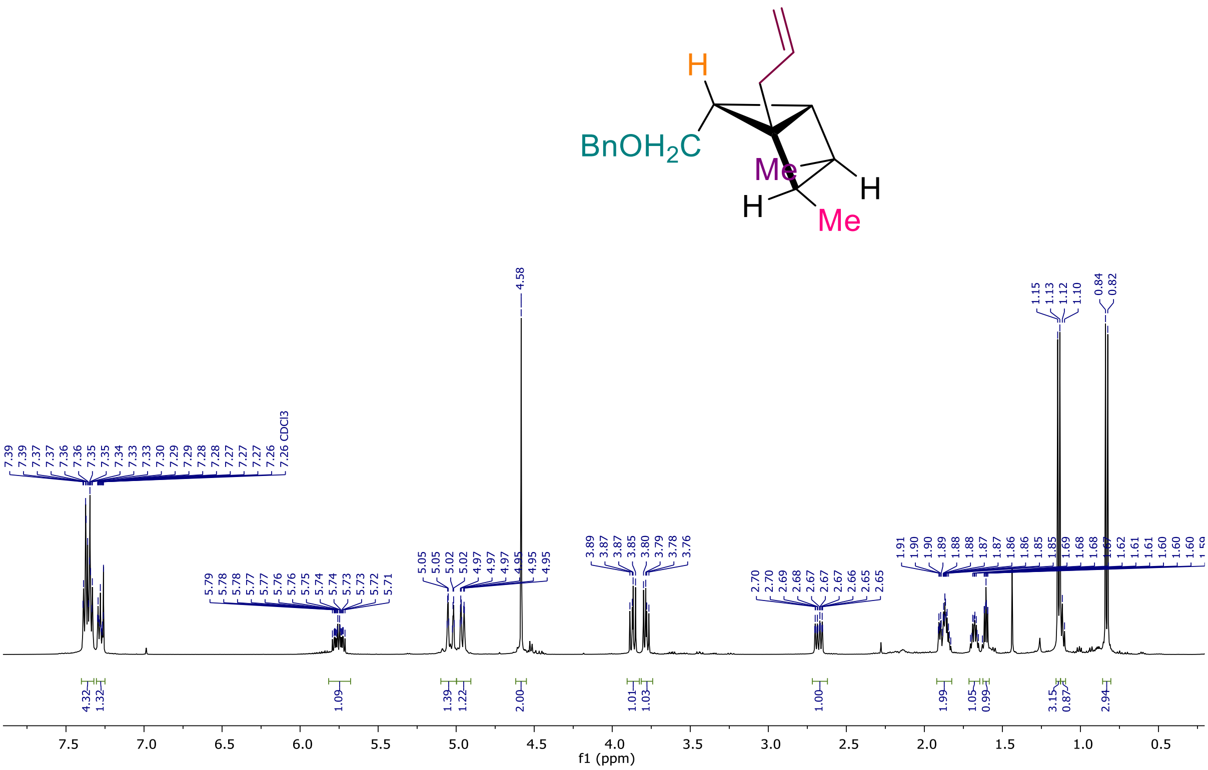

H
BnOH2C
Me
H
H
Me
7.39 7.39 7.37 7.37 7.36 7.35 7.35 7.34 7.33 7.33 7.30 7.29 7.29 7.28 7.28 7.27 7.27 7.27 7.26 7.26 CDCl3
5.79 5.78 5.78 5.77 5.77 5.76 5.76 5.75 5.74 5.74 5.73 5.73 5.72 5.71
5.05 5.05 5.02 5.02 4.97 4.97 4.97 4.95 4.95 4.95
4.58
3.89 3.87 3.87 3.85 3.80 3.79 3.78 3.76
2.70 2.70 2.69 2.68 2.68 2.67 2.67 2.67 2.66 2.65 2.65
1.91 1.90 1.90 1.89 1.88 1.88 1.87 1.87 1.86 1.86 1.85 1.85 1.69 1.68 1.68 1.67 1.62 1.61 1.61 1.60 1.60 1.60
1.15 1.13 1.12 1.10
0.84 0.82
4.32 1.32 1.09 1.39 1.22 2.00 1.01 1.03 1.00 1.99 1.05 0.99 3.15 0.87 2.94
7.5 7.0 6.5 6.0 5.5 5.0 4.5 4.0 3.5 3.0 2.5 2.0 1.5 1.0 0.5
f1 (ppm)

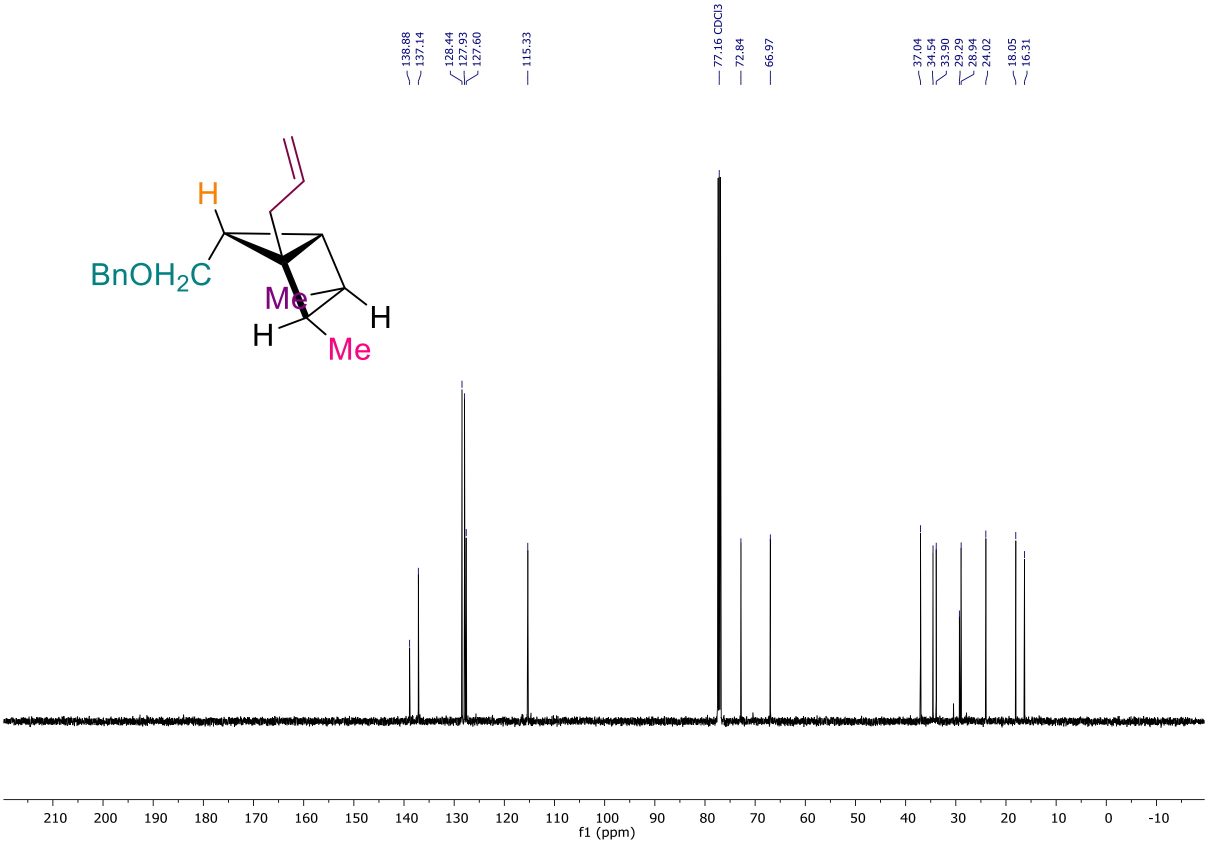

138.88 137.14 128.44 127.93 127.60 115.33
77.16 CDCl3
72.84 66.97
37.04 34.54 33.90 29.29 28.94 24.02 18.05 16.31
H
BnOH2C
Me
H
H
Me
210 200 190 180 170 160 150 140 130 120 110 100 90 80 70 60 50 40 30 20 10 0 -10
f1 (ppm)

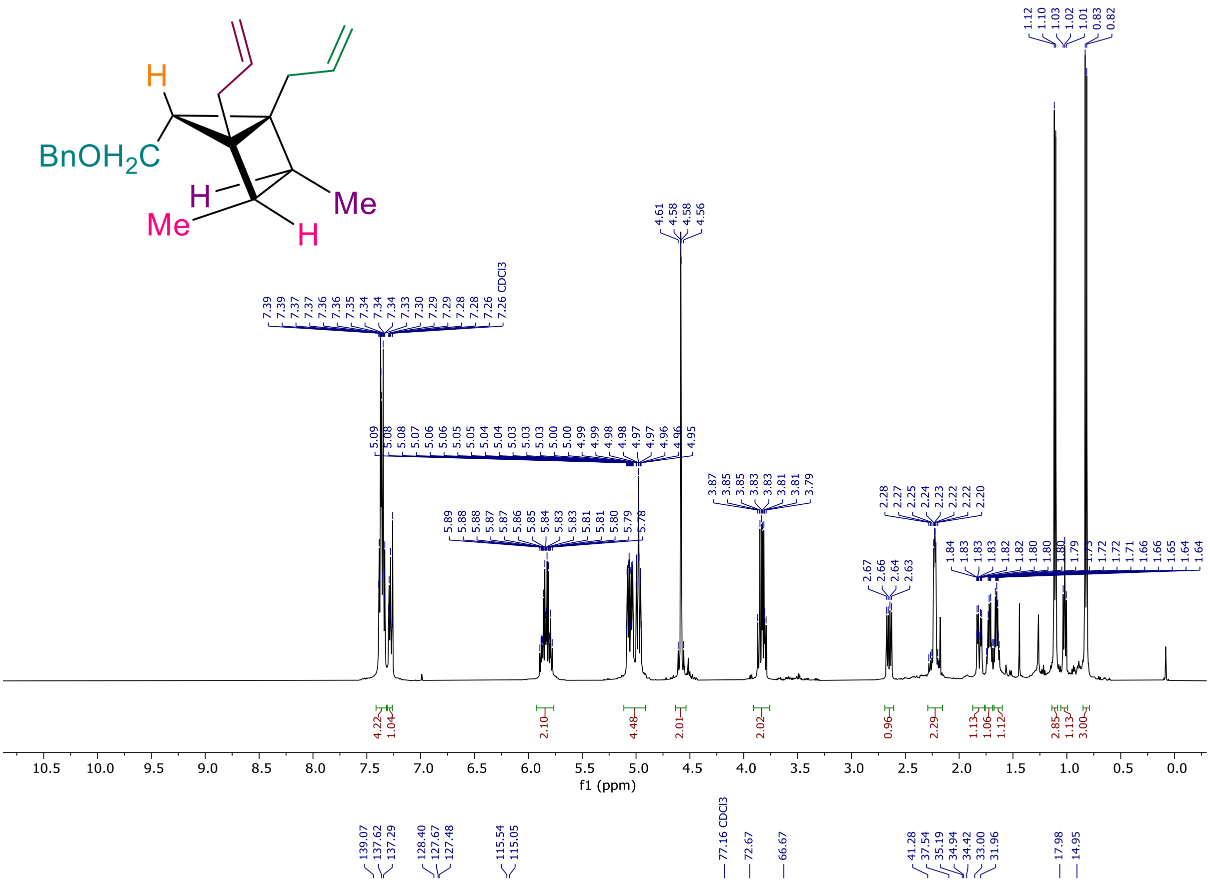

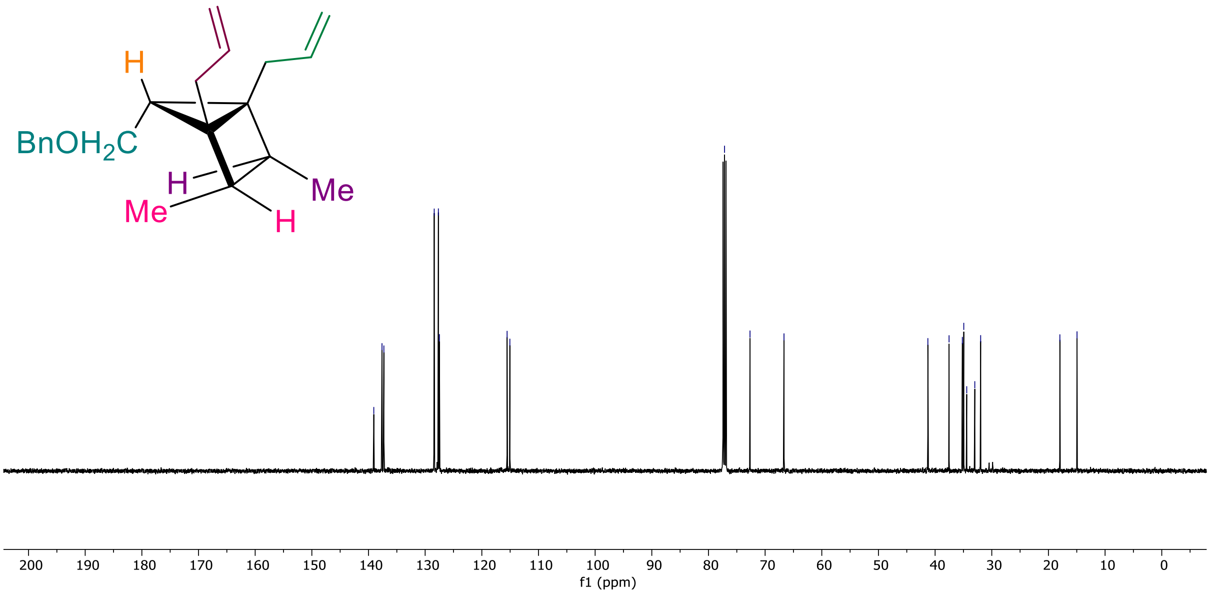

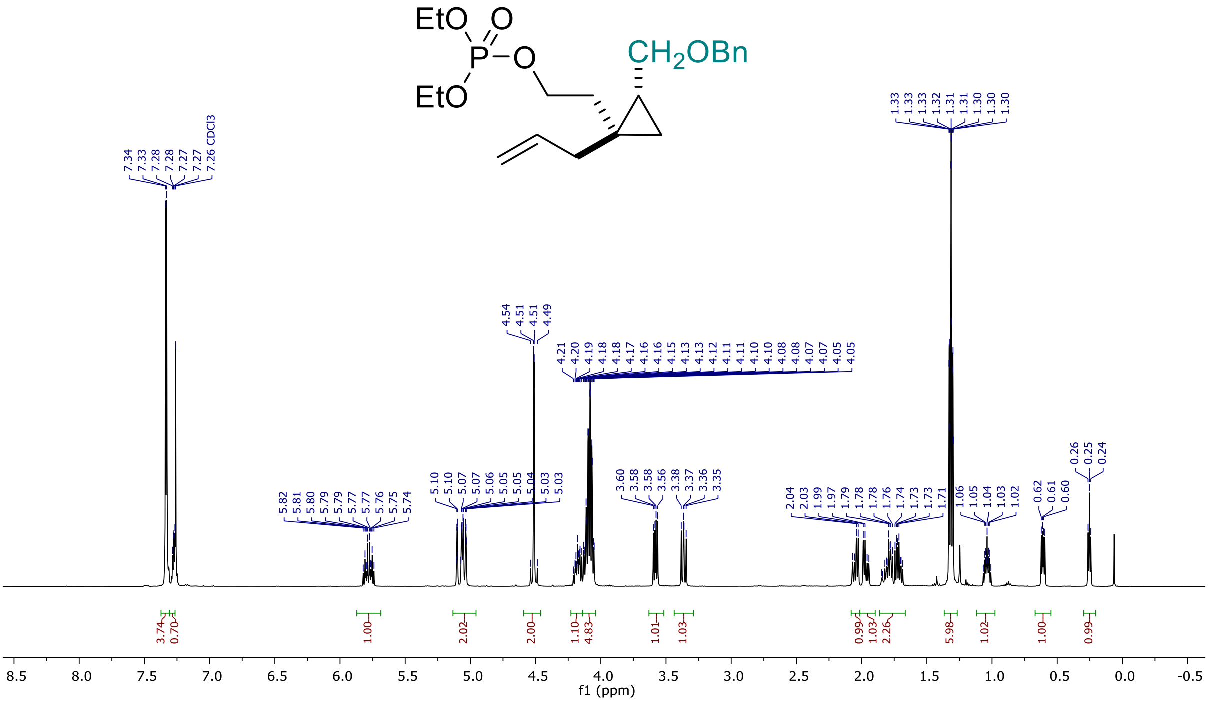
EtO
O
P
O
EtO
CH2OBn
7.34 7.33 7.28 7.28 7.27 7.27 7.26 CDCl3
5.82 5.81 5.80 5.79 5.77 5.77 5.76 5.75 5.74
5.10 5.10 5.07 5.07 5.06 5.05 5.05 5.04 5.03 5.03
4.54 4.51 4.51 4.49
4.21 4.20 4.19 4.18 4.18 4.17 4.16 4.16 4.15 4.13 4.13 4.12 4.11 4.11 4.10 4.10 4.08 4.08 4.07 4.07 4.05 4.05
3.60 3.58 3.58 3.56 3.38 3.37 3.36 3.35
2.04 2.03 1.99 1.97 1.79 1.78 1.76 1.74 1.73 1.73 1.71
1.33 1.33 1.33 1.32 1.31 1.31 1.30 1.30 1.30
1.06 1.05 1.04 1.03 1.02
0.62 0.61 0.60
0.26 0.25 0.24
3.74 0.70 1.00 2.02 2.00 1.10 4.83 1.01 1.03 0.99 1.03 2.26 5.98 1.02 1.00 0.99
8.5 8.0 7.5 7.0 6.5 6.0 5.5 5.0 4.5 4.0 3.5 3.0 2.5 2.0 1.5 1.0 0.5 0.0 -0.5
f1 (ppm)

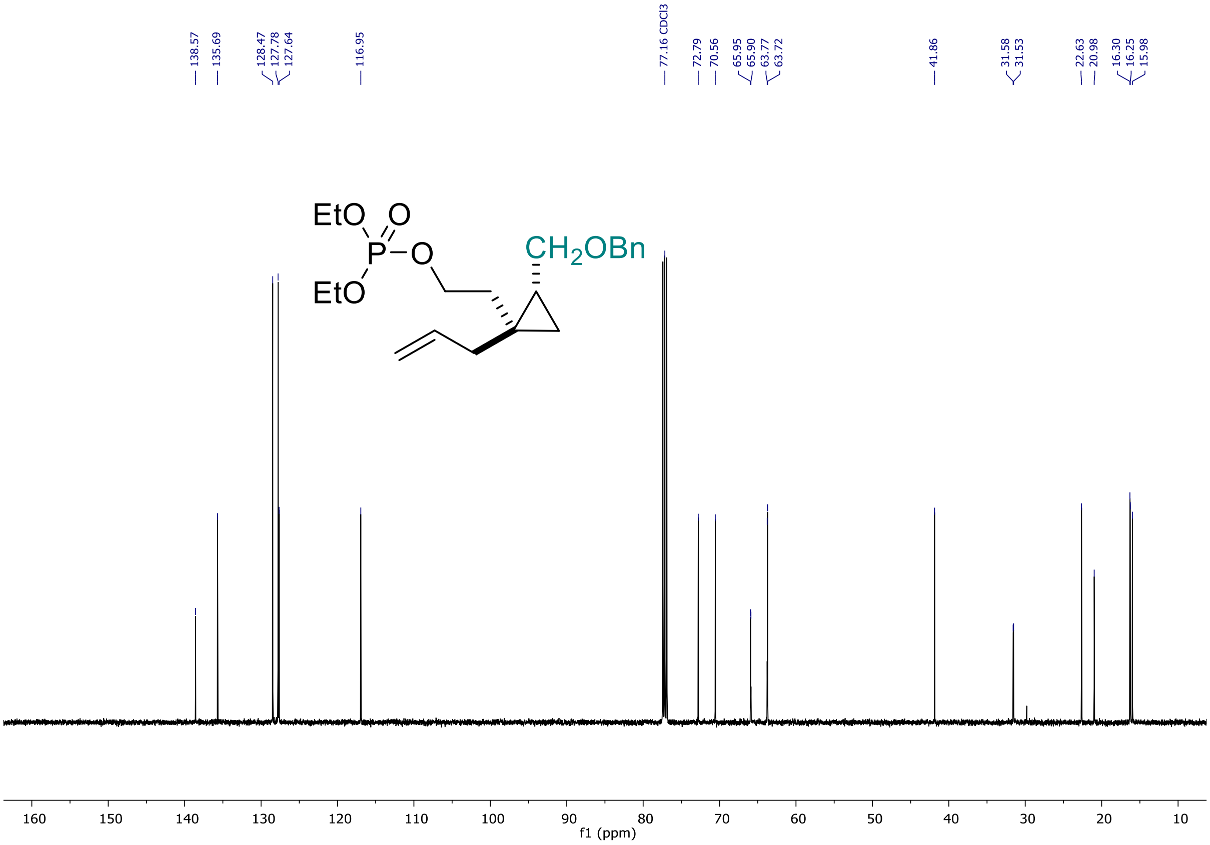
138.57 135.69 128.47 127.78 127.64 116.95 77.16 CDCl3 72.79 70.56 65.95 65.90 63.77 63.72 41.86 31.58 31.53 22.63 20.98 16.30 16.25 15.98
EtO
O
P
O
EtO
CH2OBn
160 150 140 130 120 110 100 90 80 70 60 50 40 30 20 10
f1 (ppm)

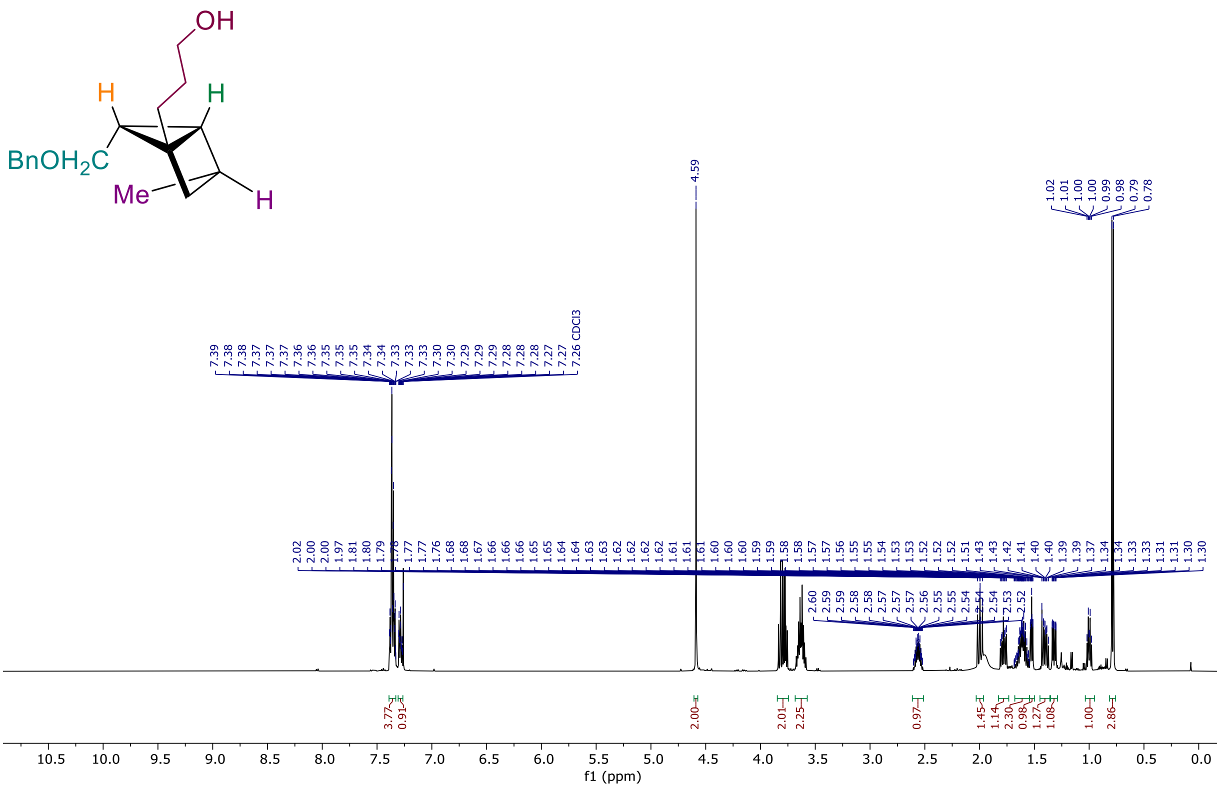

OH
H
H
BnOH2C
Me
H
4.59
7.26 CDCl3
3.77
0.91
2.00
2.01
2.25
0.97
1.45
1.14
2.30
0.98
1.27
1.08
1.00
2.86
f1 (ppm)

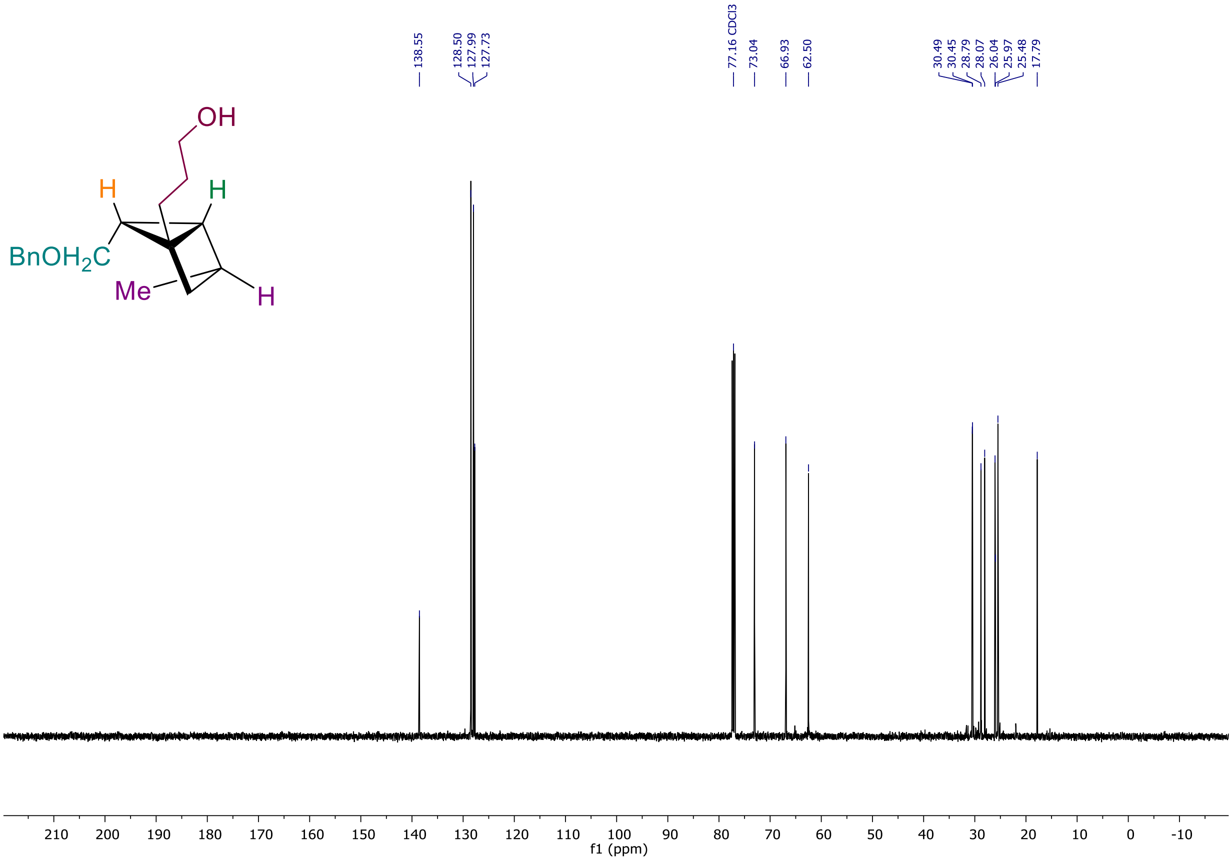

OH
H
H
BnOH2C
Me
H
138.55
128.50
127.99
127.73
77.16 CDCl3
73.04
66.93
62.50
30.49
30.45
28.79
28.07
26.04
25.97
25.48
17.79
f1 (ppm)

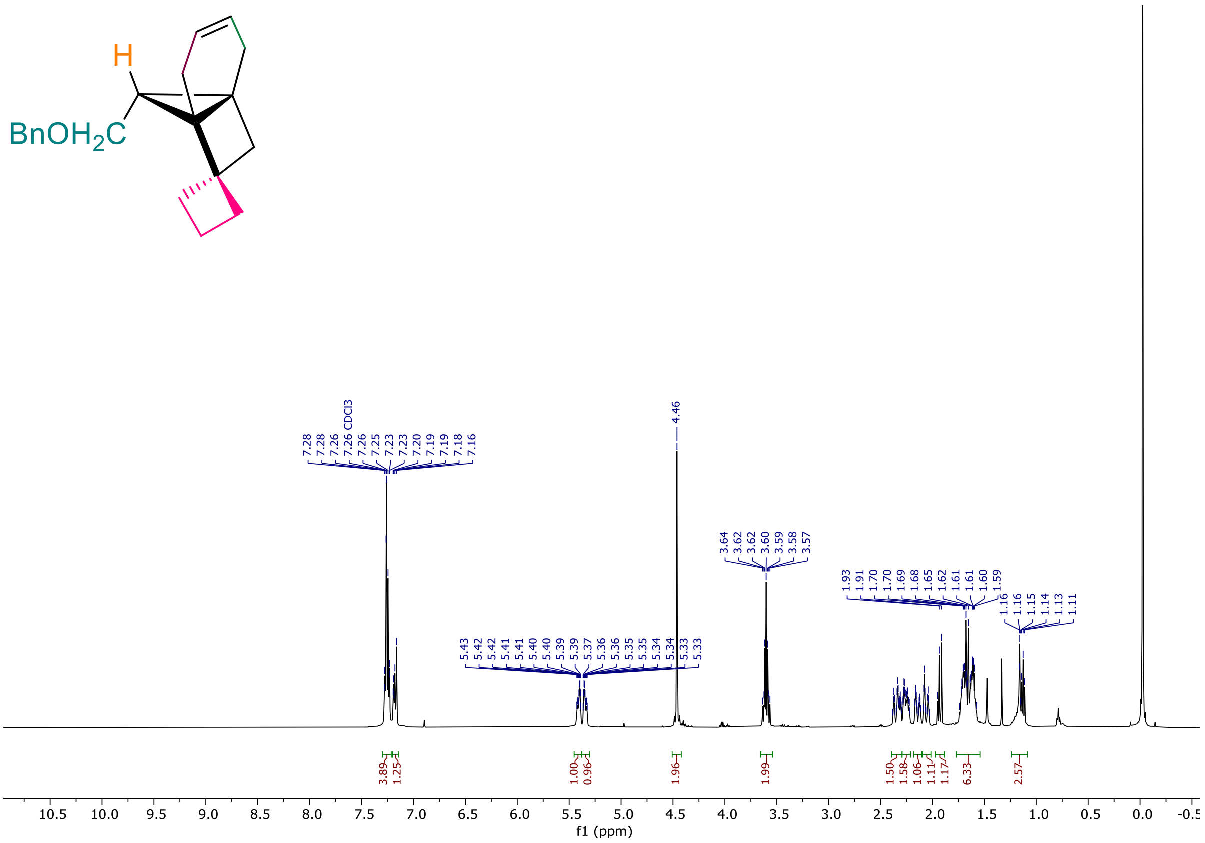
H
BnOH2C
7.28
7.28
7.26
7.26 CDCl3
7.26
7.25
7.23
7.23
7.20
7.19
7.19
7.18
7.16
4.46
5.43
5.42
5.42
5.41
5.41
5.40
5.40
5.39
5.39
5.37
5.36
5.36
5.35
5.35
5.34
5.34
5.33
5.33
3.64
3.62
3.62
3.60
3.59
3.58
3.57
1.93
1.91
1.70
1.70
1.69
1.68
1.65
1.62
1.61
1.61
1.60
1.59
1.16
1.16
1.15
1.14
1.13
1.11
3.89
1.25
1.00
0.96
1.96
1.99
1.50
1.58
1.06
1.11
1.17
6.33
2.57
f1 (ppm)

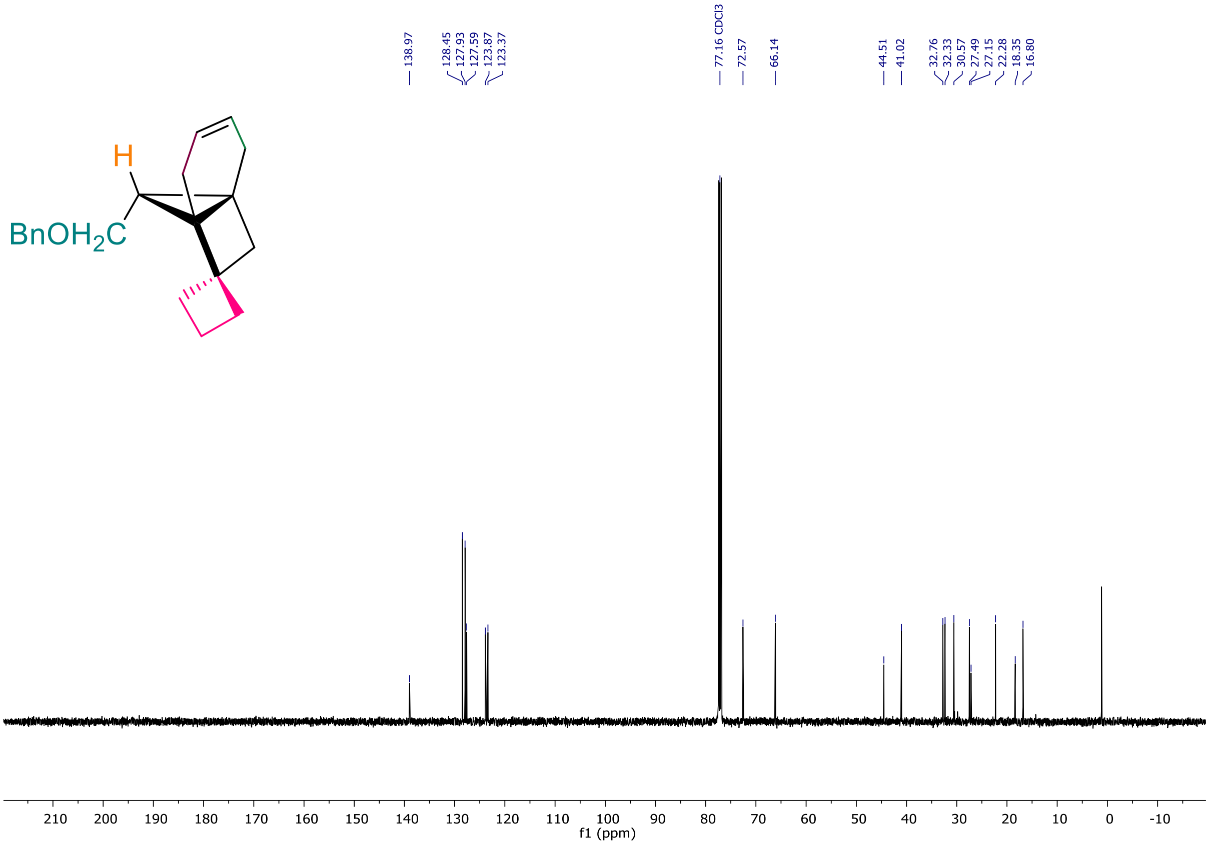
138.97
128.45
127.93
127.59
123.87
123.37
77.16 CDCl3
72.57
66.14
44.51
41.02
32.76
32.33
30.57
27.49
27.15
22.28
18.35
16.80
H
BnOH2C
f1 (ppm)
